# ERBB2 and KRAS alterations mediate response to EGFR inhibitors in early stage gallbladder cancer

**DOI:** 10.1002/ijc.31916

**Published:** 2018-12-08

**Authors:** Prajish Iyer, Shailesh V. Shrikhande, Malika Ranjan, Asim Joshi, Nilesh Gardi, Ratnam Prasad, Bhasker Dharavath, Rahul Thorat, Sameer Salunkhe, Bikram Sahoo, Pratik Chandrani, Hitesh Kore, Bhabani Mohanty, Vikram Chaudhari, Anuradha Choughule, Dhananjay Kawle, Pradip Chaudhari, Arvind Ingle, Shripad Banavali, Poonam Gera, Mukta R. Ramadwar, Kumar Prabhash, Savio George Barreto, Shilpee Dutt, Amit Dutt

**Affiliations:** ^1^ Integrated Cancer Genomics Laboratory Advanced Centre for Treatment Research Education in Cancer (ACTREC), Tata Memorial Centre Navi Mumbai Maharashtra India; ^2^ Homi Bhabha National Institute Mumbai Maharashtra India; ^3^ Department of Gastrointestinal and Hepato‐Pancreato‐Biliary Surgical Oncology Tata Memorial Centre, Ernest Borges Marg Mumbai Maharashtra India; ^4^ Laboratory Animal Facility Advanced Centre for Treatment, Research and Education in Cancer, Tata Memorial Centre Navi Mumbai Maharashtra India; ^5^ Shilpee laboratory Advanced Centre for Treatment Research Education In Cancer (ACTREC), Tata Memorial Centre Navi Mumbai Maharashtra India; ^6^ Small Animal Imaging facility Advanced Centre for Treatment Research Education In Cancer (ACTREC), Tata Memorial Centre Navi Mumbai Maharashtra India; ^7^ Department of Medical Oncology Tata Memorial Centre, Ernest Borges Marg Mumbai Maharashtra India; ^8^ Tissue Biorepository Advanced Centre for Treatment Research and Education in Cancer (ACTREC), Tata Memorial Centre Navi Mumbai Maharashtra India; ^9^ Department of Pathology Tata Memorial Centre, Ernest Borges Marg Mumbai Maharashtra India

**Keywords:** gallbladder cancer, whole exome sequencing, ErbB pathway, KRAS mutation, targeted therapy

## Abstract

The uncommonness of gallbladder cancer in the developed world has contributed to the generally poor understanding of the disease. Our integrated analysis of whole exome sequencing, copy number alterations, immunohistochemical, and phospho‐proteome array profiling indicates *ERBB2* alterations in 40% early‐stage rare gallbladder tumors, among an ethnically distinct population not studied before, that occurs through overexpression in 24% (*n* = 25) and recurrent mutations in 14% tumors (*n* = 44); along with co‐occurring *KRAS* mutation in 7% tumors (*n* = 44). We demonstrate that ERBB2 heterodimerizes with EGFR to constitutively activate the ErbB signaling pathway in gallbladder cells. Consistent with this, treatment with *ERBB2*‐specific, *EGFR*‐specific shRNA or with a covalent EGFR family inhibitor Afatinib inhibits tumor‐associated characteristics of the gallbladder cancer cells. Furthermore, we observe an *in vivo* reduction in tumor size of gallbladder xenografts in response to Afatinib is paralleled by a reduction in the amounts of phospho‐ERK, in tumors harboring *KRAS* (G13D) mutation but not in *KRAS* (G12V) mutation, supporting an essential role of the ErbB pathway. In overall, besides implicating *ERBB2* as an important therapeutic target under neo‐adjuvant or adjuvant settings, we present the first evidence that the presence of *KRAS* mutations may preclude gallbladder cancer patients to respond to anti‐EGFR treatment, similar to a clinical algorithm commonly practiced to opt for anti‐EGFR treatment in colorectal cancer.

## Abstract 2

**What's new?**

This study presents the first genomic landscape of early‐stage gallbladder cancer among an understudied ethnic population. Besides suggesting anti‐EGFR therapy as a therapeutic option based on ERBB2 alteration, the evidence suggests that presence of KRAS (G12V) but not KRAS (G13D) mutation may impede treatment response. The findings could potentially lead to early adoption of a clinical algorithm to treat gallbladder cancer patients under neo‐adjuvant or adjuvant settings similar to the one commonly used for anti‐EGFR treatment in colorectal cancer. Furthermore, the findings rationalize inclusion of gallbladder patients under genomically matched basket clinical trials such as the NCI‐Molecular Analysis for Therapy Choice.

**Abbreviations**
BCA: Bicinchoninic acid assay
NCI‐MATCH: NCI‐Molecular Analysis for Therapy Choice
PET‐CT: Positron Emission Tomography‐Computed Tomography
RTK: Receptor tyrosine kinases
SGOL: Segment‐of‐Gain‐Or‐Loss
SPSS: Statistical Package for Social Sciences
TKI: Tyrosine Kinase Inhibitors
TMH‐TTR: Tumor Tissue repository of Tata Memorial Hospital

## Introduction

Genomically matched therapies targeting activated tyrosine kinases have shown promise across multiple cancer types.^1^ The success of tyrosine kinase inhibitors (TKIs) such as imatinib, a BCR‐ABL fusion protein inhibitor^2^; vemurafenib, a RAF inhibitor^3^; lapatinib, an inhibitor of ERBB2^4^; erlotinib and crizotinib, inhibitors of EGFR and ALK, respectively^5^, ^6^; and, others have provided a powerful validation for precision cancer medicine. Although these treatments offer great promise, selective genomic profiling of tumors tends to impede broader implementation of genome‐based cancer care.^7^ For example, an inadequacy to account for multiple relevant genetic alterations likely resulted in comparable outcomes in a recently performed randomized trial where multiple cancer type patients were profiled for selected driver alterations and randomized to receive genomically‐matched *versus* conventional therapy.^8^ Such important clinical studies underscore the need for convergence of information for multiple genetic alterations to ensure the success of future clinical trial designs, with specific emphasis for consideration of co‐occurring alterations that could potentially render tumors unlikely to benefit from genomically‐matched treatments. Some prototypical examples include *KRAS*, *NRAS*, and *BRAF* mutations in colorectal cancers or secondary *EGFR* mutations in lung cancer against anti‐ EGFR targeted therapies.^9^

The EGFR family of receptor tyrosine kinases (RTK) consists of *EGFR*, *HER2*, *HER3* and *HER4* (human EGFR‐related‐ 2, −3, and − 4). A ligand‐bound EGFR family member forms a homo‐ or hetero‐dimer to activate the PI3K‐AKT–mTOR or RAS–RAF‐MAPK downstream signaling pathway to evade apoptosis and enhance cell proliferation.^10^ Interestingly, of all EGFR family members, HER2 lacks a ligand binding domain and forms preferred partner for other members to heterodimerize even in the absence of ligand.^11^ Deregulation of EGFR family RTK‐signaling network endows tumor cells with attributes to sustain their malignant behavior and survival, as is frequently observed in breast cancer, lung cancer, pancreatic cancer, head and neck cancer and colorectal cancer.^12^ Interfering with the EGFR pathway thus forms the basis for the development of targeted anticancer therapies such as RTK‐targeted antibodies (Cetuximab and Herceptin) and small‐molecule inhibitors of RTK kinase (Erlotinib, Lapatinib, Afatinib, etc.) that have shown a dramatic clinical response.^12^ In such responses, however, the co‐occurrence of a *KRAS* mutation – a downstream component of the pathway‐‐ preclude patients from anti‐EGFR treatment in colorectal cancer, wherein *KRAS* codon 12, but not codon 13 mutations are associated with poor outcomes,^13^ underscoring their prognostic impact.

Gallbladder cancer, the most common malignancy of biliary tract, is a rare form of cancer in the world where chemotherapy and other palliative treatments have little effect on the overall survival of patients.^14^ The poor understanding of gallbladder cancer due to its uncommonness in the western world but high prevalence in Chile and the Indian subcontinent lends itself to the need for further research.^15^ While the 5‐year survival rate of an early stage T1 gallbladder carcinoma is nearly 100%, it significantly decreases as the disease progresses, with less than 15% for T3/T4 advanced stage tumors.^16^ A hope for longer‐term survival has specifically been promising for an early stage T2 carcinomas with an intermediate 5‐year survival.^17^ Literature suggests *HER2* overexpression in 12–15% of advanced stage gallbladder cancers with a favorable response to HER2 directed therapy.^18^, ^19^ Moreover, few recent studies analyzed whole exome sequence of advanced stage gallbladder tumors with consistent findings.^15^, ^19^, ^20^, ^21^ In order to understand the landscape of somatic alterations among a clinically distinct early staged pT1/pT2 gallbladder cancer patients, we performed whole exome sequencing of 17 early staged tumor‐normal paired gallbladder samples, 5 gallbladder cancer cell lines followed by validation in 27 additional tumor samples. Here, we report novel somatic mutations of *ERBB2* in gallbladder cancer, and its therapeutic implication in the presence and absence of *KRAS* (G12 V) and (G13D) mutations.

## Materials and Methods

### Patient information

A total of 27 fresh frozen samples (10 tumor‐normal paired and 7 orphan tumors) were utilized for whole exome sequencing. An additional set of 27 FFPE samples were utilized as a validation set. Tumor‐normal paired samples were collected at Tata Memorial Hospital and Advanced Centre for Treatment, Research and Education in Cancer (ACTREC), Mumbai. (ACTREC‐TMC) Internal Review Board (IRB) ‐‐IRB Project Number # 104‐‐ approved study protocols. Formalin‐fixed paraffin‐embedded tissue blocks were collected from the tissue repository of Tata memorial hospital (TMH‐TTR) in compliance with the guidelines. These tissues were examined for tumor content and the tumor content was in the range of 40–90%. Patient samples and characteristics are provided in the Supporting Information Table S1 and S5.

### Data description

Whole exome sequence data (150 bp paired end reads) from a rare set of early‐staged 27 fresh frozen gallbladder samples (with tumor content in the range of 40–90%) were generated for our study with coverage of >100×, using Illumina platform, to analyze tumor specific somatic mutations and copy number alterations. Paired‐end raw sequence reads were mapped to human reference genome (build hg19) using BWA v. 0.6.2.^22^ Quality control analysis of bam files were carried out using qualimap (v0.7.1),^23^ followed by base quality score recalibration and indel re‐alignment to call variants from each sample separately using GATK Unified Genotyper (version 2.5–2).^24^ For copy number estimation, the BAM files prepared for variant calling were used using Control‐FREEC.^25^ The read count ratio were converted to copy numbers followed by segmentation using lasso method. Segmented copy number data generated by control‐FREEC was further used for annotation and post‐processing using R programming to infer SGOL score to help rank the region and define cut‐off for downstream analysis. The raw datasets are available from the ArrayExpress database (accession number E‐MTAB‐6619).

### DNA extraction

Genomic DNA was extracted from fresh frozen samples by using Qiagen Blood and Cell culture DNA kit. The extracted DNA yield and quality were assessed using Nanodrop ND2000 (Thermo scientific). The extracted DNA (about 1 μg) from the fresh‐frozen tissue specimens were sent to Genotypic Technology Pvt Ltd, Bangalore for exome sequencing. Genomic DNA from FFPE blocks was extracted using Qiagen QiAmp DNA FFPE Tissue kit as per manufacturer instructions. The extracted DNA yield and quality were assessed using Nanodrop ND2000 (Thermo scientific). These samples were further checked for integrity by PCR amplification of GAPDH (96 bp). These samples were used for extended Sanger validation of identified variants in exome sequencing.

### Exome analysis pipeline and somatic mutation calling

The variant analysis was performed as described previously.^26^, ^27^ MutSigCV v2.0^28^ and IntOgen^29^ were used for identification of the significantly mutated gene and *p* value ≤0.05 was considered as the threshold for significance. The variants were excluded if they were present in exclusively in dbSNP, TMC‐SNPdb or both. Also, we removed variants that were identified in all three databases – COSMIC (v68),^30^ dbSNP (v142)^31^ and TMC‐SNPdb database.^27^ The annotated cancer‐associated variants were annotated using Oncotator (v1.1.6.0)^32^ and restricted our further analysis to only coding variants. Intogen (https://www.intogen.org/search) was used to calculate the significance of frequently mutated gene in our cohort. Since our dataset was inherently not suitable for above tools due to limited number of tumor samples (*n* = 17), we have also performed extensive functional prediction tool based analysis for nonsynonymous variants using nine different tools as described earlier.^26^ Total number of identified somatic substitutions in exome sequencing was extracted from MutSigCV output and was processed to calculate the number and frequency distribution of various transitions and transversions.

### Exome sequencing capture, library construction, and sequencing

Exome capture and sequencing were performed as described previously.^33^ Briefly, Agilent Sure select in‐solution (low‐input capture‐500 ng) were used to capture ~62 Mb region of human genome comprising of ~201,121 exons representing ~20,974 gene sequences, including 5’UTR, 3’UTR, microRNAs and other noncoding RNA. Sequencing was run with 150 bp paired end reads to achieve coverage of 100X and was performed according to Illumina standard protocol.

### Copy number analysis from exome sequencing data

Control‐FREEC^25^ was used for copy number analysis from BAM files of variant calling analysis. Genes with Segments‐of‐Gain‐Or‐Loss (SGOL) score ≥ 4 were defined as amplified genes and ≤ −2 as deleted genes by cghMCR package of R (http://bioconductor.org/packages/release-/bioc/html/cghMCR.html). The validation of somatic copy number changes was performed as described previously.^33^

### Cell culture and reagents

Human GBC cell lines (OCUG1, SNU308, TGBC2TKB, NOZ, and G415) obtained as a kind gift from Dr. Akhilesh Pandey (IOB, Bangalore) were cultured in DMEM media containing 10% FBS, 100 units/mL penicillin, and 100 mg/mL streptomycin and amphotericin. All cell lines were incubated at 37 °C with 5% CO_2_. The cell lines were authenticated by DNA short tandem repeat (STR) profiling using Promega Geneprint 10 system in conjunction with GeneMarker HID software tool. All cell lines were made mycoplasma free if necessary with EZKill Mycoplasma removal reagent (HiMedia).

### Soft agar assay

All experiments were performed in triplicates as described earlier.^34^ Briefly, anchorage‐independent growth was assessed for the knockdown clones of *ERBB2* and *EGFR* along with respective scrambled control. About 1 mL of 2× DMEM supplemented with 20% FBS containing (1 mL of 1.6% agar) to obtain 0.8% agar was added to the six well plate as bottom agar and was allowed to solidify. Next, 5 * 10^3^ cells were supplemented with 1 mL of 2X DMEM containing 0.8% agar to obtain 0.4% agar and were added to the bottom agar as top agar. The cells were incubated for 2 weeks at 37 °C and 5% CO2. Colonies were counted under a microscope with a magnification of 10X.

### Virus production

293FT cells were seeded in 6‐well plates 1 day before transfection and each of the lentiviral constructs along with packaging plasmids ‐pPAX helper vector and pVSVG were transfected using Lipofectamine 3,000 reagent (Invitrogen) as per manufacturer's protocol. The viral soup was collected 48 and 72 h post transfection, passed through the 0.45 μM filter and stored at 4 °C. Respective cells for transduction were seeded one day before infection in a six‐well plate and allowed to grow to reach 50–60% confluency. One milliliter of the virus soup (1:1 dilution) and 8 μg/mL of polybrene (Sigma) was added to cells and incubated for 6 h. Cells were selected with puromycin (Sigma) (2 μg/mL) selection for 2 days as further described earlier.^33^

### Growth curve

Growth curve assay was performed on a 24 well plate format with a cell density of 20,000 cells/well. Cell growth was assessed post 48 h and 96 h by counting the cells using a hemocytometer and was recorded. Cell proliferation was calculated as percentage proliferation normalized to scrambled control. All the experiments were performed in triplicates.

### MTT assay

Thousand cells per well were seeded in 96 well plate followed by incubation with the drug for 72 h and six replicate per concentration and subsequently incubated with MTT (0.5 mg/mL) for 4 h and then MTT assay was performed and data was acquired at 570 nm using Microplate reader. Percentage cell viability was calculated against vehicle treated.

### Western blotting

Cells were lysed in RIPA buffer and protein concentration was estimated using BCA (MP Biomedical) method. Fifty micrograms protein was separated on 10% SDS‐PAGE gel, the transfer was verified using Ponceau S (Sigma), transferred to nitrocellulose membrane and blocked in Tris‐buffered saline containing 5% BSA (Sigma) and 0.05% Tween‐20(Sigma). The primary antibody against Total HER2 (sc‐33,684 Dilution 1:500), Total EGFR (1005) (sc‐03 Dilution 1:500), Total ERK2(C‐14) (sc‐ 154 Dilution 1:500) and β‐Actin(I‐19)‐R (sc‐1,616‐R Dilution 1:3000) were obtained from Santa Cruz biotechnology. The primary antibodies Phospho‐HER2 (Tyr1248) (AP0152 Dilution 1:500) from Abclonal and Phospho‐p44/42 (T202/Y204) MAPK (#4370) Dilution 1:1000), Phospho‐*EGFR* (Y1068) (#2234 Dilution 1:500) were obtained from Cell signaling technology respectively. Thiazolyl blue tetrazolium bromide (MTT, TC191) was obtained from Hi‐Media.

### Receptor tyrosine kinase proteome array

The relative amount of 49 tyrosine kinases were evaluated using Proteome Profiler Human Phospho‐ RTK array kit (ARY001B – Proteome Profiler, R&D systems) and the protocols were followed as per manufacturer's recommendation. Briefly, cells were harvested, washed with 1X PBS and lysed after which 400 μg of protein was mixed with a buffer and incubated with preblocked nitrocellulose membrane at 4 °C. Subsequently, the membranes were probed using detection antibodies and probed using streptavidin‐HRP, after which signals were developed using the chemi‐reagents provided with the kit. The Pixel density of each spot in the array in duplicate was quantified using Image J macro‐Protein array analyzer plug in. The average pixel density of the duplicate spots for each of the kinases was subtracted from the negative density and was plotted, as detailed earlier.^35^

### Invasion assay

Invasion ability of the cells was assessed in Transwell system using cell culture inserts for 24 well plates with 8 μm pores (BD Biosciences, NJ). The upper side of the cell culture insert was coated with Matrigel (BD Biosciences, San Jose, CA). GBC cells were seeded at a density of 2 × 10^4^ on the upper side of the coated Matrigel in presence of serum free DMEM. Complete DMEM media with 10% FBS was added to the lower side of the insert and were incubated at 37 °C in 5% CO_2_ incubator for 12‐14 h. Post incubation the non‐migratory cells on the lower side of the cell culture insert were removed using a cotton swab. The transwell chambers were fixed and stained with 0.1% crystal violet. The invasion ability was estimated by counting the cells that have migrated to the lower side of the cell culture insert. Cells in visual field with a magnification of 20X were counted in each Transwell chamber in triplicates.

### Wound healing assay

Confluent monolayers in 6‐well plate were subjected to scratch with a sterile pipette tip. After this, cells were washed with 1× PBS to remove debris and subsequently incubated with media. Cell migration at the wound surface was measured during a period of 20 h under an inverted microscope. The quantification of cell migration was done using Cell Profiler^36^ wound healing pipeline for three independent wounds in three independent experiments.

### Immunohistochemistry

Immunohistochemical analysis was done using the standard protocol of Vectastatin Universal kit. Briefly, antigen retrieval was performed by incubating the slides in preheated citrate buffer (pH 6) using a pressure cooker for 10 min. The slides were allowed to cool at room temperature before rinsing with TBST (Tris‐Buffered saline‐ Tween 20 (1%). The endogenous peroxidase activity was blocked by incubating the slides in 3% hydrogen peroxide. The slides were blocked by horse serum for 1 h before incubating with the primary antibody (HER2 DAKO A0485, Phospho‐p44/42 (T202/Y204) MAPK #4370, Total ERK2(C‐14) sc‐154) for overnight at 4 °C in moist chamber. Post incubation the slides were rinsed with TBST and incubated with universal secondary antibody (Vectastatin). The chromogenic reaction was performed using 3′‐3′‐ diaminobenzidine chromogen solution for 5 min which results in brown signal. The slides were rinsed in deionized water and counterstained with hemotoxylin. Finally, the slides were dehydrated and mounted with a mounting medium and cover slip.

### Coimmunopreciptation assay

For immunoprecipitation, cells were harvested in NP‐40 lysis buffer (50 mM Tris pH 7.4, 150 mM NaCl, 0.5% NP‐40, 1 mM EDTA along with protease and phosphatase inhibitors), Protein lysate supernatant were combined with the anti‐EGFR antibody and incubated overnight on a rotator at 4 °C. Protein G‐Sepharose beads (50 μL) were added to the cell lysates the next day and were on a rotator at 4 °C for 4 h. The Protein G‐Sepharose beads were isolated by centrifugation at 2000*g* for 2 min. Further, these beads were washed three times with NP‐40 lysis buffer and heated for 10 min at 100 °C in loading buffer. Samples were run on SDS‐PAGE and then probed by immunoblot for HER2.

### *In vivo* study

Five‐ to six‐week old female NOD‐SCID mice were injected subcutaneously with 3 × 10^6^ cells/mL in 100–200 μL PBS G415 (*N* = 13), NOZ (*N* = 10) and OCUG1(*N* = 10). After injecting the cells, the size of the resulting tumors was determined every third day using calipers. Afatinib inhibitor was administered to the randomized group of mice by oral gavage at 15 mg/kg body weight along with vehicle control (1% Tween 80) for a period of 15 days after the tumor volume has reached between 100 and 150 mm^3^. micro PET‐CT scan was performed at the end of drug treatment. The tumor volume was calculated using the formula – (Width^2^ * Length) /2. After 15 days, the mice were euthanized with CO_2_. Tumors were excised and tissues were stored for molecular and histopathological analysis.

### Statistical analysis

Prism software (GraphPad) was used to analyze proliferation and drug sensitivity of cells to inhibitors, and to determine the statistical significance of differences between the groups by applying an unpaired Student's *t* test. *p* Values <0.05 were considered significant. The Kaplan–Meier estimation of patient survival and correlation analysis were assessed using R packages survival (http://cran.r-project.org/package=survival), and IBM SPSS v20.

### Study approval

The study protocols were approved by ACTREC‐TMC Institutional Review Board, Project #104. The animal study protocols were approved by Institutional Ethics Animal Committee of ACTREC.

### Availability of Supporting Information

The datasets supporting the results of this article are available from the ArrayExpress database (accession number E‐MTAB‐6619).

## Results

### Integrated genomics and proteomics approach identify aberrant alterations in members of the *EGFR* family in gallbladder cancer

**Figure 1:**
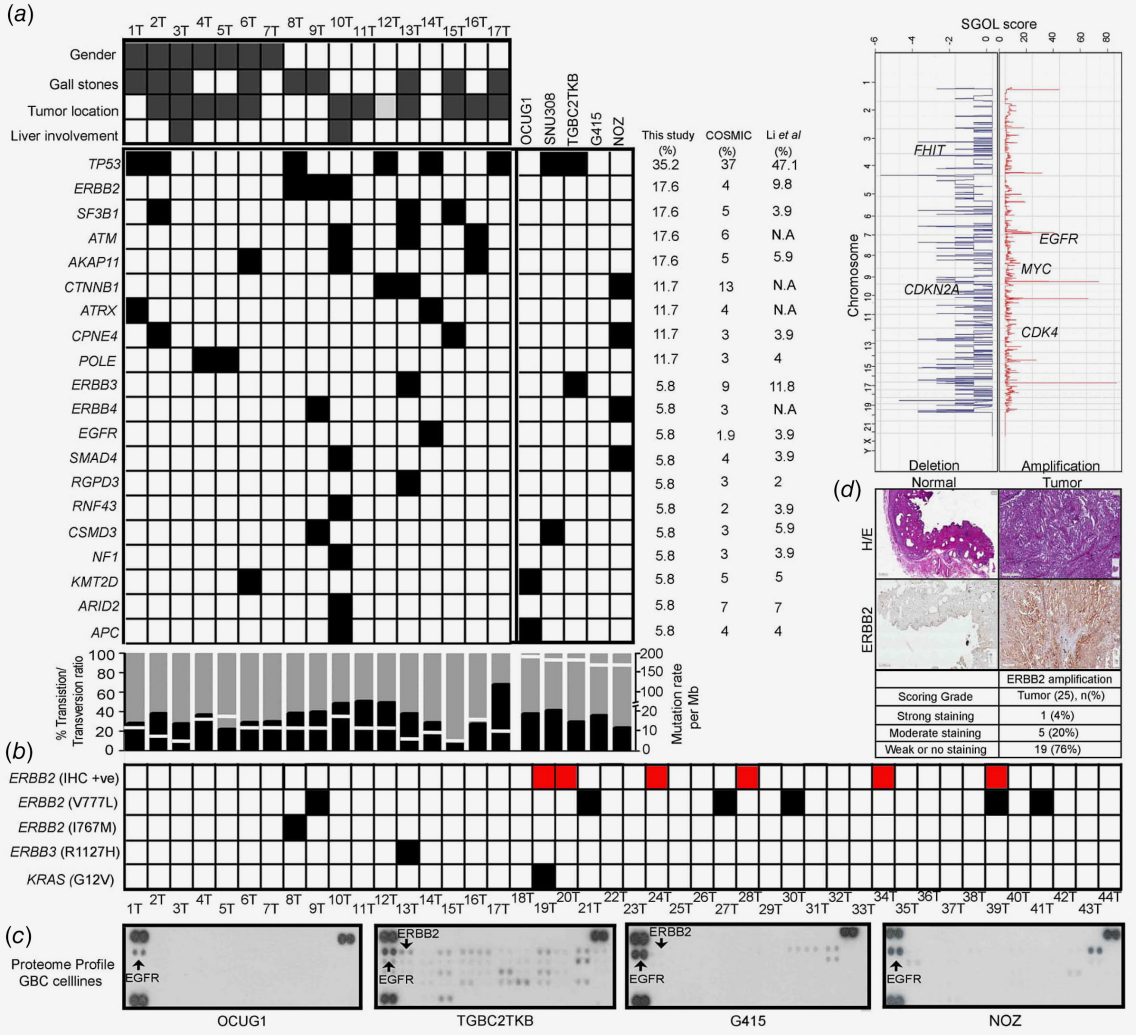
Integrated genomic and proteomic analysis of gallbladder cancer**.** (*a*) The heat map represents somatic mutation landscape in gallbladder cancer patients (*n* = 17) and primary tumor derived cancer cell lines (*n* = 5) using whole exome sequencing. Clinicopathological features such as gender, gallstones, tumor location and liver involvement are shown. The gray solid boxes denote females, presence of gallstone, tumor location (neck) and positive for liver involvement. The white box denotes males, absence of gallstones, tumor location (body) and negative for liver involvement. The genes are arranged in decreasing order of their frequency. Black solid box indicates the presence of mutation in the heatmap. Mutation frequencies of the genes mentioned are shown in our study, COSMIC‐GBC and Li *et al*. study. The transition to transversion ratio is shown in percentage for each patient indicated by different shades (Black denotes transversion and gray denotes transition). Somatic mutation rate/30 Mb is derived from whole exome sequencing data is indicated by white line. Overall copy number changes derived from whole exome sequencing data. The horizontal‐axis is represented by a score of segment gain or segment loss (SGOL score) while the vertical‐axis represents the chromosomal positions. Copy number gain is indicated by red with positive SGOL score while copy number loss is indicated by blue with a negative SGOL score. Representative cancer‐associated genes are annotated in their respective amplified/deleted regions. (*b*) Schematic representation of *ERBB* family mutation validation by Sanger sequencing in an additional set of 27 samples. Solid box indicate presence for mutation in the respective samples, white boxes indicates no event. (*c*) RTK array analysis of gallbladder cancer cells (OCUG1, TGBC2TKB, G415, and NOZ) for 10 min exposure of blot is shown. Each RTK is spotted in duplicate and the pair of dots in each corner of the membrane corresponds to positive and negative control. Tyrosine phosphorylation of EGFR (ERBB1) and ERBB2 were observed consistently, indicated by arrow. D) Immunohistochemistry was performed for ERBB2 expression in tumor samples (*n* = 25). Representative images of IHC stained photomicrographs from tumor and normal samples are shown. Brown color indicates positive expression. The corresponding H/E images are indicated in the upper panel. Below table indicates the quantification of ERBB2 immunostaining data. [Color figure can be viewed at http://wileyonlinelibrary.com]

We performed whole‐exome sequencing on paired tumor and germline DNA samples from 17 patients with gallbladder cancer and 5 gallbladder cancer cell lines (Supporting Information Table S1 and S2). We achieved >100‐fold mean sequence coverage of targeted exonic regions. The average nonsynonymous mutation rate was found to be 7.7 mutations per megabase (Supporting Information Table S3), which is significantly higher than as reported for other populations.^37^ The nucleotide mutation pattern was observed to be enriched for C>T transition followed by A>G transition (Supporting Information Fig. S1), consistent with previous reports.^37^ A total of 5,060 somatic variants found across 17 tumors consisted of 3,239 missense, 1,449 silent, 131 nonsense, 135 indels and 106 splice site mutations. Somatic mutations in genes previously reported to be altered in gallbladder cancer, including recurrent mutations in *TP53* (35.2%)*, ERBB2, SF3B1, ATM* and *AKAP11* at 17.6% each were found to be mutated at comparable frequencies^19^, ^37^ (Fig. 1 *a* and Supporting Information Table S3). For validation of a few *TP53, ERBB2, ERBB3, SMAD4* and *CTNNB1* mutations, sanger‐based sequencing were carried out in a subset of patients (Supporting Information Fig. S2). Among set novel alterations, we observed significant somatic mutations in chromatin modifier genes such as *SF3B1*, *ATRX*, *CREBBP* and *EZH2* that are known to play a significant role in other cancer types.^38^ In addition, we found two tumor samples harbored known activating kinase domain mutations in *ERBB2*, (V777L) and (I767M); while two samples harbored *EGFR* (I1005V) and *ERBB3* (R112H) mutation (Supporting Information Table S4), as reported earlier.^19^, ^39^ Subsequently, based on directed sequencing of *ERBB2* kinase domain, we identified 5 more samples with *ERBB2* mutations harboring (V777L) mutations in an additional set of 27 gallbladder cancer samples (Fig. 1 *b*). Thus, *ERBB2* (V777L) mutations were mutated with an overall frequency of 13% in 6 of 44 gallbladder cancer patients. Additionally, immunohistochemical staining of ERBB2 protein was positive (2 or 3+ intensity) in 24% primary tumors (6 of 25), where adequate tissues were available (Fig. 1 *d*; Supporting Information Figure S5; and, Table S6). In overall, similar to breast cancer, somatic *ERBB2* alterations occur in 40% gallbladder samples (10 of 25) either through mutations or over expression.^39^, ^40^ Interestingly, copy number analysis using cghMCR software identified *EGFR* amplification with a highest Segment Gain Or Loss (SGOL) score of 18 (Fig. 1 *a*), as reported earlier.^18^ Genomic amplifications were also observed at loci harboring *CDK4, MDM4, CCND1, CCNE1, MYC, STK11* and *BRD3,* and deletions in *FHIT*, *SMAD4*, *TRIM33* and *APC*.

Next, to correlate differential activation of signaling molecules with their genomic alterations, we performed phospho‐proteomic profile of four gallbladder cell lines for 49 receptor tyrosine kinases using a phospho‐RTK array. Consistent with whole exome findings, we observed varying levels of EGFR and ERBB2 constitutive phosphorylation in all gallbladder cancer cell lines based on their phospho‐proteome (Fig. 1 *c*) and follow up validation by western blot analysis (Supporting Information Fig. S3A). Interestingly, the whole exome data analysis and Sanger sequencing based validation also revealed that gallbladder patient and a primary tumor derived NOZ cells harbor *KRAS* (G12V) mutation; the G415 gallbladder cells harbor *KRAS* (G13D) mutant allele; while the OCUG1 and SNU308 gallbladder cells were wild type for *KRAS* (Fig. 1 *b*; Supporting Information Figure S2). These four cell lines thus represent diverse gallbladder cancer sub‐classes based on their *KRAS* mutant allele status.^37^ Of note, *KRAS* mutations are known to predict plural clinical outcome in response to EGFR inhibitors in colorectal and lung cancer along with other mutations (Supporting Information Table S4).^41^, ^42^

### *ERBB2* and *EGFR* are essential for gallbladder cancer cells not harboring *KRAS G12 V* mutant allele

**Figure 2:**
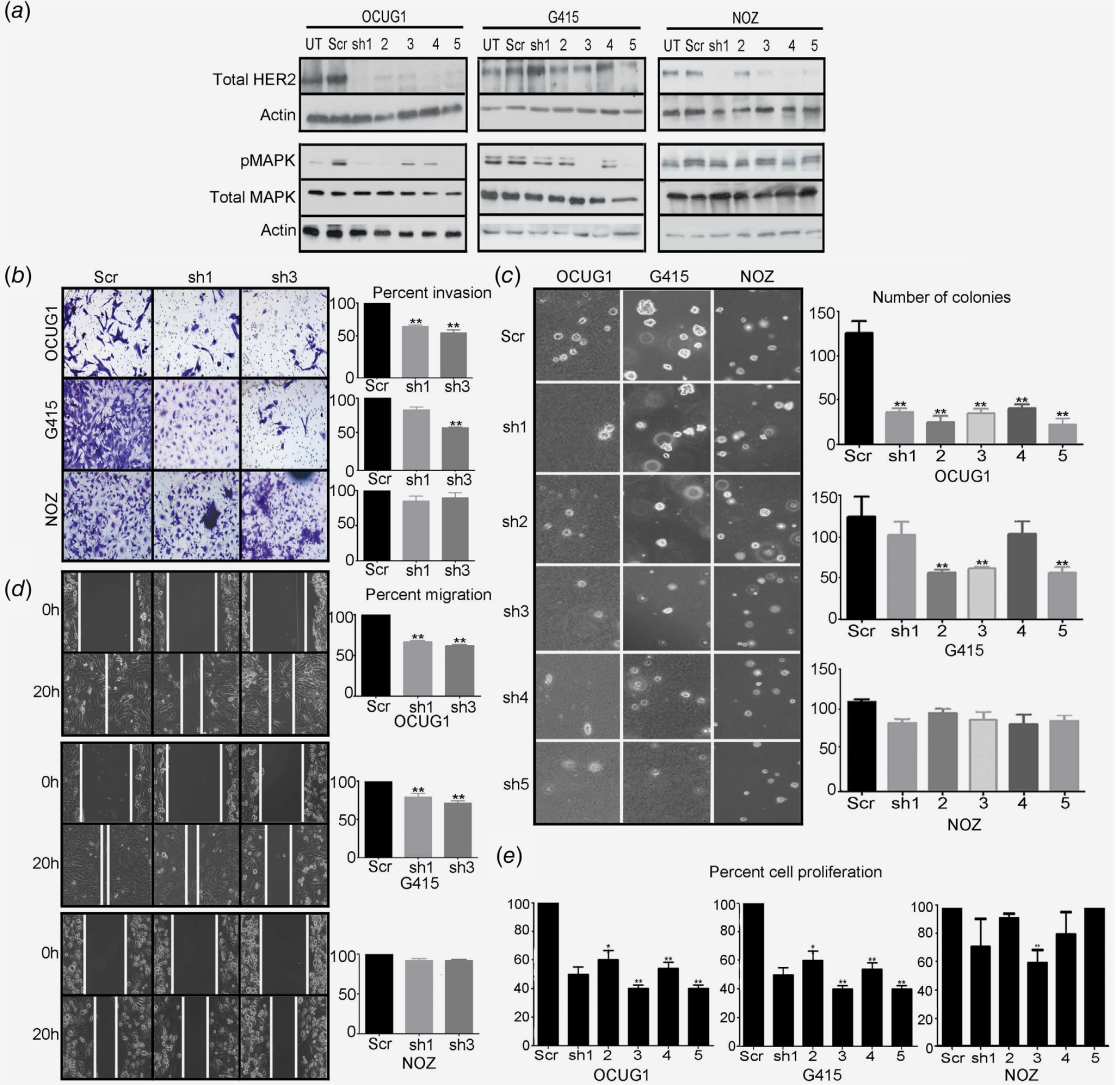
Knockdown of *ERBB2* expression with shRNA inhibits survival of gallbladder cancer cells that do not harbor *KRAS* (G12V) mutant allele. (*a*) Western blot analysis with 5 shRNA constructs used to knock down ERBB2 expression were packaged into lentivirus and used to infect OCUG1, G415, and NOZ cells. Anti‐ERBB2 immunoblot shows that hairpins 3 and 5 efficiently and consistently knock down endogenous *ERBB2* expression across all cells (A upper panel) with concomitant decrease in downstream signaling as assessed by anti‐phospho‐MAPK immunoblot in OCUG1 and G415 cells but not in NOZ cells that harbor a constitutively active *KRAS* (G12V) mutation (A lower panel). Actin is included as a loading control. Scr, scrambled hairpin and untransfected cells (UT) used as a negative control. Knockdown of *ERBB2* expression with shRNA inhibits; invasion characteristics as assessed by matrigel assay (*b*); anchorage‐independent growth as shown by soft agar assay (*c*) and, migration as assessed by scratch assay (*d*) of OCUG1 (with wild type *KRAS*) and G415 (with *KRAS* (G13D)) cells but not NOZ gallbladder cancer cell lines that harbor an activating *KRAS* (G12V) mutation. The graph on the right panel represents percent inhibition normalized to scrambled (Scr) control cells. Similarly, knockdown of *ERBB2* expression with shRNA inhibits percent growth as determined by MTT assay with bar graph plotted with readings obtained on day 4 relative to day 1 for OCUG1, G415, and NOZ cells (*e*) for each shRNA construct and normalized to scrambled control cells. Representative plates from three independent experiments are presented. Colonies were photographed and quantitated after 2 weeks for soft agar assay (Magnification: ×10); 1 day for invasion; and 20 h for migration assay. *p < 0.05. [Color figure can be viewed at http://wileyonlinelibrary.com]

To determine the significance of *EGFR* and *ERBB2* constitutive phosphorylation and *KRAS* mutant alleles in gallbladder cancer cells, we set out to establish whether expression of *ERBB2* is required for gallbladder tumor cell survival. We tested a series of five shRNA constructs in three gallbladder tumor cell lines expressing *ERBB2* with wild type *KRAS* in OCUG1 cells, along with G415 and NOZ cells harboring the *KRAS* (G13D) and *KRAS* (G12V) mutant alleles, respectively. We identified three shRNA constructs that efficiently knocked down expression of *ERBB2* and inhibited the constitutive phosphorylation of MAPK in OCUG1 and G415 cells but not in NOZ cells (Fig. 2 *a*), consistent with drug sensitive outcome described in colorectal cancer wherein cells harboring wild type *KRAS* or mutant *KRAS* (G13D) allele are sensitive to *EGFR* inhibitor but not those harboring mutant *KRAS* (G12V) mutant allele.^43^ This suggests that *KRAS* (G13D) but not *KRAS* (G12V) still requires upstream *EGFR* signaling in gallbladder cancer cells, similar to as established in colorectal cancer.^44^ Next, we used these cells to demonstrate that knockdown of *ERBB2* inhibited anchorage‐independent growth, cell survival, cell invasion and migration efficiently in OCUG1 and G415 cells but not in NOZ cells (Figs. 2 *b*–2e). Furthermore, unlike other *EGFR* family members, *ERBB2* does not require ligand binding for dimerization but can be activated by heterodimerization,^45^ we asked if *EGFR* mediates the activation of downstream signaling pathways. We performed coimmunoprecipitation of *EGFR* and *ERBB2* to establish that *ERBB2* interacts with *EGFR* in gallbladder cells (Supporting Information Fig. S3B), possibly similar to *ERBB3* as shown earlier in gallbladder cells.^37^ Moreover to test if *ERBB2* requires *EGFR* also for sustained signaling and transforming potential, we knocked down the expression of *EGFR* in OCUG1 and G415 cells. The knockdown of *EGFR* inhibited anchorage‐independent growth, cell survival, cell invasion and migration in OCUG1 but not in G415 cells, similar to *ERBB2* knockdown (Supporting Information Fig. S4). Taken together, this suggests that *ERBB2* requires *EGFR* or other members of the family possibly to dimerize for activation, such that down‐regulation of *EGFR* and potentially other members suppress the functionality of *ERBB2,* as has been previously reported in breast cancer.^46^

### Gallbladder cancer cells not harboring *KRAS* (G12V) mutant allele are sensitive to irreversible *EGFR* inhibitors *in vitro* and *in vivo*

**Figure 3:**
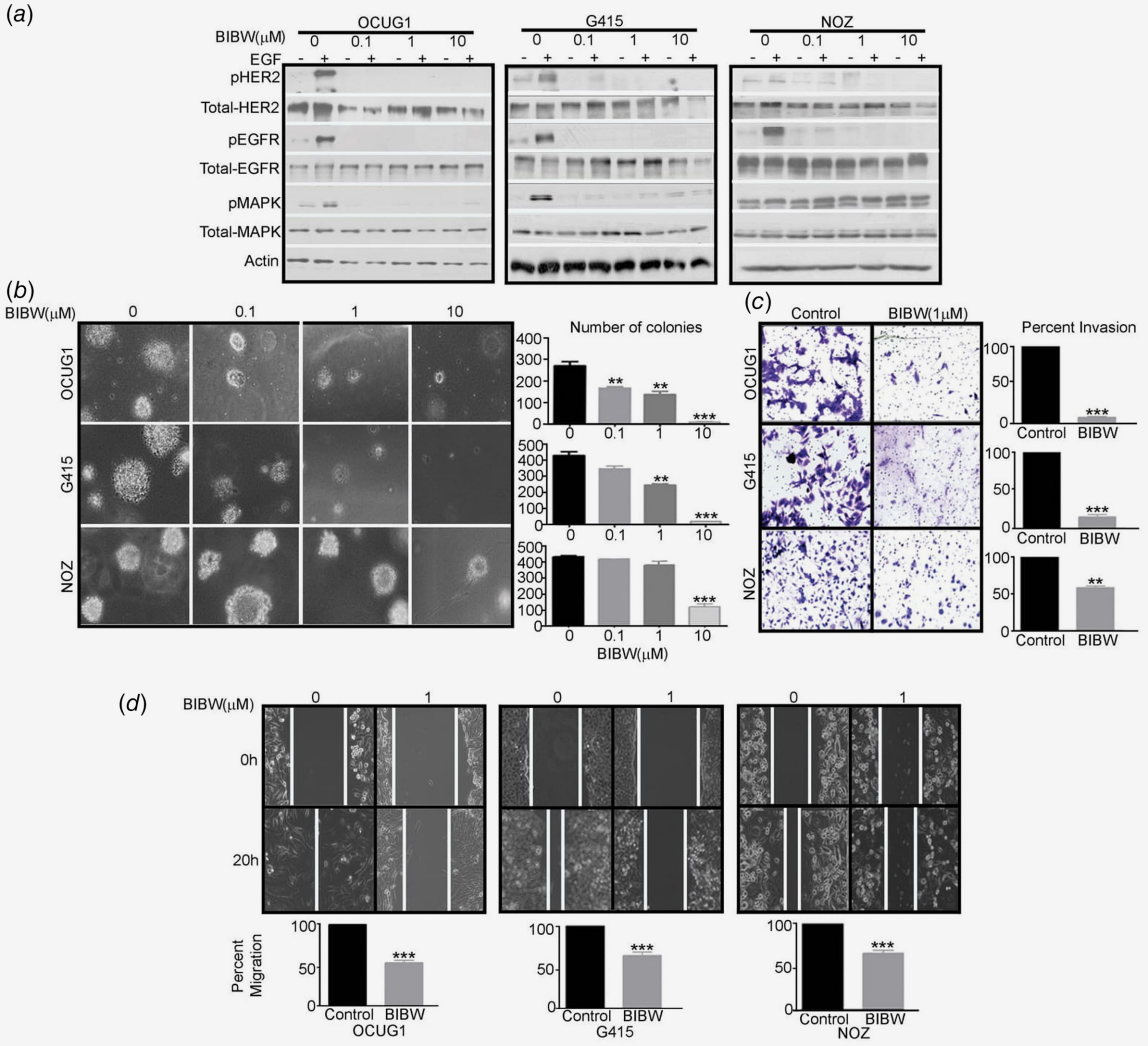
ERBB2 tyrosine kinase activity is essential for gallbladder cancer cells that do not harbor *KRAS* (G12V) mutant allele. (*a*) Treatment of OCUG1, G415 and NOZ gallbladder cancer cells for 10–12 h with 0–10 μM covalent EGFR inhibitor BIBW‐2992 inhibits both basal and ligand‐induced (5‐min stimulation with 20 ng/ml EGF) EGFR and ERBB2 phosphorylation, as evident from immunoblotting with anti‐phospho antibodies specifically recognizing EGFR (pY1068) and ERBB2 (pY1248). However, EGFR inhibitor BIBW‐2992 inhibits MAPK activation as determined by pMAPK p42/p44 (Thr202/Thr204) antibody, a downstream effector component of EGFR‐ and ERBB2‐ dependent signaling pathways in OCUG1 (with wild type *KRAS*) and G415 (with *KRAS* (G13D)) cells but not in NOZ gallbladder cancer cell lines that harbor an activating *KRAS* (G12V) mutation. Actin was used as a loading control. Treatment with the indicated concentrations of EGFR inhibitor BIBW‐2992 inhibited soft agar colony formation (*b*); invasion (*c*); and, migration (*d*) by the OCUG1, G415 but not NOZ gallbladder cancer cell lines with hyper phosphorylated ERBB2. *p < 0.05 *vs*. control. Representative plates from three independent experiments are presented. Colonies were photographed and quantitated after 2 weeks for soft agar assay (Magnification: ×10); 1 day for invasion; and 20 h for migration assay. Quantification of effects of BIBW‐2992 for assays is indicated in the form of bar graph. [Color figure can be viewed at http://wileyonlinelibrary.com]

**Figure 4:**
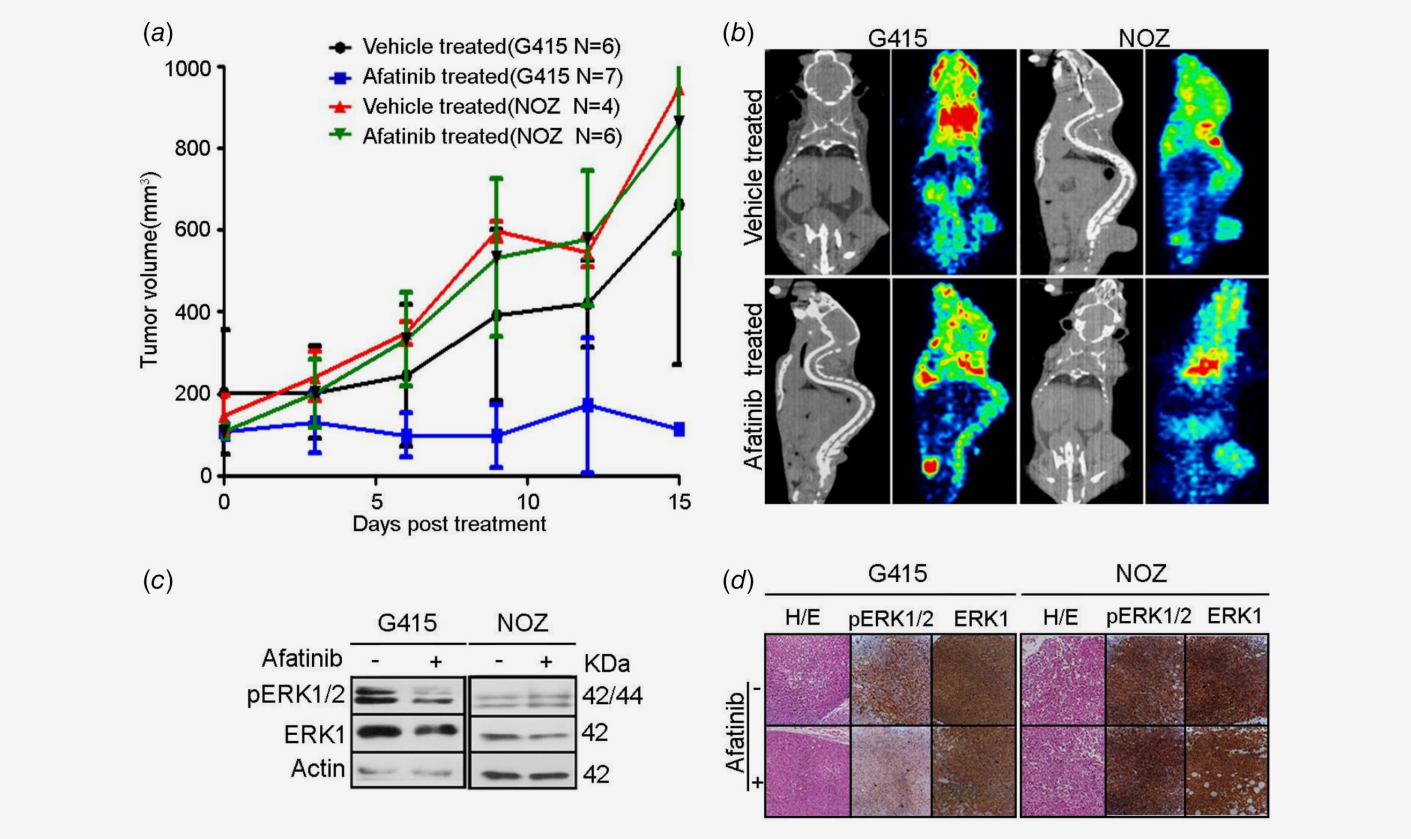
*In vivo* sensitivity of gallbladder cancer cell lines to EGFR inhibitor. (*a*) G415 and NOZ xenografts developed in NOD‐SCID mice were subjected to afatinib (15 mg/kg) or vehicle treatment for a period of 15 days. The plot shows the tumor volume (mm^3^) during the course of drug treatment indicating reduction of tumor volume in afatinib treated G415 xenografts. (*b*) CT scan and PET imaging by F^18^‐FDG uptake is shown for vehicle and afatinib treated xenografts. The gradient color code is shown for uptake of F^18^‐FDG with red indicating maximum uptake (*c*) Immunoblot analysis of phosphorylation of MAPK (pERK1/2, ERK1) is shown for vehicle(−) and afatinib(+) treated xenografts. Actin is used as the loading control. (*d*) Immunohistochemical staining of pERK1/2, ERK1 is shown for vehicle(−) and afatinib(+) treated xenografts. [Color figure can be viewed at http://wileyonlinelibrary.com]

Next, we investigated whether inhibition of kinase activity of EGFR family receptor tyrosine kinases would be effective against gallbladder cancer cell lines. Treatment of the OCUG1 and G415 cells with BIBW‐2992,^47^ but not reversible EGFR inhibitor gefitinib (data not shown), similarly abolished phosphorylation of MAPK in OCUG1 cells, which was constitutively phosphorylated in the untreated gallbladder cell lines compared to the NOZ cells, wherein no significant effect on phospho MAPK levels were observed despite ectopic expression of wild type *ERBB2* or *ERBB3*.^19^ The treatment with BIBW‐2992 also resulted in a marked decrease in migration, invasion, and colony formation ability of OCUG1 and G415 cells, whereas no effect was observed on NOZ cells harboring *KRAS* (G12V) mutant allele (Figs. 3 *a–* 3 *d*). Furthermore, when injected subcutaneously into NOD/SCID mice, 13 of 13 mice injected with G415 cells formed tumors ~13 days post injection; 10 of 10 mice injected with NOZ cells ~6 days of post injection; while none of 10 mice injected with OCUG1 cells formed tumors up to 2 months post injection of cells (Supporting Information Table S9). When the tumors reached ~100‐150 mm^3^, tumors were treated orally with 15 mg/Kg irreversible EGFR inhibitor Afatinib‐ or vehicle for a period of 15 days. Consistent with *in vitro* data, tumors treated with Afatinib slowed or reversed their growth compared to vehicle in G415 xenografts (*n* = 7) but not in NOZ (*n* = 6) xenografts. The overall effect on tumor burden in vehicle‐treated *versus* Afatinib‐treated mice were 5.7‐folds lower in G415 xenografts, while no significant differences were observed in in NOZ xenografts (Figs. 4 *a* and 4 *b*). This reduction in tumor size in G415 xenografts was paralleled by the reduction in the amounts of phospho‐ERK1/2 by immunohistochemical analyses (Figs. 4 *c–* 4 *d*, lower panel) of explanted tumors, further validating our *in vitro* findings (Fig. 3 *a*) and implicating *ERBB2* as an important therapeutic target under neo‐adjuvant or adjuvant settings in treating gallbladder cancer patients.

## Discussion

Our study represents the first genomic landscape of an early‐stage gallbladder cancer among an ethnically distinct population that reveals somatic mutations in *TP53, ERBB2, ATM, AKAP11, SMAD4* and *CTNNB1* similar to as reported in advance‐stage gallbladder tumors.^15^, ^19^, ^20^, ^21^ Our mutation pattern analysis revealed an enrichment for C>T transition followed by A>G transition, a signature which suggests an underlying chronic inflammation leading to GC to AT polyclonal transition,^48^ as reported earlier.^49^ We also observed significant somatic mutations in chromatin modifier genes such as *SF3B1*, *ATRX*, *CREBBP* and *EZH2* that have not been reported earlier in gallbladder cancer, indicating potential therapeutic options. Analyzing the potential effects of somatic alterations on survival of gallbladder cancer patients, we observed a trend among patients with wild type *TP53* to survive longer than patients with *TP53* mutations, which is known to predict failure of chemotherapy in several cancer types^50^ and is consistent with previous reports observed in gallbladder cancer.^51^

Additionally, consistent with a recent report that described alterations in *ERBB2* and *ERBB3* at a frequency of 9.8% and 11.8% respectively among Chinese gallbladder cancer,^19^, ^37^ we found recurrent activating *ERBB2* (V777L) mutation in 6 of 44 gallbladder cancer samples with an overall mutation frequency of 13%, in addition to IHC based over expression across 24% (6 of 25) primary tumors. Taken together, *ERBB2* is altered in 40% gallbladder samples (*n* = 25) either through mutation or over expression. *ERBB2* (V777L) mutation and ERBB2 overexpression has been shown to be sensitive to lapatinib in biliary tract cancer, breast cancer cell lines and other isogenic systems overexpressing the alteration.^18^, ^39^ Functional studies performed using gallbladder cell lines establish that *ERBB2* and *EGFR* are essential for the survival of gallbladder cancer cells. Given that *ERBB2* lacks the ligand binding domain, the coimmunoprecipitation experiments suggest that ERBB2 dimerize with EGFR, and possibly with other members, to constitutively activate the pathway. Interestingly, genetic or pharmacological ablation of *ERBB2* and *EGFR* function, using EGFR small‐molecule irreversible inhibitor BIBW‐2992, diminishes the survival, anchorage‐independent growth, migration and invasion characteristics of gallbladder cancer cell lines, suggesting members of the EGFR family as an effective therapeutic target. Furthermore, while *KRAS* mutations in gallbladder cancer have been reported to occur at a frequency from 3% to 30%,^52^ some co‐occurring with activating *ERBB3* mutation, we observed *KRAS* (G12V) and (G13D) mutation in primary gallbladder tumors and gallbladder cancer cell lines that are known to be associated with differential clinical outcome in response to anti‐*EGFR* therapy in colorectal cancer.^53^, ^54^ The biological characteristics of *KRAS* mutation is known to vary by cancer types as those found in pancreatic and nonsmall cell lung cancers are predominantly at codon 12, while in colorectal and gallbladder mutations appears to be in codon 12 and codon 13.^55^ Moreover, clinical response among patients along with *in vitro* and *in vivo* studies with isogenic colon cell line indicate *KRAS* (G13D) mutation as sensitive but (G12V) as resistant to anti‐*EGFR* therapy suggesting codon 13 mutations are still dependent on inductive upstream *EGFR* signaling and exhibit weaker *in vitro* transforming activity than codon 12 mutations.^53^

A recent study by Li *et al*. suggested that NOZ gallbladder cancer cells are responsive to ERBB2 inhibitors based on ectopic expression of mutant *ERBB2* constructs.^19^ However, we believe that the evidence presented in our study argues for ERBB2 inhibitors as unlikely to be relevant among gallbladder cancer, such as NOZ tumor cells, that harbor *KRAS G12V* mutation. The wild‐type NOZ cell line, in absence of ectopic expression of mutant *ERBB2,* show endogenous constitutive MAPK phosphorylation, both by the Li *et al*. study^19^ and as presented here (Figs. 2 *a* and 3 *a*); does not show any significant inhibition of MAPK phosphorylation when treated with EGFR inhibitors,^19^ as also presented here (Fig. 3 *a*); and, does not show significant inhibition of cell survival when treated with EGFR inhibitors,^19^ and as presented here (Figs. 3 *b*–3 *d*). Finally, even the knockdown of *ERBB2* had no impact on MAPK phosphorylation, or on the survival of NOZ cells, suggesting that the wild‐type NOZ cells are refractory to ERBB2 inhibitor due to the *KRAS G12V* mutation (Figs. 2a–2 *e*). An oversight to consider a co‐occurring *KRAS* activating mutation portray an important corollary to a clinical situation that could potentially lead to an inaccurate prognosis if the decision is restricted exclusively based on activating *ERBB2* alteration.

In summary, besides suggesting adoption of anti‐EGFR therapy as a therapeutic option in early‐stage gallbladder cancer based on *ERBB2* alteration, we present the first evidence that presence of *KRAS* (G12V) but not *KRAS* (G13D) mutation may preclude such patients to respond to the treatment, similar to the clinical algorithm commonly practiced based on *EGFR* alteration in colorectal cancer. As low prevalence rate of the disease, target accrual in clinical trials has been a bottleneck in gallbladder cancer, our study forms the basis to include gallbladder patients for an anti‐EGFR therapy under basket clinical trials such as the NCI–Molecular Analysis for Therapy Choice (NCI‐MATCH) trials that are genomically matched.^56^

## Authors contributions

P.I. and AD designed research. P.I., M.R., P.C., N.G., B.D., S.S., R.P., R.T., B. M., and B.S. performed research. S.G.B., V.C., A.C., M.R.R., P.G., K.P., S.D. and S.V.S. contributed reagents and samples. P.I., M.R., P.C. N.G., A.J., H.K., P. Chau., A.I., and AD analyzed data. P.I. and AD wrote the paper.

## Supporting information


**Supplementary figure S1. Characteristic features of variants identified from whole exome sequencing data**.
The distribution of different types of somatic mutations in gallbladder tumors (**A**) and cancer cell lines (**C**) is represented. Each bar represents percentage of each type of mutation identified by Mutect and GATK from whole exome sequencing data. Bar graph representation of specified transitions and transversions resulting in non‐synonymous somatic mutations identified by Mutect and GATK for primary tumors (**B**) and cell line samples (**D**).
**Supplementary Figure S2. Sanger validation of mutations identified by exome sequencing.**
**A)** Heat map representation of mutations identified by whole exome sequencing in the discovery set and its validation by directed sequencing in an additional set of 27 primary tumors and 5 gallbladder cell lines. Solid box indicates the samples in which the corresponding mutations are validated by Sanger sequencing and white indicates no event. Gray solid box indicates samples not attempted for Sanger validation.
**B)** Sequencing chromatograms for somatic *ERBB2* and *KRAS* mutations found in the whole experiment. The reverse complement of reverse sequencing reads is displayed by Mutation Surveyor. Two different alleles, marked by overlapping peaks are present in the tumor (T) sample but the normal (N) sample marked with an arrow.
**Supplementary Figure S3: Constitutive phosphorylation and heterodimerization of ERBB2 and EGFR in gallbladder cancer cells.**
**A)** Immunoblot analysis of OCUG1, G415 and NOZ gallbladder cancer cells for phosphorylation of HER2 and EGFR is shown. Actin is used as a loading control.
**B**) Equal amount of whole cell lysates(400 μg) were subjected to immunoprecipitation using anti‐EGFR antibody and rabbit isotype antibody IgG. Further, immunoblotting was performed with anti‐HER2 antibody to detect heterodimerization of EGFR‐ERBB2. 10% of whole cell lysate was loaded as a input control.
**Supplementary Figure S4: Knockdown of *EGFR* expression with shRNA inhibits survival of gallbladder cancer cells that do not harbor *KRAS* mutant allele.**
Western blot analysis with a shRNA constructs to knock down *EGFR* expression in OCUG1 and G415 cells. Anti‐EGFR immunoblot shows that hairpins efficiently consistently knock down endogenous EGFR expression with concomitant decrease in MAPK phosphorylation in OCUG1 cells but not in G415 cells that harbor a constitutively active KRAS (G13D) mutation. Actin is included as a loading control. Scr, scrambled hairpin used as a negative control. Knockdown of *EGFR* expression with shRNA inhibits anchorage‐independent growth as shown by soft agar assay (**B**); and, invasion characteristics as assessed by matrigel assay (**C**).
**Supplementary Figure S5: ERBB2 overexpression in gallbladder tumor samples**
Representative images of IHC stained photomicrographs from 4 tumors and 2 normal samples are shown. Brown color indicates positive expression.
**Table S1:** Demographics of the gallbladder primary tumor samples
**Table S2:** Exome sequencing quality control and statistics of primary tumor samples and celllines.
**Table S3:** Statistics of alterations sample‐wise in exome sequencing of primary tumor samples and cell lines
**Table S4:** Total list of alterations in the exome of primary tumor samples
**Table S5:** Clinical characteristics of gallbladder primary tumor cohort
**Table S6:** IHC scores for ERBB2 amplification in gallbladder samples (n = 25)
**Table S7:** Primers for validation of alterations
**Table S8:** STR Profiling of gallbladder cancer cell lines
**Table S9:** Tumor volume of mice during the course of treatment
