# Supplementary material for: ERBB2 and KRAS alterations mediate response to EGFR inhibitors in early stage gallbladder cancer
Source: Int J Cancer. 2018 Dec 8;144(8):2008–19. doi: 10.1002/ijc.31916 (PMC6378102; doi:10.1002/ijc.31916)
Supplement: Supplementary file 1 — Supplementary figure S1. Characteristic features of variants identified from whole exome sequencing data. The distribution of different types of somatic mutations in gallbladder tumors (A) and cancer cell lines (C) is represented. Each bar represents percentage of each type of mutation identified by Mutect and GATK from whole exome sequencing data. Bar graph representation of specified transitions and transversions resulting in non‐synonymous somatic mutations identified by Mutect and GATK for primary tumors (B) and cell line samples (D). Supplementary Figure S2. Sanger validation of mutations identified by exome sequencing. A) Heat map representation of mutations identified by whole exome sequencing in the discovery set and its validation by directed sequencing in an additional set of 27 primary tumors and 5 gallbladder cell lines. Solid box indicates the samples in which the corresponding mutations are validated by Sanger sequencing and white indicates no event. Gray solid box indicates samples not attempted for Sanger validation. B) Sequencing chromatograms for somatic ERBB2 and KRAS mutations found in the whole experiment. The reverse complement of reverse sequencing reads is displayed by Mutation Surveyor. Two different alleles, marked by overlapping peaks are present in the tumor (T) sample but the normal (N) sample marked with an arrow. Supplementary Figure S3: Constitutive phosphorylation and heterodimerization of ERBB2 and EGFR in gallbladder cancer cells. A) Immunoblot analysis of OCUG1, G415 and NOZ gallbladder cancer cells for phosphorylation of HER2 and EGFR is shown. Actin is used as a loading control. B) Equal amount of whole cell lysates(400 μg) were subjected to immunoprecipitation using anti‐EGFR antibody and rabbit isotype antibody IgG. Further, immunoblotting was performed with anti‐HER2 antibody to detect heterodimerization of EGFR‐ERBB2. 10% of whole cell lysate was loaded as a input control. Supplementary Figure S4: Knockdown of EGFR expressio [file IJC-144-2008-s001.pdf]

Supplementary Figure S1. Characteristic features of variants identified from whole exome sequencing data

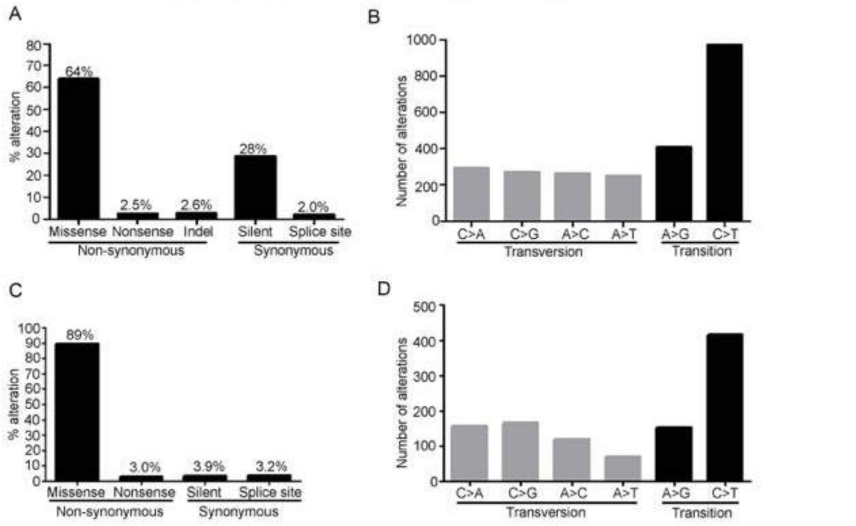

Supplementary Figure S2. Sanger validation of mutations identified by exome sequencing

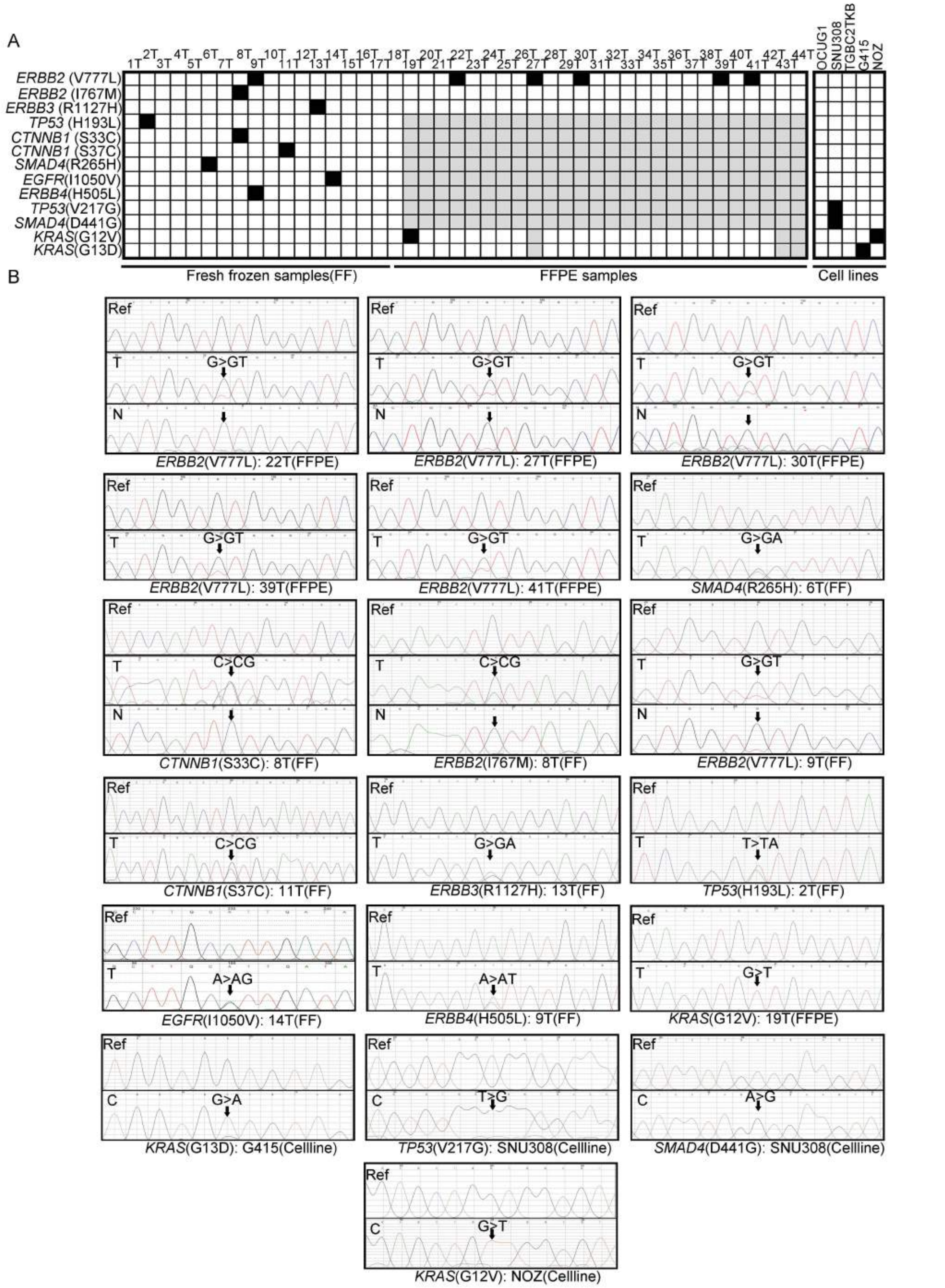

Supplementary Figure S3. Constitutive phosphorylation and heterodimerization of ERBB2 and EGFR in gallbladder cancer cells

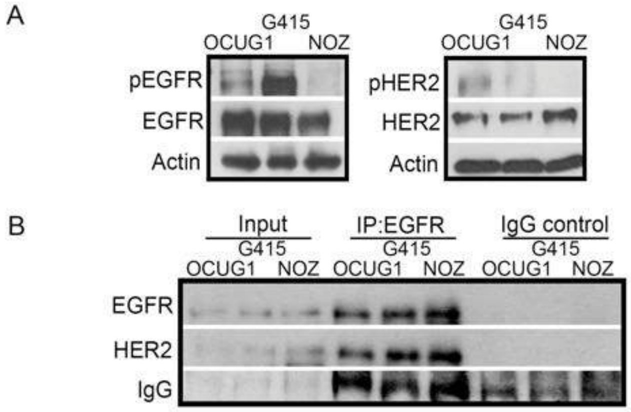

Supplementary Figure S4. Knockdown of *EGFR* expression with shRNA inhibits survival of gallbladder cancer cells that do not harbor *KRAS* mutant allele

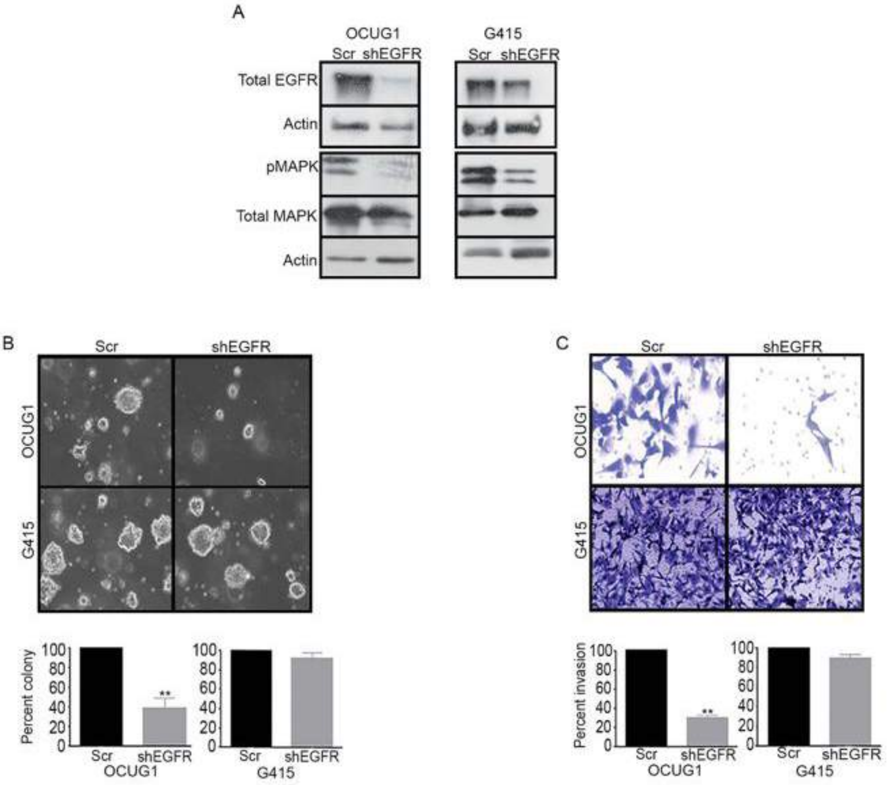

Supplementary Figure S5.: ERBB2 overexpression in gallbladder tumor samples

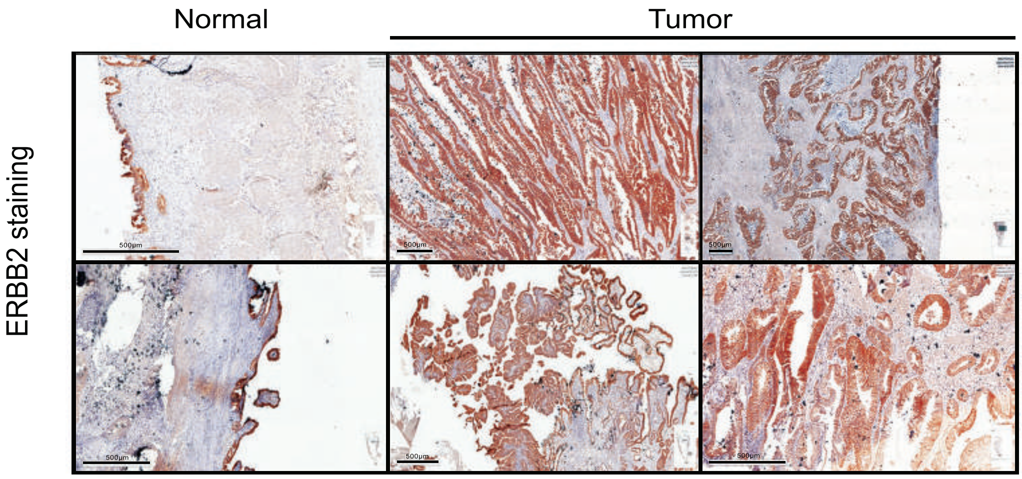

Table S1: Demographics of the gallbladder primary tumor samples

Clinical Characteristics	Variables	Frequency (N=44)
**Ethnicity**	Asian (Indian)	44
**Age**	Median range	53(31-75)
**Sex**	Male	17
	Female	27
**Tumor Stage**	Early (T1 and T2)	29
	Late (T3 and T4)	14
	Could not be accessed	1
**Nodal Stage**	Node positive	16(36%)
	Node negative	28(63%)
**Histological type**	Adenocarcinoma	33
	Adenosquamous	4
	Squamous	3
	No information	4
**Degree of Differentiation**	Poor	10
	Moderate	28
	Well	2
	No information	4
**Lymphovascular emboli**	Yes	3
	No	40
	No info	1
**Perineural invasion**	Yes	4
	No	39
	No info	1
**Gallstones**	Yes	11
	No	32
	No info	1

Table S2: Exome sequencing quality control and statistics of primary tumor samples and celllines.

Sample No	Sample ID	Sample type	Total Reads	Mapped Reads	Mapped reads in pair	Duplication Rate(%)	Mapping %	Coverage
1N	AD0752	Normal	63858844	35885515	34086308	11.5	56.2	168
2N	AD0759	Normal	43455624	27369663	26143858	17.6	63.0	129
3N	AD0754	Normal	54998184	37809365	36492238	22.7	68.7	177
4N	AD0755	Normal	66115458	47741170	46444766	21.3	72.0	224
5N	AD0756	Normal	39594984	23065796	21858806	16.7	58.3	108
6N	AD0757	Normal	51254660	34364520	32857726	18.0	67.0	161
7N	AD0437	Normal	44676668	29362140	28289852	25.5	65.7	139
8N	AD0439	Normal	49994928	29952529	28671136	28.1	59.0	142
9N	AD0746	Normal	37563776	21449942	20284354	17.3	57.1	100
18N	AD0435	Normal	43581276	28263519	27285190	23.1	64.9	134
1T	AD0739	Tumor	37795668	25657903	24820778	24.9	67.9	122
2T	AD0758	Tumor	59032310	36867711	35288198	19.4	62.5	173
3T	AD0741	Tumor	46034048	28780365	27600442	24.0	62.5	136
4T	AD0742	Tumor	27047422	17293941	16669644	30.0	63.9	82
5T	AD0743	Tumor	27047423	17293841	16669744	20.0	63.0	114
6T	AD0744	Tumor	46434392	27105282	26073070	12.2	58.4	129
7T	AD0438	Tumor	48018464	30157789	28970210	25.6	62.8	143
8T	AD0440	Tumor	39564826	24359457	23292676	25.2	61.6	115
9T	AD0748	Tumor	54159317	32433869	31016282	10.0	59.9	152
10T	AD0745	Tumor	63778254	41274673	39646496	28.7	64.7	195
13T	AD0750	Tumor	48550174	28391632	27054752	10.0	58.5	133
15T	AD0747	Tumor	74760204	48372303	46588150	14.9	64.0	227
11T	AD0740	Tumor	37795669	25657907	24820778	24.9	67.9	128
12T	AD0736	Tumor	54826754	35184594	35057946	12.1	62.1	167
14T	AD0738	Tumor	36430489	25438691	25703748	25.2	71.0	127
16T	AD0477	Tumor	46034058	28780375	27600442	24.0	63.5	136
18T	AD0761	Tumor	43581277	28263519	27285190	23.1	64.9	134
OCUG1	AD1019	Cellline	68889377	68328077	68185712	26.9	65.9	220
G415	AD1020	Cellline	73569111	73125971	73032062	26.69	66.9	235
NOZ	AD1021	Cellline	64521902	63920376	63796570	26.37	67.9	205
SNU308	AD1022	Cellline	62013432	61500302	61396508	26.05	68.9	198
TGBC2TKB	AD1023	Cellline	63793578	62868021	62689988	26.5	69.9	204

Table S3: Statistics of alterations sample-wise in exome sequencing of primary tumor samples and cell lines

		Unpaired tumor samples							Paired tumor samples									
		5T	13T	6T	14T	7T	15T	16T	8T	1T	9T	2T	3T	10T	11T	12T	4T	17T
**Overall distribution**	Total number of variants	10242	6601	18193	14198	8079	21303	31940	10253	19491	6048	2566	9995	14709	33874	29240	54578	62559
	3UTR entries	360	213	312	229	307	122	423	381	524	216	142	384	265	1254	988	2086	10089
	5Flank entries	0	0	0	0	0	0	1	0	1	0	0	1	1	2	1	1	2
	5UTR entries	156	89	170	113	138	82	216	264	242	101	69	223	199	552	484	888	1863
	IGR entries	1362	758	6415	5343	1002	10698	14151	1854	6476	1180	472	1570	6002	4728	4396	7594	10821
	Intron entries	5630	3263	8128	5845	4248	7957	12436	5357	9270	3006	968	5563	5560	18204	14612	31722	28375
	De_novo_Start_InF000rame entries	0	1	2	0	3	0	2	0	4	2	2	1	0	7	7	5	15
	De_novo_Start_OutOfFrame entries	7	5	6	3	6	1	6	4	3	2	1	2	3	15	16	20	33
	Frame_Shift_Del entries	18	23	18	6	17	6	13	32	19	5	7	16	12	42	46	69	68
	Frame_Shift_Ins entries	10	3	3	7	7	0	9	27	15	10	6	19	11	27	36	47	60
	In_Frame_Del entries	3	3	2	0	1	0	3	1	3	2	1	1	3	2	2	8	10
	In_Frame_Ins entries	0	2	2	0	1	0	1	2	2	0	0	0	0	1	2	4	6
	lincRNA entries	155	57	677	503	96	1167	1512	156	435	126	56	173	552	468	408	739	968
	RNA entries	454	279	681	557	303	894	1281	462	810	272	128	448	653	1243	1096	2040	1922
	Start_Codon_Del entries	0	0	0	0	0	0	0	0	0	0	0	1	0	1	0	1	0
	Start_Codon_Ins entries	0	0	0	1	0	0	0	1	0	0	0	1	0	0	0	2	1
	Start_Codon_SNP entries	2	3	0	0	1	1	1	1	0	0	0	0	0	9	8	5	9
	Stop_Codon_Del entries	0	0	0	0	0	0	0	0	0	0	1	0	0	1	1	1	2
	Stop_Codon_Ins entries	0	0	0	0	0	0	0	1	0	0	0	1	0	0	1	1	1
	Missense mutation entries	1156	1019	970	894	1057	236	1107	916	903	636	388	780	873	3579	3448	4652	4157
	Nonsense mutation entries	16	22	15	9	23	7	27	18	24	19	13	13	62	52	48	51	40
	Nonstop entries	2	2	2	0	0	1	1	3	2	0	0	0	2	7	9	6	6
	Silent entries	875	825	747	668	821	114	710	729	723	446	295	764	481	3561	3519	4467	3955
	Splice entries	36	34	43	20	48	17	40	44	35	25	17	34	30	119	112	169	156
**NOVEL, dbSNP, COSMIC and TMC-SNPdb distribution**	Total number of variants	10242	6601	18193	14198	8079	21303	31940	10253	19491	6048	2566	9995	14709	33874	29240	54578	62559
	Novel entries	1624	962	1242	982	1085	1002	1755	633	825	541	262	228	1456	1061	805	1449	1770
	Exclusive Cosmic entries	25	23	20	25	15	8	31	17	21	15	14	7	102	26	19	28	20
	Exclusive DBsnp entries	2537	1766	9731	8353	1883	15707	20712	490	4205	244	75	137	4830	1616	1244	2352	7633
	Exclusive TMC-SNPdb entries	281	123	210	122	225	210	318	618	878	611	307	571	468	776	559	1616	955
	Cosmic+DBsnp common entries	89	124	60	80	75	18	67	18	14	12	19	6	31	69	63	86	103
	Cosmic+TMC-SNPdb common entries	8	3	5	2	5	3	19	39	33	48	12	25	20	46	19	73	47
	DBsnp+TMC-SNPdb common entries	5262	3177	6537	4300	4345	4309	8687	7536	12726	4180	1538	8081	7345	26353	22533	43886	47405
	Cosmic+dbSNP+TMC-SNPdb common entr	416	423	388	334	446	46	351	902	789	397	339	940	457	3927	3998	5088	4626
	Total Cosmic entries	538	573	473	441	541	75	468	976	857	472	384	978	610	4068	4099	5275	4796
**COSMIC and Novel distribution**	Total number of variants	1746	1112	1327	1089	1180	1031	1872	707	893	616	307	266	1609	1202	906	1636	1940
	3UTR entries	55	32	40	33	37	22	58	18	29	13	7	4	52	39	24	41	373
	5Flank entries	0	0	0	0	0	0	0	0	0	0	0	0	0	0	0	0	0
	5UTR entries	22	9	15	19	15	13	24	5	12	10	5	5	21	10	15	19	89
	IGR entries	170	112	259	213	132	277	431	93	131	56	25	29	212	126	91	144	207
	Intron entries	876	471	554	396	574	427	728	264	390	233	92	100	605	536	370	770	734
	De_novo_Start_InF000rame entries	0	1	0	0	1	0	0	0	0	0	0	0	0	0	0	0	0
	De_novo_Start_OutOfFrame entries	0	1	1	2	1	1	0	1	0	0	0	0	3	2	1	0	1
	Frame_Shift_Del entries	5	10	6	2	3	5	5	4	3	0	2	2	2	8	5	7	8
	Frame_Shift_Ins entries	4	1	1	1	3	0	4	2	0	2	0	1	3	3	2	5	12
	In_Frame_Del entries	2	1	1	0	0	0	3	1	1	1	0	0	1	0	0	2	3
	In_Frame_Ins entries	0	2	0	0	0	0	0	0	0	0	0	0	0	0	1	0	0
	lincRNA entries	24	7	19	11	11	18	32	10	8	9	4	8	23	9	7	23	27
	RNA entries	55	33	60	31	38	44	55	24	39	15	10	9	52	44	30	49	44
	Start_Codon_Del entries	0	0	0	0	0	0	0	0	0	0	0	0	0	0	0	0	0
	Start_Codon_Ins entries	0	0	0	1	0	0	0	1	0	0	0	1	0	0	0	0	0
	Start_Codon_SNP entries	0	1	0	0	1	0	0	0	0	0	0	0	0	2	2	0	1
	Stop_Codon_Del entries	0	0	0	0	0	0	0	0	0	0	0	0	0	0	0	0	0
	Stop_Codon_Ins entries	0	0	0	0	0	0	0	0	0	0	0	0	0	0	0	0	0
	Missense mutation entries	352	277	247	247	214	155	364	185	195	171	101	63	456	267	229	376	275
	Nonsense mutation entries	5	9	2	5	11	6	16	1	8	8	3	2	49	6	7	5	1
	Nonstop entries	2	0	0	0	0	1	0	0	1	0	0	0	2	0	0	1	1
	Silent entries	163	139	113	123	130	57	144	88	74	94	52	42	116	127	111	185	154
	Splice entries	11	6	9	5	9	5	8	10	2	4	6	0	12	23	11	9	10

Table S4: Total list of alterations in the exome of primary tumor samples

Gene	Mutation	Reccurrence	Functional Prediction tools	5T	13T	6T	14T	7T	15T	16T	8T	1T	9T	2T	3T	10T	11T	12T	4T	17T	Cosmic	dbSNP	TMC-SNPdb	Variant_Classification
**A1CF**	p.L552L	1	0		■																COSM3675808,COSM3675810,C	rs34190540		Silent
**AAR2**	p.A223A	1	0				■														COSM1411487	rs716431		Silent
**ABCA13**	p.S780N	1	2			■															COSM4162269,COSM4162268	rs116299940		Missense_Mutation
**ABCA13**	p.I1434V	1	1			■															COSM4162270,COSM4162271	rs17132195		Missense_Mutation
**ABCA13**	p.K2436R	1	1			■															COSM4162279,COSM4162278	rs17132206		Missense_Mutation
**ABCA13**	p.T162T	1	0			■															COSM4162265,COSM4162264	rs17132152		Silent
**ABCA13**	p.Q483Q	1	0			■															COSM4162267,COSM4162266	rs113452211		Silent
**ABCA13**	p.S2154L	1	0			■															COSM4162273,COSM4162272	rs17092911		Missense_Mutation
**ABCA13**	p.T2264T	1	0			■															COSM4162276,COSM4162277	rs17132201		Silent
**ABCA6**	p.E1463Q	1	1		■																COSM1303261	rs143326198		Missense_Mutation
**ABCA9**	p.R262Q	1	1		■																COSM3691750	rs61744800		Missense_Mutation
**ABCB1**	p.N21D	1	2		■																COSM1178512	rs9282564		Missense_Mutation
**AC092850.1**	p.H204H	1	0			■															COSM4146830	rs73151964		Silent
**ACACB**	p.F2363F	1	0									■									COSM934768	rs202202415		Silent
**ACAN**	p.T985S	2	2								■		■								COSM3678472,COSM3678471		1	Missense_Mutation
**ACOT7**	p.T341T	1	0					■													COSM328033	rs61739537		Silent
**ACSS2**	p.R458C	1	7		■																COSM4097832,COSM4097831	rs373930264		Missense_Mutation
**ADAM21**	p.F448Y	1	2					■													COSM1181797,COSM1181796	rs201068485		Missense_Mutation
**ADAM28**	p.A534V	1	1														■				COSM3720712	rs6982284		Missense_Mutation
**ADAM30**	p.E754K	2	0										■				■				COSM349740	rs61752482		Missense_Mutation
**ADAM7**	p.P192P	1	0					■													COSM3763272	rs61752047		Silent
**ADAMTS12**	p.Y425Y	1	0				■														COSM1067367	rs530632109		Silent
**ADAMTSL4**	p.P654P	1	0					■													COSM2182336,COSM4022018,C	rs370147831		Silent
**ADNP2**	p.A715A	1	0				■														COSM1563991	rs117499189		Silent
**AGAP8**	p.T520M	1	3				■														COSM3998004	rs201435946		Missense_Mutation
**AGAP8**	p.E545D	3	1	■			■												■		COSM2145901	rs587648055		Missense_Mutation
**AHDC1**	p.SF1036fs	1	1								■										COSM907895	rs111827498		Frame_Shift_Del
**AHRR**	p.G391V	1	0															■			COSM3761240	rs2303738		Missense_Mutation
**AHRR**	p.P547P	3	0	■															■	■	COSM592846	rs111334085		Silent
**AIM1**	p.V819I	1	0				■														COSM174180	rs368874479		Missense_Mutation
**AIM1**	p.V1621V	3	0	■															■	■	COSM3429821,COSM3429822	rs200448332		Silent
**AK9**	p.VF295fs	1	0														■				COSM1487106	rs201441562		Frame_Shift_Del
**AL603965.1**	p.G60D	4	2								■	■	■		■						COSM4144711		1	Missense_Mutation
**ALOX5**	p.G106G	1	0		■																COSM189216	rs150281723		Silent
**ALPK1**	p.E910D	3	5	■															■	■	COSM4002908	rs35308602		Missense_Mutation
**ALPK1**	p.G870S	3	1	■															■	■	COSM4002907	rs2074380		Missense_Mutation
**ALPK1**	p.N916D	3	0	■															■	■	COSM4002909	rs2074381		Missense_Mutation
**ALS2**	p.P192P	1	0													■					COSM3425953,COSM3425954	rs368315644		Silent
**ANAPC4**	p.S777S	1	0		■																COSM149659	rs3214036		Silent
**ANGPTL7**	p.R140H	3	2	■															■	■	COSM1332677	rs28991002		Missense_Mutation
**ANKRD20A2**	p.S819Y	1	2							■											COSM3982881	rs201420500		Missense_Mutation
**ANKRD20A2**	p.D45D	1	0																■		COSM3848589		1	Silent
**ANKRD30A**	p.A886S	2	2	■															■		COSM4144666,COSM4144667	rs201628233		Missense_Mutation
**ANKRD30BL**	p.V142F	1	1											■							COSM1153486,COSM1153485		1	Missense_Mutation
**ANKRD30BL**	p.K60K	1	0					■													COSM3990586,COSM3990587	rs199974298		Silent
**ANKRD30BL**	p.H36H	1	0					■													COSM3990588,COSM3990589	rs199695856		Silent
**ANKRD30BL**	p.N29N	1	0					■													COSM3746521,COSM3746522	rs111295191		Silent
**ANKRD36**	p.S598L	1	1																	■	COSM4134194,COSM4134193	rs369954022		Missense_Mutation
**ANKRD36**	p.S598S	1	0																	■	COSM4134196,COSM4134195	rs373073058		Silent
**ANKRD36C**	p.E1646G	1	4																■		COSM1615233,COSM1615235,COSM1615234		1	Missense_Mutation
**ANKRD36C**	p.A1620V	2	1					■										■			COSM4134005,COSM4134007,C	rs76606859		Missense_Mutation
**ANKRD36C**	p.R439S	1	0							■											COSM4134126		1	Missense_Mutation

**ANKRD62**	p.L899S	1	6	COSM437909	rs150303184		Missense_Mutation
**ANKUB1**	p.R45W	3	1	COSM1198778	rs144785380		Missense_Mutation
**ANO2**	p.M397I	1	2	COSM3998903,COSM147395,CO	rs17788563		Missense_Mutation
**APLP2**	p.R467H	1	6	COSM4019335		1	Missense_Mutation
**APOF**	p.R113H	1	0	COSM147548	rs117088942		Missense_Mutation
**AQP7**	NULLL	1	1	COSM3720837,COSM1756134,COSM3720838		1	Splice_Site
**ARHGAP21**	p.R609Q	3	3	COSM146907	rs141641049		Missense_Mutation
**ARHGAP24**	p.R5L	1	2	COSM4003179	rs61758879		Missense_Mutation
**ARID5B**	p.T885T	1	0	COSM1238227	rs368683439		Silent
**ARSE**	p.T262T	1	0	COSM3759460,COSM3759459	rs17325750		Silent
**ASB15**	p.R282*	1	1	COSM249444	rs116956332		Nonsense_Mutation
**ASXL1**	p.G704R	1	7	COSM110713	rs151317625		Missense_Mutation
**ATN1**	p.-488fs	1	0	COSM431781		1	Frame_Shift_Ins
**ATP1A3**	p.P788P	1	0	COSM997170	rs150943961		Silent
**ATP2A2**	p.I686I	1	1	COSM298252	rs140766323		Silent
**ATP2C2**	p.A667A	1	0	COSM3720917	rs142596443		Silent
**AXIN1**	p.P239P	1	0	COSM4060721	rs527251369		Silent
**B3GALNT2**	p.H64R	3	5	COSM4029734		1	Missense_Mutation
**BAG3**	p.R261W	1	0	COSM1195203	rs548032105		Missense_Mutation
**BANF2**	p.S13S	3	0	COSM1165739	rs201427750		Silent
**BCR**	p.P1007P	1	0	COSM1177927	rs11558694		Silent
**BIRC7**	p.G57G	2	0	COSM3758731	rs11537908		Silent
**BMP8A**	p.S326S	1	0	COSM3751189	rs2889739		Silent
**BPHL**	p.R260C	1	7	COSM1077580	rs201051901		Missense_Mutation
**BPIFA1**	p.P72P	1	0	COSM1025890	rs143582801		Silent
**BPIFB2**	p.V409I	1	1	COSM117349	rs543901024		Missense_Mutation
**BRAP**	p.R36H	1	5	COSM238981	rs181224221		Missense_Mutation
**BRD7**	p.E339K	1	3	COSM1378189	rs538440586		Missense_Mutation
**BSND**	p.P234L	1	0	COSM1734910	rs563130435		Missense_Mutation
**BZRAP1**	p.P1356Q	1	3	COSM1644253,COSM1644252		1	Missense_Mutation
**C16orf46**	p.P294P	1	0	COSM3937188,COSM3937187	rs2287994		Silent
**C19orf40**	p.V68M	1	6	COSM3388842	rs141801484		Missense_Mutation
**C22orf15**	p.R92H	1	1	COSM4102996,COSM4102997	rs140216229		Missense_Mutation
**C22orf43**	p.DA126fs	2	0	COSM249001	rs10564183,rs142649853,rs6697		Frame_Shift_Del
**C2orf70**	p.Q177L	1	7	COSM4001633	rs2272466		Missense_Mutation
**C4orf21**	p.I1410T	3	2	COSM3683195	rs3828539		Missense_Mutation
**C4orf21**	p.Q1876Q	3	0	COSM3683194	rs80213659		Silent
**C4orf26**	p.A38V	1	2	COSM1431000,COSM1430999	rs374774709		Missense_Mutation
**C4orf40**	p.E171G	1	0	COSM215950	rs138334849		Missense_Mutation
**C5orf60**	p.L6L	1	0	COSM482626		1	Silent
**C9orf129**	p.G35G	3	0	COSM3849099	rs199652907		Silent
**CACNA1C**	p.T1870M	2	3	COSM3931613,COSM224722,CO	rs201777030		Missense_Mutation
**CACNA1G**	p.N1590N	1	0	COSM3192545,COSM3192544,C	rs201091394		Silent
**CACNA2D4**	p.R628*	1	2	COSM937789,COSM937788	rs200098356		Nonsense_Mutation
**CAMSAP3**	p.P362S	3	2	COSM3933238,COSM3933236,C	rs3745358		Missense_Mutation
**CAMTA2**	p.T1062M	1	6	COSM4067740,COSM4067741	rs557325117		Missense_Mutation
**CAP2**	p.F247F	1	0	COSM3623045	rs150167032		Silent
**CAPN5**	p.S591L	1	7	COSM196457		1	Missense_Mutation
**CASZ1**	p.D1700E	1	1	COSM1497462	rs546647724		Missense_Mutation
**CATSPERG**	p.M1137T	3	0	COSM1630902,COSM1630903	rs2286550		Missense_Mutation
**CBWD1**	p.S115N	1	0	COSM221745	rs12380		Missense_Mutation
**CBX4**	p.-507fs	2	0	COSM1386956		1	Frame_Shift_Ins
**CCBE1**	p.R162Q	2	3	COSM1199765	rs138986040		Missense_Mutation
**CCDC120**	p.A571G	1	1	COSM4156881		1	Missense_Mutation
**CCDC19**	p.R350*	1	2	COSM3476630	rs373901651		Nonsense_Mutation
**CCT8L2**	p.R133W	1	4	COSM1414734	rs576628432		Missense_Mutation
**CCZ1**	p.E214D	1	4	COSM3774292	rs143367105		Missense_Mutation

**CD209**	p.S118S	3	0								■		■						■		COSM386914,COSM386915		1	Silent
**CD34**	p.S28F	4	1												■	■		■	■		COSM4143290		1	Missense_Mutation
**CD80**	p.T210A	1	0							■											COSM3759636	rs62264488		Missense_Mutation
**CDC14B**	p.R432W	1	8													■					COSM3943127,COSM3943128,C	rs41281904		Missense_Mutation
**CDC42BPA**	p.S884S	2	1														■	■			COSM464064,COSM464063,COS	rs35017425		Silent
**CDH23**	p.I750I	1	0				■														COSM3440250,COSM3440252,C	rs368841307		Silent
**CDH23**	p.R2974R	1	0		■																COSM3998082	rs11000010		Silent
**CDH3**	p.F158F	2	0														■	■			COSM4062152,COSM4062151	rs140049723		Silent
**CDH8**	p.P389P	1	0			■															COSM972100	rs551243066		Silent
**CDK11B**	p.I487I	1	0			■															COSM3863038,COSM3863039	rs17434073		Silent
**CDR1**	p.R224C	1	0		■																COSM1116170	rs138645302		Missense_Mutation
**CENPN**	p.S107L	1	3													■					COSM973985,COSM196922,COS	rs528102999		Missense_Mutation
**CHL1**	p.F190F	3	0	■															■	■	COSM3660515	rs373871502		Silent
**CHSY1**	p.V604V	1	0				■														COSM3999455	rs3803422		Silent
**CISD2**	p.H124Y	1	7															■			COSM1732764	rs201294540		Missense_Mutation
**CITED4**	p.R57P	1	0																	■	COSM4143951	rs118006907		Missense_Mutation
**CKMT1B**	p.A362A	2	0														■	■			COSM1162242	rs138788098		Silent
**CKS1B**	p.L15L	3	0	■															■	■	COSM3996962	rs150349489		Silent
**CLEC16A**	p.G1039G	1	0					■													COSM3377763,COSM3377762	rs201512152		Silent
**CLLU1**	p.I73V	1	1				■														COSM319492	rs138705814		Missense_Mutation
**CNOT1**	NULLL	1	0								■										COSM1734949,COSM1734950		1	Splice_Site
**CNOT4**	p.D25D	1	0				■														COSM310183,COSM310182	rs185117909		Silent
**CNTN3**	p.D326D	1	0					■													COSM731600	rs143146733		Silent
**CNTNAP4**	p.I175I	2	0		■											■					COSM3999929,COSM3999928,C	rs76327478		Silent
**COG7**	p.A618T	2	1														■	■			COSM183388	rs145412851		Missense_Mutation
**COL5A1**	p.G530S	1	4					■													COSM1559848	rs61735045		Missense_Mutation
**COL9A3**	p.R577Q	1	5							■											COSM578569	rs140377811		Missense_Mutation
**COPA**	p.T99T	3	0	■															■	■	COSM3677424,COSM3677425	rs77097084		Silent
**CPNE2**	p.D444D	1	0																	■	COSM1378539	rs149575340		Silent
**CPNE7**	p.P633L	1	4		■																COSM435940	rs3751682		Missense_Mutation
**CPZ**	p.P6L	1	0																	■	COSM3760796	rs34964084		Missense_Mutation
**CREBBP**	p.R1446C	1	9									■									COSM88749	rs398124146		Missense_Mutation
**CRIPAK**	p.M48V	1	1								■										COSM447395	rs144787346		Missense_Mutation
**CT47B1**	p.EE97fs	1	0					■													COSM1465299		1	Frame_Shift_Del
**CTNNB1**	p.S33C	1	8															■			COSM5677	rs121913400		Missense_Mutation
**CTNNB1**	p.S37C	1	8		■																COSM5679	rs121913403		Missense_Mutation
**CTU1**	p.E203Q	1	4																	■	COSM4132337	rs12983578		Missense_Mutation
**CTU2**	p.R498Q	1	1																	■	COSM3755200,COSM3755201,C	rs11549835		Missense_Mutation
**CTU2**	p.V403I	3	0	■															■	■	COSM3737992,COSM3737990,C	rs4782321		Missense_Mutation
**CTU2**	p.-437fs	1	0																	■	COSM1479162,COSM1479163	rs149847195,rs571090098		Splice_Site
**CUBN**	p.R1810Q	2	5														■	■			COSM1249436	rs149622438		Missense_Mutation
**CUBN**	p.G3347R	3	1	■															■	■	COSM3997868	rs146027947		Missense_Mutation
**CYP21A2**	p.P106P	1	0					■													COSM451235	rs577302226		Silent
**DAAM2**	p.Q600R	1	3		■																COSM4004036	rs139876341		Missense_Mutation
**DAB1**	p.R26H	1	5					■													COSM1661361	rs559018567		Missense_Mutation
**DAO**	p.R22H	1	1		■																COSM1358564	rs200257378		Missense_Mutation
**DARC**	p.S152F	1	3			■															COSM139332	rs147620003		Missense_Mutation
**DCDC1**	p.R963Q	1	2							■											COSM3446711	rs539137800		Missense_Mutation
**DDI2**	p.P375L	1	1		■																COSM3677419	rs150526409		Missense_Mutation
**DDX11**	p.P368S	1	7		■																COSM3954641,COSM3954642	rs201612562		Missense_Mutation
**DDX53**	p.I348I	1	0			■															COSM3770170	rs529875384		Silent
**DEPDC7**	p.T455I	1	1			■															COSM3752331,COSM3752332	rs17852859		Missense_Mutation
**DGKG**	p.R312C	1	8					■													COSM1421485	rs199698541		Missense_Mutation
**DHRS4**	p.T193N	1	3															■			COSM1135548		1	Missense_Mutation
**DHX29**	p.G595G	1	0																	■	COSM1620392		1	Silent
**DHX57**	p.F1322F	2	1														■	■			COSM1020835	rs146662521		Silent
**DIS3L2**	p.Y265Y	1	1					■													COSM3938984,COSM3938983,C	rs202042951		Silent

**DMXL1**	p.Q2531Q	1	0	COSM4003267,COSM4003268	rs17144974		Silent
**DNAH10**	p.V3420I	2	1	COSM936646,COSM936647	rs199619464		Missense_Mutation
**DNAH2**	p.V2882M	1	7	COSM179876	rs373084871		Missense_Mutation
**DNAH3**	p.A4057T	1	2	COSM2130374,COSM2130373	rs369704821		Missense_Mutation
**DNAH8**	p.R4273C	3	7	COSM150084,COSM150085	rs78849281		Missense_Mutation
**DNAH9**	p.E2112K	1	7	COSM1725224	rs144754222		Missense_Mutation
**DND1**	p.W285*	1	1	COSM1166679	rs560601583		Nonsense_Mutation
**DND1**	p.E334K	1	0	COSM448706	rs77880328		Missense_Mutation
**DNMBP**	p.V17I	1	5	COSM70506	rs373271192		Missense_Mutation
**DOC2A**	p.R265H	2	5	COSM969753	rs200195038		Missense_Mutation
**DOCK5**	p.M987L	1	6	COSM249512	rs74894413		Missense_Mutation
**DOCK7**	p.R94Q	2	6	COSM4009276,COSM4009274,C	rs570514826		Missense_Mutation
**DPCR1**	p.Q836P	2	1	COSM3252516	rs538824498		Missense_Mutation
**DPH6**	p.P236R	1	7	COSM3678171	rs10519996		Missense_Mutation
**DPP10**	p.H82H	1	0	COSM3836640,COSM3836639	rs143448690		Silent
**DPP6**	p.E79K	1	2	COSM3747708,COSM3747707		1	Missense_Mutation
**DPY19L2**	p.I647V	1	0	COSM1128502	rs557778926		Missense_Mutation
**DPY19L2**	p.S646S	1	0	COSM3704344	rs577993411		Silent
**DSG4**	p.R86Q	1	3	COSM1718699,COSM1718698	rs368463545		Missense_Mutation
**DSPP**	p.DSSDSSN733	1	1	COSM448307		1	Frame_Shift_Del
**DST**	p.V1453L	1	4	COSM4004103,COSM4004102	rs76257231		Missense_Mutation
**DUXA**	p.R19H	1	6	COSM1002106	rs202088667		Missense_Mutation
**DYRK4**	p.R388C	1	9	COSM939610	rs201628488		Missense_Mutation
**DYTN**	p.A465T	1	0	COSM442191,COSM442190	rs373029999		Missense_Mutation
**EBF3**	p.T306T	1	0	COSM3675682,COSM3675681	rs76892753		Silent
**EFCAB13**	p.Y559*	1	1	COSM1750100	rs370786503		Nonsense_Mutation
**EFHC1**	p.R353Q	1	0	COSM1080248	rs377732929		Missense_Mutation
**EIF3A**	p.D1076D	1	0	COSM3751559	rs141834480		Silent
**ELFN2**	p.N205N	1	0	COSM1033994	rs201872308		Silent
**ELMO3**	p.R352C	1	7	COSM3754981	rs33948247		Missense_Mutation
**ELP3**	p.H30H	1	0	COSM3982502	rs146779290		Silent
**ENPP6**	p.V107I	1	1	COSM1243227	rs371373488		Missense_Mutation
**EP400**	p.Q2764Q	1	0	COSM1360187,COSM3749209	rs74479394		Silent
**EPHA1**	p.V485M	1	4	COSM1448843,COSM1448844	rs144470322		Missense_Mutation
**EPPK1**	p.V1864I	1	0	COSM3698890	rs143485250		Missense_Mutation
**EPS8**	NULLL	2	0	COSM310912		1	Splice_Site
**ERBB2**	p.V777L	1	2	COSM14062	rs121913471		Missense_Mutation
**ERBB3**	p.R1127H	1	6	COSM1363017,COSM1363018	rs2271188		Missense_Mutation
**ERVW-1**	p.R159C	2	1	COSM4162501	rs201142302		Missense_Mutation
**ETV3L**	p.T190A	1	0	COSM3750450,COSM3750449	rs61736984		Missense_Mutation
**EVPL**	p.S278S	1	0	COSM4069771	rs148449976		Silent
**EXO1**	p.R557H	2	0	COSM1205798	rs143800705		Missense_Mutation
**EXOC3**	p.L155L	2	0	COSM3761244	rs6871053		Silent
**F5**	p.T1690S	1	2	COSM244335	rs6011		Missense_Mutation
**F5**	p.P1366L	2	1	COSM3802772	rs200676405		Missense_Mutation
**F5**	p.N1208T	1	1	COSM4143011		1	Missense_Mutation
**F5**	p.Q1350Q	2	0	COSM3802773		1	Silent
**FAM104B**	p.G61S	2	2	COSM4156937,COSM4156936		1	Missense_Mutation
**FAM120B**	p.P457S	1	0	COSM1496244		1	Missense_Mutation
**FAM13B**	p.T280M	1	0	COSM175056	rs576080082		Missense_Mutation
**FAM21A**	p.P933P	1	0	COSM3998024		1	Silent
**FAM222A**	p.P348P	1	0	COSM430303	rs61748987		Silent
**FAM45A**	p.L92L	4	0	COSM1474398	rs577612097		Silent
**FAM47A**	p.S506P	1	0	COSM4005073		1	Missense_Mutation
**FAM86B1**	p.R251C	3	6	COSM3165248,COSM3165249		1	Missense_Mutation
**FAM98C**	p.K344fs	1	0	COSM1393362	rs142977446,rs59917662		Frame_Shift_Ins
**FAT2**	p.G1737G	1	0	COSM3747619		1	Silent

**FBXO10**	p.R825W	1	5			■															COSM1462416	rs573535073		Missense_Mutation
**FBXO34**	p.G704V	1	5			■															COSM3677967	rs10138395		Missense_Mutation
**FBXO45**	p.N220N	1	0							■											COSM460822	rs573601854		Silent
**FCGBP**	p.A4330A	2	0														■	■			COSM3766342	rs146289015		Silent
**FCGBP**	p.G2104R	1	0							■											COSM1481055	rs201168964		Missense_Mutation
**FCRL3**	p.V93G	1	5		■																COSM3996991,COSM3996992	rs79895668		Missense_Mutation
**FGFR3**	p.R248C	1	8														■				COSM1133721,COSM714	rs121913482		Missense_Mutation
**FLG**	p.R3240H	2	1						■					■							COSM206118	rs188183903		Missense_Mutation
**FLG**	p.H360H	1	0							■											COSM174360	rs375783519		Silent
**FLG2**	p.G597V	1	5																■		COSM1293198		1	Missense_Mutation
**FLG2**	p.H735Y	1	2								■										COSM1498355		1	Missense_Mutation
**FLG2**	p.G1033G	1	0																■		COSM1718885		1	Silent
**FLG2**	p.G1024G	3	0									■	■						■		COSM896183		1	Silent
**FLG2**	p.S788S	2	0															■	■		COSM1293193		1	Silent
**FLG2**	p.Q779Q	2	0															■	■		COSM3801902		1	Silent
**FLG2**	p.Q627Q	2	0										■						■		COSM3801903		1	Silent
**FMO2**	p.R174H	1	5					■													COSM1336193	rs148292940		Missense_Mutation
**FMO5**	p.V343I	1	1				■														COSM1185573,COSM1185574	rs201890460		Missense_Mutation
**FN1**	p.T715M	1	7		■																COSM167202,COSM1149010	rs144664306		Missense_Mutation
**FNDC7**	p.R443Q	1	1							■											COSM1688213,COSM1688212,C	rs150130697		Missense_Mutation
**FOXD4**	p.Y256Y	1	0							■											COSM3699362,COSM3699361	rs185822829		Silent
**FOXD4L1**	p.H174R	1	3							■											COSM404602	rs202243377		Missense_Mutation
**FOXO3**	p.S619S	1	2							■											COSM4160108	rs113367269		Silent
**FRG1**	p.D227Y	2	7							■				■							COSM4005732	rs199588911		Missense_Mutation
**FRG1B**	p.I110M	9	3	■		■		■	■	■	■			■	■			■			COSM3740068,COSM3740069,COSM3740067		1	Missense_Mutation
**FRG1B**	p.H8Y	1	1								■										COSM1483518,COSM1483519		1	Missense_Mutation
**FRG1B**	p.V12V	1	1								■										COSM1025508,COSM1025509		1	Silent
**FRG2C**	p.43_44KG>S	1	0																	■	COSM1425312		1	In_Frame_Del
**FRG2C**	p.T229T	3	0								■				■				■		COSM4158345		1	Silent
**FSCB**	p.A310V	1	3		■																COSM188888	rs147180788		Missense_Mutation
**FSD1**	p.T349T	1	0					■													COSM3756847	rs67511110		Silent
**FUT2**	p.I140F	1	6		■																COSM4000813	rs1047781		Missense_Mutation
**GABRB1**	p.I206I	1	0				■														COSM3604134	rs6286		Silent
**GAD1**	p.G506G	1	0													■					COSM1009468	rs200287610		Silent
**GAGE12J**	p.R15R	2	0														■	■			COSM295652	rs79608938		Silent
**GAL3ST4**	p.R453C	1	6				■														COSM1453388	rs150667953		Missense_Mutation
**GALNT14**	p.R396H	1	0														■				COSM1531747	rs146467165		Missense_Mutation
**GALNT8**	p.R173Q	2	0														■	■			COSM1512111	rs143866477		Missense_Mutation
**GALNTL5**	p.E113K	1	2		■																COSM134242	rs181262306		Missense_Mutation
**GBGT1**	p.R243H	1	7			■															COSM4163479	rs143563851		Missense_Mutation
**GFER**	p.F186F	1	0													■					COSM261081	rs142220504		Silent
**GGT1**	p.S428N	1	0					■													COSM137241	rs201313233		Missense_Mutation
**GH1**	p.A39T	3	7	■															■	■	COSM212250		1	Missense_Mutation
**GHSR**	p.A48A	1	0														■				COSM4157443,COSM4157442	rs2232166		Silent
**GIF**	p.T250T	1	0				■														COSM3450327	rs377432418		Silent
**GJB2**	p.V27I	1	5		■																COSM1740036	rs2274084		Missense_Mutation
**GLTP**	p.T172M	1	2							■											COSM1194781	rs548487246		Missense_Mutation
**GOLGA6B**	p.A431A	2	0												■	■					COSM4128382		1	Silent
**GOLGA6B**	p.D439N	1	0						■												COSM3816698	rs200016190		Missense_Mutation
**GOLGA8F**	p.L463P	1	1		■																COSM4148588		1	Missense_Mutation
**GOLGA8K**	p.N289H	1	1						■												COSM4148633	rs369957455		Missense_Mutation
**GOLGA8M**	p.F374F	4	0	■	■		■												■		COSM1478020,COSM1478019,C	rs199620151		Silent
**GOLGA8T**	p.A505A	1	0								■										COSM4148606,COSM4148605	rs199893302		Silent
**GON4L**	p.E1528E	4	0								■		■				■			■	COSM897000,COSM897001		1	Silent
**GON4L**	p.V26I	1	0					■													COSM1208485,COSM1208484,C	rs147220372		Missense_Mutation
**GPAT2**	p.R692C	1	4			■															COSM224198	rs201647131		Missense_Mutation
**GPLD1**	p.E574K	1	2				■														COSM3623202	rs140138874		Missense_Mutation

**GPR144**	p.G443R	1	1									■									COSM3763627	rs77622235		Missense_Mutation
**GPR149**	p.T23M	1	1				■														COSM1040234	rs570026624		Missense_Mutation
**GPR78**	p.R311Q	1	1														■				COSM1718993	rs140970706		Missense_Mutation
**GPRC5B**	p.A63A	1	0		■																COSM3506822	rs374426082		Silent
**GRAMD1B**	p.I321V	2	0														■	■			COSM4145427,COSM4145426	rs191981781		Missense_Mutation
**GRID2**	p.T68M	1	6					■													COSM1130995	rs34144324		Missense_Mutation
**GRIP1**	p.R817Q	2	3														■	■			COSM2157010,COSM3747932	rs145115262		Missense_Mutation
**GRM1**	p.Q1073Q	1	0				■														COSM4003716	rs362826		Silent
**HCN2**	p.FPPP714fs	1	1												■						COSM1002830	rs529324776,rs56112230		Frame_Shift_Del
**HERC2**	p.R3313R	1	0		■																COSM184053	rs117437554		Silent
**HLA-A**	p.W191G	1	4														■				COSM1442956		1	Missense_Mutation
**HLA-DQA1**	p.Q198K	1	0															■			COSM4161006		1	Missense_Mutation
**HLA-DRB1**	p.A169T	4	3												■		■	■		■	COSM3830219		1	Missense_Mutation
**HLA-DRB1**	p.A244A	2	0								■									■	COSM4006425		1	Silent
**HNMT**	p.Y186Y	1	0					■													COSM1006950	rs143554428		Silent
**HPS4**	p.P232P	1	0		■																COSM3681405,COSM3681406	rs3747132		Silent
**HRNR**	p.Q2469L	2	2													■			■		COSM3801865		1	Missense_Mutation
**HRNR**	p.E2698G	2	1										■		■						COSM3677286		1	Missense_Mutation
**HRNR**	p.V1753A	1	1													■					COSM1472632	rs145667921		Missense_Mutation
**HRNR**	p.R122W	2	1														■	■			COSM1334111	rs57277761		Missense_Mutation
**HRNR**	p.S2497S	2	0	■															■		COSM1472627		1	Silent
**HTR3A**	p.R240W	1	8		■																COSM3738865	rs147066399		Missense_Mutation
**IGSF3**	p.R429C	3	4										■			■				■	COSM1333075,COSM1333076		1	Missense_Mutation
**IGSF3**	p.E414G	1	2																	■	COSM1560062,COSM1560061		1	Missense_Mutation
**IKBKB**	p.R526Q	1	3		■																COSM150490	rs2272736		Splice_Site
**ILVBL**	p.A192V	3	7	■															■	■	COSM4000535	rs59102460		Missense_Mutation
**INADL**	p.R1261C	1	6			■															COSM1320957	rs182582824		Missense_Mutation
**INSL5**	p.R71C	1	4					■													COSM1742446	rs569176556		Missense_Mutation
**IRGQ**	p.A228A	1	0					■													COSM4078971	rs561591619		Silent
**IRS1**	p.G669S	1	2					■													COSM1210915	rs557319201		Missense_Mutation
**ITGB4**	p.S352S	2	0				■	■													COSM4130638,COSM4130637	rs187527294		Silent
**ITK**	p.P79P	1	0					■													COSM81308	rs201403794		Silent
**JAK3**	p.A753A	2	0					■								■					COSM3773848,COSM3773847	rs35458530		Silent
**JMJD1C**	p.GLV1158fs	1	1					■													COSM1163671,COSM150849	rs139722368		Frame_Shift_Del
**JMJD1C**	p.T516S	1	1					■													COSM146944	rs41274074		Missense_Mutation
**JPH3**	p.I411I	3	0	■															■	■	COSM388491	rs140244379		Silent
**JUN**	p.Q250Q	1	0				■														COSM3751326	rs11688		Silent
**KCNJ12**	p.FE332fs	1	1																	■	COSM278475		1	Frame_Shift_Del
**KCNJ12**	p.L271L	1	0																	■	COSM312199		1	Silent
**KCNJ12**	p.H272H	1	0																	■	COSM312200		1	Silent
**KDM4E**	p.I63I	1	0			■															COSM933556,COSM933555	rs1960159		Silent
**KDR**	p.R944Q	1	1													■					COSM1056139	rs374755237		Missense_Mutation
**KIAA1009**	p.L542L	1	0				■														COSM3762204	rs41271605		Silent
**KIAA1549L**	p.A692A	1	0														■				COSM79554	rs531225970		Silent
**KIF17**	p.R1001Q	1	5		■																COSM182818	rs574381737		Missense_Mutation
**KIF26B**	p.I1613I	2	0														■	■			COSM1340879,COSM1340880	rs7530669		Silent
**KIR3DL2**	p.S98T	1	4		■																COSM148748	rs199603670		Missense_Mutation
**KLHL1**	p.V458V	1	0			■															COSM1367609	rs376887058		Silent
**KLHL33**	p.A82V	1	6															■			COSM3936452,COSM3936453	rs569861281		Missense_Mutation
**KLK8**	p.P41L	3	5	■															■	■	COSM35633		1	Missense_Mutation
**KNSTRN**	p.R75L	1	1					■													COSM147878	rs7169404		Missense_Mutation
**KNTC1**	p.A1079A	1	0							■											COSM261708	rs538519234		Silent
**KRT1**	p.S254S	2	0														■	■			COSM940811	rs2741155		Silent
**KRT33B**	p.R277C	1	6				■														COSM302521	rs558957351		Missense_Mutation
**KRT33B**	p.S262S	1	0		■																COSM186731	rs553149537		Silent
**KRT6C**	p.N21S	2	0							■							■				COSM2010664		1	Missense_Mutation
**KRT85**	p.E352K	3	8	■															■	■	COSM1362469	rs370478744		Missense_Mutation

**KRT86**	p.R116R	1	0					■													COSM4147201	rs58580222		Silent
**KRTAP1-1**	p.S34C	1	0																	■	COSM245362		1	Missense_Mutation
**KRTAP19-1**	p.S52G	1	1										■								COSM1413637	rs562711051		Missense_Mutation
**KRTAP4-2**	p.T69T	2	0												■		■				COSM1750022,COSM1750021		1	Silent
**KRTAP4-4**	p.T139I	1	1									■									COSM4000156	rs74572864		Missense_Mutation
**KRTAP4-4**	p.H62R	2	0											■					■		COSM1521043		1	Missense_Mutation
**KRTAP4-6**	p.R196R	1	0								■										COSM1493893	rs551614044		Silent
**KRTAP4-6**	p.I191I	1	0								■										COSM1302799		1	Silent
**KRTAP5-10**	p.C188C	2	0								■					■					COSM1293435		1	Silent
**KRTAP5-3**	p.G179G	1	0														■				COSM1352871		1	Silent
**KRTAP5-5**	p.PYCCQSSCC	1	1						■												COSM253911	rs576867883,rs71025765		Frame_Shift_Del
**KSR1**	p.F473F	2	0	■																■	COSM3680116,COSM3680117	rs2289568		Silent
**KSR2**	p.A373T	2	0														■	■			COSM3416526,COSM3416525	rs376902515		Missense_Mutation
**L1TD1**	p.TSGLEEEEE	1	1			■															COSM1343768	rs532563709		Frame_Shift_Del
**LEPRE1**	p.E723K	1	1							■											COSM3489753	rs146002380		Missense_Mutation
**LGALS9C**	p.V184M	1	1																	■	COSM4129655	rs201967116		Missense_Mutation
**LIG3**	p.R445H	3	4	■															■	■	COSM977784,COSM977783	rs567712080		Missense_Mutation
**LILRB1**	p.A319T	2	1		■										■						COSM1481494	rs201552016		Missense_Mutation
**LILRB2**	p.G298S	1	4		■																COSM3389345	rs142624499		Missense_Mutation
**LILRB3**	p.Y297Y	1	0					■													COSM3757188	rs78279726		Silent
**LILRB4**	p.R367R	1	0		■																COSM148746	rs200441190		Silent
**LOXHD1**	p.Y518C	1	8														■				COSM988529,COSM988528	rs201536647		Missense_Mutation
**LPCAT1**	p.H266Y	1	3								■										COSM1542772	rs375249372		Missense_Mutation
**LRP1**	p.N1645N	1	0															■			COSM335799	rs375652681		Silent
**LRP10**	p.R562H	1	6													■					COSM432858	rs142153001		Missense_Mutation
**LRP1B**	p.C3787C	3	1	■															■	■	COSM1564791	rs35757222		Silent
**LRP2**	p.L2185F	1	6					■													COSM3569766	rs143884041		Missense_Mutation
**LRP2**	p.C3114C	1	0							■											COSM716812	rs199803029		Silent
**LRP3**	p.R226R	1	0			■															COSM3766318,COSM3766317	rs3745977		Silent
**LRP5**	p.P943P	1	0					■													COSM544958	rs201018263		Splice_Site
**LRRC37A3**	p.V449I	2	0					■				■									COSM328586		1	Missense_Mutation
**LRRC37A3**	p.A321A	1	0								■										COSM4130502		1	Silent
**LRRC49**	p.D120D	1	0		■																COSM237683	rs146593930		Silent
**LRRTM3**	p.I530R	1	5											■							COSM3998053,COSM3998052	rs75363119		Missense_Mutation
**MADD**	p.A361A	1	0		■																COSM1492611	rs34602269		Silent
**MAGEC1**	p.R290S	2	0									■							■		COSM3232920		1	Missense_Mutation
**MAMDC2**	p.D680N	1	1				■														COSM3926894	rs531060041		Missense_Mutation
**MAML1**	p.A694A	1	0							■											COSM3761165	rs62406189		Silent
**MAML2**	p.R422Q	1	4					■													COSM124296	rs559230872		Missense_Mutation
**MAML3**	p.QQQQQ505E	2	0												■				■		COSM1051420,COSM1051421		1	Frame_Shift_Del
**MAP10**	p.K593E	1	1		■																COSM3997340,COSM146823	rs59165168		Missense_Mutation
**MAP2**	p.D1077D	1	0				■														COSM4090722	rs550587107		Silent
**MAPK4**	p.F28F	2	0		■		■														COSM4000420	rs3752086		Silent
**MBTPS1**	p.R130Q	1	6			■															COSM171357	rs148577579		Missense_Mutation
**MCCD1**	p.T53M	1	0				■														COSM4160832	rs78957773		Missense_Mutation
**MCM3AP**	p.D1449E	1	1		■																COSM3758986	rs17183220		Missense_Mutation
**MCM3AP**	p.E803E	1	0		■																COSM3758988	rs17182739		Silent
**ME3**	p.A418T	1	4								■										COSM3752790	rs3182235		Missense_Mutation
**MESP2**	p.G187G	1	0																	■	COSM3754568		1	Silent
**MET**	p.T992I	3	5	■															■	■	COSM707	rs56391007		Missense_Mutation
**METTL15**	p.T67A	3	0	■															■	■	COSM3998383,COSM3998382	rs141648004		Missense_Mutation
**METTL2B**	p.C59R	1	1							■											COSM1131716	rs2896399		Missense_Mutation
**MGAM**	p.T2748M	1	2				■														COSM2862638,COSM2862639,C	rs370916883		Missense_Mutation
**MKI67**	p.E3102G	3	3	■															■	■	COSM3751609	rs34750407		Missense_Mutation
**MKI67**	p.R2845H	3	3	■															■	■	COSM3751610	rs11016072		Missense_Mutation
**MKI67**	p.T2337N	3	3	■															■	■	COSM3751612	rs7083622		Missense_Mutation
**MKL1**	p.A515T	1	0																	■	COSM3749768	rs72655385		Missense_Mutation

**MOG**	p.P72H	1	4				■														COSM228993	rs138169338		Missense_Mutation
**MRS2**	p.V178I	1	4		■																COSM1076465,COSM3928250	rs200245617		Missense_Mutation
**MSI1**	p.A276A	1	1		■																COSM371564	rs150872271		Silent
**MSRB2**	p.E46G	4	1	■	■														■	■	COSM3997897	rs2296466		Missense_Mutation
**MTOR**	p.R311C	1	7			■															COSM4020719	rs200901835		Missense_Mutation
**MTUS2**	p.S872S	1	0			■															COSM946570	rs140464903		Silent
**MUC12**	p.R726S	1	1																■		COSM4161560,COSM4161559		1	Missense_Mutation
**MUC12**	p.L3235F	2	1														■		■		COSM3684746,COSM3684745		1	Missense_Mutation
**MUC12**	p.P3420P	1	0								■										COSM4006574,COSM4006575	rs376455647		Silent
**MUC12**	p.S4694S	1	0																■		COSM4161619,COSM4161620	rs78853018		Silent
**MUC16**	p.T11030A	2	1									■							■		COSM3718543,COSM3718544		1	Missense_Mutation
**MUC17**	p.Q2374E	1	1											■							COSM162673	rs555548795		Missense_Mutation
**MUC17**	p.N2706S	1	1										■								COSM1673398	rs201007776		Missense_Mutation
**MUC17**	p.I3212M	1	1																■		COSM1732705		1	Missense_Mutation
**MUC17**	p.I3324T	1	1										■								COSM452042		1	Missense_Mutation
**MUC17**	p.A673A	2	0												■		■				COSM4161631		1	Silent
**MUC17**	p.V2428V	1	0														■				COSM3631215		1	Silent
**MUC2**	p.T1729T	1	0																	■	COSM220047,COSM220046	rs111440994		Silent
**MUC2**	p.M1776V	1	0													■					COSM4145284,COSM4145283	rs56189540		Missense_Mutation
**MUC22**	p.T1106T	1	0																	■	COSM4160627		1	Silent
**MUC22**	p.T1145T	1	0																	■	COSM4160628		1	Silent
**MUC4**	p.A2569V	1	2																	■	COSM4157739,COSM4157738		1	Missense_Mutation
**MUC4**	p.V1001E	1	1																	■	COSM1131502,COSM1617393		1	Missense_Mutation
**MUC5B**	p.G2588R	1	2																	■	COSM1475202,COSM1475201	rs200110372		Missense_Mutation
**MUC5B**	p.M3122T	1	2							■											COSM3998324,COSM3998323	rs58125533		Missense_Mutation
**MUC6**	p.P1569L	3	2														■	■	■		COSM3773559,COSM3773560		1	Missense_Mutation
**MUC6**	p.S1524S	1	0																	■	COSM4145137,COSM4145136	rs77630889		Silent
**MUC6**	p.T1523T	1	0																	■	COSM4145139,COSM4145138	rs113063572		Silent
**MYBPC2**	p.P642P	1	0			■															COSM3371440	rs558923030		Silent
**MYO10**	p.A1003T	3	3	■															■	■	COSM3927478,COSM3927479	rs72750543		Missense_Mutation
**MYO10**	p.P1369P	1	0			■															COSM449271	rs16868979		Silent
**MYO7B**	p.V531I	1	1			■															COSM3694861,COSM3694860	rs373230646		Missense_Mutation
**MYO9A**	p.R1217Q	1	3													■					COSM276235	rs193029114		Missense_Mutation
**MYO9B**	NULLL	3	0						■	■							■				COSM1736016,COSM1736015		1	Splice_Site
**MYOM2**	p.T1404I	1	6		■																COSM144677	rs147501499		Missense_Mutation
**NAGPA**	p.A449T	1	0																	■	COSM971306	rs199604440		Missense_Mutation
**NBAS**	p.R1004Q	1	4				■														COSM4133182	rs16862653		Missense_Mutation
**NBPF10**	p.I20M	4	1		■	■			■					■							COSM4142523,COSM4142522	rs9424711		Missense_Mutation
**NBPF12**	p.R104*	3	1									■	■	■							COSM3801673,COSM3801672		1	Nonsense_Mutation
**NBPF16**	p.G665V	1	2													■					COSM3747548	rs587602537		Missense_Mutation
**NCAPG2**	p.E867D	3	1	■															■	■	COSM3685126,COSM3685125	rs3214000		Missense_Mutation
**NCOA3**	p.MQQQ1243fs	1	0							■											COSM1483713		1	Frame_Shift_Del
**NCOA3**	p.Q1258Q	1	0											■							COSM1027546	rs151060280		Silent
**NCSTN**	p.S341S	3	1	■															■	■	COSM3677426,COSM3677427	rs2296270		Silent
**NDNF**	p.T375I	1	3							■											COSM3917058	rs143734180		Missense_Mutation
**NEB**	p.N7182N	2	0		■					■											COSM4001211,COSM4001212,C	rs149510427		Silent
**NEB**	NULLL	3	0					■	■							■					COSM1733400,COSM1733401,COSM1733402		1	Splice_Site
**NEBL**	p.T728A	1	1		■																COSM146901	rs71535732		Missense_Mutation
**NEFH**	p.A588V	1	1									■									COSM4137376	rs611898		Missense_Mutation
**NEFM**	p.P657P	1	0									■									COSM3647578	rs371898352		Silent
**NEFM**	p.P657P	1	0														■				COSM1294419		1	Silent
**NFAM1**	p.P158P	1	0							■											COSM1416634	rs143813591		Silent
**NFATC1**	p.G620R	1	1					■													COSM304241	rs372608876		Missense_Mutation
**NHSL1**	p.Y787Y	3	0	■															■	■	COSM3948079,COSM3948080	rs573566170		Silent
**NLRP3**	p.P317L	1	2					■													COSM2052586	rs180177462		Missense_Mutation
**NLRP8**	p.T482T	1	0							■											COSM1001748	rs369750431		Silent
**NPBWR2**	p.R237H	1	0						■												COSM4100576	rs370207328		Missense_Mutation

**NPIPA5**	p.P321L	1	2										■								COSM3678565,COSM3678566		1	Missense_Mutation
**NPIPB11**	p.P499T	1	2																■		COSM3765884,COSM3765883		1	Missense_Mutation
**NPIPB15**	p.A107A	1	0					■													COSM4129342,COSM4129343	rs201056247		Silent
**NPR1**	p.P450S	3	3	■															■	■	COSM3474477	rs556561976		Missense_Mutation
**NUBP1**	p.A252A	1	0					■													COSM1608887	rs565678589		Silent
**NUDT17**	p.S209S	1	1										■								COSM1320032	rs142717400		Silent
**NUP62**	p.G139S	1	2				■														COSM148689	rs3745489		Missense_Mutation
**NUTM2B**	p.P67L	1	2														■				COSM1966515	rs199845914		Missense_Mutation
**NWD1**	p.T1145T	1	0				■														COSM992549,COSM992548	rs575558256		Silent
**OAS2**	p.E141K	1	0			■															COSM1706314,COSM1706313	rs139641023		Missense_Mutation
**OBSCN**	p.P5577P	1	0							■											COSM262503,COSM3864676,CO	rs565920635		Silent
**ODF3**	p.A140T	2	5	■																■	COSM1582081	rs140404766		Missense_Mutation
**ODF3**	p.T42M	1	3					■													COSM925840	rs140013613		Missense_Mutation
**OPN1LW**	p.I178V	1	0			■															COSM3964941,COSM3964942	rs145009674		Missense_Mutation
**OPRL1**	p.A121A	2	0														■	■			COSM273040	rs145759308		Silent
**OPTC**	p.L270L	1	0				■														COSM3750781	rs72743637		Silent
**OR10G8**	p.G14G	3	0	■															■	■	COSM3752192	rs76493378		Silent
**OR10G8**	p.V28I	2	0														■	■			COSM1178540	rs202220125		Missense_Mutation
**OR10H1**	p.A224D	1	5				■														COSM328549	rs62619246		Missense_Mutation
**OR13C3**	p.R120I	1	5							■											COSM1103480		1	Missense_Mutation
**OR1L3**	p.V279A	1	3				■														COSM3763609	rs74634130		Missense_Mutation
**OR1L3**	p.V106A	1	0				■														COSM3763607	rs16912096		Missense_Mutation
**OR1L3**	p.N121D	1	0				■														COSM3763608	rs16912099		Missense_Mutation
**OR2L3**	p.T202A	1	2				■														COSM2232958	rs61525219		Missense_Mutation
**OR2L8**	p.G212A	4	0	■													■	■	■		COSM226498	rs200574966		Missense_Mutation
**OR2T35**	p.A317V	1	0							■											COSM4143673	rs150878651		Missense_Mutation
**OR2T35**	p.G309G	3	0							■		■				■					COSM3773489	rs74901534		Silent
**OR2T4**	p.M336V	3	0	■															■	■	COSM1260194	rs201310603		Missense_Mutation
**OR3A2**	p.R238H	1	0					■													COSM3748011	rs201033738		Missense_Mutation
**OR4A47**	p.S236P	1	5		■																COSM3752405	rs76991989		Missense_Mutation
**OR4C16**	p.V239V	3	0	■															■	■	COSM928038	rs367685638		Silent
**OR4F6**	p.C141G	1	6			■															COSM254880	rs78066807		Missense_Mutation
**OR51E2**	p.G207G	1	0			■															COSM3752378	rs1123990		Silent
**OR51E2**	p.C96C	1	0			■															COSM3752379	rs12575331		Silent
**OR51E2**	p.L60L	1	0			■															COSM3752380	rs12576387		Silent
**OR51M1**	p.V154L	1	0														■				COSM322345	rs535846608		Missense_Mutation
**OR5D13**	p.A283A	3	0	■															■	■	COSM276522	rs150209335		Silent
**OR5M10**	p.V276V	1	0													■					COSM928428	rs267603010		Silent
**OR5T2**	p.A195T	1	3		■																COSM3752472	rs77295387		Missense_Mutation
**OR7D4**	p.A119V	1	6		■																COSM4083605	rs570640089		Missense_Mutation
**OR7E24**	p.F11fs	1	0								■										COSM1398257	rs201985790		Frame_Shift_Del
**OR8D2**	p.M268V	1	0		■																COSM3676012	rs74550564		Missense_Mutation
**OR8U1**	p.H229R	1	0																	■	COSM4145880		1	Missense_Mutation
**OR8U1**	p.H229H	1	0																	■	COSM4145881		1	Silent
**OR9G1**	p.A132V	1	1		■																COSM3752484	rs78340277		Missense_Mutation
**OSBPL10**	p.V553I	1	2				■														COSM1617469,COSM1617468	rs141856244		Missense_Mutation
**OSBPL3**	p.A227A	1	0		■																COSM1180210	rs78399024		Silent
**OTOA**	p.E787*	1	1		■																COSM968503,COSM1588333	rs200988634		Nonsense_Mutation
**OVCH1**	p.T701T	1	0		■																COSM4041319	rs117919494		Silent
**P2RX7**	p.I355I	1	0			■															COSM430527	rs200138583		Silent
**PACSIN3**	p.R111R	3	0	■															■	■	COSM1604589	rs2291445		Silent
**PAK2**	p.D173E	1	1							■											COSM4116433,COSM4116434	rs565026135		Missense_Mutation
**PALM2-AKA**	p.A844A	2	0														■	■			COSM313880,COSM313881	rs76059538		Silent
**PAPPA**	p.D1579D	1	0			■															COSM1459618	rs140928157		Silent
**PARK2**	p.T240M	1	5				■														COSM98176,COSM3722940	rs137853054		Missense_Mutation
**PCDHA6**	p.P621L	1	2											■							COSM3696958,COSM3696959		1	Missense_Mutation
**PCDHB10**	p.T573T	1	0										■								COSM4159533		1	Silent

**PCDHB2**	p.L493P	2	6	■															■		COSM4003336	rs17844375		Missense_Mutation
**PCDHB2**	p.R463R	1	0									■									COSM4159503	rs142417694		Silent
**PCDHB3**	p.V486V	1	0				■														COSM1062559	rs151234490		Silent
**PCDHB5**	p.S464N	2	3	■															■		COSM3760981	rs17844422		Missense_Mutation
**PCDHGB1**	p.R531R	1	0					■													COSM1063060	rs543692676		Silent
**PCK1**	p.V368I	1	2			■															COSM4134693,COSM4134692	rs1804160		Missense_Mutation
**PCLO**	p.D3197E	3	3	■															■	■	COSM3698557,COSM3698559,C	rs200530952		Missense_Mutation
**PCMTD1**	p.N312N	1	0									■									COSM4163015		1	Silent
**PCNT**	p.R2753H	1	2		■																COSM3693851	rs743346		Missense_Mutation
**PCNT**	p.P2377L	1	1		■																COSM3693848	rs61735814		Missense_Mutation
**PCNT**	p.P179P	1	0										■								COSM1031622		1	Silent
**PCNT**	p.H237Q	1	0		■																COSM3693844	rs34500739		Missense_Mutation
**PCNT**	p.D1878D	1	0		■																COSM1414630	rs61735811		Silent
**PCNT**	p.P2358P	1	0		■																COSM3693847	rs61735815		Silent
**PCNT**	p.I2495I	1	0		■																COSM3693849	rs61735817		Silent
**PDE4DIP**	p.R2202W	4	6	■													■	■	■		COSM4021587,COSM4021589,C	rs146619065		Missense_Mutation
**PES1**	p.A169V	2	1														■	■			COSM1033309	rs149639037		Missense_Mutation
**PGAM2**	p.G97D	1	8		■																COSM150277	rs77938727		Missense_Mutation
**PGM1**	p.D385D	1	0		■																COSM1343841,COSM1343842	rs375432694		Silent
**PHF1**	p.P425L	3	2	■															■	■	COSM1078042	rs530537499		Missense_Mutation
**PHYH**	p.D269N	3	1	■															■	■	COSM916255	rs538686726		Missense_Mutation
**PI4K2B**	p.S78P	1	0													■					COSM4159010	rs313549		Missense_Mutation
**PIBF1**	NULLL	1	0														■				COSM253063		1	Splice_Site
**PIEZO1**	p.R720H	1	2																	■	COSM3755215	rs35265318		Missense_Mutation
**PIEZO1**	p.Y2444Y	2	1	■																■	COSM3680054,COSM3680053	rs140778010		Silent
**PIEZO1**	p.QE749fs	1	0																	■	COSM435924	rs144777557		Frame_Shift_Del
**PIEZO1**	p.V677V	1	0																	■	COSM3755217	rs34908386		Silent
**PIEZO1**	p.L514L	1	0																	■	COSM3755218	rs78905828		Silent
**PIEZO1**	p.T455T	1	0																	■	COSM3755219	rs61742623		Silent
**PIK3C2G**	p.A261E	2	3														■	■			COSM3753079,COSM3753078	rs7133666		Missense_Mutation
**PIK3C2G**	p.C322C	2	0														■	■			COSM3753081,COSM3753080	rs7301521		Silent
**PITPNM1**	p.P641L	1	1		■																COSM3452330	rs547678982		Missense_Mutation
**PJA2**	p.S282L	1	1											■							COSM1696018		1	Missense_Mutation
**PKD1**	p.T938M	1	1					■													COSM226770	rs148709380		Missense_Mutation
**PKD1**	p.D910D	2	0	■															■		COSM3754781	rs35965348		Silent
**PKD1**	p.P900P	2	0	■															■		COSM3754782	rs35667726		Silent
**PKHD1L1**	p.T3322T	1	0		■																COSM1094943	rs199629163		Silent
**PLA2R1**	p.T266M	1	3				■														COSM207430	rs190273929		Missense_Mutation
**PLEC**	p.R433Q	2	4														■	■			COSM454202,COSM1489112,CO	rs138924815		Missense_Mutation
**PLEC**	p.A2745V	1	3																	■	COSM3897885,COSM3897884,C	rs370351676		Missense_Mutation
**PLEC**	p.P652P	3	0	■															■	■	COSM1455525,COSM1455524,C	rs199968254		Silent
**PLEKHG3**	p.R850H	3	0	■															■	■	COSM1370592	rs138505394		Missense_Mutation
**PLEKHG4B**	p.P735P	1	0		■																COSM1065398	rs530135061		Silent
**PLIN4**	p.K335R	1	4									■									COSM4132092,COSM4132091	rs139917681		Missense_Mutation
**PLIN4**	p.V903M	1	0																■		COSM226374,COSM1600095		1	Missense_Mutation
**PLIN4**	p.D750D	1	0																	■	COSM1525710,COSM1525711		1	Silent
**PLSCR1**	p.R5Q	3	0	■															■	■	COSM1039592	rs372003252		Missense_Mutation
**PLXNA1**	p.S1413S	1	0				■														COSM3695794	rs147741166		Silent
**PMP2**	p.R27H	1	1				■														COSM3395425	rs201080587		Missense_Mutation
**POTEB2**	p.G242G	2	0														■	■			COSM1744146,COSM1744145	rs549164573		Silent
**POTEC**	p.G272G	3	0										■			■		■			COSM1480234		1	Silent
**POTED**	p.E87D	5	0											■		■	■		■	■	COSM4134835		1	Missense_Mutation
**POTED**	p.M93I	1	0																	■	COSM4134837		1	Missense_Mutation
**POTED**	p.S527S	1	0				■														COSM3668509	rs572152710		Silent
**POTEF**	p.C103R	1	2				■														COSM3682326,COSM1723815,C	rs552690286		Missense_Mutation
**POTEG**	p.P434T	1	1					■													COSM3773695	rs200779890		Missense_Mutation
**POTEI**	p.A930V	1	4				■														COSM3836819,COSM3836820	rs190931019		Missense_Mutation

**POTEM**	p.A146T	1	4	COSM3999184,COSM3999183	rs200978838		Missense_Mutation
**POTEM**	p.L379S	2	2	COSM4147953,COSM4147952		1	Missense_Mutation
**POTEM**	p.L143L	1	0	COSM3999185,COSM3999186	rs377394087		Silent
**PPP1R14B**	p.G43G	2	0	COSM1723240	rs1063811		Silent
**PRAMEF11**	p.T93K	3	0	COSM462790	rs2486717		Missense_Mutation
**PRAMEF19**	p.N379K	1	1	COSM4142403,COSM4142402		1	Missense_Mutation
**PRAMEF4**	p.K135T	1	2	COSM4142366		1	Missense_Mutation
**PRAMEF5**	p.H122R	1	1	COSM3747523	rs199562274		Missense_Mutation
**PRB1**	p.K96K	1	0	COSM314402		1	Silent
**PRCP**	p.T444S	1	3	COSM3752775,COSM3752776	rs2228312		Missense_Mutation
**PRDM7**	p.T135K	1	2	COSM3999991,COSM148139	rs7206111		Missense_Mutation
**PRELP**	p.N136N	1	0	COSM3750780	rs2233725		Silent
**PRELP**	p.P187P	1	0	COSM902480	rs531394874		Silent
**PRG4**	p.K710K	1	0	COSM399029		1	Silent
**PROSER1**	NULLL	1	0	COSM253173		1	Splice_Site
**PRR14L**	p.T961I	5	2	COSM4156166,COSM4156167	rs140079		Missense_Mutation
**PRR5**	p.D148D	1	1	COSM1641575,COSM1641573,C	rs199883341		Silent
**PTGIR**	p.R77H	1	2	COSM4127733	rs199787627		Missense_Mutation
**PTPN23**	p.P405P	1	0	COSM22179	rs201767013		Silent
**PTPRCAP**	p.A119T	1	1	COSM139914	rs138577657		Missense_Mutation
**PTPRD**	p.N962N	1	0	COSM150575	rs370343083		Silent
**PTPRN2**	p.A786A	1	0	COSM81625	rs139538258		Silent
**PYGO1**	p.A294A	2	0	COSM963073	rs535636770		Silent
**R3HDM2**	p.R962H	1	5	COSM3753411,COSM3753409,C	rs78607331		Missense_Mutation
**RABGAP1**	p.K598R	2	3	COSM150707,COSM150708	rs75992542		Splice_Site
**RAF1**	p.S257L	1	6	COSM3586964,COSM181063	rs80338796		Missense_Mutation
**RAI1**	p.S428S	2	0	COSM4064329,COSM4064328	rs113249215		Silent
**RBMX**	p.A78T	1	2	COSM1132397	rs199717308		Missense_Mutation
**RBMX**	NULLL	2	0	COSM1490534,COSM1490533	rs369256758		Start_Codon_Ins
**RBMXL1**	p.P96L	1	3	COSM1561045	rs530375965		Missense_Mutation
**RELN**	p.V3365I	1	3	COSM78407	rs115035120		Missense_Mutation
**REST**	p.S750T	1	1	COSM3302227	rs139336427		Missense_Mutation
**RETSAT**	p.V424I	1	0	COSM3758389	rs140300131		Missense_Mutation
**REXO1L1**	p.V650A	1	2	COSM1264029,COSM1264030	rs376763410		Missense_Mutation
**RGPD2**	p.E898E	2	0	COSM3840202,COSM3840203	rs200044571		Silent
**RNF125**	p.R58H	3	2	COSM1388418	rs201216500		Missense_Mutation
**RNF157**	p.T139M	1	3	COSM4130652,COSM4130653	rs61744818		Missense_Mutation
**RNF175**	p.R216Q	1	2	COSM3974768,COSM3974767,C	rs181124344		Missense_Mutation
**RNF215**	p.L269L	3	1	COSM2937060	rs200160181		Silent
**ROBO4**	p.S307S	3	0	COSM257911	rs534292971		Silent
**RP11-166B2.1**	p.P301P	1	0	COSM1317723,COSM1317722		1	Silent
**RP11-514P8.7**	p.A280S	1	4	COSM1488087		1	Missense_Mutation
**RP1L1**	p.E1459K	1	2	COSM1582736	rs142510965		Missense_Mutation
**RP1L1**	p.E1882D	2	0	COSM3762974	rs111646478		Missense_Mutation
**RPS6KA2**	p.N476N	1	0	COSM3393977,COSM3393978,C	rs147557165		Silent
**RPTN**	p.T747T	1	0	COSM1498495		1	Silent
**RPTN**	p.P526T	2	0	COSM341811		1	Missense_Mutation
**RPTN**	p.S449S	2	0	COSM3473858		1	Silent
**SAC3D1**	p.R260C	2	1	COSM1317282	rs193165731		Missense_Mutation
**SALL1**	p.N1305N	1	0	COSM971341	rs140524372		Silent
**SASH1**	p.P1173P	1	0	COSM594672	rs111485793		Silent
**SCARA5**	p.R43W	1	8	COSM241517	rs138480005		Missense_Mutation
**SCN3A**	p.D1803N	3	6	COSM300670	rs3731762		Missense_Mutation
**SCN7A**	p.A1313V	1	4	COSM3990718,COSM3990719	rs6760593		Missense_Mutation
**SCN7A**	p.V187I	1	3	COSM673628,COSM673629	rs557709889		Missense_Mutation
**SCN9A**	p.I1214I	3	0	COSM4001282,COSM4001283	rs77144869		Silent
**SEC24D**	p.S542S	4	0	COSM149730	rs76459466		Silent

**SEL1L3**	p.W1054C	1	3	COSM4003060,COSM4003062,C	rs2286866		Missense_Mutation
**SELPLG**	p.QPVPTEAQT	1	1	COSM1358534,COSM1358533	rs372173288,rs63748999		Frame_Shift_Del
**SEMA3C**	p.V321A	1	7	COSM1623125,COSM3663330	rs140244551		Missense_Mutation
**SEMG1**	p.R372S	1	0	COSM400596		1	Missense_Mutation
**SERHL2**	p.V296V	1	0	COSM1484278	rs147451679		Silent
**SESN2**	p.T320A	1	0	COSM3677647	rs2274848		Missense_Mutation
**SETX**	p.D2372N	3	6	COSM1236144	rs150673589		Missense_Mutation
**SF3B1**	p.K700E	2	8	COSM84677	rs559063155		Missense_Mutation
**SFXN4**	p.A226V	1	1	COSM1162900	rs151157939		Missense_Mutation
**SGCE**	p.R173H	1	5	COSM1093291,COSM1597237	rs548306335		Missense_Mutation
**SH3BP4**	p.S326S	3	0	COSM3758056	rs35039245		Silent
**SHARPIN**	p.R365H	1	6	COSM1193858	rs112552278		Missense_Mutation
**SHISA7**	p.T109P	1	5	COSM4132619		1	Missense_Mutation
**SIDT2**	p.A644S	1	2	COSM318023	rs118183792		Missense_Mutation
**SIPA1L1**	p.A975A	1	0	COSM553572	rs201048906		Silent
**SIRPB1**	p.L95F	1	1	COSM4134247		1	Missense_Mutation
**SLC22A2**	p.R403C	1	8	COSM1075644	rs568574662		Missense_Mutation
**SLC22A25**	NULLL	1	0	COSM3752592	rs1783657		Splice_Site
**SLC2A3**	p.A444A	1	0	COSM4147481		1	Silent
**SLC2A3**	p.L443L	1	0	COSM3813057		1	Silent
**SLC2A3**	p.H425Y	1	0	COSM1993161		1	Splice_Site
**SLC4A10**	p.N186S	1	2	COSM1590794,COSM1008285	rs537192317		Missense_Mutation
**SLC4A4**	p.G380D	1	6	COSM328669,COSM328670	rs148635969		Missense_Mutation
**SLC7A4**	p.R342H	3	8	COSM1032436	rs148642327		Missense_Mutation
**SLC9B1P1**	p.G125G	1	0	COSM3682788	rs73618462		Silent
**SMAD4**	p.R265H	1	9	COSM14122	rs377767347		Missense_Mutation
**SMARCA2**	p.QQ236fs	1	0	COSM2156556,COSM2156557		1	Frame_Shift_Del
**SMC1B**	p.D1064N	1	3	COSM1035021,COSM1035020	rs370210187		Missense_Mutation
**SNRPC**	p.R14L	2	0	COSM4006481		1	Missense_Mutation
**SPANXN2**	p.L141L	3	0	COSM3964823,COSM3964824		1	Silent
**SPATA2**	p.L150L	1	0	COSM1495064	rs148068614		Silent
**SPATA3**	p.T36T	1	0	COSM1565694		1	Silent
**SPATA31A2**	p.P155P	3	0	COSM3685645		1	Silent
**SPATA31A3**	p.T168I	1	1	COSM4163873,COSM4163872	rs200010477		Missense_Mutation
**SPHK2**	p.G90G	1	0	COSM3749529,COSM3749530,C	rs61748787		Silent
**SPINK4**	p.R94Q	1	3	COSM3906879,COSM3906880	rs140712741		Missense_Mutation
**SPOCD1**	p.P727P	1	0	COSM4031417	rs146160927		Silent
**SRA1**	p.V110fs	3	1	COSM1192026		1	Frame_Shift_Ins
**SRGAP2**	p.R95H	1	6	COSM3773467,COSM3773468,C	rs2987927		Splice_Site
**SSX7**	p.D182E	1	2	COSM1331810	rs112382781		Missense_Mutation
**ST8SIA6**	p.T95M	1	1	COSM465515	rs375777148		Missense_Mutation
**STARD13**	p.T175M	1	1	COSM3999071	rs9568878		Missense_Mutation
**STAT4**	NULLL	1	0	COSM1733732		1	Splice_Site
**STIM1**	p.R643H	1	5	COSM1353761	rs140080199		Missense_Mutation
**STK33**	p.E98D	3	2	COSM3998611,COSM3998610	rs34525052		Missense_Mutation
**SUN1**	p.G564V	1	4	COSM3982281,COSM3982282,C	rs77058871		Missense_Mutation
**SUN1**	p.R718R	1	0	COSM3982285,COSM3982286,C	rs61744747		Silent
**SVEP1**	p.T2870P	1	1	COSM4163205		1	Missense_Mutation
**SYNE1**	p.R5780Q	1	2	COSM328205,COSM328206	rs76160752		Missense_Mutation
**SYNE1**	p.L885V	1	1	COSM3761594,COSM3761595,C	rs17082709		Missense_Mutation
**SYNGAP1**	p.A601A	1	0	COSM3366468,COSM3366467	rs77934848		Silent
**SYNM**	p.A895V	1	1	COSM2141590,COSM2141589	rs372660853		Missense_Mutation
**TAP2**	p.V15A	2	1	COSM1443673,COSM1443674	rs55827768		Missense_Mutation
**TAS2R31**	p.R295W	1	0	COSM3931508	rs199894662		Missense_Mutation
**TAS2R31**	p.Q46Q	1	0	COSM3746272		1	Silent
**TAS2R41**	p.S295S	1	0	COSM1086815	rs145885431		Silent
**TBC1D26**	p.Y99C	1	3	COSM3733460,COSM3733459	rs202131240		Missense_Mutation

**TBKBP1**	p.E100E	1	0					■													COSM3680494,COSM148291	rs78884849		Silent
**TBXAS1**	p.R85Q	1	2							■											COSM204919	rs372928270		Missense_Mutation
**TCERG1**	p.A215A	1	0					■													COSM3761017	rs112548582		Silent
**TCHH**	p.Q1492Q	2	0									■							■		COSM267557		1	Silent
**TCHH**	p.QQV1437fs	1	0													■					COSM1334068		1	Frame_Shift_Del
**TCHH**	p.L1043R	1	0								■										COSM528874		1	Missense_Mutation
**TCN2**	p.T329M	1	0		■																COSM184802	rs117458738		Missense_Mutation
**TCP11L1**	p.P321P	1	0			■															COSM3752335,COSM3752334	rs61889262		Silent
**TGM6**	p.V295M	3	7	■															■	■	COSM1025258	rs200341133		Missense_Mutation
**TGOLN2**	p.T80S	1	0														■				COSM1722795		1	Missense_Mutation
**THAP11**	p.Q122Q	1	0											■							COSM1563553	rs28647874		Silent
**THRAP3**	p.L474V	1	0		■																COSM3997498	rs141254021		Missense_Mutation
**THSD7A**	p.P56R	1	0	■																	COSM1235712	rs140474426		Missense_Mutation
**TJP1**	p.T991T	3	0				■										■	■			COSM2083996	rs373971232		Silent
**TLR9**	p.H104Y	1	2			■															COSM3760161	rs34038856		Missense_Mutation
**TMC5**	p.S285I	1	0							■											COSM3754743	rs72785308		Missense_Mutation
**TMEM245**	p.L121L	1	0																	■	COSM3699332	rs140147240		Silent
**TMEM247**	p.Q128E	1	4																	■	COSM442956	rs74318890		Missense_Mutation
**TMEM63B**	p.P274P	1	1							■											COSM84980	rs377733970		Silent
**TMPRSS3**	p.A138T	1	3			■															COSM3551159,COSM3551160,C	rs140614903		Missense_Mutation
**TNK1**	p.R507H	3	0	■															■	■	COSM270735	rs369367062		Missense_Mutation
**TOR1AIP1**	p.A441A	1	1				■														COSM4025640	rs548782601		Silent
**TOX4**	p.M319V	1	0							■											COSM258628		1	Missense_Mutation
**TRAF2**	p.T401T	1	0														■				COSM3216723,COSM3216722	rs189329794		Silent
**TRAK1**	p.TE630fs	2	0																■	■	COSM308433		1	Frame_Shift_Del
**TRIOBP**	p.P754L	1	5									■									COSM1294075		1	Missense_Mutation
**TRIP10**	p.P329P	3	0	■															■	■	COSM3836102,COSM3836101,C	rs75725110		Silent
**TRPC4**	p.T259T	3	0	■															■	■	COSM1639347,COSM1639346	rs115609911		Silent
**TRPC4AP**	p.S777C	1	2		■																COSM149164	rs117729108		Missense_Mutation
**TRPM2**	p.S709S	1	0				■														COSM1414383	rs149727028		Silent
**TRPM8**	p.I171I	1	0													■					COSM3579441,COSM109347	rs151205278		Silent
**TSKS**	p.EPEEKQK25	1	0						■												COSM999411	rs550916960,rs59626794		Frame_Shift_Del
**TSPEAR**	p.I383I	1	0			■															COSM1031295	rs587652857		Silent
**TTC30B**	p.Q119R	1	3								■										COSM249631	rs144172590		Missense_Mutation
**TTN**	p.R31089H	1	7							■											COSM1401819,COSM1401818,C	rs367993101		Missense_Mutation
**TTN**	p.R8294G	2	5														■	■			COSM3757808,COSM3757807	rs72648982		Missense_Mutation
**TTN**	p.S10148S	1	0					■													COSM1133604,COSM1645620	rs367901929		Silent
**TUBA3D**	p.R156W	1	6											■							COSM3364278	rs145612794		Missense_Mutation
**TUBB2B**	p.A248V	1	5												■						COSM3830204		1	Missense_Mutation
**TUBB8**	p.A313V	1	0		■																COSM226661	rs199826048		Missense_Mutation
**TUBB8**	p.S115S	2	0		■					■											COSM3752053	rs61839057		Silent
**TUBGCP6**	p.L787L	1	0			■															COSM1535712	rs541224216		Silent
**TXNDC2**	p.K73E	3	0									■					■		■		COSM417785,COSM417786		1	Missense_Mutation
**TXNDC2**	p.E133K	1	0														■				COSM3403698,COSM3403697		1	Missense_Mutation
**UBB**	p.E92E	1	0										■								COSM307160		1	Silent
**UBR7**	p.A7T	1	0																	■	COSM3754179	rs2286653		Missense_Mutation
**UGT1A9**	p.R103Q	1	0		■																COSM286278	rs555995846		Missense_Mutation
**UGT2B11**	p.D458H	1	5											■							COSM229477	rs144149579		Missense_Mutation
**UNC80**	p.T3225T	3	0	■															■	■	COSM149055	rs141603698		Silent
**UNC93A**	p.P106L	1	6				■														COSM3410786	rs202053224		Missense_Mutation
**VCX**	p.D30G	1	0														■				COSM4002335	rs6639945		Missense_Mutation
**VCX**	p.E161E	2	0									■	■								COSM301078		1	Silent
**VCX3B**	p.R50R	1	0					■													COSM4157037,COSM458143	rs201223234		Silent
**VCX3B**	p.E161E	1	0					■													COSM1288894	rs200893803		Silent
**VEZF1**	p.Q340fs	1	0							■											COSM1479777		1	Frame_Shift_Ins
**VEZF1**	p.Q338Q	1	0																	■	COSM3820052		1	Silent
**VHLL**	p.A15V	1	0							■											COSM4023246	rs374972165		Missense_Mutation

**VPS18**	p.R56H	1	0				■														COSM1129220	rs79533486		Missense_Mutation
**VTI1A**	p.Q144Q	1	0				■														COSM3751528	rs145533830		Silent
**WDR3**	p.G901G	1	0																	■	COSM4142237		1	Silent
**WDR36**	p.G352G	1	0													■					COSM177547	rs139303905		Silent
**WDR87**	p.VREEEEE23(	1	0								■										COSM439379		1	Frame_Shift_Del
**WIPI2**	p.P199P	2	0														■	■			COSM3762744	rs1134489		Silent
**WNT4**	p.V288M	2	2														■	■			COSM1232831	rs533186105		Missense_Mutation
**WWP1**	p.R764H	3	6	■															■	■	COSM1458474	rs527247142		Missense_Mutation
**ZDHHC11**	p.V228L	1	2							■											COSM3768884,COSM3768885	rs148433225		Missense_Mutation
**ZDHHC11**	p.T339K	2	0		■					■											COSM1735550,COSM1735549	rs2335585		Missense_Mutation
**ZFAND2B**	p.P71H	1	1			■															COSM3757958,COSM3757957	rs61750009		Missense_Mutation
**ZFHX3**	p.S1517L	1	2		■																COSM2153358	rs144091993		Missense_Mutation
**ZFP14**	p.Y452Y	1	0		■																COSM3756684	rs8110644		Silent
**ZFP14**	p.Y368Y	1	0											■							COSM1393064	rs373801795		Silent
**ZFP90**	p.E446K	1	2													■					COSM264835	rs199604310		Missense_Mutation
**ZMIZ2**	p.F730L	1	3																	■	COSM3923672		1	Missense_Mutation
**ZNF100**	p.E488E	1	0																■		COSM349538		1	Silent
**ZNF100**	p.V314E	2	0								■					■					COSM710866		1	Missense_Mutation
**ZNF107**	p.R488I	1	1														■				COSM1091219		1	Missense_Mutation
**ZNF107**	p.K359E	1	0														■				COSM351635		1	Missense_Mutation
**ZNF107**	p.A380G	1	0									■									COSM3396699	rs371108369		Missense_Mutation
**ZNF195**	p.S463S	1	0								■										COSM400443		1	Silent
**ZNF208**	p.G717E	3	4	■									■						■		COSM3531481,COSM3531480,COSM3531482		1	Missense_Mutation
**ZNF208**	p.K852E	1	2													■					COSM1304302,COSM1304303,C	rs536562250		Missense_Mutation
**ZNF208**	p.V773G	1	1						■												COSM1722979,COSM1722980,C	rs539995727		Missense_Mutation
**ZNF208**	p.E908K	1	0										■								COSM1471064,COSM1471065,COSM1471063		1	Missense_Mutation
**ZNF208**	p.G605G	1	0										■								COSM148554,COSM1293748,COSM1293749		1	Silent
**ZNF208**	p.R517K	1	0																■		COSM2747397		1	Missense_Mutation
**ZNF208**	p.T451K	2	0										■						■		COSM1293751,COSM1293750,COSM1293752		1	Missense_Mutation
**ZNF225**	p.T501T	2	0											■	■						COSM1525756		1	Silent
**ZNF251**	p.N202S	1	1							■											COSM1196749,COSM1196748	rs61732715		Missense_Mutation
**ZNF253**	p.R144I	1	2								■										COSM278268,COSM278269		1	Missense_Mutation
**ZNF254**	p.R458G	3	1										■				■		■		COSM1723380		1	Missense_Mutation
**ZNF267**	p.S711T	1	1																■		COSM3817917		1	Missense_Mutation
**ZNF273**	p.R391K	1	0																■		COSM345986		1	Missense_Mutation
**ZNF319**	p.Y278Y	1	0		■																COSM971934	rs201816162		Silent
**ZNF341**	p.E50D	2	2														■	■			COSM1025979	rs577699019		Missense_Mutation
**ZNF343**	p.T458T	1	0																■		COSM4097195		1	Silent
**ZNF429**	p.Y269S	2	0								■						■				COSM1680841		1	Missense_Mutation
**ZNF429**	p.I334I	1	0																■		COSM1559508		1	Silent
**ZNF429**	p.G337G	3	0										■			■			■		COSM1559509		1	Silent
**ZNF43**	p.K704E	1	0																■		COSM148549		1	Missense_Mutation
**ZNF43**	p.P307P	1	0										■								COSM1723391		1	Silent
**ZNF430**	p.R324K	1	0																■		COSM1271395		1	Missense_Mutation
**ZNF431**	p.R252I	1	3													■					COSM993654		1	Missense_Mutation
**ZNF536**	p.P988P	1	0			■															COSM4000645	rs142744560		Silent
**ZNF551**	p.C589C	1	0														■				COSM1293900		1	Silent
**ZNF567**	p.R550I	1	7													■					COSM286929	rs192986331		Missense_Mutation
**ZNF583**	p.N280I	2	5							■		■									COSM1190168		1	Missense_Mutation
**ZNF586**	p.R346C	1	2		■																COSM4082211	rs548136012		Missense_Mutation
**ZNF599**	p.T271M	2	3														■	■			COSM1197486	rs540997796		Missense_Mutation
**ZNF626**	p.R445I	1	1					■													COSM265098	rs4809075		Missense_Mutation
**ZNF675**	p.G346G	2	0										■				■				COSM1293770		1	Silent
**ZNF675**	p.E337E	3	0										■				■		■		COSM1293771		1	Silent
**ZNF676**	p.S518S	1	0												■						COSM565862		1	Silent
**ZNF676**	p.R504P	5	0	■		■		■	■										■		COSM1471062	rs202153135		Missense_Mutation
**ZNF676**	p.A444T	1	0										■								COSM1293753		1	Missense_Mutation

**ZNF678**	p.R356K	2	0													■			■		COSM1668535,COSM1668534		1	Missense_Mutation
**ZNF679**	p.Q275K	3	0				■				■	■									COSM1550331		1	Missense_Mutation
**ZNF681**	p.S505P	2	0												■				■		COSM347727,COSM347728		1	Missense_Mutation
**ZNF695**	p.D439E	2	0										■			■					COSM1271721		1	Missense_Mutation
**ZNF705B**	p.I90L	1	2							■											COSM1137908,COSM1137907	rs2740676		Missense_Mutation
**ZNF708**	p.L251P	2	1										■		■						COSM148547		1	Missense_Mutation
**ZNF708**	p.K417E	2	0								■								■		COSM1293741		1	Missense_Mutation
**ZNF708**	p.V384I	3	0										■				■		■		COSM1189973		1	Missense_Mutation
**ZNF709**	p.R464L	1	2								■										COSM287008		1	Missense_Mutation
**ZNF709**	p.G528G	4	0												■		■	■	■		COSM991295		1	Silent
**ZNF709**	p.H420Y	2	0				■						■								COSM3528755	rs200490873		Missense_Mutation
**ZNF714**	p.P395P	1	0																■		COSM347719,COSM347718		1	Silent
**ZNF714**	p.L423P	1	0													■					COSM341782,COSM341781		1	Missense_Mutation
**ZNF716**	p.R322K	2	0								■								■		COSM1723419,COSM150298		1	Missense_Mutation
**ZNF717**	p.R440H	1	0																	■	COSM4158474,COSM4158475		1	Missense_Mutation
**ZNF721**	p.T553A	1	2										■								COSM3917821,COSM3917820		1	Missense_Mutation
**ZNF721**	p.I531N	2	2							■			■								COSM3604039,COSM3604040	rs200851544		Missense_Mutation
**ZNF721**	p.E682E	1	0												■						COSM1294140,COSM1294141		1	Silent
**ZNF724P**	p.E391E	1	0																■		COSM1293761,COSM1293760		1	Silent
**ZNF737**	p.T280S	1	2																■		COSM3531250		1	Missense_Mutation
**ZNF765**	p.G351E	2	3								■				■						COSM3893323		1	Missense_Mutation
**ZNF765**	p.S340S	1	0																■		COSM1396098		1	Silent
**ZNF776**	p.I481I	1	0															■			COSM1305211,COSM1305210	rs563977196		Silent
**ZNF780A**	p.E555E	1	0										■								COSM1304580,COSM1304579		1	Silent
**ZNF813**	p.E78G	1	1				■														COSM3736205	rs140217321		Missense_Mutation
**ZNF813**	p.R366Q	4	0								■			■		■			■		COSM3693049		1	Missense_Mutation
**ZNF814**	p.E348D	4	1									■	■				■		■		COSM397041		1	Missense_Mutation
**ZNF814**	p.T788T	2	0						■					■							COSM3836001	rs534434666		Silent
**ZNF816**	p.A571G	1	1															■			COSM1190147		1	Missense_Mutation
**ZNF816**	p.Q385Q	1	0			■															COSM148726	rs76104029		Silent
**ZNF845**	p.M423T	2	0									■	■								COSM1305052		1	Missense_Mutation
**ZNF845**	p.T479T	3	0								■				■				■		COSM1000794		1	Silent
**ZNF845**	p.E856E	2	0										■						■		COSM3362995		1	Silent
**ZNF85**	p.K370E	1	2									■									COSM1611895		1	Missense_Mutation
**ZNF85**	p.E373G	1	1									■									COSM1611896		1	Missense_Mutation
**ZNF850**	p.G731C	4	0								■					■	■		■		COSM1480972		1	Missense_Mutation
**ZNF90**	p.A534E	2	0									■	■								COSM148544		1	Missense_Mutation
**ZNF91**	p.R1033Q	2	2	■															■		COSM3724347,COSM3724348	rs1821846		Missense_Mutation
**ZNF91**	p.T952N	1	2										■								COSM4076415,COSM4076416		1	Missense_Mutation
**ZNF91**	p.T1013K	2	1								■								■		COSM1189984		1	Missense_Mutation
**ZNF91**	p.K1051K	1	0										■								COSM1723436,COSM1723437	rs368262222		Silent
**ZNF92**	p.R201I	2	2									■					■				COSM1091292		1	Missense_Mutation
**ZNF92**	p.R313I	2	2									■							■		COSM288709		1	Missense_Mutation
**ZNF92**	p.E205G	2	0									■					■				COSM1723787		1	Missense_Mutation
**ZNF92**	p.F277S	3	0								■	■							■		COSM3396701		1	Missense_Mutation
**ZNF92**	p.T316T	2	0									■							■		COSM1330556		1	Silent
**ZNF92**	p.I419T	1	0										■								COSM1673198		1	Missense_Mutation
**ZNF98**	p.R426I	1	3										■								COSM993977,COSM993978		1	Missense_Mutation
**ZNF99**	p.E478Q	1	5							■											COSM1129915		1	Missense_Mutation
**ZNF99**	p.G375S	1	4										■								COSM3404045	rs200740712		Missense_Mutation
**ZNF99**	p.M553I	1	0														■				COSM2748507		1	Missense_Mutation
**ZNF99**	p.C374C	1	0								■										COSM4076402		1	Silent
**ZSCAN31**	p.H26H	1	0				■														COSM4160479	rs853677		Silent
**AC011298.1**	p.H6Q	1	0								■										COSM3766551			Missense_Mutation
**ACACA**	p.R183Q	1	7													■					COSM185746			Missense_Mutation
**ACOX2**	p.E516K	1	4		■																COSM420327			Missense_Mutation
**ACSM5**	p.I35I	1	0			■															COSM2129733			Silent

**ADAM29**	p.R696Q	1	1													■					COSM265251			Missense_Mutation
**ADHFE1**	p.R379Q	1	8			■															COSM194617			Missense_Mutation
**AGAP8**	p.N322D	1	3				■														COSM3675780			Missense_Mutation
**AGAP8**	p.E523E	3	0	■			■												■		COSM2145906			Silent
**AMER1**	p.P160S	1	1				■														COSM1123908,COSM1123907			Missense_Mutation
**AMER2**	p.T241M	1	2										■								COSM4046722,COSM4046723			Missense_Mutation
**AMPD3**	p.T130M	3	0	■															■	■	COSM1350545			Missense_Mutation
**ANK2**	p.S1872L	1	2														■				COSM1309636			Missense_Mutation
**ANKAR**	p.E1177K	2	4														■	■			COSM4089669,COSM4089668			Missense_Mutation
**ANKRD22**	p.R119Q	1	0													■					COSM225299			Missense_Mutation
**ANKRD26**	p.A1086T	1	2				■														COSM4013541,COSM4013540			Missense_Mutation
**ANKRD30A**	p.V254A	1	0														■				COSM1297228,COSM3670527			Missense_Mutation
**ANKRD36**	p.F129V	1	1				■														COSM477780,COSM477781			Missense_Mutation
**AOC2**	p.R597H	2	6														■	■			COSM1383472			Missense_Mutation
**APC**	p.R2204*	1	2													■					COSM1059633			Nonsense_Mutation
**APC**	p.S1189*	1	1													■					COSM25151			Nonsense_Mutation
**APPBP2**	p.R497*	1	2													■					COSM982210			Nonsense_Mutation
**ARAF**	p.P376P	1	0			■															COSM1121839			Silent
**ARAF**	p.I445I	1	0																	■	COSM2965918			Silent
**ARHGAP20**	p.S693S	1	0							■											COSM4017737,COSM4017738			Silent
**ARHGAP36**	p.R427C	1	5							■											COSM265332			Missense_Mutation
**ARID2**	p.Q1318*	1	1													■					COSM268708			Nonsense_Mutation
**ASH1L**	p.R2078C	1	6													■					COSM170344			Missense_Mutation
**C12orf10**	p.R22W	1	0					■													COSM318991			Missense_Mutation
**C1orf65**	p.L548L	2	0						■					■							COSM3484126			Silent
**C7orf65**	p.P16L	1	4																	■	COSM453138			Missense_Mutation
**CACNA1A**	p.R1626C	1	7		■																COSM438670,COSM438671,COSM1480612,COSM438669			Missense_Mutation
**CASP14**	p.F48F	3	0	■															■	■	COSM4074910			Silent
**CCDC15**	p.Y592F	4	1			■								■	■	■					COSM1188410,COSM1188409			Missense_Mutation
**CCDC74A**	p.G255A	1	2																	■	COSM1006598			Missense_Mutation
**CDH10**	p.D709D	1	0				■														COSM1178088			Silent
**CDH20**	p.E656K	1	4		■																COSM3891215			Missense_Mutation
**CEP104**	p.T921M	1	4							■											COSM908952			Missense_Mutation
**CFB**	p.G135S	1	4							■											COSM3921405			Missense_Mutation
**CIITA**	p.A479A	1	0					■													COSM3957104			Silent
**CLEC16A**	p.K75N	1	7													■					COSM1201338			Missense_Mutation
**CMTM5**	p.A63V	1	4		■																COSM1192177,COSM1192178			Missense_Mutation
**CNBD2**	p.L445L	1	0							■											COSM3545815			Silent
**CNTNAP5**	p.R1234*	1	1													■					COSM1006141			Nonsense_Mutation
**COL5A1**	p.D665D	1	0		■																COSM753242			Silent
**COL6A3**	p.E1807K	1	5													■					COSM3579609			Missense_Mutation
**COPS2**	p.R100W	1	6													■					COSM4055396			Missense_Mutation
**CPED1**	p.N182H	1	6													■					COSM274318			Missense_Mutation
**CRYBG3**	p.R397Q	3	1	■															■	■	COSM3824738			Missense_Mutation
**CTCFL**	p.E423K	1	4													■					COSM3423741,COSM192130			Missense_Mutation
**CTD-2207O23**	p.T266M	1	0			■															COSM243040			Missense_Mutation
**CWF19L2**	p.R704W	1	5				■														COSM4017524,COSM4017523			Missense_Mutation
**CYHR1**	p.A144fs	1	0		■																COSM1455657			Frame_Shift_Del
**DAB2**	p.S310L	1	4													■					COSM1437539			Missense_Mutation
**DCAF8L2**	p.E145E	1	0																	■	COSM1467346			Silent
**DCHS2**	p.L1731I	1	3													■					COSM274744			Missense_Mutation
**DDX56**	p.R157C	1	5			■															COSM1089969			Missense_Mutation
**DDX60L**	p.L1348L	1	0							■											COSM3825512			Silent
**DHX9**	p.R905*	1	2													■					COSM900728			Nonsense_Mutation
**DIRC2**	p.V408L	1	5															■			COSM3720429			Missense_Mutation
**DISC1**	p.S301F	1	3		■																COSM232163,COSM232162,COSM232161			Missense_Mutation
**DKKL1**	p.E116E	1	0				■														COSM191799			Silent

**DLG2**	p.R532Q	1	6								■										COSM1357419,COSM1357420,COSM1357421,COSM1357418	Missense_Mutation
**DMBT1**	p.Q288R	1	0														■				COSM1504795,COSM1504793,COSM1504794	Missense_Mutation
**DNAJC2**	p.R357*	1	2													■					COSM1083559,COSM1083558	Nonsense_Mutation
**DOCK3**	p.D1879D	3	0	■															■	■	COSM3408772,COSM3408771	Silent
**DPP10**	p.D727N	1	9							■											COSM350355,COSM350354	Missense_Mutation
**DYX1C1**	p.T303M	2	1														■	■			COSM1478208	Missense_Mutation
**DZIP3**	p.D1017D	1	0		■																COSM4112090	Silent
**EGFR**	NULLL	1	0														■				COSM1580369	Splice_Site
**EMR1**	p.S348T	1	3												■						COSM1711679	Missense_Mutation
**EP400**	p.T2224M	1	8				■														COSM937177	Missense_Mutation
**ERBB2**	p.S310F	1	7													■					COSM48358	Missense_Mutation
**ERBB2**	p.I767M	1	6								■										COSM51317	Missense_Mutation
**ERI2**	p.E270*	1	0													■					COSM968147	Nonsense_Mutation
**ERICH1**	p.D273D	1	0		■																COSM1100928	Silent
**F5**	p.H1215H	1	0															■			COSM4143010	Silent
**FAM155A**	p.Q79R	1	1								■										COSM3968526	Missense_Mutation
**FAM166A**	p.P135P	1	0							■											COSM1107092	Silent
**FGA**	p.R308Q	1	1									■									COSM480981,COSM3601172	Missense_Mutation
**FGD6**	p.K623N	1	6													■					COSM1677253	Missense_Mutation
**FHOD1**	p.E1006K	1	5													■					COSM3387536	Missense_Mutation
**FLG2**	p.Q1034Q	1	0		■																COSM146622	Silent
**FLG2**	p.S660S	3	0			■								■		■					COSM1293197	Silent
**FMO3**	p.I199T	1	7					■													COSM530573	Missense_Mutation
**FNDC1**	p.E259K	1	3													■					COSM275202	Missense_Mutation
**FOXD4**	p.D21N	1	3				■														COSM364818,COSM364819	Missense_Mutation
**FOXP1**	p.S599S	1	0				■														COSM1048338	Silent
**FRG2C**	p.S183S	1	0										■								COSM1753391	Silent
**FRY**	p.R1110Q	1	4													■					COSM946721	Missense_Mutation
**FZD4**	p.P355P	1	0													■					COSM4037250	Silent
**GCM2**	p.A174T	1	5													■					COSM483246	Missense_Mutation
**GIMAP4**	p.V120L	1	7								■										COSM1548702	Missense_Mutation
**GIPC2**	p.E115K	1	0													■					COSM168689	Missense_Mutation
**GNPDA2**	p.D206N	1	7													■					COSM1055631	Missense_Mutation
**GOLGA8I**	p.S445T	1	1								■										COSM2082065,COSM2082066	Missense_Mutation
**GPR52**	p.R329Q	1	3													■					COSM899869	Missense_Mutation
**GRIA1**	p.S904C	1	6													■					COSM3852995,COSM3852994	Missense_Mutation
**GRIK1**	p.R83I	1	5													■					COSM1029836,COSM1029837	Missense_Mutation
**HELQ**	p.I944I	1	0			■															COSM3606602	Silent
**HERC2**	p.R3906C	1	4													■					COSM960137	Missense_Mutation
**HERC6**	p.R644C	1	4													■					COSM281683	Missense_Mutation
**HIVEP2**	p.S800Y	1	7													■					COSM1074021	Missense_Mutation
**HMCN1**	p.R3978Q	1	5													■					COSM1689420	Missense_Mutation
**HSPA13**	p.F277F	1	0													■					COSM1029451	Silent
**ICA1L**	p.S243P	1	2													■					COSM209616	Missense_Mutation
**IFNA7**	p.R3Q	1	2			■															COSM3906572	Missense_Mutation
**IKZF4**	p.R108Q	2	8														■	■			COSM4043422,COSM4043423	Missense_Mutation
**IL33**	NULLL	1	0														■				COSM1181070	Splice_Site
**IL5RA**	p.R371R	1	1										■								COSM3592389	Silent
**IMPG2**	p.S839L	1	5													■					COSM1035756	Missense_Mutation
**INCENP**	p.R126H	1	7							■											COSM929557,COSM929556	Missense_Mutation
**INSL5**	p.L131F	1	8													■					COSM1503390	Missense_Mutation
**ITFG1**	p.R202*	1	1													■					COSM3421001	Nonsense_Mutation
**ITGA11**	p.R550*	1	1													■					COSM1210972	Nonsense_Mutation
**KCNH1**	p.L286R	1	6								■										COSM4027850	Missense_Mutation
**KCNN2**	p.F503F	1	0													■					COSM167031,COSM167030	Silent
**KDR**	p.R1229Q	1	4											■							COSM3604533	Missense_Mutation
**KIAA0753**	p.E278*	1	2													■					COSM275681	Nonsense_Mutation

**KIAA1586**	p.S509F	1	1							■											COSM1754895			Missense_Mutation
**KIAA1755**	p.F746F	1	0								■										COSM3840963			Silent
**KIF13A**	p.S1591L	1	1		■																COSM1076315,COSM1596156,COSM1596155			Missense_Mutation
**KIF21B**	p.P1246L	2	3														■	■			COSM1320589			Missense_Mutation
**KIR2DL1**	p.R71H	1	1				■														COSM440293			Missense_Mutation
**KLHDC3**	p.P283P	1	0				■														COSM1444614			Silent
**KMT2C**	p.R4549C	1	8													■					COSM245710,COSM245709			Missense_Mutation
**KRT1**	p.T481T	1	0				■														COSM940805			Silent
**KRTAP10-10**	p.T135A	1	0							■											COSM3681319			Missense_Mutation
**KRTAP10-12**	p.T96I	1	2			■															COSM1600097			Missense_Mutation
**KRTAP10-4**	p.S327C	1	1										■								COSM4134994			Missense_Mutation
**KRTAP4-12**	p.R87R	1	0											■							COSM293753			Silent
**KRTAP4-6**	p.C194C	1	0								■										COSM3517243			Silent
**LAD1**	p.F432F	1	0		■																COSM902185			Silent
**LGSN**	p.R482Q	1	2													■					COSM226164			Missense_Mutation
**LRP3**	p.R54C	1	7													■					COSM994687			Missense_Mutation
**LRPAP1**	p.V216I	3	0	■															■	■	COSM1213794			Missense_Mutation
**LRRC30**	p.I260I	1	0													■					COSM4073235			Silent
**LRRTM4**	p.E348K	1	4													■					COSM3583499,COSM3583498,COSM3583497			Missense_Mutation
**LSM11**	p.R180W	1	7													■					COSM177533			Missense_Mutation
**MAGEA12**	p.V161F	1	5							■											COSM1625648			Missense_Mutation
**MAGEC1**	p.S336C	1	5							■											COSM3232935			Missense_Mutation
**MAGEC1**	p.I340T	1	2							■											COSM3232937			Missense_Mutation
**MAGEC1**	p.F451S	1	1									■									COSM4154864			Missense_Mutation
**MAGEC1**	p.S380S	1	0											■							COSM3558667			Silent
**MAP2**	p.S482A	1	0															■			COSM4090708			Missense_Mutation
**MAPK15**	p.P102P	1	0																	■	COSM3747738			Silent
**MAS1**	p.R144H	2	7														■	■			COSM218865			Missense_Mutation
**MCM3AP**	p.R32Q	1	0													■					COSM1414606			Missense_Mutation
**MDFIC**	p.C227Y	1	7							■											COSM1673471			Missense_Mutation
**METTL2B**	p.C59S	2	0				■			■											COSM3662914			Missense_Mutation
**METTL3**	p.R508C	1	7										■								COSM3885838			Missense_Mutation
**MICALCL**	p.R393I	1	2													■					COSM1352145			Missense_Mutation
**MNDA**	p.F252F	1	0													■					COSM282839			Silent
**MSL2**	p.K104T	1	6													■					COSM204725			Missense_Mutation
**MUC16**	p.G13270G	1	0					■													COSM715546,COSM715545			Silent
**MUC16**	p.L2520L	1	0							■											COSM3668408,COSM3668409			Silent
**MUC17**	p.T2702M	1	4										■								COSM1083314			Missense_Mutation
**MUC17**	p.G2828S	1	3											■							COSM3703090			Missense_Mutation
**MUC17**	p.T676A	1	1														■				COSM3876553			Missense_Mutation
**MUC17**	p.N1741T	1	1						■												COSM4161656			Missense_Mutation
**MUC17**	p.T2183A	1	1									■									COSM1471960			Missense_Mutation
**MUC17**	p.N2194T	1	1									■									COSM1312520			Missense_Mutation
**MUC17**	p.L2703L	1	0										■								COSM1547580			Silent
**MUC7**	p.P82H	1	1				■														COSM3946528			Missense_Mutation
**MUC7**	p.P285P	1	0														■				COSM3826131			Silent
**MYOM2**	p.R1248Q	1	3													■					COSM276240			Missense_Mutation
**N4BP3**	p.A81A	1	0		■																COSM3854080			Silent
**NACA2**	p.P17P	1	0													■					COSM121281			Silent
**NAV1**	p.S1656S	1	0								■										COSM1639679			Silent
**NBPF6**	p.K41K	1	0						■												COSM3975809,COSM3975810			Silent
**NDUFA10**	p.A172V	1	9			■															COSM1669898			Missense_Mutation
**NEB**	p.R5783H	1	5		■																COSM1007558,COSM1590730,COSM1007556			Missense_Mutation
**NLRP14**	p.D968N	1	0													■					COSM136817			Missense_Mutation
**NLRP3**	p.R773W	1	4					■													COSM1690084			Missense_Mutation
**NMT2**	p.R54*	1	3				■														COSM276361			Nonsense_Mutation
**NOL4**	NULLL	1	0										■								COSM253038			Splice_Site

**NPIPB11**	p.P719R	2	0	■															■		COSM3679386,COSM3679385			Missense_Mutation
**NPIPB15**	p.P355T	1	3				■														COSM2690685,COSM2690686			Missense_Mutation
**NR3C2**	p.S965S	1	0								■										COSM302772			Silent
**NRXN3**	p.E307K	1	5													■					COSM196753			Missense_Mutation
**NUP205**	p.R1180Q	1	5													■					COSM2859296			Missense_Mutation
**OR10K2**	p.S310F	1	0													■					COSM232946			Missense_Mutation
**OR2B11**	p.S279Y	1	4													■					COSM3419027			Missense_Mutation
**OR2L5**	p.R265Q	1	0													■					COSM907155			Missense_Mutation
**OSBPL10**	p.E240K	1	3													■					COSM276558,COSM276559			Missense_Mutation
**OSBPL3**	p.H733Y	1	3									■									COSM1698435			Missense_Mutation
**PA2G4**	NULLL	3	0					■			■						■				COSM253050			Splice_Site
**PARK2**	p.F209L	1	6													■					COSM1219291			Missense_Mutation
**PARP15**	p.F228F	1	0													■					COSM3056464			Silent
**PCDH20**	p.P364S	2	2						■					■							COSM948273,COSM948272			Missense_Mutation
**PCDHA8**	p.S406L	1	5							■											COSM3373695,COSM3373694			Missense_Mutation
**PCDHB3**	p.A484T	1	6				■														COSM3851391			Missense_Mutation
**PCDHGA6**	p.G599V	1	6																	■	COSM1130863			Missense_Mutation
**PCDHGA6**	p.R747W	1	4													■					COSM262813,COSM1594842			Missense_Mutation
**PCDHGB6**	p.R714C	1	2																	■	COSM1063242			Missense_Mutation
**PCNX**	p.R1836*	1	2													■					COSM2136424			Nonsense_Mutation
**PDZD2**	p.R1581H	1	1			■															COSM1067277			Missense_Mutation
**PELP1**	p.L508L	1	0		■																COSM3680496,COSM3680495			Silent
**PITRM1**	p.R900*	1	2													■					COSM1347592			Nonsense_Mutation
**PLEKHG2**	p.E1048K	1	1							■											COSM3823033,COSM3823034,COSM3823032			Missense_Mutation
**PNKD**	p.I221M	3	7	■															■	■	COSM573570,COSM1649760			Missense_Mutation
**POM121C**	p.P694L	1	3					■													COSM1739378			Missense_Mutation
**POSTN**	p.R284Q	2	4						■					■							COSM1747432			Missense_Mutation
**PPFIBP2**	p.F686F	1	1													■					COSM932111			Silent
**PPM1L**	p.R315*	1	2													■					COSM207514,COSM207513			Nonsense_Mutation
**PPP1R13B**	p.R101Q	1	3		■																COSM284182			Missense_Mutation
**PPP2R2B**	p.F80F	1	0													■					COSM482290,COSM482291,COSM1137278,COSM1137277			Silent
**PRPF40A**	p.P14R	1	1				■														COSM1263161			Missense_Mutation
**RABGAP1**	p.R971W	1	4			■															COSM3903918,COSM3903919			Missense_Mutation
**RASSF9**	p.R68*	1	1		■																COSM263268,COSM263267			Nonsense_Mutation
**RBM46**	p.K258T	1	2													■					COSM4122981			Missense_Mutation
**RBMXL1**	p.H94R	1	1															■			COSM4144225			Missense_Mutation
**RGPD3**	p.L812R	1	5		■																COSM1004884,COSM1004883			Missense_Mutation
**RHBDL1**	p.V198M	3	4	■															■	■	COSM3402468			Missense_Mutation
**RNF145**	p.A683V	1	2														■				COSM3784754			Missense_Mutation
**RPL10L**	p.K156R	1	5															■			COSM357720			Missense_Mutation
**RPL21**	p.R32Q	1	1													■					COSM3417499			Missense_Mutation
**RPRD2**	p.R946C	1	5													■					COSM3473384,COSM3473385			Missense_Mutation
**RPS7**	p.E138K	1	7													■					COSM1020576			Missense_Mutation
**RPTN**	p.G458G	1	0															■			COSM3801863			Silent
**SCUBE2**	p.D59D	2	0														■	■			COSM4037418,COSM4037419			Silent
**SEL1L3**	p.R357*	1	2		■																COSM3917664,COSM3917663,COSM3917665			Nonsense_Mutation
**SEPP1**	p.R305Q	1	2													■					COSM1671625			Missense_Mutation
**SETD8**	p.V315I	1	2																	■	COSM1225388			Missense_Mutation
**SFRP4**	p.R232Q	1	0								■										COSM258002			Missense_Mutation
**SGSM1**	p.C473C	3	0	■															■	■	COSM3708180,COSM3708181			Silent
**SHOX2**	p.D37N	1	8													■					COSM1040471,COSM1040470			Missense_Mutation
**SLC18A2**	p.L462I	1	1													■					COSM172320			Missense_Mutation
**SLC30A8**	p.S125S	1	0							■											COSM1095341			Silent
**SLC37A2**	p.D341N	3	7	■															■	■	COSM169604,COSM1352355			Missense_Mutation
**SLC5A7**	p.I68I	1	0										■								COSM714870			Silent
**SLC9B1P1**	p.F134L	1	1																■		COSM4157068			Missense_Mutation
**SMARCAD1**	p.L324I	1	4													■					COSM285200			Missense_Mutation

**SMURF1**	p.E402K	1	5													■					COSM1453241			Missense_Mutation
**SNRPB2**	p.I220I	1	0			■															COSM3840485			Silent
**SOCS2**	p.R96Q	1	7													■					COSM277418			Missense_Mutation
**SOHLH1**	p.A185A	1	0					■													COSM3699527,COSM294842			Silent
**SPAST**	p.R581*	1	1		■																COSM169112			Nonsense_Mutation
**SPATA17**	p.S296S	1	0										■								COSM1266572			Silent
**SPATA31A6**	p.D624D	1	0			■															COSM1314849			Silent
**STAM2**	NULLL	2	0	■															■		COSM1735986			Splice_Site
**SYNE2**	p.S3374*	1	0							■											COSM699021			Nonsense_Mutation
**SYNJ2**	p.R116C	2	7														■	■			COSM3860020,COSM3860021			Missense_Mutation
**TAPT1**	NULLL	1	0					■													COSM1733487			Splice_Site
**TBC1D3G**	p.Y163Y	1	0							■											COSM2154776			Silent
**TCHH**	p.E1695E	2	0	■															■		COSM1491724			Silent
**TCHH**	p.W1016R	2	0										■					■			COSM230875			Missense_Mutation
**TEKT3**	p.R315Q	1	7			■															COSM231365			Missense_Mutation
**TEX15**	p.S2291Y	1	1													■					COSM1099145			Missense_Mutation
**TGFB1I1**	p.R67W	1	6													■					COSM4060498,COSM4060497			Missense_Mutation
**TGM3**	p.D690N	1	0													■					COSM285687			Missense_Mutation
**THAP11**	p.Q113Q	1	0												■						COSM4061991			Silent
**THY1**	p.H61H	1	1				■														COSM4018559,COSM4018560			Silent
**TNFRSF11A**	p.C34Y	1	6			■															COSM564120			Missense_Mutation
**TP53**	p.H193L	1	9									■									COSM99919,COSM99918,COSM99916,COSM3732881,COSM27441			Missense_Mutation
**TP63**	p.R594*	1	2													■					COSM3591234,COSM285877			Nonsense_Mutation
**TPM4**	p.S170Y	1	6													■					COSM181737,COSM181736			Missense_Mutation
**TSHZ3**	p.G876R	1	5		■																COSM1392507,COSM1392506			Missense_Mutation
**TTC6**	p.L245V	1	0																	■	COSM4148090			Missense_Mutation
**TTLL5**	p.R735Q	1	8													■					COSM1371183			Missense_Mutation
**TTN**	p.R17221C	1	7													■					COSM1645510,COSM1645511,COSM1645508,COSM1645512,COS			Missense_Mutation
**TTN**	p.F883F	1	0				■														COSM1403558,COSM1403559,COSM1403557,COSM1403555,COS			Silent
**TUBGCP6**	p.T1100T	1	0											■							COSM1158820			Silent
**TYR**	p.S27S	1	0							■											COSM429996			Silent
**USP17L10**	p.Q430H	1	1		■																COSM3683335			Missense_Mutation
**USP24**	p.R1367*	1	2													■					COSM278032,COSM278031			Nonsense_Mutation
**USP47**	p.F305F	1	0													■					COSM541102			Silent
**UTRN**	p.S3240F	1	8													■					COSM205666			Missense_Mutation
**VENTX**	p.G179G	1	0													■					COSM3790626			Silent
**VPS13B**	p.C1174Y	1	1				■														COSM1453479,COSM1453480			Missense_Mutation
**WHSC1**	p.T1150A	1	7													■					COSM3733388,COSM215940			Missense_Mutation
**YBEY**	NULLL	1	0			■															COSM309695			Splice_Site
**YTHDC2**	p.R911Q	1	4													■					COSM278175			Missense_Mutation
**ZCCHC6**	p.D196D	1	0			■															COSM3908643			Silent
**ZFP36**	p.S15S	1	0					■													COSM996284			Silent
**ZNF117**	p.S300T	2	0	■															■		COSM216071			Missense_Mutation
**ZNF136**	p.R464Q	1	1													■					COSM991202			Missense_Mutation
**ZNF189**	p.R285Q	1	3													■					COSM1103319			Missense_Mutation
**ZNF253**	p.V201I	1	0															■			COSM1271183,COSM1271184			Missense_Mutation
**ZNF257**	p.K429K	1	0																■		COSM1392123			Silent
**ZNF273**	p.R366I	3	1	■		■													■		COSM3396700			Missense_Mutation
**ZNF3**	p.H329Q	1	3									■									COSM1673352			Missense_Mutation
**ZNF347**	p.S556N	1	0												■						COSM1000715			Missense_Mutation
**ZNF382**	p.T458T	1	0								■										COSM566095			Silent
**ZNF429**	p.A489A	1	0												■						COSM1559523			Silent
**ZNF43**	p.K759E	2	0	■															■		COSM1293744			Missense_Mutation
**ZNF438**	p.N230S	1	1							■											COSM684507			Missense_Mutation
**ZNF479**	p.R435K	1	1						■												COSM388413			Missense_Mutation
**ZNF493**	p.T441A	2	0									■						■			COSM248585			Missense_Mutation
**ZNF506**	p.P364P	1	0									■									COSM148538			Silent

**ZNF614**	p.T438A	1	1											■							COSM567233			Missense_Mutation
**ZNF626**	p.S354Y	1	5										■								COSM3822452			Missense_Mutation
**ZNF678**	p.D156E	6	0	■						■					■	■	■		■		COSM3396148,COSM3396147			Missense_Mutation
**ZNF678**	p.Q322K	4	0	■						■		■							■		COSM3927624,COSM303650			Missense_Mutation
**ZNF714**	p.M360I	3	0	■								■							■		COSM394678,COSM394679			Missense_Mutation
**ZNF721**	p.K436E	1	0								■										COSM1665267,COSM1665266			Missense_Mutation
**ZNF727**	p.K394K	3	0	■								■							■		COSM1451757			Silent
**ZNF727**	p.S404T	3	0	■								■							■		COSM3833068			Missense_Mutation
**ZNF732**	p.T515A	2	1							■	■										COSM1665271			Missense_Mutation
**ZNF732**	p.T494I	1	0				■														COSM1289200			Missense_Mutation
**ZNF776**	p.R477Q	3	1											■			■	■			COSM193794,COSM1305209			Missense_Mutation
**ZNF776**	p.I453I	1	0							■											COSM1305208,COSM1305207			Silent
**ZNF804B**	p.R188*	1	1													■					COSM167453			Nonsense_Mutation
**ZNF814**	p.E297D	3	1						■			■		■							COSM4132682			Missense_Mutation
**ZNF845**	p.S452S	3	0	■													■		■		COSM475211			Silent
**ZNF85**	p.E373D	1	2									■									COSM1611897			Missense_Mutation
**ZNF85**	p.T480K	1	0					■													COSM1189966			Missense_Mutation
**ZNF878**	p.R153S	1	1														■				COSM1293730,COSM1293729,COSM1293728			Missense_Mutation
**ZNF91**	p.S541T	1	1									■									COSM1189989			Missense_Mutation
**ZNF91**	p.N534T	1	1														■				COSM1189990			Missense_Mutation
**ZNF92**	p.E150E	1	0									■									COSM1550314			Silent
**ZNF92**	p.T204T	2	0									■					■				COSM1723786			Silent
**ZNF99**	p.K771N	4	0	■							■	■							■		COSM1611986			Missense_Mutation
**ZNF99**	p.T635T	1	0										■								COSM1129916			Silent
**ZNF99**	p.G589G	2	0										■						■		COSM2748502			Silent
**37135**	p.E377K	1	7														■							Missense_Mutation
**39142**	p.F335V	1	3													■								Missense_Mutation
**39326**	p.DN270fs	2	0	■															■					Frame_Shift_Del
**AACS**	p.D82D	1	0		■																			Silent
**AARS**	p.N317I	3	7	■															■	■				Missense_Mutation
**ABCA1**	p.T947P	1	8			■																		Missense_Mutation
**ABCA12**	p.S74fs	1	0						■															Frame_Shift_Del
**ABCA13**	p.S1389C	3	4	■															■	■				Missense_Mutation
**ABCA13**	p.L3387L	1	0				■																	Silent
**ABCA5**	p.S923*	1	1						■															Nonsense_Mutation
**ABCB11**	p.L81fs	1	0							■														Frame_Shift_Ins
**ABCB8**	p.M372V	1	3					■																Missense_Mutation
**ABCC10**	p.V1218G	1	8						■															Missense_Mutation
**ABCC12**	p.N1256H	1	8													■								Missense_Mutation
**ABCC2**	p.L49L	1	0							■														Silent
**ABCC5**	p.V144M	1	6							■														Missense_Mutation
**ABCC9**	p.R281R	1	0													■								Silent
**ABCD1**	p.Y527D	1	7	■																				Missense_Mutation
**ABCF3**	p.M222L	1	3	■																				Missense_Mutation
**ABHD12B**	p.V329F	1	7			■																		Missense_Mutation
**ABHD12B**	p.E327D	1	1													■								Missense_Mutation
**ABHD17B**	p.S97L	1	6										■											Missense_Mutation
**ABI3BP**	p.G1088G	1	1																	■				Silent
**ABLIM1**	p.L492L	2	0														■	■						Silent
**AC136604.1**	p.C107R	1	1			■																		Missense_Mutation
**AC136604.1**	p.L106L	1	0			■																		Silent
**ACAD10**	p.R416Q	1	0				■																	Missense_Mutation
**ACAD11**	p.L479L	1	0				■																	Silent
**ACAD8**	p.R296Q	1	3		■																			Missense_Mutation
**ACAN**	p.G1543E	1	3												■									Missense_Mutation
**ACAN**	p.F1542V	2	2		■										■									Missense_Mutation
**ACAN**	p.F1542L	2	2		■										■									Missense_Mutation

**ACAT2**	p.P204S	2	6	Missense_Mutation
**ACAT2**	p.M373T	1	2	Missense_Mutation
**ACBD3**	p.A313V	1	2	Missense_Mutation
**ACCS**	p.V392F	3	5	Missense_Mutation
**ACIN1**	p.R849R	1	0	Silent
**ACOT11**	p.V298V	1	0	Silent
**ACOT8**	p.P189L	1	2	Missense_Mutation
**ACPP**	p.N119fs	3	0	Frame_Shift_Del
**ACPT**	p.T107T	1	0	Silent
**ACRC**	p.S209C	1	3	Missense_Mutation
**ACRC**	p.S367F	1	3	Missense_Mutation
**ACRC**	p.P179P	3	0	Silent
**ACRC**	p.D187E	1	0	Missense_Mutation
**ACRC**	p.V188A	1	0	Missense_Mutation
**ACSM3**	p.G206A	3	0	Missense_Mutation
**ACSM3**	p.G206G	1	0	Silent
**ACSM4**	p.E70K	3	1	Missense_Mutation
**ACSM5**	p.S396C	1	0	Missense_Mutation
**ACSS3**	p.I252M	1	2	Missense_Mutation
**ACTA1**	p.E255A	1	8	Missense_Mutation
**ACVR2A**	p.G107G	1	0	Silent
**ADAL**	p.LCL232fs	1	0	Frame_Shift_Del
**ADAM18**	p.F70V	1	6	Missense_Mutation
**ADAM22**	p.G770G	1	1	Silent
**ADAM30**	p.E663K	1	1	Missense_Mutation
**ADAMTS1**	p.E389Q	1	7	Missense_Mutation
**ADAMTS15**	p.F127V	1	6	Missense_Mutation
**ADAMTS16**	p.G886E	1	7	Missense_Mutation
**ADAMTS20**	p.T154T	1	0	Silent
**ADAMTS4**	p.G539D	1	8	Missense_Mutation
**ADAMTS8**	p.T847T	2	0	Silent
**ADAMTS8**	p.LP21fs	1	0	Frame_Shift_Ins
**ADAMTS9**	p.T175I	3	7	Missense_Mutation
**ADAMTSL2**	p.D153A	1	1	Missense_Mutation
**ADAMTSL2**	p.V606M	1	0	Missense_Mutation
**ADAMTSL4**	p.T733K	1	5	Missense_Mutation
**ADAR**	p.Y1173H	1	3	Missense_Mutation
**ADAR**	p.S264G	1	0	Missense_Mutation
**ADARB2**	p.L589L	1	0	Silent
**ADCK1**	p.P320P	2	0	Silent
**ADCK2**	p.K136N	1	1	Missense_Mutation
**ADCK2**	p.S83R	2	0	Missense_Mutation
**ADCY8**	p.T220T	1	0	Silent
**ADD3**	p.P553P	1	0	Silent
**ADH1A**	p.K340N	1	7	Missense_Mutation
**ADHFE1**	p.G240D	1	8	Missense_Mutation
**ADPRHL1**	p.R15G	1	4	Missense_Mutation
**ADRBK1**	p.N434N	1	0	Silent
**ADRM1**	p.N303N	1	0	Silent
**ADSS**	p.V269V	3	0	Silent
**AFAP1L1**	p.R580H	1	5	Missense_Mutation
**AFAP1L1**	p.A692A	2	0	Silent
**AFAP1L2**	p.L347V	1	6	Missense_Mutation
**AFF4**	p.N612N	1	0	Silent
**AGAP1**	p.A723S	1	1	Missense_Mutation
**AGAP3**	p.R533R	1	1	Silent
**AGER**	p.G397G	3	0	Silent

**AGPAT5**	p.S314F	3	5	■															■	■				Missense_Mutation
**AHNAK**	p.L1931M	1	3			■																		Missense_Mutation
**AIM1**	p.A883G	2	1							■						■								Missense_Mutation
**AIRE**	p.G228G	1	0					■																Silent
**AK5**	p.F343L	1	1													■								Missense_Mutation
**AKAP11**	p.I1117V	1	5			■																		Missense_Mutation
**AKAP11**	p.T230I	1	1							■														Missense_Mutation
**AKAP11**	p.A1421T	1	1													■								Missense_Mutation
**AKAP8**	p.E580K	1	3					■																Missense_Mutation
**AKAP9**	p.Q2227Q	1	0							■														Silent
**AKAP9**	p.3012_3013SS	1	0								■													In_Frame_Del
**AKT3**	p.R464G	1	2										■											Missense_Mutation
**AL358813.2**	p.S54S	1	0				■																	Silent
**AL359195.1**	p.S116T	1	0						■															Missense_Mutation
**AL445989.1**	p.C54Y	1	2														■							Missense_Mutation
**ALCAM**	p.C392C	1	1					■																Silent
**ALDH1B1**	p.T53P	1	7				■																	Missense_Mutation
**ALDH1L1**	p.S708Y	1	7													■								Missense_Mutation
**ALDH1L2**	p.A26S	1	8					■																Missense_Mutation
**ALDOC**	p.V306M	1	5				■																	Splice_Site
**ALK**	p.L520*	1	0										■											Nonsense_Mutation
**ALKBH1**	p.P357T	3	0	■															■	■				Missense_Mutation
**ALKBH8**	p.S368P	1	3				■																	Missense_Mutation
**ALMS1**	p.P1691T	1	0					■																Missense_Mutation
**ALPK2**	p.D1104H	1	4										■											Missense_Mutation
**ALPK2**	p.L1338F	1	0								■													Missense_Mutation
**ALS2CR11**	p.R197C	1	1							■														Missense_Mutation
**ALX3**	p.R211H	1	8													■								Missense_Mutation
**AMBP**	p.S18R	1	0										■											Missense_Mutation
**AMDHD2**	p.L233L	3	0	■															■	■				Silent
**AMER2**	p.H507Q	1	5													■								Missense_Mutation
**AMOT**	p.L263L	3	0	■															■	■				Silent
**AMOTL2**	p.A470A	1	0							■														Silent
**AMPD1**	p.N318Y	1	7		■																			Missense_Mutation
**AMY2A**	p.V80G	1	9													■								Missense_Mutation
**ANAPC1**	p.N1532H	1	5													■								Missense_Mutation
**ANAPC2**	p.F600F	3	0	■															■	■				Silent
**ANK2**	p.D3490N	1	7										■											Missense_Mutation
**ANK2**	p.D2325H	1	1													■								Missense_Mutation
**ANK3**	p.K2511Q	1	2		■																			Missense_Mutation
**ANK3**	p.P1644P	1	0			■																		Silent
**ANKAR**	p.L791R	2	4														■	■						Missense_Mutation
**ANKFN1**	p.R25R	1	0			■																		Silent
**ANKK1**	p.A468T	1	8							■														Missense_Mutation
**ANKLE2**	NULLL	1	0																	■				De_novo_Start_OutOfFrame
**ANKRD10**	p.L348L	2	0														■	■						Silent
**ANKRD11**	p.D714Y	1	6													■								Missense_Mutation
**ANKRD11**	p.S1977A	2	0				■		■															Missense_Mutation
**ANKRD20A4**	p.A360V	2	1	■															■					Missense_Mutation
**ANKRD20A4**	p.K471K	2	0														■	■						Silent
**ANKRD26**	p.-755fs	1	0													■								Frame_Shift_Ins
**ANKRD30A**	p.S274P	2	0									■				■								Missense_Mutation
**ANKRD30A**	p.K890K	1	0													■								Silent
**ANKRD30B**	p.L5L	1	0																	■				Silent
**ANKRD30BL**	p.P239P	1	0					■																Silent
**ANKRD30BL**	p.A70A	1	0													■								Silent
**ANKRD32**	p.L702L	1	0							■														Silent

**ANKRD36**	p.KP889fs	1	0			■																		Frame_Shift_Del
**ANKRD36C**	p.D1639N	1	1				■																	Missense_Mutation
**ANKRD53**	p.E189K	1	4													■								Missense_Mutation
**ANKRD61**	p.T237M	1	4				■																	Missense_Mutation
**ANKUB1**	p.L62L	3	0	■															■	■				Silent
**ANTXR2**	p.L246L	1	0													■								Silent
**ANXA9**	p.I167M	1	3					■																Missense_Mutation
**AOC1**	p.V260M	1	1				■																	Missense_Mutation
**AP1B1**	p.K30K	1	0							■														Silent
**AP4E1**	p.V609L	1	0				■																	Missense_Mutation
**AP5B1**	p.L807R	1	2	■																				Missense_Mutation
**APC2**	p.L537R	1	7		■																			Missense_Mutation
**APOB**	p.D2664E	1	6					■																Missense_Mutation
**APOB**	p.G1529D	1	6			■																		Missense_Mutation
**APOB**	p.Q2274Q	1	0										■											Silent
**APOBEC3D**	p.C332R	1	0			■																		Missense_Mutation
**APOBEC3F**	p.E282D	1	1							■														Missense_Mutation
**APOBEC4**	p.E259Q	1	0							■														Missense_Mutation
**APOH**	p.D236G	1	0										■											Missense_Mutation
**APP**	p.K315K	2	1														■	■						Silent
**APPL1**	p.S374C	1	6		■																			Missense_Mutation
**AQP10**	p.P249P	2	0	■															■					Silent
**AQP7**	p.M80I	1	3												■									Missense_Mutation
**ARAP2**	p.G250R	2	6														■	■						Missense_Mutation
**ARFGAP1**	p.A307A	3	0	■															■	■				Silent
**ARFIP2**	p.R86W	1	6							■														Missense_Mutation
**ARFIP2**	p.S41S	1	0													■								Silent
**ARHGAP11B**	p.A118A	1	0				■																	Silent
**ARHGAP15**	p.Q226H	1	1													■								Missense_Mutation
**ARHGAP17**	p.P810A	3	7		■						■			■										Missense_Mutation
**ARHGAP17**	p.P810R	1	6											■										Missense_Mutation
**ARHGAP21**	p.H550R	1	3					■																Missense_Mutation
**ARHGAP21**	p.R194H	1	2		■																			Missense_Mutation
**ARHGAP22**	p.M303I	2	7						■					■										Missense_Mutation
**ARHGAP23**	p.G1061G	1	1							■														Silent
**ARHGAP36**	NULLL	2	0						■					■										Splice_Site
**ARHGAP5**	p.R398G	1	3										■											Missense_Mutation
**ARHGAP6**	p.E351fs	1	0													■								Frame_Shift_Del
**ARHGEF15**	p.Q447E	2	0														■	■						Missense_Mutation
**ARHGEF26**	NULLL	1	1													■								Splice_Site
**ARHGEF38**	p.R591H	1	6				■																	Missense_Mutation
**ARHGEF4**	p.R191R	1	0							■														Silent
**ARHGEF4**	p.A435A	2	0														■	■						Silent
**ARID1B**	p.R1926R	1	0								■													Silent
**ARID2**	p.L1776fs	1	0													■								Frame_Shift_Ins
**ARID3B**	p.K359R	1	5							■														Missense_Mutation
**ARID3C**	p.P263P	1	0							■														Silent
**ARID4A**	p.K1136N	1	3													■								Missense_Mutation
**ARID5B**	p.D242V	1	6							■														Missense_Mutation
**ARL14EP**	p.S126T	3	0	■															■	■				Missense_Mutation
**ARL6IP1**	p.M46T	1	5							■														Missense_Mutation
**ARMC2**	p.E206G	2	2														■	■						Missense_Mutation
**ARMCX1**	p.G146G	1	0					■																Silent
**ARNT**	p.S672S	1	0					■																Silent
**ARSG**	p.G49A	2	5	■															■					Missense_Mutation
**ARSH**	p.G142R	1	7			■																		Missense_Mutation
**ARSI**	p.A490G	1	3										■											Missense_Mutation

**ARV1**	p.I158V	1	4				■																	Missense_Mutation
**ARVCF**	p.D532N	1	5					■																Missense_Mutation
**ASAP1**	p.N83T	1	1													■								Missense_Mutation
**ASAP2**	p.N714T	1	1			■																		Missense_Mutation
**ASB10**	p.R357H	1	3		■																			Missense_Mutation
**ASB5**	p.G253G	1	0													■								Silent
**ASCC3**	p.F672V	1	9													■								Missense_Mutation
**ASH1L**	p.F316C	1	6													■								Missense_Mutation
**ASH1L**	p.V1861V	3	0	■															■	■				Silent
**ASH1L**	p.K1056K	1	0								■													Silent
**ASIC1**	p.Q225P	1	6									■												Missense_Mutation
**ASIC3**	p.K426K	2	1														■	■						Silent
**ASIC3**	p.P521A	3	0	■						■									■					Missense_Mutation
**ASIC4**	p.A143V	1	3					■																Missense_Mutation
**ASPG**	p.G290D	1	7								■													Missense_Mutation
**ASPG**	p.S570*	1	1							■														Nonsense_Mutation
**ASPM**	p.K610E	1	0					■																Missense_Mutation
**ASPSCR1**	p.D602D	1	0					■																Silent
**ASRGL1**	p.E142Q	1	7													■								Missense_Mutation
**ASS1**	p.Y133Y	1	0				■																	Silent
**ASTL**	p.I147I	1	0							■														Silent
**ASZ1**	p.E180D	1	2		■																			Missense_Mutation
**ATAD2**	p.L637I	1	5			■																		Missense_Mutation
**ATAD2B**	p.Q1116E	1	3		■																			Missense_Mutation
**ATAD5**	p.G1068R	1	8		■																			Missense_Mutation
**ATE1**	p.R212R	1	1							■														Silent
**ATG16L2**	p.P59P	1	0								■													Silent
**ATG2A**	p.R50Q	1	4									■												Missense_Mutation
**ATL1**	p.E411Q	1	3									■												Missense_Mutation
**ATM**	p.W579*	1	2															■						Nonsense_Mutation
**ATM**	p.N2826H	1	2													■								Missense_Mutation
**ATP10B**	p.D689N	3	3	■															■	■				Missense_Mutation
**ATP11A**	p.S306N	1	6		■																			Missense_Mutation
**ATP11C**	p.Q745Q	1	0					■																Silent
**ATP13A1**	p.D788A	1	6												■									Missense_Mutation
**ATP13A3**	p.V96A	1	1													■								Missense_Mutation
**ATP2A3**	p.E82E	1	0					■																Silent
**ATP2B4**	p.K252T	1	2													■								Missense_Mutation
**ATP5SL**	p.R168C	3	7	■															■	■				Missense_Mutation
**ATP6V0A1**	p.L549L	1	0				■																	Silent
**ATP6V1B1**	p.L348F	1	8			■																		Missense_Mutation
**ATP8A2**	p.T365T	1	0														■							Silent
**ATP8A2**	p.A685A	3	0	■															■	■				Silent
**ATPAF1**	p.T197T	1	0					■																Silent
**ATRX**	p.Y2152H	2	6						■					■										Missense_Mutation
**ATXN3**	p.G306fs	1	0																	■				Frame_Shift_Ins
**ATXN3**	p.G306fs	1	0					■																Frame_Shift_Ins
**AUH**	p.K105N	1	5													■								Missense_Mutation
**AUTS2**	p.V944L	1	1												■									Missense_Mutation
**AVIL**	p.N158T	1	7			■																		Missense_Mutation
**AVP**	p.L14L	3	0	■															■	■				Silent
**AXL**	p.M623L	1	7			■																		Missense_Mutation
**AXL**	p.K624*	1	2			■																		Nonsense_Mutation
**B3GNT8**	p.S267S	2	0						■						■									Silent
**B3GNTL1**	p.R205Q	3	0	■															■	■				Missense_Mutation
**B4GALT2**	p.T359T	1	0			■																		Silent
**BAALC**	p.G50V	1	3																	■				Missense_Mutation

**BAG5**	p.I130V	1	0													■								Missense_Mutation
**BAI3**	p.C567R	1	6													■								Missense_Mutation
**BAI3**	p.Y818C	1	3													■								Missense_Mutation
**BAI3**	p.S211L	1	1		■																			Missense_Mutation
**BAZ1B**	p.EE1271fs	1	0				■																	Frame_Shift_Del
**BAZ1B**	p.E530E	1	0										■											Silent
**BBOX1**	p.S84N	1	8														■							Missense_Mutation
**BBS7**	p.V525D	1	4		■																			Missense_Mutation
**BBS7**	p.V525I	1	3		■																			Missense_Mutation
**BBS7**	p.E636*	2	2														■	■						Nonsense_Mutation
**BBS7**	p.C527fs	1	0		■																			Frame_Shift_Del
**BBS7**	p.V525V	1	0		■																			Silent
**BCAS3**	p.K890fs	1	0								■													Splice_Site
**BCAT1**	p.G302V	1	7							■														Splice_Site
**BCL11A**	p.I243I	1	0					■																Silent
**BCL7B**	p.S200A	1	0				■																	Missense_Mutation
**BCL9**	p.G1136S	1	3		■																			Missense_Mutation
**BCL9**	p.N577S	2	0														■	■						Missense_Mutation
**BCO2**	p.Y364C	1	6									■												Missense_Mutation
**BCOR**	p.L332L	3	0	■															■	■				Silent
**BCORL1**	p.S575A	2	0	■															■					Missense_Mutation
**BCORL1**	p.P578L	3	0	■															■	■				Missense_Mutation
**BDP1**	p.K1065N	1	3		■																			Missense_Mutation
**BDP1**	p.I1098V	2	2	■															■					Missense_Mutation
**BDP1**	p.N983D	1	1										■											Missense_Mutation
**BDP1**	p.L1051P	1	1		■																			Missense_Mutation
**BDP1**	p.L1057V	1	1		■																			Missense_Mutation
**BDP1**	p.G1058D	1	1		■																			Missense_Mutation
**BDP1**	p.D1070E	1	1		■																			Missense_Mutation
**BDP1**	p.F1072S	3	1		■							■	■											Missense_Mutation
**BDP1**	p.G1075E	3	1		■								■				■							Missense_Mutation
**BDP1**	p.G1050G	1	0		■																			Silent
**BDP1**	p.T1062T	1	0		■																			Silent
**BDP1**	p.A1083A	1	0		■																			Silent
**BEND3**	p.S262T	2	1														■	■						Missense_Mutation
**BEST3**	p.E374K	1	1				■																	Missense_Mutation
**BET1**	p.A83V	3	1	■															■	■				Missense_Mutation
**BET1**	p.L80F	1	0														■							Missense_Mutation
**BET1**	p.L80*	1	0														■							Nonsense_Mutation
**BFSP1**	NULLL	1	1													■								Splice_Site
**BFSP2**	p.V212I	1	2				■																	Missense_Mutation
**BHMT**	p.R16G	1	7				■																	Missense_Mutation
**BHMT**	p.F320V	1	3															■						Missense_Mutation
**BIRC6**	p.D950G	1	6													■								Missense_Mutation
**BIRC6**	p.V1544G	1	3		■																			Missense_Mutation
**BIRC6**	p.S1941del	1	0		■																			In_Frame_Del
**BLM**	p.T162I	1	1		■																			Missense_Mutation
**BLNK**	p.K35T	1	6													■								Missense_Mutation
**BLVRA**	p.P165P	1	0			■																		Silent
**BMP1**	p.Q730P	1	2						■															Missense_Mutation
**BMPR2**	p.V364M	2	5														■	■						Missense_Mutation
**BMPR2**	p.F127F	1	0							■														Silent
**BNIP1**	p.E105E	1	0			■																		Silent
**BPNT1**	p.S4N	1	1										■											Missense_Mutation
**BPTF**	p.D1225N	1	3													■								Missense_Mutation
**BRCA2**	p.Q1089H	1	5													■								Missense_Mutation
**BRD1**	p.K470E	1	4													■								Missense_Mutation

**BRD1**	p.A904A	1	0														■							Silent
**BRD7**	p.V322L	1	4										■											Missense_Mutation
**BRDT**	p.E771K	1	4				■																	Missense_Mutation
**BRINP1**	p.G569R	1	6							■														Missense_Mutation
**BROX**	p.A372A	1	0					■																Silent
**BRPF3**	p.R563W	1	7															■						Missense_Mutation
**BRPF3**	p.E13A	1	1										■											Missense_Mutation
**BRSK1**	p.T129P	1	6						■															Missense_Mutation
**BRSK2**	p.K152N	1	5										■											Missense_Mutation
**BRWD3**	p.R307H	1	7													■								Missense_Mutation
**BSN**	p.R1120C	1	7				■																	Missense_Mutation
**BSND**	p.P75P	1	0		■																			Silent
**BSPH1**	p.E76K	1	2													■								Missense_Mutation
**BTNL3**	p.A36G	1	6				■																	Missense_Mutation
**BUB1B**	p.A1016S	1	2									■												Missense_Mutation
**BZRAP1**	p.E392D	1	5							■														Missense_Mutation
**BZRAP1**	p.S1684S	3	0	■															■	■				Silent
**BZW1**	p.K399N	3	6	■															■	■				Missense_Mutation
**C10orf53**	p.E45D	1	3													■								Missense_Mutation
**C10orf82**	p.Q101H	1	0										■											Missense_Mutation
**C11orf53**	p.S79A	1	3	■																				Missense_Mutation
**C11orf57**	p.S125*	1	2									■												Nonsense_Mutation
**C11orf74**	p.E35*	1	3													■								Nonsense_Mutation
**C12orf42**	NULLL	1	1					■																De_novo_Start_OutOfFrame
**C12orf55**	p.Y495C	2	8														■	■						Missense_Mutation
**C12orf60**	p.D244Y	1	2															■						Missense_Mutation
**C14orf180**	p.V79I	1	0				■																	Missense_Mutation
**C15orf27**	p.P290Q	1	3		■																			Missense_Mutation
**C16orf46**	p.Q138H	3	0	■															■	■				Missense_Mutation
**C16orf71**	p.S254A	1	0										■											Missense_Mutation
**C17orf100**	p.S140I	1	1					■																Missense_Mutation
**C17orf47**	p.T213A	1	1							■														Missense_Mutation
**C17orf66**	p.Y108C	1	3															■						Missense_Mutation
**C17orf85**	p.E219Q	1	4							■														Missense_Mutation
**C18orf25**	p.D248A	1	6										■											Missense_Mutation
**C18orf63**	p.P275S	1	6													■								Missense_Mutation
**C19orf47**	p.D243A	1	5						■															Missense_Mutation
**C1orf110**	p.K66T	1	7													■								Missense_Mutation
**C1orf131**	p.G18G	1	0							■														Silent
**C1orf143**	p.M7V	1	0								■													Missense_Mutation
**C1orf173**	p.A147S	1	1													■								Missense_Mutation
**C1orf51**	p.V292G	1	5				■																	Missense_Mutation
**C1orf63**	NULLL	1	0								■													Splice_Site
**C1orf68**	p.Y81C	2	1	■															■					Missense_Mutation
**C1orf68**	p.T83T	2	0	■															■					Silent
**C1QB**	p.L19P	3	1	■															■	■				Missense_Mutation
**C1QL3**	p.Y140D	1	4	■																				Missense_Mutation
**C1QTNF2**	p.E17G	1	2										■											Missense_Mutation
**C1QTNF7**	p.G128E	1	8													■								Missense_Mutation
**C2CD2**	p.P348S	1	4														■							Missense_Mutation
**C2orf16**	p.H1662L	1	1			■																		Missense_Mutation
**C2orf16**	p.S1650S	1	0								■													Silent
**C2orf16**	p.S1730S	1	0									■												Silent
**C2orf16**	p.S1754S	1	0					■																Silent
**C2orf53**	p.F24C	1	2							■														Missense_Mutation
**C2orf57**	p.G130D	1	0			■																		Missense_Mutation
**C2orf71**	p.S660G	1	3			■																		Missense_Mutation

**C3AR1**	p.N19D	1	0							■														Missense_Mutation
**C3orf17**	p.E372Q	2	6														■	■						Missense_Mutation
**C3orf17**	p.K253N	1	4													■								Missense_Mutation
**C3orf17**	p.E520D	1	2													■								Missense_Mutation
**C3orf20**	p.R651H	1	3					■																Missense_Mutation
**C3orf62**	p.194_195IE>K	1	0			■																		In_Frame_Del
**C4A**	p.A1205A	1	0							■														Silent
**C4A**	p.V1207A	1	0							■														Missense_Mutation
**C4orf50**	p.E653K	1	0					■																Missense_Mutation
**C5AR1**	p.D37D	3	0	■															■	■				Silent
**C5orf30**	p.T65T	1	0													■								Silent
**C5orf34**	p.E514Q	1	0									■												Missense_Mutation
**C5orf42**	p.DI1874fs	2	1	■															■					Frame_Shift_Del
**C5orf42**	p.M107I	1	1													■								Missense_Mutation
**C5orf51**	p.A70D	1	6														■							Splice_Site
**C6orf10**	p.E393G	2	3	■															■					Missense_Mutation
**C6orf10**	p.R392S	1	0							■														Missense_Mutation
**C6orf10**	p.Q388Q	1	0						■															Silent
**C6orf10**	p.K383K	1	0														■							Silent
**C6orf10**	p.S382P	4	0	■					■								■		■					Missense_Mutation
**C6orf15**	p.G270C	1	4								■													Missense_Mutation
**C6ORF50**	p.F54I	3	2	■															■	■				Missense_Mutation
**C7**	p.S507Y	3	8	■															■	■				Missense_Mutation
**C7orf26**	p.S362A	1	1			■																		Missense_Mutation
**C8orf31**	p.H94N	1	3							■														Missense_Mutation
**C9**	p.V361A	3	7	■															■	■				Missense_Mutation
**C9orf129**	p.S32R	1	4							■														Missense_Mutation
**C9orf139**	p.D99N	1	1					■																Missense_Mutation
**C9orf152**	p.C180C	1	0			■																		Silent
**C9orf9**	p.P213S	1	5					■																Missense_Mutation
**CA10**	p.D139G	1	6									■												Missense_Mutation
**CA11**	p.V321V	1	0																	■				Splice_Site
**CA7**	p.H47H	1	0							■														Silent
**CAAP1**	p.K214K	3	0	■															■	■				Silent
**CABIN1**	p.L1649F	1	4				■																	Missense_Mutation
**CABLES1**	p.E621D	1	4							■														Missense_Mutation
**CACHD1**	p.L861L	1	0							■														Silent
**CACNA1A**	p.A1596G	1	4								■													Missense_Mutation
**CACNA1C**	p.E1507K	1	8													■								Missense_Mutation
**CACNA1C**	p.N1866N	3	0	■															■	■				Silent
**CACNA1C**	p.S2136S	1	0			■																		Silent
**CACNA1G**	p.P2222P	1	0						■															Silent
**CACNA1I**	p.F1664F	1	0					■																Silent
**CACNA2D1**	p.A220D	2	8	■															■					Splice_Site
**CACNA2D1**	NULLL	1	0											■										Splice_Site
**CACNB1**	p.G578R	1	3					■																Missense_Mutation
**CACNB3**	p.E45E	1	0			■																		Silent
**CACTIN**	p.A360A	1	0						■															Silent
**CADM3**	p.L48R	1	7					■																Missense_Mutation
**CALD1**	p.G3G	1	0	■																				Silent
**CAMSAP2**	p.L126L	1	0						■															Silent
**CAND1**	p.V79M	1	6													■								Missense_Mutation
**CAND1**	p.K801*	1	2															■						Nonsense_Mutation
**CANT1**	p.Q97Q	1	0													■								Silent
**CAP1**	p.P303H	2	5						■					■										Missense_Mutation
**CAPN11**	p.A689E	1	6							■														Missense_Mutation
**CAPN12**	p.K183K	3	0	■															■	■				Silent

**CAPN13**	p.R129W	2	6	Missense_Mutation
**CAPN13**	p.T227I	2	4	Missense_Mutation
**CAPN14**	p.G314R	1	7	Missense_Mutation
**CAPN9**	p.E316del	3	0	In_Frame_Del
**CAPS**	p.A89T	3	0	Missense_Mutation
**CASC4**	p.E401G	1	5	Missense_Mutation
**CASD1**	p.E588G	1	5	Missense_Mutation
**CASP2**	p.Y284Y	1	0	Silent
**CASP6**	p.G203A	1	3	Missense_Mutation
**CASZ1**	p.P1342A	1	1	Missense_Mutation
**CASZ1**	p.P1103P	1	0	Silent
**CATSPERB**	p.R116G	1	4	Missense_Mutation
**CATSPERB**	p.S715*	1	2	Nonsense_Mutation
**CBLB**	p.L464L	1	0	Silent
**CBLN1**	p.G110E	1	8	Missense_Mutation
**CBR4**	p.S111C	1	4	Missense_Mutation
**CBWD3**	p.A141T	2	7	Missense_Mutation
**CCDC103**	p.P157P	1	0	Silent
**CCDC103**	p.G165G	1	0	Silent
**CCDC11**	p.C395G	2	1	Missense_Mutation
**CCDC110**	p.E541G	1	0	Missense_Mutation
**CCDC13**	p.D642Y	1	4	Missense_Mutation
**CCDC136**	p.V501G	1	6	Missense_Mutation
**CCDC14**	p.S690S	1	0	Silent
**CCDC142**	p.C291Y	1	1	Missense_Mutation
**CCDC144NL**	p.Y192*	1	1	Nonsense_Mutation
**CCDC15**	p.R606L	1	3	Missense_Mutation
**CCDC15**	p.L510F	1	2	Missense_Mutation
**CCDC15**	p.I579K	1	1	Missense_Mutation
**CCDC15**	p.N478N	1	0	Silent
**CCDC15**	p.Q491Q	2	0	Silent
**CCDC155**	p.-530fs	1	0	Frame_Shift_Ins
**CCDC157**	p.E57*	1	3	Nonsense_Mutation
**CCDC170**	p.S262I	1	3	Missense_Mutation
**CCDC173**	p.A372G	1	2	Missense_Mutation
**CCDC173**	p.E363E	1	0	Silent
**CCDC18**	p.E391Q	1	5	Missense_Mutation
**CCDC33**	p.G259V	1	6	Missense_Mutation
**CCDC34**	p.H101Y	1	0	Missense_Mutation
**CCDC6**	p.M449L	1	0	Missense_Mutation
**CCDC63**	p.T350K	1	5	Missense_Mutation
**CCDC66**	p.S868L	1	2	Missense_Mutation
**CCDC66**	p.H423D	1	0	Missense_Mutation
**CCDC73**	p.G557G	1	0	Silent
**CCDC74A**	p.A256P	1	1	Missense_Mutation
**CCDC74A**	p.G255G	3	0	Silent
**CCDC74B**	p.N236N	1	0	Silent
**CCDC8**	p.E350D	1	0	Missense_Mutation
**CCDC8**	p.N334S	1	0	Missense_Mutation
**CCDC8**	p.A329G	1	0	Missense_Mutation
**CCDC88A**	p.T475T	1	0	Silent
**CCDC88C**	p.N1466D	1	1	Missense_Mutation
**CCHCR1**	p.E659K	1	1	Missense_Mutation
**CCKBR**	p.Y419Y	1	0	Silent
**CCKBR**	p.S442S	3	0	Silent
**CCM2**	p.S218C	1	7	Missense_Mutation
**CCNA1**	p.P123T	2	0	Missense_Mutation

**CCNB1**	p.M201I	1	7		■																			Missense_Mutation
**CCNB3**	p.R240Q	1	0													■								Missense_Mutation
**CCNE2**	p.I71F	1	3											■										Missense_Mutation
**CCNJL**	p.G337D	3	1	■															■	■				Missense_Mutation
**CCNL2**	p.T251M	1	4							■														Missense_Mutation
**CCNY**	p.A219A	1	0				■																	Silent
**CCPG1**	p.F552L	1	1				■																	Missense_Mutation
**CCRL2**	p.S261Y	3	5	■															■	■				Missense_Mutation
**CCRL2**	p.E193K	2	3						■					■										Missense_Mutation
**CCS**	p.N142N	1	0																	■				Silent
**CCT7**	p.R539H	2	5														■	■						Missense_Mutation
**CD163L1**	p.R827H	3	1	■															■	■				Missense_Mutation
**CD1C**	p.A135D	1	5				■																	Missense_Mutation
**CD200R1**	p.L232P	1	0							■														Missense_Mutation
**CD209**	p.K131N	1	3			■																		Missense_Mutation
**CD209**	p.E126D	1	3									■												Missense_Mutation
**CD209**	p.E126K	1	3									■												Missense_Mutation
**CD209**	p.R129L	2	2			■				■														Missense_Mutation
**CD209**	p.K119M	1	2								■													Missense_Mutation
**CD209**	p.S141S	1	0									■												Silent
**CD209**	p.L137L	1	0									■												Silent
**CD209**	p.L130L	1	0									■												Silent
**CD209**	p.L120L	1	0										■											Silent
**CD2BP2**	p.P325A	2	1														■	■						Missense_Mutation
**CD33**	p.V69V	1	0			■																		Silent
**CD34**	p.S28Y	1	1					■																Missense_Mutation
**CD7**	p.G24R	1	5					■																Missense_Mutation
**CD97**	p.P155P	1	0					■																Silent
**CDAN1**	p.P954L	1	4		■																			Missense_Mutation
**CDC37**	p.E184K	1	7					■																Missense_Mutation
**CDC5L**	p.L251P	1	4							■														Missense_Mutation
**CDCA7**	p.G105S	2	1														■	■						Missense_Mutation
**CDCP1**	p.G744R	1	5		■																			Missense_Mutation
**CDCP1**	p.R173S	1	0																	■				Missense_Mutation
**CDCP1**	p.V42V	1	0			■																		Silent
**CDH22**	p.V65G	2	6	■															■					Missense_Mutation
**CDH26**	p.G747G	1	0			■																		Silent
**CDH6**	p.G663D	1	8													■								Missense_Mutation
**CDK10**	p.E82E	1	0			■																		Silent
**CDK11B**	p.V164V	1	0														■							Silent
**CDK11B**	p.S80S	1	0							■														Silent
**CDKL4**	p.N89N	3	0	■															■	■				Silent
**CDKN1B**	p.K192I	1	3										■											Missense_Mutation
**CDKN1B**	p.K192N	1	3										■											Missense_Mutation
**CDKN1B**	p.Q193*	1	2										■											Nonsense_Mutation
**CDON**	p.P472A	1	3							■														Missense_Mutation
**CDSN**	p.N275K	1	2				■																	Missense_Mutation
**CEACAM4**	p.H242P	1	0						■															Missense_Mutation
**CEBPB**	p.S237R	1	0							■														Missense_Mutation
**CELA2B**	p.D114D	1	0			■																		Silent
**CELSR1**	p.R926R	1	1				■																	Silent
**CELSR1**	p.S2809S	1	0					■																Silent
**CELSR2**	p.G2243G	1	0	■																				Silent
**CELSR3**	p.A2239A	1	0								■													Silent
**CENPC**	p.R943T	1	2										■											Missense_Mutation
**CENPF**	p.Q1610*	1	1							■														Nonsense_Mutation
**CENPJ**	p.A259A	1	0			■																		Silent

**CEP120**	p.N747S	1	0													■								Missense_Mutation
**CEP135**	p.SA548fs	1	1			■																		Frame_Shift_Del
**CEP152**	p.E500*	1	1													■								Nonsense_Mutation
**CEP170**	p.Q250K	1	3													■								Missense_Mutation
**CEP170**	p.Y963*	1	2										■											Nonsense_Mutation
**CEP170**	p.A803T	1	1							■														Missense_Mutation
**CEP170B**	p.E775A	1	2						■															Missense_Mutation
**CEP41**	p.K158N	1	5				■																	Missense_Mutation
**CEP85L**	p.T548K	3	2	■															■	■				Missense_Mutation
**CEP95**	p.F104C	1	8													■								Missense_Mutation
**CEP97**	p.S122N	2	1														■	■						Missense_Mutation
**CERK**	p.S300R	1	7				■																	Missense_Mutation
**CES5A**	p.P158S	1	7							■														Missense_Mutation
**CETP**	p.V290V	1	0							■														Silent
**CFC1**	p.S14T	1	1								■													Missense_Mutation
**CFTR**	p.A455V	2	7		■					■														Missense_Mutation
**CGN**	p.E9A	2	4									■	■											Missense_Mutation
**CGN**	p.D674V	3	2	■															■	■				Missense_Mutation
**CGREF1**	p.E249D	2	1										■				■							Missense_Mutation
**CHAD**	p.T248T	1	0							■														Silent
**CHD1**	p.D1609V	1	4														■							Missense_Mutation
**CHD3**	p.L652Q	1	7									■												Missense_Mutation
**CHD3**	p.L1522L	1	0				■																	Silent
**CHD6**	p.1_2insG	1	0		■																			In_Frame_Ins
**CHD7**	p.A778G	1	4							■														Missense_Mutation
**CHD8**	p.V1810M	1	9							■														Missense_Mutation
**CHD8**	p.E928*	1	3													■								Nonsense_Mutation
**CHD9**	p.I1986T	1	8								■													Missense_Mutation
**CHD9**	p.T2480M	1	6			■																		Missense_Mutation
**CHD9**	p.P1817R	1	3			■																		Missense_Mutation
**CHEK2**	p.S471S	1	0							■														Silent
**CHKB**	p.Y136D	1	7																■					Missense_Mutation
**CHML**	p.T170T	1	0		■																			Silent
**CHPF2**	p.F219V	1	3				■																	Missense_Mutation
**CHPF2**	p.-455fs	3	0	■															■	■				Frame_Shift_Ins
**CHRM4**	p.K147*	1	1													■								Nonsense_Mutation
**CHRNA7**	p.I76V	1	2		■																			Missense_Mutation
**CHST12**	p.P195P	1	0		■																			Silent
**CHST2**	p.N400S	1	1		■																			Missense_Mutation
**CHST6**	p.H335R	1	1								■													Missense_Mutation
**CHST8**	p.V147V	1	0							■														Silent
**CIC**	p.H973P	1	6						■															Missense_Mutation
**CIRH1A**	p.K591R	3	2	■															■	■				Missense_Mutation
**CISH**	p.T198P	1	0													■								Missense_Mutation
**CIT**	p.P401P	1	1											■										Silent
**CITED4**	p.P117R	1	3																	■				Missense_Mutation
**CIZ1**	p.Q422R	1	2														■							Missense_Mutation
**CIZ1**	p.G387R	1	2								■													Missense_Mutation
**CIZ1**	p.G387A	1	1								■													Missense_Mutation
**CKAP2**	p.R316T	2	3						■					■										Missense_Mutation
**CKAP2**	p.N235S	3	0	■															■	■				Missense_Mutation
**CKAP2L**	p.S251R	1	0		■																			Missense_Mutation
**CLASRP**	p.E103E	1	0														■							Silent
**CLCN7**	p.A259T	2	7														■	■						Missense_Mutation
**CLCNKA**	p.V294G	1	3						■															Missense_Mutation
**CLDND1**	p.S26P	1	5													■								Missense_Mutation
**CLEC2B**	p.V12I	3	0	■															■	■				Missense_Mutation

**CLEC2D**	p.A54A	3	0	Silent
**CLEC4A**	p.Y7F	1	5	Missense_Mutation
**CLEC4C**	p.S107R	1	5	Missense_Mutation
**CLEC4M**	p.E134D	1	3	Missense_Mutation
**CLEC4M**	p.L126L	1	0	Silent
**CLEC4M**	p.T140T	1	0	Silent
**CLMN**	p.S811A	1	1	Missense_Mutation
**CLP1**	p.V190V	1	0	Silent
**CLPS**	p.Y76H	1	6	Missense_Mutation
**CLSPN**	p.S1096R	1	4	Missense_Mutation
**CLTA**	p.E82Q	1	5	Missense_Mutation
**CLTC**	p.K760N	1	7	Missense_Mutation
**CLTC**	p.Q1497*	1	2	Nonsense_Mutation
**CLUAP1**	p.S314S	1	0	Silent
**CMAS**	p.S387N	3	4	Missense_Mutation
**CMIP**	p.F307L	1	6	Missense_Mutation
**CMYA5**	p.S3978L	1	7	Missense_Mutation
**CMYA5**	p.I3282T	3	5	Missense_Mutation
**CMYA5**	p.I798V	1	2	Missense_Mutation
**CNDP2**	p.Y208Y	3	0	Silent
**CNNM1**	p.D846A	1	4	Missense_Mutation
**CNOT6L**	p.K33Q	1	4	Missense_Mutation
**CNPPD1**	p.T361T	1	0	Silent
**CNR2**	p.V231L	1	0	Missense_Mutation
**CNTFR**	p.G39V	1	7	Missense_Mutation
**CNTLN**	p.Q745H	1	0	Missense_Mutation
**CNTN4**	p.R272G	1	4	Missense_Mutation
**CNTNAP1**	p.A384P	1	3	Missense_Mutation
**CNTNAP3B**	NULLL	1	1	Splice_Site
**CNTNAP3B**	p.Q894*	1	1	Nonsense_Mutation
**CNTRL**	p.Q1095*	1	1	Nonsense_Mutation
**CNTROB**	p.L68V	1	1	Missense_Mutation
**COA6**	p.S24S	2	0	Silent
**COBL**	p.Q1239Q	1	0	Silent
**COG1**	p.L411R	1	5	Missense_Mutation
**COG1**	p.L412V	2	0	Missense_Mutation
**COL11A1**	p.G943G	1	0	Silent
**COL11A1**	p.F154F	1	0	Silent
**COL12A1**	p.P1020A	1	7	Missense_Mutation
**COL16A1**	p.V569L	1	1	Missense_Mutation
**COL17A1**	p.K496R	1	1	Missense_Mutation
**COL4A2**	p.M999K	1	2	Missense_Mutation
**COL4A3**	p.R1609*	1	2	Nonsense_Mutation
**COL6A3**	p.K2926N	1	1	Missense_Mutation
**COL6A5**	p.G25G	1	0	Silent
**COL7A1**	p.P855A	1	3	Missense_Mutation
**COL7A1**	p.A2810P	1	2	Missense_Mutation
**COL9A1**	p.G320V	1	8	Missense_Mutation
**COLCA1**	p.*125Y	1	0	Nonstop_Mutation
**COPS2**	p.N264K	1	7	Missense_Mutation
**COPS2**	p.S178L	1	6	Missense_Mutation
**COQ2**	p.L127V	1	7	Missense_Mutation
**CORIN**	p.H366H	2	0	Silent
**CP**	p.T1043P	1	6	Missense_Mutation
**CPEB4**	p.L165L	1	0	Silent
**CPNE4**	p.E383K	2	3	Missense_Mutation
**CPPED1**	p.D274E	1	6	Missense_Mutation

**CPSF7**	p.D441D	1	0					■																Silent
**CPT1B**	p.A398A	3	0	■															■	■				Silent
**CR1L**	p.V5L	1	1									■												Missense_Mutation
**CR2**	p.K421fs	3	0	■															■	■				Frame_Shift_Ins
**CRB1**	p.I280V	1	2			■																		Missense_Mutation
**CRISPLD1**	p.V171V	1	0													■								Silent
**CRNKL1**	p.S138W	1	2							■														Missense_Mutation
**CRYBB2**	p.F201C	1	7									■												Missense_Mutation
**CSE1L**	p.T520A	1	5		■																			Missense_Mutation
**CSF1R**	p.A220T	1	3							■														Missense_Mutation
**CSF2**	p.T95S	1	0					■																Missense_Mutation
**CSGALNACT**	p.R521H	1	4													■								Missense_Mutation
**CSMD1**	p.T551N	1	5															■						Missense_Mutation
**CSMD3**	p.R3025P	1	8										■											Missense_Mutation
**CSMD3**	p.S635N	1	1							■														Missense_Mutation
**CSPP1**	p.I1037I	1	0													■								Silent
**CSRP2**	p.I215M	1	6													■								Missense_Mutation
**CST11**	p.E4A	1	0							■														Missense_Mutation
**CTD-2600O9.**	p.R76W	1	4	■																				Missense_Mutation
**CTNNA3**	p.M1L	1	4		■																			Start_Codon_SNP
**CTNND1**	p.L866V	1	6													■								Missense_Mutation
**CUL4A**	p.M412I	1	1									■												Missense_Mutation
**CUL5**	p.Q39K	1	2			■																		Missense_Mutation
**CWC22**	p.E695A	3	2	■															■	■				Missense_Mutation
**CWF19L2**	p.E183D	1	4													■								Missense_Mutation
**CWF19L2**	p.R46H	1	4			■																		Missense_Mutation
**CXorf21**	p.V46fs	2	0						■					■										Frame_Shift_Del
**CXorf38**	p.L182L	1	0					■																Silent
**CYFIP1**	p.N99K	1	7							■														Missense_Mutation
**CYFIP1**	p.V14M	1	6									■												Missense_Mutation
**CYLC1**	p.D376D	1	0									■												Silent
**CYP11B2**	p.L410V	1	1				■																	Missense_Mutation
**CYP17A1**	p.L217V	1	0					■																Missense_Mutation
**CYP19A1**	p.T338S	3	1	■															■	■				Missense_Mutation
**CYP1A2**	p.P61L	1	7				■																	Missense_Mutation
**CYP1A2**	p.Y112C	2	7														■	■						Missense_Mutation
**CYP2A7**	p.L298H	1	5									■												Missense_Mutation
**CYP2J2**	p.R321L	2	4														■	■						Missense_Mutation
**CYP2W1**	p.P324L	1	8																	■				Missense_Mutation
**CYP4F22**	p.R283W	1	6					■																Missense_Mutation
**CYP4V2**	p.S227N	1	2		■																			Missense_Mutation
**DAAM2**	p.E424E	1	0													■								Silent
**DAB2IP**	p.L232L	1	0		■																			Silent
**DACT1**	p.Q411R	1	4				■																	Missense_Mutation
**DAGLA**	p.R197R	1	0													■								Silent
**DAGLB**	p.L274L	3	0	■															■	■				Silent
**DAP3**	p.H394D	1	1		■																			Missense_Mutation
**DARS**	NULLL	1	0													■								Splice_Site
**DBT**	p.R301H	1	6								■													Missense_Mutation
**DCAF16**	p.R154Q	1	2													■								Missense_Mutation
**DCAF4L1**	p.N172N	2	0														■	■						Silent
**DCDC1**	p.Q863Q	1	0							■														Silent
**DCDC2**	p.N24T	1	8			■																		Missense_Mutation
**DCHS2**	p.N2797D	1	3							■														Missense_Mutation
**DCLK2**	p.I424I	1	0				■																	Silent
**DCLK3**	p.P412P	1	0								■													Silent
**DCLRE1B**	p.P125S	1	6		■																			Missense_Mutation

**DCP2**	p.L355I	1	2				Missense_Mutation
**DDB1**	p.E779E	1	0				Silent
**DDI1**	p.M279T	1	2				Missense_Mutation
**DDX20**	p.R89G	1	1				Missense_Mutation
**DDX21**	p.K135N	1	3				Missense_Mutation
**DDX46**	p.E132Q	1	3				Missense_Mutation
**DDX5**	p.P151S	1	8				Missense_Mutation
**DDX52**	NULLL	2	0				Splice_Site
**DDX60L**	p.S146L	1	2				Missense_Mutation
**DENND1A**	p.PQ967fs	1	1				Frame_Shift_Del
**DENND4B**	p.P864S	1	5				Missense_Mutation
**DENND4C**	p.I629F	1	7				Missense_Mutation
**DENND5A**	p.F750C	1	7				Missense_Mutation
**DEPDC1**	p.R141*	1	1				Nonsense_Mutation
**DEPDC7**	p.L321L	1	0				Silent
**DFFA**	p.R307W	1	5				Missense_Mutation
**DFNA5**	p.L439L	1	0				Silent
**DFNB31**	p.H470H	1	0				Silent
**DGCR6**	p.D138Y	1	7				Missense_Mutation
**DGKD**	p.L1144L	1	0				Silent
**DGKD**	p.V1212V	1	0				Silent
**DGKG**	p.F58F	1	0				Silent
**DHRS3**	p.T80M	1	3				Missense_Mutation
**DHTKD1**	p.H828D	1	0				Missense_Mutation
**DHX37**	p.E54D	1	2				Missense_Mutation
**DHX9**	p.G1151V	1	4				Missense_Mutation
**DIAPH3**	p.L1001M	3	5				Missense_Mutation
**DIAPH3**	p.M1V	1	5				Start_Codon_SNP
**DIAPH3**	p.R1089P	1	2				Missense_Mutation
**DICER1**	p.T1696T	1	1				Silent
**DICER1**	p.E1797E	1	0				Silent
**DIDO1**	p.A1307P	1	1				Missense_Mutation
**DIDO1**	p.P1920P	1	0				Silent
**DIDO1**	p.P1357P	3	0				Silent
**DIP2B**	p.D1123H	1	5				Missense_Mutation
**DIS3**	p.S88F	1	2				Missense_Mutation
**DISP1**	p.G1166G	1	0				Silent
**DISP2**	p.F291I	1	5				Missense_Mutation
**DKKL1**	p.I33V	2	3				Missense_Mutation
**DLC1**	p.S592A	2	1				Missense_Mutation
**DLC1**	p.S751S	2	0				Silent
**DLEC1**	p.I1405I	1	0				Silent
**DLG2**	p.D229H	1	4				Missense_Mutation
**DLG2**	p.D229D	1	0				Silent
**DMBT1**	p.S130I	1	3				Missense_Mutation
**DMBT1**	p.S130R	1	2				Missense_Mutation
**DMBT1**	p.T1153I	1	1				Missense_Mutation
**DMBT1**	p.I117V	1	0				Missense_Mutation
**DMD**	p.P2261S	1	4				Missense_Mutation
**DMGDH**	p.V388G	3	7				Missense_Mutation
**DMRT1**	p.V39V	1	0				Silent
**DMWD**	p.T410I	1	0				Missense_Mutation
**DMXL1**	NULLL	3	1				Splice_Site
**DNAAF1**	p.E345D	1	0				Missense_Mutation
**DNAAF1**	p.V455A	1	0				Missense_Mutation
**DNAH11**	p.Y292C	1	5				Missense_Mutation
**DNAH11**	p.E1384K	1	5				Missense_Mutation

**DNAH11**	p.V1170A	1	3													■								Missense_Mutation
**DNAH11**	p.C3297C	1	1				■																	Silent
**DNAH11**	p.L1370L	1	0													■								Silent
**DNAH11**	p.K2249N	1	0				■																	Missense_Mutation
**DNAH17**	p.M548T	1	3							■														Missense_Mutation
**DNAH17**	p.I4416M	1	0					■																Missense_Mutation
**DNAH17**	p.L2016L	2	0						■					■										Silent
**DNAH2**	p.R442C	1	4														■							Missense_Mutation
**DNAH6**	p.Q93H	1	2													■								Missense_Mutation
**DNAH8**	p.Q1183R	3	6	■															■	■				Missense_Mutation
**DNAH9**	p.A4020V	1	6													■								Missense_Mutation
**DNAH9**	p.L3456fs	2	0														■	■						Frame_Shift_Del
**DNAI2**	p.P19P	2	0	■															■					Silent
**DNAJA2**	p.K226R	1	2													■								Missense_Mutation
**DNAJA2**	p.R165*	1	2													■								Nonsense_Mutation
**DNAJC16**	p.N334K	1	0				■																	Missense_Mutation
**DNAJC18**	NULLL	1	0							■														Splice_Site
**DNER**	p.Y350Y	1	0		■																			Silent
**DNHD1**	p.H1608Y	1	4									■												Missense_Mutation
**DNM3**	p.K636T	1	1													■								Missense_Mutation
**DNM3**	p.L285L	1	0		■																			Silent
**DNMT1**	p.K341K	3	0	■															■	■				Silent
**DNMT3B**	p.G16R	3	1	■															■	■				Missense_Mutation
**DOCK10**	p.P1014A	2	7						■					■										Missense_Mutation
**DOCK10**	p.Y1560Y	1	0													■								Silent
**DOCK10**	p.L676L	1	0													■								Silent
**DOCK5**	p.A546V	2	2														■	■						Missense_Mutation
**DOCK7**	p.F2034L	3	7	■															■	■				Missense_Mutation
**DOCK9**	p.Y833S	1	4							■														Missense_Mutation
**DOK4**	p.E206E	1	0			■																		Silent
**DPCR1**	p.E1019G	1	2							■														Missense_Mutation
**DPCR1**	p.V1060E	1	2							■														Missense_Mutation
**DPCR1**	p.K1068R	1	0										■											Missense_Mutation
**DPH6**	p.V227L	1	3				■																	Missense_Mutation
**DPP10**	p.Y160C	1	6							■														Missense_Mutation
**DROSHA**	p.Y269N	3	4	■															■	■				Missense_Mutation
**DSC2**	p.S740*	1	2													■								Nonsense_Mutation
**DSC3**	p.A692T	1	6													■								Missense_Mutation
**DSCAML1**	p.H440D	1	1					■																Missense_Mutation
**DSCAML1**	p.P149P	1	0		■																			Silent
**DSE**	p.T191T	1	0		■																			Silent
**DSP**	p.R1302L	1	4			■																		Missense_Mutation
**DST**	p.D6712G	1	7		■																			Missense_Mutation
**DST**	p.Q5505K	2	5			■		■																Missense_Mutation
**DST**	p.Y2023C	1	0							■														Missense_Mutation
**DTX1**	p.R224R	1	0																	■				Silent
**DTX4**	p.M265I	1	0															■						Missense_Mutation
**DUOX1**	p.L89R	1	4	■																				Missense_Mutation
**DUOX2**	p.D786A	1	4									■												Missense_Mutation
**DUSP21**	p.R77S	1	3			■																		Missense_Mutation
**DVL1**	p.S235F	1	7					■																Missense_Mutation
**DVL1**	p.L18L	1	0											■										Silent
**DVL2**	p.L117S	1	4									■												Missense_Mutation
**DYNC2H1**	p.E770K	1	1				■																	Missense_Mutation
**DYNC2H1**	p.K890K	1	0													■								Silent
**DZIP1**	p.Q597E	1	0							■														Missense_Mutation
**DZIP3**	p.R660K	1	0							■														Missense_Mutation

**EBF1**	p.S473T	1	5																	■				Missense_Mutation
**EBF4**	NULLL	1	1			■																		Splice_Site
**EBLN2**	p.F77S	3	5	■															■	■				Missense_Mutation
**ECEL1**	p.N767D	1	8							■														Missense_Mutation
**ECHDC1**	p.V232V	1	0							■														Silent
**ECI2**	p.R13R	1	0	■																				Silent
**EDAR**	p.N109Y	1	7			■																		Missense_Mutation
**EDAR**	p.L444V	1	6									■												Missense_Mutation
**EDC4**	p.L719R	1	1						■															Missense_Mutation
**EDDM3B**	p.V52A	3	1	■															■	■				Missense_Mutation
**EDEM1**	p.I253V	1	2														■							Missense_Mutation
**EDEM3**	p.N383S	2	2														■	■						Missense_Mutation
**EDNRA**	p.F250L	1	7						■															Missense_Mutation
**EEF1A1**	p.D35H	1	7													■								Missense_Mutation
**EEF1A2**	p.I368M	1	6							■														Missense_Mutation
**EEF2K**	p.R331R	1	0							■														Silent
**EFCAB5**	p.E1315Q	1	6										■											Missense_Mutation
**EFCAB7**	p.D418G	1	8		■																			Missense_Mutation
**EFEMP2**	p.Y319D	2	8	■															■					Missense_Mutation
**EFEMP2**	p.G385G	1	0					■																Silent
**EGFLAM**	p.T320T	1	0			■																		Silent
**EGFR**	NULLL	1	1														■							Splice_Site
**EGFR**	p.I1005V	1	1				■																	Missense_Mutation
**EGFR**	p.*1211*	1	0														■							Silent
**EHBP1L1**	p.G970V	1	3		■																			Missense_Mutation
**EHBP1L1**	p.K973M	1	3		■																			Missense_Mutation
**EHBP1L1**	p.P276P	1	0					■																Silent
**EHMT1**	p.G505S	2	4														■	■						Missense_Mutation
**EHMT1**	p.V212M	1	1			■																		Missense_Mutation
**EIF2AK3**	p.T528T	1	0													■								Silent
**EIF2AK4**	p.L391L	1	0							■														Silent
**EIF2S3L**	p.L377L	1	0		■																			Silent
**EIF3A**	p.M1093L	1	0														■							Missense_Mutation
**EIF4A2**	p.G322G	1	0		■																			Silent
**EIF4G1**	p.G1313V	2	6														■	■						Missense_Mutation
**EIF4G1**	p.G599G	1	0															■						Silent
**ELFN1**	p.S783R	2	0						■		■													Missense_Mutation
**ELFN2**	p.L281L	1	0			■																		Silent
**ELK4**	NULLL	1	1													■								Splice_Site
**ELMO3**	p.E449D	1	1					■																Missense_Mutation
**ELMO3**	p.R438fs	1	0					■																Frame_Shift_Del
**ELP6**	p.D147E	1	4				■																	Missense_Mutation
**EMC8**	p.K100N	1	2		■																			Missense_Mutation
**EMILIN2**	p.Y362Y	2	0														■	■						Silent
**ENGASE**	p.V489V	1	0		■																			Silent
**ENOX1**	p.R316W	3	7	■															■	■				Missense_Mutation
**ENPEP**	p.D735Y	1	8													■								Missense_Mutation
**ENTPD5**	p.A176V	1	7				■																	Missense_Mutation
**EP300**	p.V1841G	1	3									■												Missense_Mutation
**EP300**	p.T1411T	1	0				■																	Silent
**EP400**	p.L597R	1	7									■												Missense_Mutation
**EPB41L4A**	p.K398N	1	5													■								Missense_Mutation
**EPC2**	p.N795N	1	0							■														Silent
**EPG5**	p.L88L	1	0					■																Silent
**EPHA3**	p.N233S	1	3		■																			Missense_Mutation
**EPHB1**	p.L155P	1	5		■																			Missense_Mutation
**EPHB3**	p.K668*	1	2									■												Nonsense_Mutation

**EPHX2**	p.F20L	1	6													■								Missense_Mutation
**ERBB3**	p.G515G	1	0				■																	Silent
**ERBB4**	p.H505L	1	0										■											Missense_Mutation
**ERC1**	p.S1005F	1	6					■																Missense_Mutation
**ERCC6**	p.A544V	1	6				■																	Missense_Mutation
**ERF**	p.E411A	1	0			■																		Missense_Mutation
**ERG**	p.P223P	1	0		■																			Silent
**ERMARD**	p.L92L	1	0				■																	Silent
**ERN2**	p.L208L	1	0		■																			Silent
**ERO1LB**	p.R79*	2	2														■	■						Nonsense_Mutation
**ERP27**	p.I35T	1	7			■																		Missense_Mutation
**ERVW-1**	p.R433Q	1	0										■											Missense_Mutation
**ESPNL**	p.I982F	3	7	■															■	■				Missense_Mutation
**ESRP2**	p.A609A	1	0															■						Silent
**ETAA1**	p.G646*	1	2		■																			Nonsense_Mutation
**ETF1**	p.F373C	1	8													■								Missense_Mutation
**ETNPPL**	p.K238T	1	6													■								Missense_Mutation
**ETV5**	p.P256A	1	3												■									Missense_Mutation
**EVC**	p.S451F	1	3							■														Missense_Mutation
**EVPL**	p.E542K	1	7								■													Missense_Mutation
**EXO5**	p.R202H	1	0							■														Missense_Mutation
**EXOC1**	p.E659G	1	4														■							Missense_Mutation
**EXOC1**	p.N87K	1	2													■								Missense_Mutation
**EXOC6**	p.D346Y	1	7													■								Missense_Mutation
**EXOC6B**	p.E63*	1	2													■								Nonsense_Mutation
**EXOG**	p.I132V	3	0	■															■	■				Missense_Mutation
**EYA2**	p.V12I	3	2	■															■	■				Missense_Mutation
**EYS**	p.S2277S	1	1					■																Silent
**EYS**	p.S351*	1	1													■								Nonsense_Mutation
**EZH2**	p.G709S	1	9													■								Missense_Mutation
**EZR**	p.P480S	1	2		■																			Missense_Mutation
**F12**	p.S489R	1	1			■																		Missense_Mutation
**F5**	p.H1251Q	1	1		■																			Missense_Mutation
**F5**	p.T1261T	1	0							■														Silent
**F5**	p.L1256L	2	0										■				■							Silent
**F8**	p.N733S	1	1								■													Missense_Mutation
**FA2H**	p.Y206Y	1	0											■										Silent
**FABP2**	p.Q43H	1	3													■								Missense_Mutation
**FABP5**	p.M23V	1	4																	■				Missense_Mutation
**FAM102A**	p.A96V	1	6							■														Missense_Mutation
**FAM104B**	p.V115I	1	1				■																	Missense_Mutation
**FAM107A**	p.P65P	1	0							■														Silent
**FAM111A**	p.S354I	1	3					■																Missense_Mutation
**FAM111B**	p.N499H	1	4													■								Missense_Mutation
**FAM114A1**	NULLL	1	2													■								De_novo_Start_OutOfFrame
**FAM117B**	p.H534R	1	2					■																Missense_Mutation
**FAM117B**	p.T544S	1	0							■														Missense_Mutation
**FAM120B**	p.V377F	1	4									■												Missense_Mutation
**FAM120B**	p.G8*	1	1													■								Nonsense_Mutation
**FAM120B**	p.C416Y	1	1									■												Missense_Mutation
**FAM120B**	p.T391T	1	0							■														Silent
**FAM120B**	p.P407S	1	0														■							Missense_Mutation
**FAM120B**	p.S455T	1	0							■														Missense_Mutation
**FAM124B**	p.D29H	1	6													■								Missense_Mutation
**FAM134B**	p.G317G	1	0		■																			Silent
**FAM135B**	p.R725Q	1	5	■																				Missense_Mutation
**FAM153A**	p.R297Q	1	0							■														Missense_Mutation

**FAM155A**	p.R77R	1	0								■													Silent
**FAM178A**	p.R286S	1	2					■																Missense_Mutation
**FAM179B**	p.I1281S	1	6													■								Missense_Mutation
**FAM179B**	p.I1148I	1	0								■													Silent
**FAM193A**	p.S288A	1	5				■																	Missense_Mutation
**FAM193B**	p.P563A	2	0													■	■							Missense_Mutation
**FAM194B**	p.G120A	1	1								■													Missense_Mutation
**FAM194B**	p.E119K	1	1								■													Missense_Mutation
**FAM194B**	p.E114D	1	1								■													Missense_Mutation
**FAM194B**	p.G102V	1	1									■												Missense_Mutation
**FAM194B**	p.A101E	3	1					■			■						■							Missense_Mutation
**FAM194B**	p.E125E	1	0								■													Silent
**FAM194B**	p.E111E	1	0								■													Silent
**FAM208B**	p.E2419D	1	1													■								Missense_Mutation
**FAM214A**	p.D484N	2	4														■	■						Missense_Mutation
**FAM21A**	p.F1323L	2	1	■															■					Missense_Mutation
**FAM21C**	p.P960A	1	3		■																			Missense_Mutation
**FAM24B**	p.V19A	1	4					■																Missense_Mutation
**FAM47A**	p.L211L	1	0			■																		Silent
**FAM47B**	p.V573G	1	2				■																	Missense_Mutation
**FAM47C**	p.L755H	1	1						■															Missense_Mutation
**FAM47C**	p.P284P	1	0									■												Silent
**FAM47C**	p.E381E	1	0											■										Silent
**FAM47C**	p.L428P	2	0								■			■										Missense_Mutation
**FAM47C**	p.G435V	1	0								■													Missense_Mutation
**FAM47C**	p.P440P	1	0								■													Silent
**FAM47C**	p.E441E	1	0								■													Silent
**FAM47C**	p.V747V	2	0	■															■					Silent
**FAM49A**	p.V266A	1	5													■								Missense_Mutation
**FAM50A**	p.T150A	1	0			■																		Missense_Mutation
**FAM57B**	p.S158A	1	0		■																			Missense_Mutation
**FAM63A**	p.D361A	1	5									■												Missense_Mutation
**FAM65B**	p.L309L	2	0														■	■						Silent
**FAM69B**	p.G88R	1	1											■										Missense_Mutation
**FAM71B**	p.H99H	1	0					■																Silent
**FAM71E1**	p.L96R	1	7	■																				Missense_Mutation
**FAM83D**	p.K415N	1	0			■																		Missense_Mutation
**FAM92B**	p.S30L	1	4		■																			Missense_Mutation
**FANCB**	p.N178H	2	5														■	■						Missense_Mutation
**FANCC**	p.H514Y	3	1	■															■	■				Missense_Mutation
**FANCF**	p.R135L	3	0	■															■	■				Missense_Mutation
**FANCI**	p.A97T	1	1													■								Splice_Site
**FANCM**	p.E1024Q	1	3							■														Missense_Mutation
**FANCM**	p.N1251S	1	1					■																Missense_Mutation
**FARP2**	p.K261N	2	8	■															■					Missense_Mutation
**FAS**	NULLL	1	1													■								Splice_Site
**FASTKD2**	p.K175R	1	0			■																		Missense_Mutation
**FASTKD3**	p.S456P	1	3					■																Missense_Mutation
**FAT1**	p.R532W	1	8										■											Missense_Mutation
**FAT2**	p.S2779C	1	7				■																	Missense_Mutation
**FAT2**	p.L2524L	1	0					■																Silent
**FAT2**	p.K1722Q	1	0													■								Missense_Mutation
**FAT3**	p.E1333Q	1	1							■														Missense_Mutation
**FBN2**	p.P2064T	1	4															■						Missense_Mutation
**FBN2**	p.D1810D	2	0														■	■						Silent
**FBN2**	p.G1030G	1	0				■																	Silent
**FBRS**	p.A912A	1	0				■																	Silent

**FBXL13**	p.Y83C	1	4			■																		Missense_Mutation
**FBXO25**	p.E33G	1	1							■														Missense_Mutation
**FBXO40**	p.V387V	1	0					■																Silent
**FBXO41**	p.A95E	1	0																	■				Missense_Mutation
**FBXO47**	p.I38L	1	0				■																	Missense_Mutation
**FCF1**	p.V119V	1	0							■														Silent
**FCGBP**	p.E1957G	1	5			■																		Missense_Mutation
**FCGBP**	p.G4347S	1	2			■																		Missense_Mutation
**FCRL3**	p.I367M	1	3									■												Missense_Mutation
**FGD6**	p.P315S	1	8				■																	Missense_Mutation
**FGFR3**	NULLL	1	1														■							Splice_Site
**FGFR3**	NULLL	1	1														■							Splice_Site
**FGFR3**	NULLL	1	1														■							Splice_Site
**FGFR3**	NULLL	1	0														■							Splice_Site
**FGGY**	p.E9Q	2	1						■					■										Missense_Mutation
**FGR**	p.S486S	1	0								■													Silent
**FHOD3**	p.K1175Q	3	7	■															■	■				Missense_Mutation
**FHOD3**	p.G766G	1	0												■									Silent
**FKBP11**	p.S99N	1	5		■																			Missense_Mutation
**FKBP9**	p.H567Q	1	5									■												Missense_Mutation
**FLG**	p.R3743G	2	4														■	■						Missense_Mutation
**FLG**	p.S625R	3	3	■															■	■				Missense_Mutation
**FLG**	p.H3188D	1	1									■												Missense_Mutation
**FLG**	p.P2067P	1	0				■																	Silent
**FLG2**	p.S844C	1	5								■													Missense_Mutation
**FLG2**	p.S921N	1	4													■								Missense_Mutation
**FLG2**	p.S775F	1	4									■												Missense_Mutation
**FLG2**	p.G485V	1	4														■							Missense_Mutation
**FLG2**	p.G901E	1	3													■								Missense_Mutation
**FLG2**	p.S739T	1	3										■											Missense_Mutation
**FLG2**	p.Y583C	1	3									■												Missense_Mutation
**FLG2**	p.H918R	1	2													■								Missense_Mutation
**FLG2**	p.H918N	1	2					■																Missense_Mutation
**FLG2**	p.Q767H	3	2	■								■							■					Missense_Mutation
**FLG2**	p.G766D	2	2									■	■											Missense_Mutation
**FLG2**	p.G766R	5	2	■								■	■		■				■					Missense_Mutation
**FLG2**	p.G614R	1	2															■						Missense_Mutation
**FLG2**	p.C495R	1	2														■							Missense_Mutation
**FLG2**	p.L572F	1	1							■														Missense_Mutation
**FLG2**	p.V511G	1	1															■						Missense_Mutation
**FLG2**	p.C495S	1	1			■																		Missense_Mutation
**FLG2**	p.G965G	1	0												■									Silent
**FLG2**	p.T902T	2	0					■								■								Silent
**FLG2**	p.S768S	4	0	■					■			■							■					Silent
**FLG2**	p.G581G	3	0							■		■			■									Silent
**FLG2**	p.Q552Q	1	0														■							Silent
**FLI1**	p.A102T	3	2	■															■	■				Missense_Mutation
**FLNA**	p.S1411L	1	7					■																Missense_Mutation
**FMO2**	p.E314Q	1	2							■														Missense_Mutation
**FMO6P**	p.G9R	3	3	■															■	■				Missense_Mutation
**FMR1**	p.R193R	1	0			■																		Silent
**FNDC1**	p.R581K	2	1														■	■						Missense_Mutation
**FNDC3A**	p.R996R	1	0														■							Splice_Site
**FOXD2**	p.H289fs	1	0																	■				Frame_Shift_Ins
**FOXD4**	p.R197T	1	7			■																		Missense_Mutation
**FOXD4**	p.R253fs	1	0							■														Frame_Shift_Ins
**FOXD4L3**	p.G25R	1	3							■														Missense_Mutation

**FOXD4L3**	p.P378L	1	1	Missense_Mutation
**FOXI1**	p.V194E	1	3	Missense_Mutation
**FOXJ2**	p.P229A	1	1	Missense_Mutation
**FOXK1**	p.S459A	1	2	Missense_Mutation
**FOXL1**	p.A159A	1	0	Silent
**FPGS**	p.S441R	2	2	Missense_Mutation
**FRAS1**	p.Q2617L	1	5	Splice_Site
**FREM1**	p.V453I	3	2	Missense_Mutation
**FREM1**	p.Y1768F	2	1	Missense_Mutation
**FREM2**	p.C2164R	1	7	Missense_Mutation
**FREM2**	p.M327I	1	2	Missense_Mutation
**FRG1**	p.P93A	1	5	Missense_Mutation
**FRG1**	p.N144I	1	5	Splice_Site
**FRMD1**	p.G7E	1	2	Missense_Mutation
**FRMD6**	p.R129K	1	1	Missense_Mutation
**FRYL**	p.K1779R	1	3	Missense_Mutation
**FSIP1**	p.Q203H	1	5	Missense_Mutation
**FSIP2**	p.L6154F	1	2	Missense_Mutation
**FSIP2**	p.I6153I	1	0	Silent
**FSIP2**	p.A6292G	1	0	Missense_Mutation
**FSTL1**	p.D93V	1	2	Missense_Mutation
**FTH1**	p.56_57RR>R	1	0	In_Frame_Del
**FTSJ3**	p.E272K	2	5	Missense_Mutation
**FUCA2**	p.P288Q	1	2	Missense_Mutation
**FUT10**	NULLL	1	1	Splice_Site
**FYN**	p.N49D	3	3	Missense_Mutation
**FZD1**	p.S241W	1	4	Missense_Mutation
**FZD3**	p.A560V	1	5	Missense_Mutation
**FZD3**	p.P466P	1	0	Silent
**GABBR2**	p.G636E	1	5	Missense_Mutation
**GABRA6**	p.H158R	1	7	Missense_Mutation
**GABRG3**	p.KT351fs	2	0	Frame_Shift_Del
**GABRQ**	p.V347V	1	0	Silent
**GADD45B**	p.D34Y	1	7	Missense_Mutation
**GADD45GIP1**	p.A205A	2	0	Silent
**GAK**	p.N97H	1	6	Missense_Mutation
**GALC**	p.T558S	1	1	Missense_Mutation
**GALK1**	p.T223I	3	6	Missense_Mutation
**GALM**	p.N294N	1	0	Silent
**GAS2L1**	p.P286L	1	0	Missense_Mutation
**GBA2**	p.D354A	1	6	Missense_Mutation
**GBP1**	p.F368L	1	7	Missense_Mutation
**GBP1**	p.R522T	1	5	Missense_Mutation
**GCFC2**	p.R378K	2	4	Missense_Mutation
**GCM1**	p.S298R	1	1	Missense_Mutation
**GCNT2**	p.A179A	1	0	Silent
**GCNT4**	p.Q40R	1	0	Missense_Mutation
**GCNT4**	p.Q40E	1	0	Missense_Mutation
**GCNT6**	p.E93K	3	4	Missense_Mutation
**GCNT6**	p.R258C	1	2	Missense_Mutation
**GDAP2**	p.D333H	1	7	Missense_Mutation
**GDF2**	p.P86P	1	0	Silent
**GDF7**	p.Y427H	1	7	Missense_Mutation
**GDNF**	p.L163F	1	8	Missense_Mutation
**GDPD4**	p.L298L	2	0	Silent
**GFER**	p.R150K	1	1	Missense_Mutation
**GGA3**	p.L436V	1	0	Missense_Mutation

**GGH**	p.Q175Q	1	0							■														Silent
**GGT1**	p.Q158P	1	4									■												Missense_Mutation
**GGTLC1**	p.P105L	2	7			■		■																Missense_Mutation
**GJA10**	p.L283F	1	1					■																Missense_Mutation
**GK2**	p.L301L	1	0									■												Silent
**GKAP1**	p.G207R	3	2	■															■	■				Missense_Mutation
**GLB1L2**	p.K239E	1	1													■								Missense_Mutation
**GLDC**	p.Q265*	2	3														■	■						Nonsense_Mutation
**GLI2**	p.V377V	1	0								■													Silent
**GLRA2**	p.S81R	1	7			■																		Missense_Mutation
**GLRA3**	NULLL	1	1													■								Splice_Site
**GLTSCR1L**	p.G891G	1	0				■																	Silent
**GNAL**	p.V249I	1	2		■																			Missense_Mutation
**GNAS**	p.P364L	1	0											■										Missense_Mutation
**GNE**	p.D289H	1	4													■								Missense_Mutation
**GNLY**	p.R131H	2	4														■	■						Missense_Mutation
**GNPNAT1**	p.D83Y	1	6							■														Missense_Mutation
**GNPTG**	p.D215N	2	6														■	■						Missense_Mutation
**GNPTG**	p.S277F	1	3							■														Missense_Mutation
**GOLGA6L2**	p.G651R	1	0																	■				Missense_Mutation
**GOLGA6L2**	p.M364I	1	0															■						Missense_Mutation
**GOLGA8H**	p.E435D	1	0	■																				Missense_Mutation
**GOLGA8J**	p.P296A	1	1														■							Missense_Mutation
**GOLGA8J**	p.S260S	1	0			■																		Silent
**GOLGA8K**	p.E425D	1	0								■													Splice_Site
**GOLGA8M**	p.K327E	1	0		■																			Missense_Mutation
**GOLGA8Q**	p.I188I	1	0									■												Silent
**GOLGA8Q**	p.E209K	1	0					■																Missense_Mutation
**GOLGA8S**	p.D564E	2	0	■															■					Missense_Mutation
**GOLGB1**	p.K695T	1	4													■								Missense_Mutation
**GOLGB1**	p.Q757R	1	2														■							Missense_Mutation
**GP1BA**	p.P16A	1	0			■																		Missense_Mutation
**GP6**	p.Q377R	1	3					■																Missense_Mutation
**GPATCH1**	p.N649N	2	0														■	■						Silent
**GPATCH11**	p.E96K	1	2							■														Missense_Mutation
**GPATCH8**	p.S40L	1	6													■								Missense_Mutation
**GPC6**	p.S191R	1	5											■										Missense_Mutation
**GPC6**	p.R229T	1	5							■														Missense_Mutation
**GPD1L**	p.D197N	1	2									■												Missense_Mutation
**GPR107**	p.I146M	1	1							■														Missense_Mutation
**GPR108**	p.L341L	3	0	■															■	■				Silent
**GPR110**	p.V509A	1	1													■								Missense_Mutation
**GPR112**	p.L1223M	1	3												■									Missense_Mutation
**GPR112**	p.L2175P	1	2							■														Missense_Mutation
**GPR124**	p.S539R	1	3														■							Missense_Mutation
**GPR149**	p.A382V	2	1														■	■						Missense_Mutation
**GPR149**	p.F302F	1	0		■																			Silent
**GPR149**	p.A166A	1	0		■																			Silent
**GPR155**	p.D128H	1	4							■														Missense_Mutation
**GPR75**	p.D245N	1	4													■								Missense_Mutation
**GPR78**	p.A320A	1	0														■							Silent
**GPR98**	p.D3264G	1	2				■																	Missense_Mutation
**GPR98**	p.Q1780Q	1	0		■																			Silent
**GPR98**	p.L5965L	1	0			■																		Silent
**GPRASP1**	p.M380R	1	0															■						Missense_Mutation
**GPRC5D**	p.A305A	1	0					■																Silent
**GPRC5D**	p.V241V	3	0	■															■	■				Silent

**GRB7**	p.Q80L	1	1						■															Missense_Mutation
**GREB1L**	p.E798K	2	6						■					■										Missense_Mutation
**GRHL2**	p.W36*	1	2													■								Nonsense_Mutation
**GRIA1**	p.A258T	1	4				■																	Missense_Mutation
**GRIA3**	p.N63Y	1	8							■														Missense_Mutation
**GRIA3**	p.N61Y	2	7		■					■														Missense_Mutation
**GRID2IP**	p.L178L	1	0				■																	Silent
**GRIK2**	p.A856D	1	4							■														Missense_Mutation
**GRIN2B**	p.Y322Y	3	0	■															■	■				Silent
**GRIPAP1**	p.E117A	1	0			■																		Missense_Mutation
**GRM1**	p.A11A	1	0													■								Silent
**GRM5**	p.A593A	1	0					■																Silent
**GRM7**	p.P650S	1	8													■								Missense_Mutation
**GRPEL1**	p.D43V	1	4				■																	Missense_Mutation
**GSAP**	p.D636D	3	0	■															■	■				Silent
**GSTCD**	p.P392A	4	4	■				■									■		■					Missense_Mutation
**GSTO2**	p.M145T	2	7														■	■						Missense_Mutation
**GTF2IRD1**	p.R931Q	3	0	■															■	■				Missense_Mutation
**GTF3C4**	p.L501V	1	0			■																		Missense_Mutation
**GTPBP3**	p.P30S	1	0														■							Missense_Mutation
**GTSE1**	p.R493W	1	7			■																		Missense_Mutation
**GUCY1A3**	p.I331I	1	0										■											Silent
**GZMM**	p.M40K	1	7															■						Missense_Mutation
**HARS2**	p.K76R	1	2								■													Missense_Mutation
**HAUS5**	p.S223S	3	0	■															■	■				Silent
**HAUS8**	p.N310T	1	1					■																Missense_Mutation
**HAVCR1**	p.M182K	1	1					■																Missense_Mutation
**HDAC1**	p.F150L	1	8		■																			Missense_Mutation
**HDAC4**	p.G204R	2	3														■	■						Splice_Site
**HDLBP**	p.K726K	1	1							■														Silent
**HEATR1**	p.L636V	1	2													■								Missense_Mutation
**HEATR4**	p.S739F	3	6	■															■	■				Missense_Mutation
**HEATR4**	p.L869L	1	0							■														Silent
**HEATR4**	p.N482D	1	0			■																		Missense_Mutation
**HEATR4**	p.P161A	1	0									■												Missense_Mutation
**HEATR5A**	p.T85I	1	4					■																Missense_Mutation
**HEATR5B**	p.P1803S	1	0			■																		Missense_Mutation
**HECTD2**	p.A258T	1	4					■																Missense_Mutation
**HECTD4**	p.I539M	1	3							■														Missense_Mutation
**HECW1**	p.E747A	1	3							■														Missense_Mutation
**HECW1**	p.H951H	3	0	■															■	■				Silent
**HECW2**	p.T708I	1	7													■								Missense_Mutation
**HEMK1**	p.Y258C	1	7				■																	Missense_Mutation
**HEPH**	p.T890T	1	0				■																	Silent
**HERC6**	p.D781H	1	5															■						Missense_Mutation
**HEXDC**	p.H504Y	1	1										■											Missense_Mutation
**HGF**	p.Y392Y	3	0	■															■	■				Silent
**HGS**	p.T716P	1	0															■						Missense_Mutation
**HHATL**	p.S390*	1	3														■							Nonsense_Mutation
**HHIP**	p.-12fs	1	0							■														Frame_Shift_Ins
**HIP1**	p.D371N	1	6													■								Missense_Mutation
**HIP1**	p.E383E	1	0													■								Silent
**HIPK1**	p.P1135S	1	2			■																		Missense_Mutation
**HIPK1**	p.L1044L	1	1				■																	Silent
**HIRIP3**	p.E146D	1	0						■															Missense_Mutation
**HIST1H2BE**	p.I95I	3	0	■															■	■				Silent
**HIST1H2BI**	NULLL	1	0		■																			De_novo_Start_OutOfFrame

**HIST1H3D**	p.E74K	1	3													■								Missense_Mutation
**HIST1H4C**	p.M1T	1	4					■																Start_Codon_SNP
**HIST3H2A**	p.K16T	1	5													■								Missense_Mutation
**HIST3H3**	p.C97C	1	0													■								Silent
**HIVEP1**	p.V1802I	1	1			■																		Missense_Mutation
**HIVEP2**	p.I556S	1	0													■								Missense_Mutation
**HJURP**	p.S295Y	1	1					■																Missense_Mutation
**HKDC1**	p.F622F	1	0							■														Silent
**HLA-DQA2**	p.D184E	1	0														■							Missense_Mutation
**HLA-DQB2**	p.S173P	1	1														■							Missense_Mutation
**HMG20A**	p.R162C	1	8													■								Missense_Mutation
**HMGB3**	p.EE197fs	1	0								■													Frame_Shift_Del
**HMGCLL1**	p.204_205HM>	3	0	■															■	■				In_Frame_Del
**HN1L**	p.T39R	1	6							■														Missense_Mutation
**HNRNPCL1**	p.I82T	1	3					■																Missense_Mutation
**HNRNPUL2**	p.E45K	1	2																	■				Missense_Mutation
**HOGA1**	p.S27S	1	0						■															Silent
**HOOK1**	p.L381R	1	7													■								Missense_Mutation
**HOXC11**	p.R288K	1	5				■																	Missense_Mutation
**HPDL**	p.TA204fs	1	0		■																			Frame_Shift_Del
**HPGD**	p.G93E	1	8															■						Missense_Mutation
**HRC**	p.H300Q	1	0								■													Missense_Mutation
**HRNR**	p.G1991G	1	0														■							Silent
**HS3ST3A1**	p.I211V	1	1		■																			Missense_Mutation
**HSD17B11**	p.I41F	1	8			■																		Missense_Mutation
**HSD17B4**	p.V625A	3	1	■															■	■				Missense_Mutation
**HSP90B1**	p.L698I	1	6		■																			Missense_Mutation
**HSPA12B**	p.R292C	1	5																	■				Missense_Mutation
**HSPA12B**	p.G399D	1	0								■													Missense_Mutation
**HSPA2**	p.E176A	1	6										■											Missense_Mutation
**HSPA9**	p.D357V	3	6	■															■	■				Missense_Mutation
**HSPBAP1**	p.I72V	2	1														■	■						Missense_Mutation
**HSPG2**	p.N1256T	1	5																	■				Missense_Mutation
**HSPG2**	NULLL	2	1	■															■					Splice_Site
**HTR3B**	p.G425V	1	3									■												Missense_Mutation
**HTR3D**	p.V61V	3	0	■															■	■				Silent
**HTR3D**	p.L316L	3	0	■															■	■				Silent
**HTT**	p.L2221V	1	5		■																			Missense_Mutation
**HUWE1**	p.R1675R	2	1														■	■						Silent
**HUWE1**	p.G1654G	1	1				■																	Silent
**HYDIN**	p.R2361L	1	5							■														Missense_Mutation
**HYDIN**	p.H4594R	1	3														■							Missense_Mutation
**HYDIN**	p.E1509G	1	2					■																Missense_Mutation
**HYDIN**	p.A3161A	3	0	■															■	■				Silent
**HYDIN**	p.S1970S	1	0			■																		Silent
**IAPP**	p.A75A	1	6										■											Silent
**IARS**	p.L1152P	1	7		■																			Missense_Mutation
**ICA1**	p.LSFFFFFF321	1	0									■												Frame_Shift_Del
**IDE**	p.E997K	1	2													■								Missense_Mutation
**IDH3B**	NULLL	1	1															■						Splice_Site
**IDS**	p.L453L	1	0									■												Silent
**IFIT3**	p.V157V	1	0				■																	Silent
**IFIT5**	p.N306N	1	0								■													Silent
**IFITM1**	p.S60S	2	0						■					■										Silent
**IFNA6**	p.S177L	1	0							■														Missense_Mutation
**IFT81**	p.E157K	2	4														■	■						Missense_Mutation
**IFT88**	p.D817N	1	3													■								Missense_Mutation

**IGDCC4**	p.L937L	1	0			■																		Silent
**IGDCC4**	p.Q152Q	1	0										■											Silent
**IGF2**	p.H168P	1	5			■																		Missense_Mutation
**IGF2**	p.T163P	2	1	■															■					Missense_Mutation
**IGF2BP3**	p.D270H	1	7										■											Missense_Mutation
**IGF2R**	p.T402T	1	0			■																		Silent
**IGFN1**	p.L3665M	1	1													■								Missense_Mutation
**IGLL1**	p.D152N	2	1														■	■						Missense_Mutation
**IGSF10**	p.Q500R	1	0		■																			Missense_Mutation
**IKBIP**	p.L303V	1	0													■								Missense_Mutation
**IKBIP**	p.T245M	1	0													■								Missense_Mutation
**IKZF4**	p.P433P	2	0	■															■					Silent
**IL16**	p.S524G	1	3								■													Missense_Mutation
**IL17RC**	p.Q373*	2	1														■	■						Nonsense_Mutation
**IL1RAP**	p.R588R	1	0						■															Silent
**IL20RA**	p.T202K	1	4					■																Missense_Mutation
**IL27RA**	p.A415A	1	0	■																				Splice_Site
**IL32**	NULLL	1	0			■																		De_novo_Start_OutOfFrame
**IL5RA**	p.L373W	1	5										■											Missense_Mutation
**IMP4**	p.P158L	1	6					■																Missense_Mutation
**INCA1**	p.T55S	1	0		■																			Missense_Mutation
**INHBA**	p.T23P	1	2										■											Missense_Mutation
**INMT**	p.C213C	2	0														■	■						Silent
**INO80**	p.P76A	1	3			■																		Missense_Mutation
**INPP5B**	p.L415L	1	0												■									Silent
**INPP5F**	p.F806F	1	0											■										Silent
**INTS1**	p.M1624I	3	0	■															■	■				Missense_Mutation
**INTU**	p.M508V	1	4		■																			Missense_Mutation
**IPO8**	p.Q324R	1	6													■								Missense_Mutation
**IQSEC1**	p.M699I	1	1													■								Missense_Mutation
**IQUB**	p.R338T	1	7								■													Missense_Mutation
**IQUB**	p.Q299L	1	7								■													Missense_Mutation
**IQUB**	p.Q299K	1	6								■													Missense_Mutation
**IQUB**	p.E72G	1	0								■													Missense_Mutation
**IRGQ**	p.D298A	1	7							■														Missense_Mutation
**IRS1**	p.SSN685fs	3	0	■															■	■				Frame_Shift_Del
**IRX1**	p.K331T	1	5								■													Missense_Mutation
**ISM1**	p.G134S	1	2			■																		Missense_Mutation
**ITGA11**	p.L185L	1	0							■														Silent
**ITGA8**	p.V724L	1	5			■																		Missense_Mutation
**ITGA8**	p.I460V	1	2		■																			Missense_Mutation
**ITGAE**	p.A931V	1	0				■																	Missense_Mutation
**ITGAL**	p.S989R	1	1				■																	Missense_Mutation
**ITGAM**	p.V119A	1	0													■								Missense_Mutation
**ITGB3BP**	p.R95I	1	3													■								Missense_Mutation
**ITGB6**	p.R735S	3	1	■															■	■				Missense_Mutation
**ITIH5**	NULLL	1	1															■						Splice_Site
**ITK**	p.V377V	1	0					■																Silent
**ITLN1**	NULLL	1	1											■										Splice_Site
**ITM2A**	p.R206H	1	0				■																	Missense_Mutation
**ITPKB**	p.L450R	1	1									■												Missense_Mutation
**ITPKB**	p.S355R	1	1									■												Missense_Mutation
**ITPRIP**	p.E498K	1	4					■																Missense_Mutation
**ITPRIP**	p.S535C	1	3										■											Missense_Mutation
**ITPRIPL1**	p.Q441E	1	0									■												Missense_Mutation
**ITSN2**	p.A1058A	1	0							■														Silent
**JAK1**	p.L103L	1	0													■								Silent

**JAK3**	p.E739A	2	4				Missense_Mutation
**JMJD6**	p.A75A	1	0				Silent
**JMJD7-PLA2**	p.F804L	1	7				Missense_Mutation
**KALRN**	p.E47A	1	7				Missense_Mutation
**KALRN**	p.K64R	1	3				Missense_Mutation
**KALRN**	p.I119I	3	0				Silent
**KANSL1**	p.S917*	1	1				Nonsense_Mutation
**KAT2A**	p.E312K	1	4				Missense_Mutation
**KATNAL1**	p.K374Q	1	8				Missense_Mutation
**KATNAL1**	p.I306V	1	5				Missense_Mutation
**KATNBL1**	p.S225L	1	2				Missense_Mutation
**KBTBD4**	p.S465A	2	5				Missense_Mutation
**KBTBD6**	p.Q606Q	1	0				Silent
**KBTBD7**	p.I25M	1	0				Missense_Mutation
**KCNA2**	p.Y68Y	1	0				Silent
**KCNA3**	p.V173G	1	5				Missense_Mutation
**KCNA5**	p.D277D	1	0				Silent
**KCNC4**	p.G91D	1	0				Missense_Mutation
**KCNH3**	p.E1011A	1	3				Missense_Mutation
**KCNH4**	p.S724S	1	0				Silent
**KCNH7**	p.V812V	1	0				Silent
**KCNH8**	p.S1062S	2	0				Silent
**KCNJ12**	p.V23V	1	0				Silent
**KCNK1**	p.I304N	1	3				Missense_Mutation
**KCNK10**	p.G86G	1	0				Silent
**KCNK17**	p.G167E	1	0				Missense_Mutation
**KCNK18**	p.S252S	3	0				Silent
**KCNK4**	p.V162G	1	6				Missense_Mutation
**KCNK5**	p.L327R	1	0				Missense_Mutation
**KCNMB1**	p.S112C	1	2				Missense_Mutation
**KCNMB2**	p.*236L	3	0				Nonstop_Mutation
**KCNS1**	p.Q480L	1	1				Missense_Mutation
**KCNT2**	p.D1066G	2	8				Missense_Mutation
**KCNU1**	p.R1046S	1	0				Missense_Mutation
**KCTD17**	p.S204A	1	0				Missense_Mutation
**KCTD21**	p.M32I	1	6				Missense_Mutation
**KDM4C**	p.L102L	3	0				Silent
**KDM5A**	p.Q529Q	1	0				Silent
**KDM5C**	p.G452G	1	0				Silent
**KIAA0226**	p.A649G	2	6				Missense_Mutation
**KIAA0355**	p.Q704P	1	3				Missense_Mutation
**KIAA0355**	p.L487L	1	0				Silent
**KIAA0586**	p.S60F	1	5				Missense_Mutation
**KIAA0907**	p.Q415H	1	1				Missense_Mutation
**KIAA1024**	p.E390K	1	2				Missense_Mutation
**KIAA1109**	p.C1133S	1	5				Missense_Mutation
**KIAA1211**	p.R992R	1	0				Silent
**KIAA1239**	p.M675T	2	2				Missense_Mutation
**KIAA1324L**	p.T756T	1	1				Silent
**KIAA1324L**	p.I644V	1	1				Missense_Mutation
**KIAA1551**	p.Q37H	1	1				Missense_Mutation
**KIAA1671**	p.S1506R	1	1				Missense_Mutation
**KIAA1671**	p.S577S	1	0				Silent
**KIAA1671**	p.Q580R	1	0				Missense_Mutation
**KIAA1671**	p.R702Q	1	0				Missense_Mutation
**KIAA1683**	p.R1176C	1	5				Missense_Mutation
**KIAA1755**	p.P486R	1	2				Missense_Mutation

**KIAA1755**	p.P466H	1	1							■														Missense_Mutation
**KIAA1841**	p.L232I	1	7								■													Missense_Mutation
**KIAA1875**	p.Q56Q	3	0	■															■	■				Silent
**KIAA1984**	p.T278M	1	0				■																	Missense_Mutation
**KIAA2026**	p.N1685K	1	2													■								Missense_Mutation
**KIDINS220**	p.S1623S	3	0	■															■	■				Silent
**KIF13A**	p.I1284T	1	1				■																	Missense_Mutation
**KIF15**	p.R463*	1	1					■																Nonsense_Mutation
**KIF1C**	p.Q1066K	1	0			■																		Missense_Mutation
**KIF20B**	p.N589H	1	3													■								Missense_Mutation
**KIF21B**	p.D622H	1	7							■														Missense_Mutation
**KIF22**	p.L581L	1	0													■								Silent
**KIF24**	p.D1175N	1	0													■								Missense_Mutation
**KIF26A**	p.C325C	2	0	■																■				Silent
**KIF4B**	p.Q393*	1	1							■														Nonsense_Mutation
**KIR2DL1**	p.G179R	4	3	■							■								■	■				Missense_Mutation
**KIR2DL3**	p.L38L	3	0	■															■	■				Silent
**KIR3DL1**	p.C195R	1	5													■								Missense_Mutation
**KIRREL**	p.T21P	1	1															■						Missense_Mutation
**KIRREL3**	p.S683R	1	4									■												Missense_Mutation
**KLF6**	p.G236A	1	6								■													Missense_Mutation
**KLF6**	p.I67I	1	2													■								Silent
**KLHDC4**	p.T263T	1	0				■																	Silent
**KLHL1**	p.Y650F	1	8			■																		Missense_Mutation
**KLHL14**	p.R500R	1	0		■																			Silent
**KLHL20**	p.I277I	1	0													■								Silent
**KLHL20**	p.V538V	1	0				■																	Silent
**KLHL21**	p.T550S	2	5														■	■						Missense_Mutation
**KLHL29**	p.G838G	3	0	■															■	■				Silent
**KLHL32**	p.D43N	1	8													■								Missense_Mutation
**KLHL5**	p.E167D	1	2													■								Missense_Mutation
**KLK13**	p.T267I	1	3				■																	Missense_Mutation
**KLK14**	p.P15L	1	2		■																			Missense_Mutation
**KLK2**	p.G158V	1	8				■																	Missense_Mutation
**KLK3**	p.V238I	1	2			■																		Missense_Mutation
**KLRG1**	p.T114R	3	0	■															■	■				Missense_Mutation
**KLRK1**	p.Y199Y	3	0	■															■	■				Silent
**KMO**	p.L58H	1	7													■								Missense_Mutation
**KMO**	p.R63G	1	6													■								Missense_Mutation
**KMT2C**	p.Q3836Q	1	1				■																	Silent
**KMT2D**	p.H1707Q	1	6			■																		Missense_Mutation
**KPNA2**	p.S488L	1	4													■								Missense_Mutation
**KPNA7**	p.E492E	1	0		■																			Silent
**KPNA7**	p.T51T	1	0													■								Silent
**KRBA1**	p.G818R	3	3	■															■	■				Missense_Mutation
**KRBOX4**	p.AGEDRP79fs	1	0							■														Frame_Shift_Del
**KREMEN1**	p.E77V	2	6	■															■					Missense_Mutation
**KRT16**	p.G21G	1	0			■																		Silent
**KRT17**	p.R406C	1	4			■																		Missense_Mutation
**KRT17**	p.R122R	2	0	■			■																	Silent
**KRT35**	p.D76A	1	6						■															Missense_Mutation
**KRT5**	p.L296L	1	1							■														Silent
**KRT74**	p.A32A	1	0													■								Silent
**KRTAP10-10**	p.S139A	1	2									■												Missense_Mutation
**KRTAP10-10**	p.Y75C	2	0														■	■						Missense_Mutation
**KRTAP10-10**	p.P141P	1	0											■										Silent
**KRTAP10-4**	p.G316A	1	1										■											Missense_Mutation

**KRTAP10-5**	p.C158C	1	0			■																		Silent
**KRTAP10-5**	p.L100V	1	0										■											Missense_Mutation
**KRTAP10-5**	p.P96P	2	0	■															■					Silent
**KRTAP10-6**	p.C156C	2	0				■						■											Silent
**KRTAP10-7**	p.L102L	1	0									■												Silent
**KRTAP10-7**	p.S148S	3	0	■														■	■					Silent
**KRTAP10-9**	p.P94S	1	1											■										Missense_Mutation
**KRTAP10-9**	p.T91T	1	0						■															Silent
**KRTAP16-1**	p.T390M	2	1														■	■						Missense_Mutation
**KRTAP21-2**	p.G26G	3	0	■													■		■					Silent
**KRTAP21-2**	p.S21S	1	0					■																Silent
**KRTAP4-1**	p.R104R	1	0																	■				Silent
**KRTAP4-11**	p.P185A	1	3				■																	Missense_Mutation
**KRTAP4-6**	p.P82P	1	0															■						Silent
**KRTAP4-7**	p.P145A	2	2	■															■					Missense_Mutation
**KRTAP4-9**	p.Y80F	1	2															■						Missense_Mutation
**KRTAP5-1**	p.G250G	1	0				■																	Silent
**KRTAP5-3**	p.C200Y	1	5																■					Missense_Mutation
**KRTAP5-3**	p.S149S	1	0										■											Silent
**KRTAP5-4**	p.S195C	1	0								■													Missense_Mutation
**KRTAP5-5**	p.C179*	1	1									■												Nonsense_Mutation
**KRTAP5-5**	p.S203C	1	0								■													Missense_Mutation
**KRTAP9-9**	p.R112G	1	1					■																Missense_Mutation
**KRTDAP**	p.N81N	1	0		■																			Silent
**KSR2**	p.L241Q	1	1															■						Missense_Mutation
**L1TD1**	p.E122*	1	1													■								Nonsense_Mutation
**L1TD1**	p.F469L	1	0													■								Missense_Mutation
**LACTB**	p.E195K	1	5													■								Missense_Mutation
**LAMB1**	p.T183A	1	6									■												Missense_Mutation
**LAMB1**	p.A897A	1	0							■														Silent
**LAMB2**	p.R1594S	1	3													■								Splice_Site
**LAMB2**	p.A1571T	1	1													■								Missense_Mutation
**LAMB2**	p.I715V	1	1					■																Missense_Mutation
**LAMC2**	p.D532Y	1	7				■																	Missense_Mutation
**LAMC3**	p.L1390L	1	1		■																			Silent
**LARP1B**	p.N398T	1	2													■								Missense_Mutation
**LARP4**	p.S491*	1	1													■								Nonsense_Mutation
**LARP4**	p.T246T	1	0					■																Silent
**LARP4B**	p.S718A	1	4												■									Missense_Mutation
**LARP4B**	p.S720S	2	1														■	■						Silent
**LARS2**	p.L728F	1	6													■								Missense_Mutation
**LATS2**	p.K714K	1	0					■																Silent
**LCAT**	p.Y339S	1	7		■																			Missense_Mutation
**LCP2**	p.P210R	1	0			■																		Missense_Mutation
**LDB1**	p.T278T	1	0		■																			Silent
**LDB3**	p.K251N	2	5						■					■										Missense_Mutation
**LDHAL6B**	p.G345G	1	0							■														Silent
**LDLRAD3**	p.P275L	1	7		■																			Missense_Mutation
**LDLRAD4**	p.P111L	3	4	■															■	■				Missense_Mutation
**LEMD3**	p.S753Y	1	3					■																Missense_Mutation
**LEPRE1**	p.A195A	1	0															■						Silent
**LEPREL1**	p.S544Y	1	6				■																	Missense_Mutation
**LGMN**	p.T104A	1	6							■														Missense_Mutation
**LGR4**	p.L597I	1	7							■														Missense_Mutation
**LGSN**	p.L95L	3	0	■															■	■				Silent
**LHFPL4**	NULLL	2	1														■	■						Splice_Site
**LIAS**	p.R26G	1	0					■																Missense_Mutation

**LIFR**	p.A1032D	1	5							■														Missense_Mutation
**LIG3**	p.S242R	2	0												■	■								Missense_Mutation
**LILRA3**	p.Q230Q	1	0													■								Silent
**LILRB2**	p.G12R	3	3	■															■	■				Splice_Site
**LIMK1**	p.I486F	1	8					■																Missense_Mutation
**LIMK1**	p.C221G	1	3									■												Missense_Mutation
**LINC00692**	p.K98Q	1	1			■																		Missense_Mutation
**LINC00935**	p.E58K	1	0																	■				Missense_Mutation
**LIPE**	p.C464S	1	6								■													Missense_Mutation
**LL22NC03-63**	p.H19P	1	1					■																Missense_Mutation
**LMAN1L**	p.P451P	1	0					■																Silent
**LMF1**	p.P457L	1	7										■											Missense_Mutation
**LMLN**	p.V533I	1	1			■																		Missense_Mutation
**LMNA**	p.I299M	1	6			■																		Missense_Mutation
**LPAR4**	p.R136H	1	9								■													Missense_Mutation
**LPCAT4**	p.L250F	2	2														■	■						Splice_Site
**LPHN3**	p.A71V	1	5							■														Missense_Mutation
**LPIN1**	p.R206R	1	0					■																Silent
**LPIN2**	p.S151F	1	5									■												Missense_Mutation
**LPIN2**	p.L255V	3	4	■															■	■				Missense_Mutation
**LPIN2**	p.L58L	1	0				■																	Silent
**LPO**	p.R148W	1	6		■																			Splice_Site
**LRCH3**	p.E473Q	1	2					■																Missense_Mutation
**LRIG2**	p.F643F	2	0		■		■																	Silent
**LRP1**	p.M2186R	1	5			■																		Missense_Mutation
**LRP1**	p.L3268L	1	0							■														Silent
**LRP12**	p.S421S	1	0				■																	Silent
**LRP2**	p.L2051L	1	0													■								Silent
**LRRC16B**	p.D130A	1	2										■											Missense_Mutation
**LRRC17**	p.W346*	1	2					■																Nonsense_Mutation
**LRRC28**	p.E4K	1	4													■								Missense_Mutation
**LRRC30**	p.R23S	2	0														■	■						Missense_Mutation
**LRRC39**	p.I331M	3	0	■															■	■				Missense_Mutation
**LRRC43**	p.R444G	1	2			■																		Missense_Mutation
**LRRC56**	p.P278P	1	0					■																Silent
**LRRC8C**	p.R797Q	1	4							■														Missense_Mutation
**LRRD1**	NULLL	1	1							■														Splice_Site
**LSM11**	p.L116L	1	0																	■				Silent
**LST3**	p.C463S	2	8	■															■					Missense_Mutation
**LTBP2**	p.E1753A	1	5				■																	Missense_Mutation
**LTBP2**	NULLL	2	1						■					■										Splice_Site
**LUC7L3**	p.I61I	1	0					■																Silent
**LUZP4**	p.G206S	1	1		■																			Missense_Mutation
**LUZP4**	p.G213G	2	0		■			■																Silent
**LUZP4**	p.G220G	1	0					■																Silent
**LUZP4**	p.V239L	2	0			■		■																Missense_Mutation
**LY6H**	p.D108E	1	3													■								Missense_Mutation
**LY75-CD302**	p.N369I	1	6							■														Missense_Mutation
**LYG2**	p.T165R	1	2				■																	Missense_Mutation
**LYSMD3**	p.H22R	1	5			■																		Missense_Mutation
**LZTS1**	p.R303M	1	0					■																Missense_Mutation
**MACC1**	p.S112A	2	3														■	■						Missense_Mutation
**MACF1**	p.H2366Y	1	1			■																		Missense_Mutation
**MAD1L1**	p.S614S	2	0														■	■						Silent
**MADD**	p.S916N	2	2														■	■						Missense_Mutation
**MAFB**	p.A89A	1	0								■													Silent
**MAGEB2**	p.S161G	1	2			■																		Missense_Mutation

**MAGEC1**	p.P54S	1	4									■												Missense_Mutation
**MAGEC1**	p.Q582L	1	4									■												Missense_Mutation
**MAGEC1**	p.S383Y	1	3									■												Missense_Mutation
**MAGEC1**	p.V272M	1	2												■									Missense_Mutation
**MAGEC1**	p.L373V	1	1							■														Missense_Mutation
**MAGEC1**	p.S313S	1	0												■									Silent
**MAGEC1**	p.T329A	1	0																	■				Missense_Mutation
**MAGEE1**	p.S442S	1	0											■										Silent
**MAGEE2**	p.E462G	2	3														■	■						Missense_Mutation
**MAGI1**	p.T1274P	1	3							■														Missense_Mutation
**MAMDC4**	p.T168P	1	3									■												Missense_Mutation
**MAML3**	p.F185L	1	4													■								Missense_Mutation
**MAMLD1**	p.S719S	1	0			■																		Silent
**MAN1B1**	p.R592G	1	4							■														Missense_Mutation
**MAN1C1**	p.V220V	1	0										■											Silent
**MAN2B1**	p.S579L	1	1		■																			Missense_Mutation
**MAN2B1**	p.E203E	1	0		■																			Silent
**MAN2B2**	p.L569R	1	1			■																		Missense_Mutation
**MAN2C1**	p.P652L	1	3				■																	Missense_Mutation
**MANF**	p.I105S	1	7													■								Missense_Mutation
**MAOB**	p.L372L	1	0							■														Silent
**MAP1A**	p.E1690D	1	0												■									Missense_Mutation
**MAP2K2**	p.G153C	1	8					■																Missense_Mutation
**MAP3K12**	p.E487D	1	7							■														Missense_Mutation
**MAP3K19**	p.D1139H	1	2															■						Missense_Mutation
**MAP3K4**	p.F570F	1	0			■																		Silent
**MAP4**	p.V2162V	1	0										■											Silent
**MAP6**	p.I620M	1	0							■														Missense_Mutation
**MAP6**	p.I609M	2	0								■								■					Missense_Mutation
**MAP7**	p.V448V	1	0					■																Silent
**MAP7D3**	p.D309D	1	0			■																		Silent
**MAPK10**	p.E217*	1	3													■								Nonsense_Mutation
**MAPT**	p.E336A	2	3	■															■					Missense_Mutation
**MARK3**	p.P484P	2	1	■															■					Silent
**MARK4**	p.G702G	1	0	■																				Silent
**MARS2**	p.R157C	1	7						■															Missense_Mutation
**MAS1L**	p.V222A	1	0		■																			Missense_Mutation
**MASP1**	NULLL	2	0														■	■						De_novo_Start_OutOfFrame
**MAST4**	p.Q1730Q	3	1	■															■	■				Silent
**MATN2**	p.L479F	1	5			■																		Missense_Mutation
**MATR3**	p.N788N	1	0									■												Silent
**MBD3L1**	p.L148L	1	0					■																Silent
**MBD4**	p.P560P	1	0					■																Silent
**MBD6**	p.S788L	1	3							■														Missense_Mutation
**MBD6**	p.L801F	1	3							■														Missense_Mutation
**MBNL2**	p.A315T	1	3													■								Missense_Mutation
**MBNL2**	p.E334*	1	1													■								Nonsense_Mutation
**MBOAT2**	p.A38V	1	2			■																		Missense_Mutation
**MBTPS1**	p.A416S	1	4				■																	Missense_Mutation
**MCAM**	p.S593A	1	3							■														Missense_Mutation
**MCF2L**	p.R621C	1	5					■																Missense_Mutation
**MCM2**	p.L319L	1	0			■																		Silent
**MCM3AP**	p.K395E	3	0	■															■	■				Missense_Mutation
**MCPH1**	p.S454G	1	5													■								Missense_Mutation
**MDC1**	p.S1365T	1	0														■							Missense_Mutation
**MDC1**	p.P1303P	1	0								■													Silent
**MDGA2**	p.I461K	1	2							■														Missense_Mutation

**MDH2**	p.P214H	2	5	■							■													Missense_Mutation
**MDH2**	p.P214T	1	1	■																				Missense_Mutation
**MEA1**	p.A102T	1	4													■								Splice_Site
**MED13L**	p.S445F	2	3														■	■						Missense_Mutation
**MED13L**	p.I982L	3	2	■															■	■				Missense_Mutation
**MED14**	p.L335L	2	0														■	■						Silent
**MED15**	p.P281L	1	4					■																Missense_Mutation
**MED15**	p.R67*	1	2													■								Nonsense_Mutation
**MED25**	p.S234A	2	4						■			■												Missense_Mutation
**MEF2A**	p.Q416P	1	1	■																				Missense_Mutation
**MEF2A**	p.A496T	3	1	■															■	■				Missense_Mutation
**MEGF8**	p.T2274S	1	1							■														Missense_Mutation
**MEGT1**	p.M243L	1	0			■																		Missense_Mutation
**MEI4**	p.V51L	1	0															■						Missense_Mutation
**MEN1**	p.K514K	1	0													■								Silent
**MEP1A**	p.R357G	1	7					■																Missense_Mutation
**MESDC2**	p.G164R	2	7														■	■						Missense_Mutation
**MESDC2**	p.R226Q	1	2		■																			Missense_Mutation
**METTL14**	p.K271N	1	3													■								Missense_Mutation
**METTL15**	p.K93K	1	0				■																	Silent
**METTL25**	p.I175V	1	0										■											Missense_Mutation
**METTL4**	p.E239D	1	5													■								Missense_Mutation
**MFAP3L**	p.D106N	1	3							■														Missense_Mutation
**MFGE8**	p.C335G	1	8				■																	Missense_Mutation
**MFSD1**	p.K215I	1	6					■																Missense_Mutation
**MFSD7**	p.V141G	1	3						■															Missense_Mutation
**MFSD7**	p.G293V	1	0								■													Missense_Mutation
**MFSD8**	p.V501A	1	0													■								Missense_Mutation
**MGA**	p.G1449V	3	8	■															■	■				Missense_Mutation
**MGA**	p.R2566S	1	6					■																Missense_Mutation
**MGA**	p.48_49insL	1	0															■						In_Frame_Ins
**MGAM**	p.I450T	1	7			■																		Missense_Mutation
**MIA3**	NULLL	2	1														■	■						Splice_Site
**MICAL3**	p.D762N	1	8		■																			Missense_Mutation
**MICAL3**	p.Q728R	1	7													■								Missense_Mutation
**MICAL3**	p.R1004R	1	0									■												Silent
**MIEF2**	p.L94V	1	0						■															Missense_Mutation
**MKI67**	p.N356H	1	3													■								Missense_Mutation
**MKI67**	p.E1644*	1	1													■								Nonsense_Mutation
**MLC1**	p.S229L	1	8						■															Missense_Mutation
**MLF2**	p.R166H	1	7								■													Missense_Mutation
**MLLT1**	p.P476P	1	0												■									Silent
**MLLT3**	p.D365E	1	4		■																			Missense_Mutation
**MLLT4**	p.I72I	1	0													■								Silent
**MLPH**	p.R375*	1	1					■																Nonsense_Mutation
**MMP24**	p.N570S	1	1							■														Missense_Mutation
**MN1**	p.Q540fs	1	0																	■				Frame_Shift_Ins
**MON1B**	p.T436T	3	0	■															■	■				Silent
**MORC2**	p.R687R	1	0					■																Silent
**MORN5**	p.H151R	3	4	■															■	■				Missense_Mutation
**MOS**	p.I117L	1	5							■														Missense_Mutation
**MOV10**	p.R510R	1	0									■												Silent
**MOV10**	p.L573L	1	0													■								Silent
**MOXD1**	p.N596K	3	0	■															■	■				Missense_Mutation
**MPDZ**	p.H254R	1	6				■																	Missense_Mutation
**MPDZ**	p.K1085T	1	3								■													Splice_Site
**MRGPRG**	p.L59L	1	0																	■				Silent

**MRPL11**	p.E107E	1	0				■																	Silent
**MRPL39**	p.E137K	1	4							■														Missense_Mutation
**MS4A5**	p.QC198fs	1	0				■																	Frame_Shift_Del
**MSH3**	p.F198L	1	2				■																	Missense_Mutation
**MSL3**	p.Q281*	2	3						■					■										Nonsense_Mutation
**MSLNL**	p.H173Q	1	0						■															Missense_Mutation
**MSLNL**	p.T172K	1	0						■															Missense_Mutation
**MSLNL**	p.H167Q	1	0							■														Missense_Mutation
**MTHFD1**	p.F354C	1	1								■													Missense_Mutation
**MTHFD2**	p.V57G	1	6					■																Missense_Mutation
**MTM1**	p.F513L	1	4													■								Missense_Mutation
**MTMR7**	p.D92V	1	6				■																	Missense_Mutation
**MTMR8**	p.L292M	1	2				■																	Missense_Mutation
**MTO1**	p.S60A	1	1			■																		Missense_Mutation
**MTOR**	p.Q1959R	1	4								■													Missense_Mutation
**MTOR**	p.T2068T	1	0							■														Silent
**MTOR**	p.G1407G	2	0			■				■														Silent
**MUC12**	p.A1914S	1	1												■									Missense_Mutation
**MUC12**	p.V3021I	1	1			■																		Missense_Mutation
**MUC12**	p.H3661D	1	1			■																		Missense_Mutation
**MUC12**	p.P4341Q	1	1							■														Missense_Mutation
**MUC12**	p.G4412C	1	1				■																	Missense_Mutation
**MUC12**	p.A4706D	1	1																■					Missense_Mutation
**MUC12**	p.F460F	1	0					■																Silent
**MUC12**	p.T1710T	1	0							■														Silent
**MUC12**	p.D2116D	1	0						■															Silent
**MUC12**	p.S3662S	1	0			■																		Silent
**MUC12**	p.T3672T	1	0						■															Silent
**MUC13**	p.I81F	1	1	■																				Missense_Mutation
**MUC13**	p.I73I	1	0						■															Silent
**MUC16**	p.T12395A	1	5							■														Missense_Mutation
**MUC16**	p.P11039S	1	4				■																	Missense_Mutation
**MUC16**	p.S5982F	1	3													■								Missense_Mutation
**MUC16**	p.T2552P	1	3					■																Missense_Mutation
**MUC16**	p.A10910S	1	2			■																		Missense_Mutation
**MUC16**	p.S11044P	1	1				■																	Missense_Mutation
**MUC16**	p.V11041M	1	1										■											Missense_Mutation
**MUC16**	p.P11013L	1	1				■																	Missense_Mutation
**MUC16**	p.A10993T	1	1														■							Missense_Mutation
**MUC16**	p.G10974S	1	1									■												Missense_Mutation
**MUC16**	p.H6679Y	1	1									■												Missense_Mutation
**MUC16**	p.S882S	1	0													■								Silent
**MUC17**	p.K2584M	1	3										■											Missense_Mutation
**MUC17**	p.T2802I	2	3	■															■					Missense_Mutation
**MUC17**	p.A3024V	1	3										■											Missense_Mutation
**MUC17**	p.P3287S	1	3												■									Missense_Mutation
**MUC17**	p.T3453A	2	3										■					■						Missense_Mutation
**MUC17**	p.P1220L	1	2												■									Missense_Mutation
**MUC17**	p.L2703P	1	2										■											Missense_Mutation
**MUC17**	p.L2704V	1	2										■											Missense_Mutation
**MUC17**	p.L2934F	1	2								■													Missense_Mutation
**MUC17**	p.P3233T	1	2															■						Missense_Mutation
**MUC17**	p.Y3335S	1	2										■											Missense_Mutation
**MUC17**	p.I3389M	1	2				■																	Missense_Mutation
**MUC17**	p.L3448M	3	2	■														■	■					Missense_Mutation
**MUC17**	p.L388P	4	1	■								■			■				■					Missense_Mutation
**MUC17**	p.N397S	2	1										■	■										Missense_Mutation

**MUC17**	p.T435P	1	1	Missense_Mutation
**MUC17**	p.K480R	1	1	Missense_Mutation
**MUC17**	p.P494T	1	1	Missense_Mutation
**MUC17**	p.I560M	1	1	Missense_Mutation
**MUC17**	p.F600S	3	1	Missense_Mutation
**MUC17**	p.A784V	1	1	Missense_Mutation
**MUC17**	p.C786S	1	1	Missense_Mutation
**MUC17**	p.M870I	1	1	Missense_Mutation
**MUC17**	p.P871T	1	1	Missense_Mutation
**MUC17**	p.N1591S	1	1	Missense_Mutation
**MUC17**	p.L1632P	1	1	Missense_Mutation
**MUC17**	p.S1734A	2	1	Missense_Mutation
**MUC17**	p.A2363V	1	1	Missense_Mutation
**MUC17**	p.Y2372S	1	1	Missense_Mutation
**MUC17**	p.S2373T	1	1	Missense_Mutation
**MUC17**	p.D2384G	1	1	Missense_Mutation
**MUC17**	p.M2387I	1	1	Missense_Mutation
**MUC17**	p.Y2392S	1	1	Missense_Mutation
**MUC17**	p.V2402M	1	1	Missense_Mutation
**MUC17**	p.M2643T	1	1	Missense_Mutation
**MUC17**	p.T2721I	1	1	Missense_Mutation
**MUC17**	p.I2898T	2	1	Missense_Mutation
**MUC17**	p.I2933M	1	1	Missense_Mutation
**MUC17**	p.L2934P	1	1	Missense_Mutation
**MUC17**	p.G2983S	1	1	Missense_Mutation
**MUC17**	p.R2985G	1	1	Missense_Mutation
**MUC17**	p.A3294V	1	1	Missense_Mutation
**MUC17**	p.A3306V	1	1	Missense_Mutation
**MUC17**	p.Y3315S	1	1	Missense_Mutation
**MUC17**	p.I3442T	1	1	Missense_Mutation
**MUC17**	p.S431S	1	0	Silent
**MUC17**	p.S668S	1	0	Silent
**MUC17**	p.T790T	1	0	Silent
**MUC17**	p.T966T	1	0	Silent
**MUC17**	p.A1067A	1	0	Silent
**MUC17**	p.V2369V	1	0	Silent
**MUC17**	p.T2385T	2	0	Silent
**MUC17**	p.L2399L	1	0	Silent
**MUC17**	p.T2593T	1	0	Silent
**MUC17**	p.S2924S	1	0	Silent
**MUC17**	p.S3045S	1	0	Silent
**MUC2**	p.T1830T	2	0	Silent
**MUC2**	p.Q1860L	1	0	Missense_Mutation
**MUC2**	p.*2818G	1	0	Nonstop_Mutation
**MUC4**	p.R5343H	1	2	Missense_Mutation
**MUC4**	p.T3067N	1	2	Missense_Mutation
**MUC4**	p.G2204G	1	0	Silent
**MUC5B**	p.F3794Y	1	3	Missense_Mutation
**MUC5B**	p.G4502V	1	3	Missense_Mutation
**MUC5B**	p.G4701V	1	3	Missense_Mutation
**MUC5B**	p.I3797N	2	2	Missense_Mutation
**MUC5B**	p.L3802Q	1	2	Missense_Mutation
**MUC5B**	p.P4440A	1	2	Missense_Mutation
**MUC5B**	p.T4627S	1	1	Missense_Mutation
**MUC5B**	p.T3143T	1	0	Silent
**MUC5B**	p.P4440P	1	0	Silent
**MUC5B**	p.T4685T	1	0	Silent

**MUC6**	p.T1687T	1	0									■												Silent
**MUC7**	p.T175S	1	1		■																			Missense_Mutation
**MUC7**	p.V260D	1	1													■								Missense_Mutation
**MUC7**	p.P295P	2	0	■						■														Silent
**MUM1**	p.T213I	1	1		■																			Missense_Mutation
**MVB12A**	p.S175G	1	0			■																		Missense_Mutation
**MVK**	p.G47E	1	5							■														Missense_Mutation
**MX2**	p.I433I	1	0													■								Silent
**MXI1**	p.R202Q	1	5				■																	Missense_Mutation
**MXRA5**	p.G672V	1	1			■																		Missense_Mutation
**MYADM**	p.F94C	2	4				■		■															Missense_Mutation
**MYADML2**	p.P264L	3	6	■															■	■				Missense_Mutation
**MYADML2**	p.N266K	1	0						■															Missense_Mutation
**MYBBP1A**	p.P1245P	1	0		■																			Silent
**MYBPC1**	p.R1120C	1	7													■								Missense_Mutation
**MYBPC3**	p.D770A	1	5					■																Splice_Site
**MYC**	p.L61Q	1	6							■														Missense_Mutation
**MYCBP2**	p.F1077L	1	5													■								Missense_Mutation
**MYCBPAP**	p.D739H	1	3							■														Missense_Mutation
**MYF6**	p.D93A	1	8		■																			Missense_Mutation
**MYH11**	p.D1382Y	1	8				■																	Missense_Mutation
**MYH14**	p.R776R	1	0			■																		Silent
**MYH15**	p.E1368A	1	6					■																Missense_Mutation
**MYH4**	p.E984E	1	0				■																	Silent
**MYH7**	p.M1840I	1	1							■														Missense_Mutation
**MYL1**	p.E169G	1	8			■																		Missense_Mutation
**MYLK**	p.L1311P	1	3													■								Missense_Mutation
**MYLK**	p.L1295L	1	0													■								Silent
**MYLK2**	p.I424N	3	7	■															■	■				Missense_Mutation
**MYO10**	p.R239T	2	4	■															■					Missense_Mutation
**MYO16**	p.S462L	1	3							■														Missense_Mutation
**MYO1B**	p.Y357Y	3	0	■															■	■				Silent
**MYO1E**	p.V59V	1	0							■														Silent
**MYO1F**	p.E1069D	1	1										■											Missense_Mutation
**MYO3A**	p.T1417A	1	0					■																Missense_Mutation
**MYO3B**	p.I162N	1	8													■								Missense_Mutation
**MYO5C**	p.R39*	1	0			■																		Nonsense_Mutation
**MYO7A**	p.L1412V	1	5			■																		Missense_Mutation
**MYO9B**	p.S1645L	1	6			■																		Missense_Mutation
**MYOM2**	p.A1446G	3	0	■		■													■					Missense_Mutation
**MYRF**	p.F387L	1	7							■														Missense_Mutation
**MYT1**	p.E280E	2	0	■															■					Silent
**MYT1L**	p.R558R	1	0												■									Silent
**MYZAP**	p.Q382E	3	5	■															■	■				Missense_Mutation
**MYZAP**	p.P63P	3	0	■															■	■				Silent
**MZT1**	p.L28M	1	5			■																		Missense_Mutation
**N4BP3**	p.I377I	1	0		■																			Silent
**NADK**	p.S438S	1	0							■														Silent
**NAF1**	p.G24G	1	0																	■				Silent
**NAGLU**	p.V341V	1	0			■																		Splice_Site
**NAGPA**	p.G338G	1	0															■						Silent
**NARG2**	p.T753A	3	4	■															■	■				Missense_Mutation
**NAT1**	p.S219N	1	0													■								Missense_Mutation
**NBEA**	p.R1990K	1	7													■								Missense_Mutation
**NBN**	p.L708I	2	7						■					■										Missense_Mutation
**NBPF1**	p.V1115M	2	1											■	■									Missense_Mutation
**NBPF1**	p.R1098G	1	1				■																	Missense_Mutation

**NBPF1**	p.L648V	2	1				Missense_Mutation
**NBPF1**	p.D1019D	1	0				Silent
**NBPF10**	p.A101T	1	1				Missense_Mutation
**NBPF10**	p.D648G	1	1				Missense_Mutation
**NBPF12**	p.C42S	4	2				Missense_Mutation
**NBPF14**	p.E475G	1	4				Missense_Mutation
**NBPF14**	p.C550C	1	0				Silent
**NBPF14**	p.E475E	1	0				Silent
**NBPF16**	p.Y519F	1	3				Missense_Mutation
**NCAPD2**	p.L128L	3	0				Silent
**NCAPH2**	p.A270V	1	1				Missense_Mutation
**NCKAP1**	NULLL	1	0				Splice_Site
**NCKAP5**	p.S399G	2	2				Missense_Mutation
**NCKAP5**	p.P1422P	1	0				Silent
**NCKAP5L**	p.K910K	1	1				Silent
**NCL**	p.L309L	1	0				Silent
**NCOA6**	p.N1108K	1	2				Missense_Mutation
**NCOA6**	p.P1185P	1	0				Silent
**NCOR1**	p.S2259S	1	0				Silent
**NCOR2**	p.Q2285R	1	7				Missense_Mutation
**NCR1**	p.Q165R	2	0				Missense_Mutation
**NDN**	p.L140I	3	0				Missense_Mutation
**NDST1**	p.V441V	1	0				Silent
**NDST3**	p.Q870Q	1	0				Silent
**NDUFA10**	p.T189A	1	1				Missense_Mutation
**NDUFAF2**	p.F138F	1	0				Silent
**NDUFAF5**	p.S31C	1	4				Missense_Mutation
**NDUFB2**	NULLL	1	0				De_novo_Start_OutOfFrame
**NDUFB5**	p.I80F	1	7				Missense_Mutation
**NDUFS8**	p.E63A	1	7				Missense_Mutation
**NEB**	p.S5526S	2	0				Silent
**NEB**	p.P1695P	1	0				Silent
**NEDD9**	p.R765R	1	0				Silent
**NEFH**	p.S586S	1	0				Silent
**NEIL3**	p.K195R	1	5				Missense_Mutation
**NEK1**	p.-461fs	2	1				Frame_Shift_Ins
**NELL2**	p.Q260Q	1	1				Silent
**NEMF**	p.A175V	1	0				Missense_Mutation
**NES**	p.L1053R	1	5				Missense_Mutation
**NEU3**	p.F220L	1	1				Missense_Mutation
**NEUROD2**	p.L121R	1	4				Missense_Mutation
**NEUROG2**	p.L201R	1	4				Missense_Mutation
**NF1**	p.K63N	1	7				Missense_Mutation
**NFASC**	p.A389A	1	1				Silent
**NFE2L3**	p.L574L	1	0				Silent
**NFIA**	p.P154A	3	4				Missense_Mutation
**NFIL3**	p.T199T	2	0				Silent
**NFKBIA**	p.P56L	3	1				Missense_Mutation
**NFKBIE**	p.R49R	1	0				Silent
**NFKBIZ**	p.S332*	1	1				Nonsense_Mutation
**NFS1**	p.M247T	1	5				Missense_Mutation
**NFX1**	p.G266D	1	2				Missense_Mutation
**NFXL1**	p.W359*	1	2				Nonsense_Mutation
**NFYC**	p.T409A	1	3				Missense_Mutation
**NHS**	p.Q806Q	2	0				Silent
**NIN**	p.N510K	1	7				Missense_Mutation
**NIN**	p.F1226V	1	4				Missense_Mutation

**NIPBL**	p.Q143E	1	5													■								Missense_Mutation
**NIPBL**	p.D719E	1	2			■																		Missense_Mutation
**NIPBL**	p.D730E	1	2	■																				Missense_Mutation
**NIPBL**	p.I1317I	1	0										■											Silent
**NIT1**	p.Y300N	1	4															■						Missense_Mutation
**NKRF**	p.A544T	1	3							■														Missense_Mutation
**NKRF**	p.A334T	1	0		■																			Missense_Mutation
**NKTR**	p.TK454fs	1	1		■																			Frame_Shift_Del
**NKTR**	p.N836D	2	1														■	■						Missense_Mutation
**NKX3-2**	p.R257L	1	8							■														Missense_Mutation
**NLGN1**	p.S143L	1	2							■														Missense_Mutation
**NLGN4X**	p.Q714Q	1	0													■								Silent
**NLRC5**	p.K31R	1	3					■																Missense_Mutation
**NLRP1**	p.L248Q	1	2									■												Missense_Mutation
**NLRP10**	p.D249V	1	8					■																Missense_Mutation
**NLRP12**	p.A535P	1	2													■								Missense_Mutation
**NLRP9**	p.C771C	1	0					■																Silent
**NLRX1**	p.A548A	1	0									■												Silent
**NME8**	p.F200L	1	5													■								Missense_Mutation
**NMI**	p.E240Q	1	1							■														Missense_Mutation
**NMRAL1**	p.G17A	2	8														■	■						Missense_Mutation
**NNT**	p.E904D	1	6													■								Missense_Mutation
**NOC2L**	p.F593F	1	0			■																		Silent
**NOD1**	p.V98M	1	2							■														Missense_Mutation
**NOD2**	p.R459C	3	1	■															■	■				Missense_Mutation
**NOL12**	p.T124P	1	1				■																	Missense_Mutation
**NOL4**	p.D85E	1	4							■														Missense_Mutation
**NOM1**	p.V784V	1	0					■																Silent
**NOMO3**	p.V623G	1	4							■														Missense_Mutation
**NOMO3**	p.C594C	1	0					■																Silent
**NOP58**	p.K518N	2	6						■					■										Missense_Mutation
**NOS1**	p.E333Q	1	2							■														Missense_Mutation
**NOS1**	p.P128A	3	0	■															■	■				Missense_Mutation
**NOS3**	p.M207I	3	6	■															■	■				Missense_Mutation
**NOS3**	p.Q1038H	1	2														■							Missense_Mutation
**NOTCH1**	p.G774A	1	7								■													Missense_Mutation
**NOTCH2NL**	p.D61H	1	2																	■				Missense_Mutation
**NOTCH4**	p.A1496V	1	0							■														Missense_Mutation
**NOTCH4**	p.G682G	1	0							■														Silent
**NPAS3**	p.S160S	2	0														■	■						Silent
**NPHP4**	p.L167F	1	6									■												Missense_Mutation
**NPHS1**	p.Q746H	1	2			■																		Missense_Mutation
**NPIPB11**	p.G715V	2	0	■															■					Missense_Mutation
**NPIPB11**	p.G505V	2	0		■												■							Missense_Mutation
**NPIPB11**	p.G505W	1	0														■							Missense_Mutation
**NPIPB11**	p.A469A	2	0								■					■								Silent
**NPIPB5**	p.P524T	2	4	■															■					Missense_Mutation
**NPIPB5**	p.A545A	2	0						■			■												Silent
**NPIPB6**	p.I300L	1	0							■														Missense_Mutation
**NPIPB7**	p.P312P	2	0	■															■					Silent
**NPIPB9**	p.M1T	2	2														■	■						Start_Codon_SNP
**NPTN**	p.W153C	1	7										■											Missense_Mutation
**NPY4R**	p.P353S	1	5														■							Missense_Mutation
**NR1H3**	p.L113L	1	0													■								Silent
**NR1H4**	p.F151S	1	8									■												Missense_Mutation
**NR1H4**	p.R227H	1	1				■																	Missense_Mutation
**NR4A2**	p.P108P	3	0	■															■	■				Silent

**NRG1**	p.V240G	1	4				■																	Missense_Mutation
**NRK**	p.A1095A	1	0		■																			Silent
**NRXN2**	p.L420R	2	7	■															■					Missense_Mutation
**NSMCE4A**	p.R300H	1	7			■																		Missense_Mutation
**NT5C1A**	p.D213D	3	0	■															■	■				Silent
**NTF3**	p.Q73R	1	1		■																			Missense_Mutation
**NTPCR**	p.Q131Q	1	0													■								Silent
**NTRK2**	p.E572K	1	7													■								Missense_Mutation
**NTRK3**	p.L10L	1	0					■																Splice_Site
**NUBP2**	p.L157L	1	0			■																		Silent
**NUDT17**	p.L197L	1	1		■																			Silent
**NUDT4**	NULLL	1	1							■														Splice_Site
**NUF2**	p.Q237E	1	0							■														Missense_Mutation
**NUP107**	p.A456G	2	1														■	■						Missense_Mutation
**NUP153**	p.A683T	1	3				■																	Missense_Mutation
**NUP50**	p.V34A	3	5	■															■	■				Missense_Mutation
**NUPR1**	p.S76R	1	5						■															Missense_Mutation
**NUTM2A**	p.A619T	1	0					■																Missense_Mutation
**NUTM2D**	p.L755L	1	0															■						Silent
**NUTM2E**	p.I566T	1	1							■														Missense_Mutation
**NUTM2F**	p.S550C	2	1	■															■					Missense_Mutation
**NVL**	p.D427N	1	8													■								Missense_Mutation
**NXF3**	p.G57R	1	2			■																		Missense_Mutation
**NXPE2**	p.G173D	1	8					■																Missense_Mutation
**NYNRIN**	p.L1362R	1	3				■																	Missense_Mutation
**NYNRIN**	p.T1756T	1	0																	■				Silent
**OBSCN**	p.D4735H	1	3								■													Missense_Mutation
**ODF2**	p.L294L	1	0							■														Silent
**OLFM4**	p.N366D	1	0													■								Missense_Mutation
**OLFML2A**	p.K261N	2	4						■					■										Missense_Mutation
**OLIG1**	p.G230A	1	2																	■				Missense_Mutation
**OMA1**	p.V47I	3	0	■															■	■				Missense_Mutation
**ONECUT3**	p.A91E	1	4																	■				Missense_Mutation
**OR10A3**	p.T258T	1	0													■								Silent
**OR10G6**	p.R282Q	1	0							■														Missense_Mutation
**OR10G8**	p.L42L	1	0				■																	Silent
**OR10R2**	p.L182V	1	2													■								Missense_Mutation
**OR11G2**	p.W193C	1	2							■														Missense_Mutation
**OR14A16**	p.R136K	2	0														■	■						Missense_Mutation
**OR1J1**	p.G96R	3	4	■															■	■				Missense_Mutation
**OR2B11**	p.A259V	1	3													■								Missense_Mutation
**OR2F2**	p.G233V	2	7														■	■						Missense_Mutation
**OR2G2**	p.N198K	1	5								■													Missense_Mutation
**OR2J1**	p.L115L	1	0															■						Silent
**OR2L13**	p.HS127fs	2	0						■					■										Frame_Shift_Del
**OR2L3**	p.F101L	1	3										■											Missense_Mutation
**OR2L3**	p.L26L	1	0										■											Silent
**OR2S2**	p.FS95fs	1	1		■																			Frame_Shift_Del
**OR4A5**	p.K302M	1	0								■													Missense_Mutation
**OR4C12**	p.M21L	1	0													■								Missense_Mutation
**OR4C15**	p.Y113Y	1	0				■																	Silent
**OR4K13**	p.S53S	1	0		■																			Silent
**OR4M2**	p.A238A	1	0		■																			Silent
**OR4S1**	p.A199V	1	1				■																	Missense_Mutation
**OR4X1**	p.G150A	1	4									■												Missense_Mutation
**OR51A7**	p.L148V	1	0													■								Missense_Mutation
**OR51E1**	p.F28V	1	1								■													Missense_Mutation

**OR51F2**	p.R237L	3	2	■															■	■				Missense_Mutation
**OR52D1**	p.M1T	2	4														■	■						Start_Codon_SNP
**OR52E4**	p.T53S	1	0					■																Missense_Mutation
**OR52E6**	p.Q271E	1	0							■														Missense_Mutation
**OR52M1**	p.-291fs	1	0		■																			Frame_Shift_Ins
**OR52N1**	p.I199I	1	0													■								Silent
**OR56B4**	p.I227L	1	2								■													Missense_Mutation
**OR5F1**	p.T57A	1	5				■																	Missense_Mutation
**OR5K4**	p.V40L	1	1								■													Missense_Mutation
**OR5M3**	p.K193Q	1	5													■								Missense_Mutation
**OR5M8**	p.T145T	1	0							■														Silent
**OR5M9**	p.V153A	1	2													■								Missense_Mutation
**OR6B3**	p.A264A	1	0													■								Silent
**OR6C2**	p.F10C	1	7													■								Missense_Mutation
**OR6C2**	p.A114T	3	1	■															■	■				Missense_Mutation
**OR6F1**	p.F177L	1	7													■								Missense_Mutation
**OR8B2**	p.L63L	1	0													■								Silent
**OR8B3**	p.R165T	1	5													■								Missense_Mutation
**OR8B4**	p.A246A	3	0	■															■	■				Silent
**OR8D2**	p.Y177C	3	6	■															■	■				Missense_Mutation
**OR8D2**	p.S142P	1	5				■																	Missense_Mutation
**OR8G5**	p.S299S	1	0													■								Silent
**OR8H3**	p.S265Y	1	5								■													Missense_Mutation
**OR9Q1**	p.C72Y	1	7			■																		Missense_Mutation
**OSBPL2**	p.V242V	1	0							■														Silent
**OSBPL3**	p.Q455E	1	5													■								Missense_Mutation
**OSBPL7**	p.V580V	1	0									■												Silent
**OSGIN2**	p.R221H	1	2													■								Missense_Mutation
**OSTN**	p.R125T	1	0							■														Missense_Mutation
**OTOG**	p.I1016L	1	1							■														Missense_Mutation
**OTUB2**	p.D197D	1	0				■																	Silent
**OTUD7A**	p.G534G	1	0			■																		Silent
**OVOL1**	p.L90V	1	0				■																	Missense_Mutation
**OXR1**	p.K78N	1	3															■						Missense_Mutation
**OXTR**	p.P95P	1	0													■								Silent
**P2RY2**	p.P366P	1	0					■																Silent
**PABPC4**	p.R41R	1	0							■														Silent
**PACSIN2**	p.A352T	1	0							■														Missense_Mutation
**PADI4**	p.T324N	3	0	■															■	■				Missense_Mutation
**PAFAH1B1**	p.D90A	1	2																	■				Missense_Mutation
**PAIP2B**	p.*124*	1	0		■																			Silent
**PAK1IP1**	p.R383S	1	2													■								Missense_Mutation
**PAK3**	p.R498H	3	6	■															■	■				Missense_Mutation
**PAM**	p.T299K	1	8										■											Missense_Mutation
**PAPLN**	NULLL	1	1					■																Splice_Site
**PAPOLG**	p.V609A	3	6	■															■	■				Missense_Mutation
**PAPPA**	p.F310C	1	1													■								Missense_Mutation
**PAPPA2**	p.S1662Y	1	2													■								Missense_Mutation
**PAPSS1**	p.S150N	1	2					■																Missense_Mutation
**PARD3**	p.P278T	1	2						■															Missense_Mutation
**PARD6B**	p.K95N	1	6				■																	Missense_Mutation
**PARL**	p.SL218fs	2	1														■	■						Frame_Shift_Del
**PARN**	p.E364G	1	5			■																		Missense_Mutation
**PARP2**	p.A141D	1	1							■														Missense_Mutation
**PASD1**	p.S346L	3	0	■															■	■				Missense_Mutation
**PAX3**	p.T258T	1	0				■																	Silent
**PAX4**	p.R12Q	1	8									■												Missense_Mutation

**PAXBP1**	p.G582R	1	1															■						Missense_Mutation
**PCDH10**	p.N570T	1	7	■																				Missense_Mutation
**PCDH12**	p.Q61*	1	1													■								Nonsense_Mutation
**PCDH15**	p.I1492V	1	1														■							Missense_Mutation
**PCDH19**	p.N1134K	2	0						■					■										Missense_Mutation
**PCDH20**	p.E545K	1	4													■								Missense_Mutation
**PCDHA10**	p.E655A	1	4						■															Missense_Mutation
**PCDHA13**	p.S536R	1	1						■															Missense_Mutation
**PCDHA6**	p.P453P	1	0							■														Silent
**PCDHA7**	p.S632N	1	5			■																		Missense_Mutation
**PCDHA7**	p.S668A	1	4						■															Missense_Mutation
**PCDHA7**	p.N487fs	1	0		■																			Frame_Shift_Del
**PCDHB10**	p.N181K	1	0		■																			Missense_Mutation
**PCDHB11**	p.R716T	1	4													■								Missense_Mutation
**PCDHB12**	p.C714C	1	0				■																	Silent
**PCDHB12**	p.L745L	2	0						■					■										Silent
**PCDHB14**	p.S472R	1	4		■																			Missense_Mutation
**PCDHB15**	p.R65W	1	6													■								Missense_Mutation
**PCDHB16**	p.Q69R	1	0									■												Missense_Mutation
**PCDHB2**	p.T426T	1	0															■						Silent
**PCDHB8**	p.R184C	1	6								■													Missense_Mutation
**PCDHGA8**	p.F242C	1	6													■								Missense_Mutation
**PCDHGA9**	p.R227C	1	0													■								Missense_Mutation
**PCF11**	p.P907P	1	0	■																				Silent
**PCLO**	p.T3584A	1	4		■																			Missense_Mutation
**PCNT**	p.L2983R	2	3	■															■					Missense_Mutation
**PCNT**	p.E180D	1	2														■							Missense_Mutation
**PCNT**	p.V135I	3	0	■															■	■				Missense_Mutation
**PCNXL3**	p.P1900S	1	0									■												Missense_Mutation
**PCNXL4**	p.L851V	1	0													■								Missense_Mutation
**PCSK5**	p.L1106I	1	3													■								Missense_Mutation
**PCSK5**	p.KWNGMKW	1	0										■											In_Frame_Del
**PDCD2L**	p.-335fs	3	0	■															■	■				Frame_Shift_Ins
**PDE10A**	p.A411V	1	7															■						Missense_Mutation
**PDE3A**	p.K1070Q	1	4													■								Missense_Mutation
**PDE4DIP**	p.L2170R	2	1						■															Missense_Mutation
**PDE4DIP**	p.E748E	4	0	■													■		■	■				Silent
**PDE4DIP**	p.V296V	1	0																	■				Silent
**PDLIM2**	p.P308A	1	4					■																Missense_Mutation
**PDS5A**	p.E479K	1	2							■														Missense_Mutation
**PDXDC1**	p.V204V	2	0														■	■						Silent
**PDZD2**	p.D1942D	1	0				■																	Silent
**PDZRN4**	p.D133N	1	7				■																	Missense_Mutation
**PDZRN4**	p.L145L	1	0				■																	Silent
**PEAK1**	p.E384E	1	0							■														Silent
**PEG3**	p.M190I	1	0													■								Missense_Mutation
**PER1**	p.S1040S	1	0								■													Silent
**PER1**	p.355_356PD>I	1	0									■												In_Frame_Del
**PER2**	p.G55C	1	6															■						Missense_Mutation
**PER3**	p.S1021S	1	0										■											Silent
**PEX1**	p.G1084E	1	8									■												Missense_Mutation
**PEX1**	p.G1084R	1	8									■												Missense_Mutation
**PEX1**	p.G1084G	1	0									■												Silent
**PEX13**	p.G216E	1	1							■														Missense_Mutation
**PEX14**	p.Q296Q	1	0			■																		Silent
**PEX16**	p.Q320P	1	0	■																				Missense_Mutation
**PEX6**	p.S963R	1	8							■														Missense_Mutation

**PF4V1**	p.L87W	3	6	■															■	■				Missense_Mutation
**PGAM4**	p.S31R	1	6										■											Missense_Mutation
**PGM1**	p.G398R	2	7														■	■						Missense_Mutation
**PGM2L1**	p.D378D	1	0														■							Silent
**PGPEP1L**	p.L42L	1	0		■																			Silent
**PGR**	p.L901P	1	9														■							Missense_Mutation
**PGR**	p.E723V	1	9														■							Missense_Mutation
**PGR**	p.P737A	1	8														■							Missense_Mutation
**PGR**	p.D360H	1	5		■																			Missense_Mutation
**PGR**	p.F303I	1	5														■							Missense_Mutation
**PGR**	p.P415L	1	4														■							Missense_Mutation
**PGR**	p.L511F	1	2														■							Missense_Mutation
**PGR**	p.S273P	1	2														■							Missense_Mutation
**PGR**	NULLL	1	1														■							Splice_Site
**PGR**	NULLL	1	1														■							Splice_Site
**PGR**	p.L568L	1	0														■							Silent
**PGR**	p.R319R	1	0														■							Silent
**PHACTR4**	p.E481Q	1	4													■								Missense_Mutation
**PHC2**	p.L310R	1	2	■																				Missense_Mutation
**PHF14**	p.K505T	1	5			■																		Missense_Mutation
**PHF16**	p.S674F	1	3										■											Missense_Mutation
**PHF20L1**	p.G41D	1	4													■								Missense_Mutation
**PHF3**	p.S680I	1	5													■								Missense_Mutation
**PHLDB2**	p.E13K	1	2									■												Missense_Mutation
**PHLDB3**	p.S5G	3	2	■															■	■				Missense_Mutation
**PHLPP1**	p.Y895C	2	4														■	■						Missense_Mutation
**PHRF1**	p.S48N	2	3														■	■						Missense_Mutation
**PHRF1**	p.P348P	1	0			■																		Silent
**PHTF1**	p.C259F	1	2															■						Missense_Mutation
**PI4KA**	p.T1104T	3	0	■															■	■				Silent
**PIAS4**	p.D82G	1	0				■																	Missense_Mutation
**PIGN**	p.I867T	1	6			■																		Missense_Mutation
**PIGN**	p.S719A	1	0					■																Missense_Mutation
**PIGO**	p.L138L	2	0														■	■						Silent
**PIGR**	p.L299R	1	5															■						Missense_Mutation
**PIGS**	p.A25V	1	1							■														Missense_Mutation
**PIGZ**	p.R360Q	1	0							■														Missense_Mutation
**PIK3AP1**	p.D717A	1	4						■															Missense_Mutation
**PIK3CG**	p.R476H	1	6													■								Missense_Mutation
**PIK3CG**	p.K1099E	1	4														■							Missense_Mutation
**PIK3R3**	p.K191N	1	9										■											Missense_Mutation
**PIK3R6**	NULLL	1	0													■								De_novo_Start_OutOfFrame
**PITPNM2**	p.S521F	1	6				■																	Missense_Mutation
**PIWIL1**	p.P379P	1	0						■															Silent
**PKD1**	p.V838L	1	2				■																	Missense_Mutation
**PKD1L1**	p.F538L	1	4													■								Missense_Mutation
**PKD1L1**	p.D1412N	1	2							■														Missense_Mutation
**PKHD1**	p.L2617F	1	7					■																Missense_Mutation
**PKHD1L1**	p.P3058L	1	8													■								Missense_Mutation
**PKLR**	p.H41H	3	0	■															■	■				Silent
**PKM**	p.Y148C	1	8			■																		Missense_Mutation
**PKNOX1**	p.E75D	1	7												■									Missense_Mutation
**PKNOX2**	p.N283K	1	3													■								Missense_Mutation
**PKP2**	p.R533T	1	7				■																	Missense_Mutation
**PLA2G4C**	p.L60F	3	2	■															■	■				Missense_Mutation
**PLA2R1**	p.R387H	2	1														■	■						Missense_Mutation
**PLB1**	p.Q226Q	3	0	■															■	■				Silent

**PLCB1**	p.L408I	1	2													■								Missense_Mutation
**PLCE1**	p.N588N	1	0		■																			Silent
**PLCG1**	p.T596T	1	0		■																			Silent
**PLCG2**	p.P444P	1	0		■																			Silent
**PLCH2**	p.P1181A	1	0					■																Missense_Mutation
**PLCL1**	p.W660C	1	7															■						Missense_Mutation
**PLCL2**	p.L743L	1	2							■														Silent
**PLEKHA4**	p.R542W	1	4							■														Missense_Mutation
**PLEKHG1**	p.S905R	1	0	■																				Missense_Mutation
**PLEKHG2**	p.A705A	1	0	■																				Silent
**PLEKHG4**	p.D1063A	1	6						■															Missense_Mutation
**PLEKHH1**	p.F1126L	1	7				■																	Missense_Mutation
**PLEKHH1**	p.V530I	1	4			■																		Missense_Mutation
**PLEKHH1**	p.R1288T	1	3					■																Missense_Mutation
**PLEKHH2**	p.S288A	1	3							■														Missense_Mutation
**PLEKHN1**	p.A516T	1	2		■																			Missense_Mutation
**PLIN4**	p.D310E	1	3								■													Missense_Mutation
**PLIN4**	p.V529A	3	1	■								■							■					Missense_Mutation
**PLIN4**	p.L558L	2	0														■	■						Silent
**PLIN4**	p.T543T	2	0	■																				Silent
**PLIN4**	p.T542T	1	0																■					Silent
**PLIN4**	p.S527G	3	0	■								■							■					Missense_Mutation
**PLIN4**	p.V525V	3	0	■								■							■					Silent
**PLIN4**	p.G285G	1	0								■													Silent
**PLIN4**	p.T281S	1	0								■													Missense_Mutation
**PLIN4**	p.T229T	1	0														■							Silent
**PLIN4**	p.E211D	2	0						■								■							Missense_Mutation
**PLIN5**	p.L173R	1	6						■															Missense_Mutation
**PLK3**	p.S171A	1	1							■														Missense_Mutation
**PLS1**	p.R435Q	1	3													■								Missense_Mutation
**PLXNA1**	p.D863G	1	2																	■				Missense_Mutation
**PLXNA3**	p.V41V	1	0													■								Silent
**PLXNB2**	p.S650A	1	1			■																		Missense_Mutation
**PLXNB2**	p.F1047F	1	0					■																Silent
**PLXNB3**	p.F1123V	1	1					■																Missense_Mutation
**PMFBP1**	p.R480K	1	0							■														Missense_Mutation
**PML**	p.V767L	1	0				■																	Missense_Mutation
**PMS2**	p.S621F	1	8													■								Missense_Mutation
**PNCK**	p.K313N	1	6													■								Missense_Mutation
**PNLDC1**	p.440_441insK	1	0		■																			In_Frame_Ins
**PNPLA6**	p.R1051Q	1	6														■							Missense_Mutation
**PNPT1**	p.T78A	1	8				■																	Missense_Mutation
**POC1B-GAL**	p.Q124R	2	1														■	■						Missense_Mutation
**POC5**	p.N43D	1	5			■																		Missense_Mutation
**POFUT1**	p.E176D	1	1		■																			Missense_Mutation
**POGZ**	p.C532C	1	0		■																			Silent
**POLE**	p.S2018R	2	2	■															■					Missense_Mutation
**POLI**	p.M310V	3	5	■															■	■				Missense_Mutation
**POLN**	p.R253H	3	1	■															■	■				Missense_Mutation
**POLQ**	p.Q2333*	1	3										■											Nonsense_Mutation
**POLR2A**	p.G409G	2	0														■	■						Silent
**POLR2A**	p.T1639T	1	0					■																Silent
**POLR2A**	p.P1669P	1	0					■																Silent
**POLR2B**	p.E543K	1	7									■												Missense_Mutation
**POLR3B**	p.AS314del	1	0													■								In_Frame_Del
**POLR3H**	p.G43E	1	7													■								Missense_Mutation
**POM121C**	p.Q704P	2	7	■															■					Missense_Mutation

**PON3**	p.T139I	1	7													■								Missense_Mutation
**POSTN**	p.R463C	1	8													■								Missense_Mutation
**POTEC**	p.C103C	2	0								■	■												Silent
**POTED**	p.H86N	1	0					■																Missense_Mutation
**POTED**	p.N435D	2	0	■															■					Missense_Mutation
**POTEF**	p.G76S	1	2														■							Missense_Mutation
**POTEG**	p.G150G	1	0				■																	Silent
**POTEI**	p.H86N	1	2									■												Missense_Mutation
**POTEM**	p.E384E	1	0									■												Silent
**POTEM**	p.S317S	1	0					■																Silent
**POU3F2**	p.S10N	1	1					■																Missense_Mutation
**POU4F2**	p.-56fs	1	0																	■				Frame_Shift_Ins
**PP2D1**	p.S120Y	1	1													■								Missense_Mutation
**PPAP2C**	p.L56L	1	0		■																			Silent
**PPAPDC1A**	p.R42H	1	4													■								Missense_Mutation
**PPAPDC3**	p.Q27P	1	3									■												Missense_Mutation
**PPBP**	p.E51Q	3	0	■															■	■				Missense_Mutation
**PPFIA2**	p.N1127T	1	7													■								Missense_Mutation
**PPIA**	p.K155Q	1	3											■										Missense_Mutation
**PPIG**	p.G92G	1	0								■													Silent
**PPIP5K1**	p.I1244I	1	0		■																			Silent
**PPL**	p.E1104G	1	6			■																		Missense_Mutation
**PPM1E**	p.I237I	1	0				■																	Silent
**PPM1H**	p.V216G	3	2							■							■	■						Missense_Mutation
**PPM1L**	p.R338fs	1	0															■						Frame_Shift_Del
**PPP1R10**	p.R814R	1	0										■											Silent
**PPP1R10**	p.G812G	1	0										■											Silent
**PPP1R10**	p.P799P	1	0															■						Silent
**PPP1R10**	p.G796G	1	0															■						Silent
**PPP1R10**	p.G795G	1	0															■						Silent
**PPP1R10**	p.G793G	1	0															■						Silent
**PPP1R10**	p.S790G	1	0															■						Missense_Mutation
**PPP1R12C**	p.E506Q	1	0											■										Missense_Mutation
**PPP1R13B**	p.P459L	1	5		■																			Missense_Mutation
**PPP1R15A**	p.A427A	1	0														■							Silent
**PPP1R26**	p.T729T	1	0							■														Silent
**PPP2CA**	p.P291P	1	0								■													Silent
**PPP4R4**	p.K642K	1	0							■														Silent
**PPP6R3**	p.P509T	1	7										■											Missense_Mutation
**PPWD1**	p.K422K	1	0							■														Silent
**PPWD1**	p.H567H	1	0									■												Silent
**PPY**	p.-37fs	1	0				■																	Frame_Shift_Ins
**PRAMEF16**	p.Q365Q	1	0					■																Silent
**PRAMEF6**	p.R193C	1	1					■																Missense_Mutation
**PRB1**	p.S40T	1	0														■							Missense_Mutation
**PRB4**	p.S178T	1	1								■													Missense_Mutation
**PRDM9**	p.R867R	1	0				■																	Silent
**PREB**	p.Q254R	3	0	■															■	■				Missense_Mutation
**PRG4**	p.A609A	1	0													■								Silent
**PRG4**	p.A671A	1	0					■																Silent
**PRG4**	p.P836P	1	0			■																		Silent
**PRH2**	p.P140P	2	1	■															■					Silent
**PRH2**	p.P143P	2	0	■															■					Silent
**PRICKLE2**	p.G443G	2	0						■					■										Silent
**PRKD1**	p.T833T	1	0					■																Silent
**PRKD3**	p.D859N	1	7							■														Missense_Mutation
**PROM1**	p.A649S	1	6													■								Missense_Mutation

**PRR14L**	p.S1843N	3	3	■															■	■				Missense_Mutation
**PRR14L**	p.P2088P	1	0										■											Silent
**PRR19**	p.P351P	1	0		■																			Silent
**PRR23A**	p.A56T	1	1		■																			Missense_Mutation
**PRRT3**	p.S383R	1	3						■															Missense_Mutation
**PRSS22**	p.P80A	1	1			■																		Missense_Mutation
**PRSS3**	p.D77D	2	0		■						■													Silent
**PRUNE2**	p.T623I	1	2														■							Missense_Mutation
**PRX**	p.E886E	1	0		■																			Silent
**PRX**	p.P743P	1	0				■																	Silent
**PRX**	p.I727M	1	0				■																	Missense_Mutation
**PRX**	p.K630N	1	0		■																			Missense_Mutation
**PSD4**	p.G69R	1	0		■																			Missense_Mutation
**PSD4**	p.S449A	2	0														■	■						Missense_Mutation
**PSD4**	p.P506S	3	0	■															■	■				Missense_Mutation
**PSENEN**	p.G74G	2	0						■					■										Silent
**PSG1**	p.D322N	1	2															■						Missense_Mutation
**PSG3**	p.G48A	1	5							■														Missense_Mutation
**PSG5**	p.K285R	3	0	■															■	■				Missense_Mutation
**PSG5**	p.P147S	1	0							■														Missense_Mutation
**PSG9**	p.L144L	3	0	■															■	■				Splice_Site
**PSMC5**	p.F175F	1	0													■								Silent
**PSME1**	p.-229fs	1	0														■							Frame_Shift_Ins
**PSMF1**	p.Y69C	2	7														■	■						Missense_Mutation
**PSPC1**	p.N450S	1	2								■													Missense_Mutation
**PTAFR**	p.P247T	1	7									■												Missense_Mutation
**PTBP2**	p.R419Q	1	7											■										Missense_Mutation
**PTCH1**	p.K287R	3	3	■															■	■				Missense_Mutation
**PTCHD2**	p.S1348A	1	1				■																	Missense_Mutation
**PTDSS1**	p.D441E	1	2					■																Missense_Mutation
**PTGER3**	p.M285I	2	2														■	■						Missense_Mutation
**PTGFR**	p.I193I	1	0													■								Silent
**PTGR1**	p.L119M	1	6		■																			Missense_Mutation
**PTPN14**	p.N91D	1	4					■																Missense_Mutation
**PTPN14**	p.T59T	1	0				■																	Silent
**PTPN2**	p.D234Y	1	9							■														Missense_Mutation
**PTPRD**	p.R1674H	1	6					■																Missense_Mutation
**PTPRH**	p.G153R	1	2							■														Missense_Mutation
**PTPRH**	p.R380L	1	0							■														Missense_Mutation
**PTPRT**	p.E287A	2	4	■															■					Splice_Site
**PTPRU**	p.R1024W	1	7				■																	Missense_Mutation
**PTRF**	p.Q67R	1	2							■														Missense_Mutation
**PURG**	p.G254G	2	0														■	■						Silent
**PUSL1**	p.K255R	1	1							■														Missense_Mutation
**PVR**	p.T326T	1	0					■																Silent
**PWWP2A**	p.L78L	1	0											■										Silent
**PXMP4**	p.T177P	1	7		■																			Missense_Mutation
**PZP**	p.D515D	3	0	■															■	■				Splice_Site
**QRFPR**	p.L112R	1	6		■																			Missense_Mutation
**QRICH1**	p.Q428R	1	1					■																Missense_Mutation
**QRICH2**	p.E1004K	1	0					■																Missense_Mutation
**QSOX1**	p.L564V	1	1					■																Missense_Mutation
**RAB11FIP1**	p.D973A	1	2													■								Missense_Mutation
**RAB3C**	p.F59L	1	6					■																Missense_Mutation
**RAB3GAP2**	p.D526D	1	0				■																	Silent
**RAB40C**	p.N229K	1	2							■														Missense_Mutation
**RABGAP1**	p.R438I	1	7													■								Missense_Mutation

**RABL6**	p.R399C	1	1																	■				Missense_Mutation
**RAD23A**	p.H192P	1	6														■							Missense_Mutation
**RAD9B**	p.L42L	1	0							■														Silent
**RALY**	p.F24F	1	0							■														Silent
**RANBP17**	p.R957Q	1	2		■																			Missense_Mutation
**RAP1GAP**	p.P558R	1	3							■														Missense_Mutation
**RAPGEFL1**	p.A456A	1	0					■																Silent
**RASA3**	p.F176F	2	0						■					■										Silent
**RASAL1**	p.I371S	1	7													■								Missense_Mutation
**RASAL3**	p.L978L	1	0														■							Silent
**RAX**	p.P89L	1	3																	■				Missense_Mutation
**RB1**	p.-651fs	1	0										■											Frame_Shift_Ins
**RBBP6**	p.P1501P	3	0	■															■	■				Silent
**RBM33**	p.P837A	3	1	■						■									■					Missense_Mutation
**RBM34**	p.Q93Q	1	0									■												Silent
**RBM5**	p.T307P	1	6													■								Missense_Mutation
**RBM6**	p.P794P	1	0										■											Silent
**RBMX**	p.Y241H	1	6										■											Missense_Mutation
**RBMXL1**	p.P97P	1	0									■												Silent
**RBMXL1**	NULLL	1	0				■																	Start_Codon_Ins
**RC3H1**	p.L140V	1	9										■											Missense_Mutation
**RC3H2**	p.N863K	1	5													■								Missense_Mutation
**RCSD1**	p.P285H	1	3					■																Missense_Mutation
**RDH14**	p.L231Q	2	7														■	■						Missense_Mutation
**RDH16**	p.E217fs	1	0			■																		Frame_Shift_Del
**RDH8**	p.N313S	1	1										■											Missense_Mutation
**REG1B**	p.N66H	1	4													■								Missense_Mutation
**RELN**	p.I104T	1	3				■																	Missense_Mutation
**RENBP**	p.A172A	2	0	■															■					Silent
**REPS1**	p.E109Q	1	4							■														Missense_Mutation
**REPS1**	p.S666R	1	3													■								Missense_Mutation
**REPS2**	p.S292T	3	2	■															■	■				Missense_Mutation
**RERE**	NULLL	1	0								■													De_novo_Start_OutOfFrame
**RERGL**	p.Y33H	1	6										■											Missense_Mutation
**REST**	p.I763M	1	1											■										Missense_Mutation
**REV3L**	p.S2073Y	1	1													■								Missense_Mutation
**REV3L**	p.N2672N	1	0			■																		Silent
**REXO1**	NULLL	1	1			■																		Splice_Site
**RFC4**	p.F126V	1	8										■											Missense_Mutation
**RFPL4A**	p.L85V	1	4															■						Missense_Mutation
**RFTN1**	p.*579*	1	0													■								Silent
**RFX1**	NULLL	1	0			■																		Splice_Site
**RFX5**	p.K12N	1	4													■								Missense_Mutation
**RFXANK**	p.R111Q	1	0			■																		Missense_Mutation
**RGL1**	p.S757N	1	4													■								Missense_Mutation
**RGL3**	p.S710N	1	1				■																	Missense_Mutation
**RGP1**	p.T297A	1	2		■																			Missense_Mutation
**RGPD8**	p.R812S	1	0													■								Missense_Mutation
**RGS22**	p.A859S	3	1	■															■	■				Missense_Mutation
**RGS4**	p.L88L	1	0													■								Silent
**RGS7**	p.A159V	1	4				■																	Missense_Mutation
**RGSL1**	p.F107F	1	0							■														Silent
**RHOBTB3**	p.S41N	1	3				■																	Missense_Mutation
**RHOT2**	p.E177E	1	0		■																			Silent
**RICTOR**	p.G403E	1	7													■								Missense_Mutation
**RIF1**	p.N1241T	1	0													■								Missense_Mutation
**RIMS1**	p.S223R	1	6				■																	Missense_Mutation

RIMS3	p.E131K	1	6		■																		Missense_Mutation
RIPPLY3	p.L100V	2	7														■	■						Missense_Mutation
RLN3	p.A42V	1	8					■																Missense_Mutation
RMDN2	p.K90del	1	0							■														In_Frame_Del
RNF103	p.E294K	1	3													■								Missense_Mutation
RNF111	p.P772R	1	6										■											Missense_Mutation
RNF123	p.V770E	1	4		■																			Missense_Mutation
RNF139	p.T442M	1	3							■														Missense_Mutation
RNF141	p.E154Q	1	5										■											Missense_Mutation
RNF17	p.A28T	1	1											■										Missense_Mutation
RNF181	p.R34M	1	3													■								Missense_Mutation
RNF19A	p.A833E	1	4		■																			Missense_Mutation
RNF212	p.L93V	1	2			■																		Missense_Mutation
RNF223	p.P242L	1	3																	■				Missense_Mutation
RNF34	p.S427T	1	0													■								Missense_Mutation
RNF41	p.T155T	2	0														■	■						Silent
RNF43	p.R113Q	1	1													■								Missense_Mutation
ROBO1	p.S860A	1	1				■																	Missense_Mutation
ROBO3	p.A318A	2	0	■																■				Silent
ROR2	p.A793G	1	0						■															Missense_Mutation
ROS1	p.K1533N	1	3													■								Missense_Mutation
RP1	p.L128R	1	5		■																			Missense_Mutation
RP1	p.K2003T	1	4													■								Missense_Mutation
RP11-1055B8.	p.R785Q	1	7																	■				Missense_Mutation
RP11-1055B8.	p.T1948M	1	7					■																Missense_Mutation
RP11-1055B8.	p.K449R	1	2																	■				Missense_Mutation
RP11-1407O1	p.R343H	1	3													■								Missense_Mutation
RP11-166B2.1	p.P301A	1	2					■																Missense_Mutation
RP11-166B2.1	p.P301P	1	0					■																Silent
RP11-219A15.	p.Q561*	1	2		■																			Nonsense_Mutation
RP11-219A15.	p.W36C	1	1				■																	Missense_Mutation
RP1-139D8.6	p.T102T	1	0							■														Silent
RP11-830F9.6	p.P343A	1	3				■																	Missense_Mutation
RP11-830F9.6	p.I345I	1	0					■																Silent
RP11-863K10	p.F176S	3	0	■															■	■				Missense_Mutation
RP1L1	p.A2185A	1	0												■									Silent
RP1L1	p.P2183P	1	0												■									Silent
RP1L1	p.L2177S	1	0											■										Missense_Mutation
RP1L1	p.L2177L	1	0											■										Silent
RP1L1	p.Q2174Q	1	0											■										Silent
RP1L1	p.Q2167P	1	0																■					Missense_Mutation
RP1L1	p.T1892S	1	0														■							Missense_Mutation
RP1L1	p.A1879A	1	0					■																Silent
RPAP1	p.K165T	1	2						■															Missense_Mutation
RPGR	p.I748V	1	0		■																			Splice_Site
RPGRIP1L	p.T1000T	1	0											■										Silent
RPL17-C18or	p.S184S	1	4														■							Silent
RPL18A	p.R74Q	1	1		■																			Missense_Mutation
RPL27A	NULLL	1	0						■															De_novo_Start_OutOfFrame
RPS3A	p.D77Y	1	6													■								Missense_Mutation
RPS6KA5	p.P112L	1	8													■								Missense_Mutation
RPS6KB1	p.N311K	1	2													■								Missense_Mutation
RPTN	p.S304C	2	4				■										■							Missense_Mutation
RPTN	p.S328C	1	3									■												Missense_Mutation
RPTN	p.T430P	1	2																■					Missense_Mutation
RPTN	p.S425Y	1	2															■						Missense_Mutation
RPTN	p.T454P	1	1															■						Missense_Mutation

**RPTN**	p.D443N	2	1														■	■						Missense_Mutation
**RPTN**	p.K765*	1	0							■														Nonsense_Mutation
**RPTN**	p.S753N	2	0			■				■														Missense_Mutation
**RPTN**	p.H750H	2	0										■		■									Silent
**RPTN**	p.H750Q	1	0									■												Missense_Mutation
**RPTN**	p.D736D	1	0			■																		Silent
**RPTN**	p.Q441Q	1	0															■						Silent
**RPTN**	p.Y427Y	1	0															■						Silent
**RPTN**	p.S161T	1	0							■														Missense_Mutation
**RPTN**	p.G147S	1	0											■										Missense_Mutation
**RRBP1**	p.G239G	1	0										■											Silent
**RRP12**	p.S204S	1	0			■																		Silent
**RRP1B**	p.K322R	1	2				■																	Missense_Mutation
**RSAD1**	p.R166W	3	5	■															■	■				Missense_Mutation
**RSAD2**	p.P20S	1	0			■																		Missense_Mutation
**RSBN1L**	p.P77P	1	0	■																				Silent
**RSPH3**	p.R319S	1	5		■																			Missense_Mutation
**RSPH4A**	p.K68R	1	0					■																Missense_Mutation
**RSPRY1**	p.N444N	3	0	■															■	■				Silent
**RTP3**	p.-171fs	1	0								■													Frame_Shift_Ins
**RTTN**	p.L1181L	1	0							■														Silent
**RWDD3**	p.E221Q	1	4							■														Missense_Mutation
**RXFP1**	p.S271G	2	3														■	■						Missense_Mutation
**RYR1**	p.F4104F	1	0													■								Silent
**RYR2**	p.E3900V	1	5				■																	Missense_Mutation
**RYR2**	NULLL	2	1						■					■										Splice_Site
**RYR3**	p.S2844I	2	6														■	■						Missense_Mutation
**S100A9**	p.N53S	3	0	■															■	■				Missense_Mutation
**S100PBP**	p.S68*	1	1					■																Nonsense_Mutation
**S1PR3**	p.N95S	1	6													■								Missense_Mutation
**SAAL1**	p.D384G	2	0														■	■						Missense_Mutation
**SACS**	p.L2960V	1	4													■								Missense_Mutation
**SACS**	p.E2442*	1	2													■								Nonsense_Mutation
**SAE1**	p.S150S	1	0			■																		Silent
**SALL1**	p.S812S	1	0				■																	Silent
**SALL2**	p.F49L	1	3													■								Missense_Mutation
**SAMD3**	p.R226H	2	6														■	■						Missense_Mutation
**SAMD9L**	p.S1453L	1	1													■								Missense_Mutation
**SAP130**	p.S301S	1	0		■																			Silent
**SAP18**	p.M169I	1	2										■											Missense_Mutation
**SAPCD1**	p.R97H	1	0			■																		Missense_Mutation
**SAT1**	p.P66P	1	0				■																	Silent
**SBSPON**	p.Q153H	1	0							■														Missense_Mutation
**SCAF4**	p.E485D	1	3		■																			Missense_Mutation
**SCAPER**	p.K368R	1	3		■																			Missense_Mutation
**SCARA5**	p.I19I	1	0					■																Silent
**SCCPDH**	p.V398L	1	6				■																	Missense_Mutation
**SCEL**	p.E477Q	1	3						■															Missense_Mutation
**SCN1A**	p.R187W	2	8						■					■										Missense_Mutation
**SCN8A**	p.V791V	1	0													■								Silent
**SCNM1**	p.G22fs	1	0		■																			Frame_Shift_Del
**SCNN1G**	p.R563H	1	5								■													Missense_Mutation
**SCNN1G**	p.P594T	1	5					■																Missense_Mutation
**SCRN2**	p.A326P	2	0	■															■					Missense_Mutation
**SCUBE1**	p.R512T	1	3					■																Missense_Mutation
**SCUBE2**	p.K924K	1	0													■								Silent
**SCYL3**	p.G533E	2	2														■	■						Missense_Mutation

**SCYL3**	p.S476N	1	0					■																Missense_Mutation
**SDHC**	p.A27S	3	5	■															■	■				Splice_Site
**SDK1**	p.K2119Q	1	1																	■				Missense_Mutation
**SDK1**	p.N2086N	1	0		■																			Silent
**SEC14L5**	p.S356F	1	5		■																			Missense_Mutation
**SEC14L5**	p.V19I	1	1		■																			Missense_Mutation
**SEC16A**	p.W1698*	1	2							■														Nonsense_Mutation
**SEC24B**	p.T727K	1	1			■																		Missense_Mutation
**SEC24D**	p.D236N	1	7							■														Missense_Mutation
**SECISBP2L**	p.R341I	1	6													■								Missense_Mutation
**SEMA3D**	p.P339P	1	0				■																	Silent
**SEMA3F**	p.L26F	1	1										■											Missense_Mutation
**SEMA3F**	p.D654D	1	0					■																Silent
**SEMA4F**	p.A606V	2	1														■	■						Missense_Mutation
**SEMA6D**	p.N481S	1	0								■													Missense_Mutation
**SENP6**	p.T124T	1	0					■																Silent
**SERBP1**	p.P27P	1	0					■																Silent
**SERGEF**	p.Q105E	1	2							■														Missense_Mutation
**SERGEF**	p.I89I	1	0								■													Silent
**SERHL2**	p.L208P	3	6	■															■	■				Missense_Mutation
**SERPINA11**	p.P52T	1	3			■																		Missense_Mutation
**SERPINA11**	p.V192V	3	0	■															■	■				Silent
**SERPINA9**	p.G5V	1	3		■																			Missense_Mutation
**SERPINB2**	p.K56E	1	2																	■				Missense_Mutation
**SERPINC1**	p.V197I	1	8									■												Missense_Mutation
**SERPINE3**	p.L298L	1	0		■																			Silent
**SERPINH1**	p.S172C	1	8													■								Missense_Mutation
**SERPINI2**	p.K90N	1	4											■										Missense_Mutation
**SERTAD3**	p.I193F	1	2				■																	Missense_Mutation
**SERTAD3**	p.I192I	1	0				■																	Silent
**SESN1**	p.K64K	1	0				■																	Silent
**SESTD1**	p.F383L	1	5													■								Missense_Mutation
**SETD2**	p.D994H	1	5				■																	Missense_Mutation
**SETD2**	p.G1295G	1	0							■														Silent
**SETX**	p.K1612N	1	7													■								Missense_Mutation
**SF3B1**	p.E776G	1	8		■																			Missense_Mutation
**SF3B1**	p.V668V	1	0													■								Silent
**SFRP1**	p.V139E	1	6							■														Missense_Mutation
**SFSWAP**	p.D747V	1	4										■											Missense_Mutation
**SFTPA2**	p.C68C	3	0	■															■	■				Silent
**SFXN2**	p.I89L	1	4		■																			Missense_Mutation
**SGCG**	p.T112P	1	8					■																Missense_Mutation
**SGSM1**	p.D377A	1	7																	■				Missense_Mutation
**SGSM1**	p.V812V	3	0	■															■	■				Silent
**SH2D3C**	p.T469P	1	1									■												Missense_Mutation
**SH3GL2**	p.R184C	1	7													■								Missense_Mutation
**SH3RF3**	p.A257G	1	0						■															Missense_Mutation
**SH3TC2**	p.L917V	1	6													■								Missense_Mutation
**SHC4**	p.G578D	1	7				■																	Missense_Mutation
**SHCBP1**	p.Q627R	3	0	■															■	■				Missense_Mutation
**SHD**	p.P171S	2	7														■	■						Missense_Mutation
**SHD**	p.T22P	2	4	■															■					Missense_Mutation
**SHH**	p.A373V	1	7																	■				Missense_Mutation
**SHISA6**	p.V422G	1	6					■																Missense_Mutation
**SHMT1**	p.I89T	1	6		■																			Missense_Mutation
**SHOC2**	p.T484T	1	0					■																Silent
**SHOX2**	p.A150T	1	5		■																			Missense_Mutation

**SHPK**	p.F98V	1	4				■																	Missense_Mutation
**SHROOM1**	p.A297A	1	0			■																		Silent
**SI**	p.C635*	1	2									■												Nonsense_Mutation
**SIAE**	p.I441S	1	6				■																	Splice_Site
**SIAE**	p.A29S	1	5													■								Missense_Mutation
**SIAH3**	p.R211H	3	6	■															■	■				Missense_Mutation
**SIK3**	p.D240N	1	6													■								Missense_Mutation
**SIMC1**	p.H325Y	3	0		■		■									■								Missense_Mutation
**SIN3B**	p.L759Q	1	0			■																		Missense_Mutation
**SIPA1L3**	p.P1603R	1	4																	■				Missense_Mutation
**SIRPB1**	p.I65N	1	5				■																	Missense_Mutation
**SIX5**	p.G712G	1	0						■															Silent
**SLAMF7**	p.L245L	1	0													■								Silent
**SLC10A2**	p.S128G	1	3					■																Missense_Mutation
**SLC10A2**	p.K63T	1	1													■								Missense_Mutation
**SLC10A5**	p.L247L	1	0							■														Silent
**SLC12A5**	p.S75S	1	0		■																			Silent
**SLC12A7**	p.I644I	1	0											■										Silent
**SLC12A9**	p.P708P	1	0		■																			Silent
**SLC15A4**	p.A36A	1	0																	■				Silent
**SLC15A5**	NULLL	1	1													■								Splice_Site
**SLC16A5**	p.V88G	1	6	■																				Missense_Mutation
**SLC16A7**	p.S252S	1	0							■														Silent
**SLC17A6**	p.L524L	1	0													■								Silent
**SLC17A9**	p.SS424fs	2	0														■	■						Frame_Shift_Del
**SLC17A9**	p.S425S	2	0														■	■						Silent
**SLC19A2**	p.S186S	1	0													■								Silent
**SLC22A10**	p.V337A	1	1							■														Missense_Mutation
**SLC22A14**	p.R40S	1	4							■														Missense_Mutation
**SLC24A1**	p.E492K	1	7				■																	Missense_Mutation
**SLC25A13**	p.D635Y	1	8														■							Missense_Mutation
**SLC25A16**	p.T223T	1	0				■																	Silent
**SLC25A19**	p.F97F	1	0				■																	Silent
**SLC25A47**	p.R122G	1	8					■																Missense_Mutation
**SLC25A48**	p.V116G	1	5							■														Missense_Mutation
**SLC26A8**	p.R952G	1	2				■																	Missense_Mutation
**SLC27A6**	p.E280D	1	1								■													Missense_Mutation
**SLC2A4**	p.G437D	1	3															■						Missense_Mutation
**SLC34A1**	p.N199T	1	7		■																			Missense_Mutation
**SLC36A3**	p.F356S	1	0		■																			Missense_Mutation
**SLC38A4**	p.N276K	1	4		■																			Missense_Mutation
**SLC39A6**	p.C146S	1	3					■																Missense_Mutation
**SLC39A6**	p.F418I	1	2													■								Missense_Mutation
**SLC39A6**	p.L512L	1	0													■								Silent
**SLC41A3**	NULLL	1	2										■											Splice_Site
**SLC43A3**	p.E349K	1	0				■																	Missense_Mutation
**SLC45A4**	p.F71C	1	8	■																				Missense_Mutation
**SLC4A10**	p.E189Q	1	5													■								Missense_Mutation
**SLC4A10**	p.P296P	1	0					■																Silent
**SLC4A11**	p.E669K	1	0		■																			Missense_Mutation
**SLC4A11**	p.N177K	2	0						■					■										Missense_Mutation
**SLC4A5**	p.I619I	1	0				■																	Silent
**SLC4A7**	p.V303V	1	0														■							Silent
**SLC6A15**	p.V53I	1	5							■														Missense_Mutation
**SLC6A15**	p.R408H	1	4								■													Missense_Mutation
**SLC6A15**	p.EE309fs	1	0							■														Frame_Shift_Del
**SLC6A17**	p.S599C	1	7										■											Missense_Mutation

**SLC6A2**	p.A210T	1	6													■								Missense_Mutation
**SLC7A11**	p.I491L	1	2							■														Missense_Mutation
**SLC8A1**	p.V190V	1	0		■																			Silent
**SLC8A2**	p.L28Q	1	0						■															Missense_Mutation
**SLC8B1**	p.D163A	1	4													■								Missense_Mutation
**SLC9A1**	p.N252I	1	7		■																			Missense_Mutation
**SLC9A1**	p.I122I	1	1							■														Silent
**SLC9A5**	p.R669C	1	3		■																			Missense_Mutation
**SLC9A9**	p.Q567E	1	0			■																		Missense_Mutation
**SLCO1A2**	p.G455E	1	7			■																		Missense_Mutation
**SLCO6A1**	p.V202V	1	0					■																Silent
**SLFN12L**	p.I21V	2	1														■	■						Missense_Mutation
**SLIT2**	p.A673V	1	7		■																			Missense_Mutation
**SLIT2**	p.R1309P	1	6									■												Missense_Mutation
**SLIT3**	p.D1202A	1	4								■													Missense_Mutation
**SLITRK5**	p.E55K	1	4													■								Missense_Mutation
**SLITRK5**	p.A886D	1	3									■												Missense_Mutation
**SLTM**	p.D851N	3	5	■															■	■				Missense_Mutation
**SMAD7**	p.R314R	1	0							■														Silent
**SMCP**	p.S29T	1	0					■																Missense_Mutation
**SMCR8**	p.E436Q	1	0													■								Missense_Mutation
**SMEK1**	p.E367Q	1	4							■														Missense_Mutation
**SMEK2**	p.D385G	1	7							■														Missense_Mutation
**SMEK2**	p.E673K	2	5						■					■										Missense_Mutation
**SMG1**	p.H309Y	1	7													■								Missense_Mutation
**SMG1**	p.R305G	1	3													■								Missense_Mutation
**SMIM4**	p.*38L	1	0													■								Nonstop_Mutation
**SMOC2**	p.D341A	2	4	■															■					Missense_Mutation
**SMOX**	p.G491R	1	8									■												Missense_Mutation
**SMPD2**	p.D43H	2	8														■	■						Missense_Mutation
**SMPD4**	p.T840M	1	4																	■				Missense_Mutation
**SMTN**	p.S329A	1	4				■																	Missense_Mutation
**SNAP47**	p.E296K	1	6													■								Missense_Mutation
**SNAP91**	p.E133Q	1	8													■								Missense_Mutation
**SNAPC2**	p.D162E	1	0													■								Missense_Mutation
**SNRNP200**	p.C516S	1	2															■						Missense_Mutation
**SNTB1**	p.R111C	1	7						■															Missense_Mutation
**SNX19**	p.Q881E	1	6													■								Missense_Mutation
**SNX19**	p.E423E	1	0				■																	Silent
**SNX21**	p.L220L	1	0									■												Silent
**SOHLH2**	p.K218R	1	4		■																			Missense_Mutation
**SON**	p.S780S	1	0															■						Silent
**SON**	p.P1712S	2	0														■	■						Missense_Mutation
**SOST**	p.E39A	1	2						■															Missense_Mutation
**SOX9**	p.R254*	1	1		■																			Nonsense_Mutation
**SP9**	p.S325R	1	2	■																				Missense_Mutation
**SPAG6**	p.L408L	2	0														■	■						Silent
**SPANXN2**	p.D140V	1	5			■																		Missense_Mutation
**SPANXN2**	p.E126V	1	5				■																	Missense_Mutation
**SPANXN2**	p.L128L	3	0									■			■				■					Silent
**SPANXN2**	p.L115L	1	0															■						Silent
**SPATA21**	p.S384R	1	5						■															Missense_Mutation
**SPATA22**	p.M188I	1	0		■																			Missense_Mutation
**SPATA3**	p.H21Q	1	0		■																			Missense_Mutation
**SPATA31A1**	p.R1067W	1	2		■																			Missense_Mutation
**SPATA31A2**	p.H1057L	2	2														■	■						Missense_Mutation
**SPATA31A4**	p.S21S	2	0														■	■						Silent

**SPATA6**	p.G39V	1	7				■																	Missense_Mutation
**SPATS2L**	p.P423L	1	6					■																Missense_Mutation
**SPDYE1**	p.P266H	1	3							■														Missense_Mutation
**SPEN**	p.R1411T	1	3							■														Missense_Mutation
**SPEN**	p.P2055P	1	0			■																		Silent
**SPG11**	p.D2015D	3	0	■															■	■				Silent
**SPG7**	p.I743T	1	8		■																			Missense_Mutation
**SPHKAP**	p.G314E	1	0				■																	Missense_Mutation
**SPOCD1**	p.T115P	1	1							■														Missense_Mutation
**SPOCK3**	p.I382I	1	0													■								Silent
**SPPL2C**	p.P643P	1	0															■						Silent
**SPRR2A**	p.S60C	1	2						■															Missense_Mutation
**SPRR3**	p.E111D	1	4										■											Missense_Mutation
**SPRR3**	p.C66*	1	1							■														Nonsense_Mutation
**SPRR3**	p.C74*	2	1	■															■					Nonsense_Mutation
**SPRR3**	p.C74C	1	0									■												Silent
**SPRY2**	p.T113R	2	4														■	■						Missense_Mutation
**SPTA1**	p.R1507P	1	5				■																	Missense_Mutation
**SPTA1**	p.D1379N	3	0	■															■	■				Missense_Mutation
**SPTB**	p.Q1630H	1	7																	■				Missense_Mutation
**SPTB**	p.A2109S	1	0						■															Missense_Mutation
**SPTBN2**	p.E2092K	1	3			■																		Missense_Mutation
**SPTBN5**	p.S1519A	2	2	■															■					Missense_Mutation
**SRBD1**	p.Q216R	2	2														■	■						Splice_Site
**SRCAP**	p.P1872P	2	0		■							■												Silent
**SRGAP1**	p.S999C	1	0				■																	Missense_Mutation
**SRP14**	p.PTTAA124fs	1	0														■							Frame_Shift_Del
**SRP72**	p.H217R	1	0					■																Missense_Mutation
**SRPR**	p.A501V	2	3														■	■						Missense_Mutation
**SRRM1**	p.G437G	3	1	■															■	■				Silent
**SSFA2**	p.N435D	1	5													■								Missense_Mutation
**SSH2**	p.K564N	1	4													■								Missense_Mutation
**SSMEM1**	p.T73T	1	0					■																Silent
**ST18**	p.G772S	1	7		■																			Missense_Mutation
**STAM**	p.Y392H	1	4		■																			Missense_Mutation
**STAM2**	NULLL	1	0		■																			Splice_Site
**STAT1**	p.R420T	1	5										■											Missense_Mutation
**STEAP1B**	p.Y219F	3	5	■															■	■				Missense_Mutation
**STIL**	p.P959P	1	0					■																Silent
**STK10**	p.K446T	1	0						■															Missense_Mutation
**STK32C**	p.I13I	3	0	■															■	■				Silent
**STK36**	p.I121F	1	7			■																		Missense_Mutation
**STK36**	p.G1274S	1	1			■																		Missense_Mutation
**STMN2**	p.I34V	1	1				■																	Missense_Mutation
**STOX2**	p.R316R	3	0	■															■	■				Silent
**STRN3**	p.R359R	1	1													■								Silent
**STX1A**	p.I277I	1	0				■																	Silent
**STXBP5**	p.E216Q	1	2										■											Missense_Mutation
**SUCLA2**	p.G410G	1	0		■																			Splice_Site
**SULF2**	p.KM404fs	1	1		■																			Frame_Shift_Del
**SUPT3H**	p.S89C	1	2													■								Missense_Mutation
**SUSD5**	p.E107K	1	4							■														Missense_Mutation
**SV2B**	p.G247G	1	0								■													Silent
**SWT1**	p.E836E	1	0													■								Splice_Site
**SYCE2**	p.R214T	1	1			■																		Missense_Mutation
**SYCP1**	p.K117T	1	7													■								Missense_Mutation
**SYDE1**	p.D119V	1	3																	■				Missense_Mutation

**SYDE2**	p.S405G	1	0							■														Missense_Mutation
**SYNE1**	p.K35T	1	7							■														Missense_Mutation
**SYNE1**	p.I8482V	1	5		■																			Missense_Mutation
**SYNE1**	p.D4216E	1	2													■								Missense_Mutation
**SYNE1**	p.M4460V	1	1			■																		Missense_Mutation
**SYNE1**	p.E1303*	1	1		■																			Nonsense_Mutation
**SYNE2**	p.I5873M	3	6	■															■	■				Missense_Mutation
**SYNE2**	p.L3182L	2	0														■	■						Silent
**SYNGAP1**	p.S873R	1	6														■							Missense_Mutation
**SYNGAP1**	p.G1060G	1	0			■																		Silent
**SYT3**	p.G205G	1	0								■													Silent
**SYTL2**	p.D295N	3	0	■															■	■				Missense_Mutation
**SYTL5**	p.S142S	1	0			■																		Silent
**SZT2**	p.G2581V	1	5					■																Missense_Mutation
**SZT2**	p.P2262P	1	0		■																			Silent
**TAAR2**	p.I289M	1	5													■								Missense_Mutation
**TACC2**	NULLL	1	2													■								De_novo_Start_OutOfFrame
**TACC3**	p.A232G	1	0								■													Missense_Mutation
**TACO1**	p.D272N	3	4	■															■	■				Missense_Mutation
**TAF4B**	p.L323I	1	6													■								Missense_Mutation
**TALDO1**	p.I66I	1	0		■																			Silent
**TANC1**	p.D685N	3	6	■															■	■				Missense_Mutation
**TANGO2**	p.V47I	1	1		■																			Missense_Mutation
**TAOK2**	p.K26K	1	0			■																		Silent
**TAPT1**	p.Q387H	1	5			■																		Missense_Mutation
**TAPT1**	p.T79T	3	0	■															■	■				Silent
**TAS1R1**	p.E625E	1	1													■								Silent
**TAS1R2**	p.G224G	1	0					■																Silent
**TAS2R16**	p.G33D	3	5	■															■	■				Missense_Mutation
**TAS2R41**	p.S292R	1	0							■														Missense_Mutation
**TAS2R9**	p.K158E	1	0				■																	Missense_Mutation
**TATDN1**	p.K125R	1	1					■																Missense_Mutation
**TBC1D10C**	p.M227K	1	0		■																			Missense_Mutation
**TBC1D16**	p.G268S	3	1	■															■	■				Missense_Mutation
**TBC1D17**	p.P629P	1	0																	■				Silent
**TBC1D25**	p.E271A	1	2							■														Missense_Mutation
**TBC1D2B**	p.A28T	1	0																	■				Missense_Mutation
**TBC1D30**	p.T93P	1	0																	■				Missense_Mutation
**TBC1D9**	p.E559D	1	6											■										Missense_Mutation
**TBCD**	p.E755A	2	0														■	■						Missense_Mutation
**TBCK**	p.Q387*	1	2				■																	Nonsense_Mutation
**TBL1XR1**	p.I78L	1	3													■								Missense_Mutation
**TBP**	p.QQQ92fs	1	0														■							Frame_Shift_Del
**TBR1**	p.F17V	1	4													■								Missense_Mutation
**TBX15**	p.F331F	1	0								■													Silent
**TBX22**	p.F454C	1	3													■								Missense_Mutation
**TCAP**	p.C38Y	1	6							■														Missense_Mutation
**TCEB1**	p.T84S	1	2							■														Missense_Mutation
**TCF12**	p.S673N	1	5				■																	Missense_Mutation
**TCF20**	p.S925fs	1	0							■														Frame_Shift_Del
**TCF4**	p.A482A	3	0	■															■	■				Silent
**TCF7L2**	p.M131L	1	6													■								Missense_Mutation
**TCHH**	p.E1658Q	1	2					■																Missense_Mutation
**TCHH**	p.R1495S	2	2	■															■					Missense_Mutation
**TCHH**	p.L532M	1	2								■													Missense_Mutation
**TCHH**	p.R1657G	1	1					■																Missense_Mutation
**TCHH**	p.S1793P	1	0			■																		Missense_Mutation

**TCHH**	p.D1664E	2	0					■						■										Missense_Mutation
**TCHH**	p.H1663Q	1	0					■																Missense_Mutation
**TCHH**	p.D1600E	1	0						■															Missense_Mutation
**TCHH**	p.M1598L	1	0						■															Missense_Mutation
**TCHHL1**	p.L778I	1	1													■								Missense_Mutation
**TCTN2**	p.H517H	1	0				■																	Silent
**TDRD15**	p.D1611V	1	3							■														Missense_Mutation
**TDRD15**	p.T1725T	1	0								■													Silent
**TEKT4**	p.F56F	1	0													■								Silent
**TENC1**	p.R488W	1	8															■						Missense_Mutation
**TENM2**	p.R2341R	1	0													■								Silent
**TENM3**	p.R898H	1	6				■																	Missense_Mutation
**TENM4**	p.D282D	1	0		■																			Silent
**TEP1**	p.E1915E	1	0			■																		Silent
**TES**	p.L390L	1	0			■																		Silent
**TEX15**	p.S310C	1	3										■											Missense_Mutation
**TFE3**	p.G482A	1	1									■												Missense_Mutation
**TGFB1**	p.R228K	1	0																	■				Missense_Mutation
**TGFBR1**	p.E245Q	1	7									■												Missense_Mutation
**TGFBRAP1**	p.L615L	2	0														■	■						Silent
**TGM3**	p.A104V	1	1									■												Missense_Mutation
**TGM4**	p.Q670Q	1	0													■								Silent
**TGM6**	p.S646R	1	7		■																			Missense_Mutation
**TGOLN2**	p.P203S	1	3														■							Missense_Mutation
**TGOLN2**	p.P67T	2	2	■															■					Missense_Mutation
**THADA**	p.G1535R	1	1		■																			Missense_Mutation
**THADA**	p.H1430R	3	1	■															■	■				Missense_Mutation
**THAP3**	p.S187F	1	4													■								Missense_Mutation
**THBD**	p.H456H	3	0						■					■	■									Silent
**THEMIS**	NULLL	1	1									■												Splice_Site
**THSD7A**	p.D166H	1	2		■																			Missense_Mutation
**TIGD3**	p.L389P	1	0				■																	Missense_Mutation
**TKT**	p.A95V	3	2	■															■	■				Missense_Mutation
**TLCD1**	p.S83S	2	0														■	■						Silent
**TLE4**	p.R116W	1	7															■						Missense_Mutation
**TLR4**	p.E266K	2	3						■					■										Missense_Mutation
**TLR6**	p.K38R	1	1					■																Missense_Mutation
**TLR9**	p.Q722P	1	4												■									Missense_Mutation
**TM2D3**	p.S40Y	1	1				■																	Missense_Mutation
**TM4SF20**	p.L186L	3	0	■															■	■				Silent
**TM4SF4**	p.L10fs	1	0													■								Frame_Shift_Ins
**TM7SF3**	p.S442S	1	0													■								Silent
**TMC5**	p.A58A	1	0			■																		Silent
**TMCC1**	p.F87V	1	3													■								Missense_Mutation
**TMCO2**	p.E32Q	3	1	■															■	■				Missense_Mutation
**TMCO3**	p.A468V	1	1		■																			Missense_Mutation
**TMEFF2**	p.S201S	1	0													■								Silent
**TMEM102**	p.P231S	1	1				■																	Missense_Mutation
**TMEM120B**	p.*340C	1	2									■												Nonstop_Mutation
**TMEM132B**	p.A252V	1	0			■																		Missense_Mutation
**TMEM132D**	p.I545I	1	0		■																			Silent
**TMEM144**	p.S100L	1	5						■															Missense_Mutation
**TMEM145**	p.P67P	1	0				■																	Silent
**TMEM170A**	p.S12A	1	0																	■				Missense_Mutation
**TMEM19**	p.L290L	1	0							■														Silent
**TMEM200A**	p.L478F	3	3	■															■	■				Missense_Mutation
**TMEM200A**	p.S214S	2	0						■					■										Silent

**TMEM209**	p.P127P	1	0			■																		Silent
**TMEM232**	p.Y471C	1	0		■																			Missense_Mutation
**TMEM236**	p.L96L	1	0							■														Silent
**TMEM254**	p.G44R	2	7														■	■						Missense_Mutation
**TMEM45B**	p.A267A	1	0																	■				Silent
**TMEM51**	p.R128S	1	4					■																Missense_Mutation
**TMEM51**	NULLL	1	0				■																	De_novo_Start_OutOfFrame
**TMEM61**	p.D185N	1	0		■																			Missense_Mutation
**TMEM74**	p.E288*	1	1													■								Nonsense_Mutation
**TMEM75**	p.E121Q	1	2													■								Missense_Mutation
**TMF1**	p.I210I	1	0				■																	Silent
**TMSB15B**	p.K20K	1	0					■																Silent
**TMTC1**	p.S369S	2	0														■	■						Silent
**TNFAIP8L3**	p.G2R	1	2				■																	Missense_Mutation
**TNFAIP8L3**	p.N220N	2	0														■	■						Silent
**TNFRSF10B**	p.T408T	2	0														■	■						Silent
**TNFRSF10C**	p.P207T	1	2										■											Missense_Mutation
**TNFRSF17**	p.G98A	1	0					■																Missense_Mutation
**TNFRSF19**	p.P91A	1	4		■																			Missense_Mutation
**TNFRSF9**	p.E247G	1	5					■																Missense_Mutation
**TNK2**	p.S700F	1	2											■										Missense_Mutation
**TNKS**	p.R261R	1	0													■								Silent
**TNKS**	p.A1034A	1	0				■																	Silent
**TNKS1BP1**	p.S1418S	1	0							■														Silent
**TNKS2**	p.V420V	1	0							■														Silent
**TNN**	p.L271P	1	5													■								Missense_Mutation
**TNN**	p.T447T	1	0				■																	Silent
**TNNT2**	p.A281G	1	0																	■				Missense_Mutation
**TNPO3**	p.N98D	1	6					■																Missense_Mutation
**TNR**	p.P1044A	2	7						■					■										Missense_Mutation
**TNRC6A**	p.A541T	1	4					■																Missense_Mutation
**TNS3**	p.S1435T	3	3	■															■	■				Missense_Mutation
**TNXB**	p.L1473Q	1	1							■														Missense_Mutation
**TNXB**	p.P2298P	1	0		■																			Silent
**TNXB**	p.P1567P	2	0	■															■					Silent
**TOE1**	p.R337Q	1	6				■																	Missense_Mutation
**TOM1**	p.S462R	3	6	■												■			■					Missense_Mutation
**TOMM7**	p.S97fs	1	0															■						Splice_Site
**TOP1MT**	p.E176K	1	6									■												Missense_Mutation
**TOPAZ1**	p.G61G	1	0															■						Silent
**TOPBP1**	p.Q920R	1	6							■														Missense_Mutation
**TOPBP1**	p.S1016L	1	1													■								Missense_Mutation
**TOX2**	p.K239Q	1	6										■											Missense_Mutation
**TP63**	p.R97H	1	4				■																	Missense_Mutation
**TPH2**	p.E386*	1	3													■								Nonsense_Mutation
**TPI1**	p.N283S	3	4	■															■	■				Missense_Mutation
**TPO**	p.Q246Q	1	0													■								Silent
**TPR**	p.S573L	2	2						■					■										Missense_Mutation
**TPTE**	p.V281A	1	8				■																	Missense_Mutation
**TPTE**	p.D437V	1	2															■						Missense_Mutation
**TPTE2**	p.V71I	2	2	■															■					Missense_Mutation
**TRABD2A**	p.K48R	2	4														■	■						Missense_Mutation
**TRAK1**	p.L516R	2	3	■															■					Missense_Mutation
**TRAK1**	p.V518L	1	0				■																	Missense_Mutation
**TRAPPC10**	p.E1017K	1	0													■								Missense_Mutation
**TRAPPC11**	p.L1107L	1	1							■														Silent
**TRAPPC2L**	p.R18C	1	8													■								Missense_Mutation

**TRAPPC3L**	p.K46T	1	2													■								Missense_Mutation
**TRAPPC9**	p.V822I	1	0		■																			Missense_Mutation
**TRDMT1**	p.N158fs	3	0	■															■	■				Frame_Shift_Del
**TREML2**	p.S199T	1	0		■																			Missense_Mutation
**TRIM11**	p.I176L	1	1				■																	Missense_Mutation
**TRIM13**	p.I131V	1	2		■																			Missense_Mutation
**TRIM37**	p.P265L	2	8														■	■						Missense_Mutation
**TRIM37**	NULLL	1	1										■											Splice_Site
**TRIM41**	p.V500G	1	7										■				■							Missense_Mutation
**TRIM41**	p.H532P	1	0										■											Missense_Mutation
**TRIM50**	p.A262A	1	0				■																	Silent
**TRIM64C**	p.G423R	1	1														■							Missense_Mutation
**TRIM65**	p.G361D	1	0					■																Missense_Mutation
**TRIOBP**	p.Q297E	3	0	■						■									■					Missense_Mutation
**TRIOBP**	p.I763N	2	0	■															■					Missense_Mutation
**TRIP11**	p.K419Q	1	3					■																Missense_Mutation
**TRIP12**	p.Q889*	1	3													■								Nonsense_Mutation
**TROAP**	p.Y548F	2	1	■															■					Missense_Mutation
**TROAP**	p.P578P	1	0						■															Silent
**TROAP**	p.I595S	2	0		■				■															Missense_Mutation
**TRPA1**	p.L597R	1	1													■								Missense_Mutation
**TRPC7**	p.F476L	1	8					■																Missense_Mutation
**TRPM2**	p.E960K	3	7	■															■	■				Missense_Mutation
**TRPM6**	p.L49S	1	5				■																	Missense_Mutation
**TRPM7**	p.K599N	1	3										■											Missense_Mutation
**TRPM7**	p.R41R	1	0							■														Splice_Site
**TRPM8**	p.N43H	1	2													■								Missense_Mutation
**TRPV1**	p.T336P	1	8				■																	Missense_Mutation
**TRPV1**	p.D565A	1	2							■														Missense_Mutation
**TRPV5**	p.Q310E	1	3		■																			Missense_Mutation
**TRPV6**	p.N640H	1	0						■															Missense_Mutation
**TRRAP**	p.M2307I	1	2								■													Missense_Mutation
**TSC22D1**	p.Q568fs	1	1					■																Frame_Shift_Ins
**TSC22D2**	p.V271A	1	0									■												Missense_Mutation
**TSEN54**	p.L500L	1	0							■														Silent
**TSGA13**	p.L156P	1	4					■																Missense_Mutation
**TSHR**	p.E327K	3	1	■															■	■				Missense_Mutation
**TSHR**	p.P52P	1	0								■													Silent
**TSHZ3**	p.M559I	1	2				■																	Missense_Mutation
**TSHZ3**	p.D778D	2	0														■	■						Silent
**TSKS**	p.L20R	1	2				■																	Missense_Mutation
**TSKS**	p.LQ20fs	1	1				■																	Splice_Site
**TSPAN1**	p.T111T	1	0									■												Silent
**TSPYL6**	p.V35I	2	3														■	■						Missense_Mutation
**TSPYL6**	p.S30I	1	2		■																			Missense_Mutation
**TSR1**	p.T764T	2	0														■	■						Silent
**TSTA3**	p.A106T	1	2					■																Missense_Mutation
**TTC16**	p.R328C	1	8													■								Missense_Mutation
**TTC21A**	p.S269R	1	3													■								Missense_Mutation
**TTC23**	p.G309E	1	0								■													Missense_Mutation
**TTC3**	p.K769N	1	6													■								Missense_Mutation
**TTC39B**	p.V397I	1	1								■													Missense_Mutation
**TTC40**	p.KE1381fs	1	0									■												Frame_Shift_Del
**TTC6**	p.D1317E	1	0										■											Missense_Mutation
**TTC8**	p.E307D	1	4								■													Missense_Mutation
**TTI2**	p.L433V	1	3			■																		Missense_Mutation
**TTLL5**	p.G189A	1	8						■															Missense_Mutation

**TTLL5**	p.R758H	1	8			■																		Missense_Mutation
**TTLL5**	p.M859T	1	1							■														Missense_Mutation
**TTN**	p.G33027S	1	7							■														Missense_Mutation
**TTN**	p.N17941I	1	7				■																	Missense_Mutation
**TTN**	p.A21442T	1	6				■																	Missense_Mutation
**TTN**	p.I5134M	1	6							■														Missense_Mutation
**TTN**	p.I18822T	1	5													■								Missense_Mutation
**TTN**	p.P27436T	1	4													■								Missense_Mutation
**TTN**	p.T14444T	1	0			■																		Silent
**TTN**	p.T13128T	2	0														■	■						Silent
**TTN**	p.P13019P	1	0														■							Silent
**TTN**	p.P12293P	1	0			■																		Silent
**TTN**	p.T11006T	1	0			■																		Silent
**TTN**	p.S5693S	1	0			■																		Silent
**TTN**	p.S5611del	1	0							■														In_Frame_Del
**TTPA**	p.E249*	1	2													■								Nonsense_Mutation
**TUBA1A**	p.H406Y	1	8													■								Missense_Mutation
**TUBA1C**	p.D98D	1	0					■																Silent
**TUBD1**	p.N186S	1	7					■																Missense_Mutation
**TUBE1**	p.R415I	1	6													■								Missense_Mutation
**TUBGCP6**	p.A1658A	1	0					■																Silent
**TUBGCP6**	p.V1229V	1	0											■										Silent
**TUBGCP6**	p.T1221A	3	0	■										■					■					Missense_Mutation
**TUBGCP6**	p.D1150D	2	0	■															■					Silent
**TUBGCP6**	p.R1128R	1	0											■										Silent
**TUBGCP6**	p.V1097V	1	0						■															Silent
**TWIST1**	p.S93fs	1	0																	■				Frame_Shift_Ins
**TXNDC2**	p.S170G	1	0									■												Missense_Mutation
**TXNDC2**	p.D171D	1	0													■								Silent
**TXNDC2**	p.Q271P	1	0									■												Missense_Mutation
**TXNDC5**	p.A357G	1	8				■																	Missense_Mutation
**U2SURP**	p.R251I	1	5													■								Missense_Mutation
**UAP1L1**	p.T422T	3	0	■															■	■				Silent
**UBA3**	p.Y388S	1	2				■																	Missense_Mutation
**UBA7**	p.Q508*	1	1					■																Nonsense_Mutation
**UBAC1**	p.A254A	1	0									■												Silent
**UBBP4**	p.K201Q	1	0							■														Missense_Mutation
**UBBP4**	p.L202L	1	0							■														Silent
**UBBP4**	p.R206G	1	0							■														Missense_Mutation
**UBBP4**	p.R206R	1	0							■														Silent
**UBC**	p.I175I	2	0						■					■										Silent
**UBE2Q2**	p.M210T	1	7				■																	Missense_Mutation
**UBOX5**	p.L102L	1	0				■																	Silent
**UBQLN2**	p.G483G	1	0												■									Silent
**UBR1**	p.A537V	1	2													■								Missense_Mutation
**UBR2**	p.E213Q	1	1													■								Missense_Mutation
**UBR4**	p.T730T	1	0		■																			Silent
**UBTF**	p.T692M	1	2											■										Missense_Mutation
**UCKL1**	p.G87fs	1	0																	■				Splice_Site
**UFC1**	p.D3V	1	7										■											Missense_Mutation
**UGGT2**	p.P303S	1	7		■																			Missense_Mutation
**UGT1A4**	p.A58fs	1	0		■																			Frame_Shift_Del
**UGT2B11**	p.I508T	1	1							■														Missense_Mutation
**UGT2B7**	p.R144S	1	1													■								Missense_Mutation
**UHRF1BP1L**	NULLL	2	0														■	■						Splice_Site
**ULK4**	p.Y931C	1	1		■																			Missense_Mutation
**UNC13C**	p.R1579H	1	2		■																			Missense_Mutation

**UNC45B**	p.G462A	1	6									■												Missense_Mutation
**UNC5A**	p.H362P	1	5									■												Missense_Mutation
**UNC5C**	p.L601L	1	0															■						Silent
**UNC79**	p.H2375R	1	5			■																		Missense_Mutation
**UNC79**	p.H996H	1	0		■																			Silent
**UNC80**	p.K795N	1	4													■								Missense_Mutation
**UPRT**	p.A276A	3	0	■															■	■				Silent
**URB1**	p.R2008W	1	3		■																			Missense_Mutation
**URI1**	p.N399K	1	5								■													Missense_Mutation
**UROD**	p.T49P	1	7						■															Missense_Mutation
**USH1G**	p.L78*	1	2				■																	Nonsense_Mutation
**USH2A**	p.F1110L	1	4					■																Missense_Mutation
**USH2A**	p.N5160S	3	2	■															■	■				Missense_Mutation
**USP1**	p.G11G	1	0		■																			Silent
**USP10**	p.L264L	1	0					■																Silent
**USP13**	p.F741L	2	6														■	■						Missense_Mutation
**USP14**	p.F450S	1	6										■											Missense_Mutation
**USP17L10**	p.S364T	1	1										■											Missense_Mutation
**USP17L10**	p.S336S	1	0															■						Silent
**USP17L13**	p.E422K	1	1							■														Missense_Mutation
**USP17L18**	p.Q405H	2	3													■		■						Missense_Mutation
**USP17L18**	p.Q405L	2	1													■		■						Missense_Mutation
**USP17L2**	p.A161V	1	8							■														Missense_Mutation
**USP17L22**	p.H331Y	1	6							■														Missense_Mutation
**USP17L22**	p.K290K	1	0							■														Silent
**USP20**	p.E838A	1	3									■												Splice_Site
**USP32**	p.G1453D	1	2			■																		Missense_Mutation
**USP32**	p.V620L	1	2													■								Missense_Mutation
**USP35**	p.S907S	1	0		■																			Silent
**USP37**	p.F271L	2	5						■					■										Missense_Mutation
**USP45**	p.P756A	2	5														■	■						Missense_Mutation
**USP49**	p.Q107L	1	1			■																		Missense_Mutation
**USP50**	NULLL	1	0					■																De_novo_Start_InFrame
**USP53**	p.K401E	1	2			■																		Missense_Mutation
**USP54**	p.R1677T	1	0							■														Missense_Mutation
**USP54**	p.S632R	1	0								■													Missense_Mutation
**USP6NL**	p.G556G	1	0		■																			Silent
**UTP20**	p.A205A	3	0	■															■	■				Silent
**UTRN**	p.E2121*	1	2													■								Nonsense_Mutation
**VAV2**	p.H83Q	1	4							■														Missense_Mutation
**VCL**	p.R925C	1	6					■																Missense_Mutation
**VCPKMT**	p.L156L	1	0										■											Silent
**VCX**	p.L154Q	2	2	■															■					Missense_Mutation
**VCX**	p.L164Q	1	2							■														Missense_Mutation
**VCX**	p.L134Q	1	0							■														Missense_Mutation
**VCX3A**	p.S155R	1	0									■												Missense_Mutation
**VEZT**	p.S651S	1	0					■																Silent
**VIPR2**	p.Y323H	1	6			■																		Missense_Mutation
**VIT**	p.K9fs	1	0			■																		Frame_Shift_Del
**VKORC1**	p.R61Q	1	1													■								Missense_Mutation
**VNN3**	p.L125F	1	4							■														Missense_Mutation
**VPS13A**	p.D2592Y	1	7							■			■											Missense_Mutation
**VPS13B**	p.R2126K	1	1							■														Missense_Mutation
**VPS13C**	p.T542T	1	0				■																	Silent
**VPS4A**	p.L12L	3	0	■															■	■				Silent
**VRK3**	p.P102A	2	1						■	■														Missense_Mutation
**VSIG1**	p.S104A	1	3			■																		Missense_Mutation

**VSIG2**	p.S89*	1	1	Nonsense_Mutation
**VWA2**	p.D699D	1	0	Silent
**VWA3A**	p.S195R	1	1	Splice_Site
**VWA3B**	p.K904E	3	4	Missense_Mutation
**VWA5B2**	p.R721H	3	0	Missense_Mutation
**VWDE**	p.K416E	1	1	Missense_Mutation
**VWF**	p.Y1400Y	1	0	Silent
**WASH4P**	p.R141C	1	3	Missense_Mutation
**WBP1**	p.V207A	1	0	Missense_Mutation
**WBP1**	p.S232A	1	0	Missense_Mutation
**WBP11**	p.L235L	2	0	Silent
**WDFY3**	p.E3267K	1	2	Missense_Mutation
**WDFY4**	p.P2109Q	1	3	Missense_Mutation
**WDHD1**	p.S474C	1	7	Missense_Mutation
**WDR11**	p.R640L	3	6	Missense_Mutation
**WDR13**	p.R185R	1	0	Silent
**WDR13**	p.L232L	1	0	Silent
**WDR18**	p.S133R	3	7	Missense_Mutation
**WDR19**	p.I532T	1	7	Missense_Mutation
**WDR19**	p.M1316V	1	7	Missense_Mutation
**WDR27**	p.I211V	3	2	Missense_Mutation
**WDR3**	p.N793D	1	4	Missense_Mutation
**WDR33**	p.A1138A	1	0	Silent
**WDR36**	p.K416T	1	6	Missense_Mutation
**WDR36**	p.Y388H	1	3	Missense_Mutation
**WDR5**	p.T3M	1	4	Missense_Mutation
**WDR64**	p.I707L	1	2	Missense_Mutation
**WDR66**	p.V899I	2	0	Missense_Mutation
**WDR7**	p.I1062V	1	2	Missense_Mutation
**WDR88**	p.H272R	1	7	Missense_Mutation
**WDR90**	p.T275P	1	2	Missense_Mutation
**WFDC8**	p.L234M	1	2	Missense_Mutation
**WFIKKN1**	p.S162R	2	1	Missense_Mutation
**WNK1**	p.T1812S	1	1	Missense_Mutation
**WNT8A**	p.P255P	1	0	Silent
**WRNIP1**	p.G308E	1	7	Missense_Mutation
**WWC2**	p.S425L	1	5	Missense_Mutation
**WWC3**	p.I604M	3	4	Missense_Mutation
**XIRP2**	p.Q2905*	1	1	Nonsense_Mutation
**XIRP2**	p.Q750Q	1	0	Silent
**XK**	p.S140A	1	2	Missense_Mutation
**XKR4**	p.G7G	1	0	Silent
**XPO1**	p.A947A	1	0	Silent
**XPO7**	p.N132N	2	1	Silent
**XRN2**	p.S455T	1	1	Missense_Mutation
**XRRA1**	p.V532V	1	0	Silent
**YIPF2**	p.S31S	1	0	Silent
**YTHDC1**	p.E706*	1	2	Nonsense_Mutation
**YWHAZ**	p.G168G	1	0	Silent
**ZAR1**	p.T382R	1	6	Missense_Mutation
**ZAR1**	p.V179I	1	0	Missense_Mutation
**ZBED6**	p.Q811Q	1	0	Silent
**ZBTB10**	p.Y802D	1	6	Missense_Mutation
**ZBTB11**	p.A560T	1	1	Missense_Mutation
**ZBTB40**	p.K801N	1	6	Missense_Mutation
**ZBTB41**	p.N72T	1	4	Missense_Mutation
**ZBTB43**	p.Y148Y	1	0	Silent

**ZBTB8B**	p.S288R	2	2	■															■					Missense_Mutation
**ZC3H12A**	p.R158W	2	7														■	■						Missense_Mutation
**ZC3H12A**	p.R215C	1	7								■													Missense_Mutation
**ZC3H12B**	p.D820N	1	6							■														Missense_Mutation
**ZCCHC14**	p.L208F	3	5	■															■	■				Missense_Mutation
**ZDBF2**	p.L626I	1	3													■								Missense_Mutation
**ZDHHC3**	p.Y29N	1	0												■									Missense_Mutation
**ZDHHC5**	p.S371S	1	0															■						Silent
**ZFAND1**	p.E162Q	1	5										■											Missense_Mutation
**ZFAT**	p.L859L	1	0													■								Silent
**ZFHX3**	p.G3515S	1	3																	■				Missense_Mutation
**ZFHX3**	p.I3043V	1	2					■																Missense_Mutation
**ZFHX3**	p.-3514fs	3	0	■									■						■					Frame_Shift_Ins
**ZFP1**	p.A170V	1	0							■														Missense_Mutation
**ZFP14**	p.E253D	1	6												■									Missense_Mutation
**ZFP14**	p.S360R	1	2							■														Missense_Mutation
**ZFP14**	p.G374G	1	0											■										Silent
**ZFP14**	p.H362H	1	0											■										Silent
**ZFP30**	p.S144C	1	2		■																			Missense_Mutation
**ZFP62**	p.C222*	1	1							■														Nonsense_Mutation
**ZFP62**	p.G223G	1	0					■																Silent
**ZFP69B**	p.G114R	1	5								■													Splice_Site
**ZFP69B**	p.A41S	1	1				■																	Missense_Mutation
**ZFP82**	p.T333I	1	7		■																			Missense_Mutation
**ZFP82**	p.R358I	1	5					■																Missense_Mutation
**ZFP82**	p.V349V	1	0		■																			Silent
**ZFP82**	p.K336K	1	0		■																			Silent
**ZFPM2**	p.E1081D	1	2													■								Missense_Mutation
**ZFPM2**	p.A480A	3	0	■															■	■				Silent
**ZFX**	p.D70D	2	0														■	■						Silent
**ZFYVE26**	NULLL	1	1	■																				Splice_Site
**ZIC1**	p.H435Q	2	4														■	■						Missense_Mutation
**ZIK1**	p.N131K	1	2													■								Missense_Mutation
**ZIK1**	p.H133Y	1	2													■								Missense_Mutation
**ZKSCAN2**	p.E42A	1	7									■												Missense_Mutation
**ZKSCAN4**	p.G58C	1	4										■											Missense_Mutation
**ZMIZ1**	p.P955A	1	2								■													Missense_Mutation
**ZMYM1**	p.P666L	3	4	■															■	■				Missense_Mutation
**ZMYM2**	NULLL	2	1														■	■						Splice_Site
**ZMYM3**	p.I1110I	1	0													■								Silent
**ZNF100**	p.H335Q	1	6									■												Missense_Mutation
**ZNF100**	p.K280M	1	4															■						Missense_Mutation
**ZNF100**	p.R290I	1	1			■																		Missense_Mutation
**ZNF100**	p.M477I	1	0																■					Missense_Mutation
**ZNF100**	p.A474S	1	0		■																			Missense_Mutation
**ZNF100**	p.Q356R	1	0									■												Missense_Mutation
**ZNF100**	p.G351G	2	0	■															■					Silent
**ZNF100**	p.P344P	1	0													■								Silent
**ZNF101**	p.R69R	1	0			■																		Silent
**ZNF107**	p.S368S	1	0							■														Silent
**ZNF107**	p.V560A	2	0	■															■					Missense_Mutation
**ZNF107**	p.S676F	1	0								■													Missense_Mutation
**ZNF107**	p.A681E	1	0								■													Missense_Mutation
**ZNF112**	p.G620A	1	1											■										Missense_Mutation
**ZNF112**	p.P630P	1	0												■									Silent
**ZNF112**	p.K604K	1	0										■											Silent
**ZNF112**	p.Y603Y	1	0										■											Silent

**ZNF112**	p.C577C	1	0								■													Silent
**ZNF117**	p.P444L	1	6										■											Missense_Mutation
**ZNF117**	p.L345H	1	1								■													Missense_Mutation
**ZNF117**	p.E336D	1	1		■																			Missense_Mutation
**ZNF117**	p.Q472P	2	0										■				■							Missense_Mutation
**ZNF117**	p.A453A	1	0										■											Silent
**ZNF117**	p.A369A	1	0													■								Silent
**ZNF117**	p.V353L	1	0								■													Missense_Mutation
**ZNF117**	p.R182T	1	0							■														Missense_Mutation
**ZNF132**	p.L153F	1	1													■								Missense_Mutation
**ZNF132**	p.T454T	1	0										■											Silent
**ZNF135**	p.T475T	1	1											■										Silent
**ZNF136**	p.P279P	1	0					■																Silent
**ZNF14**	p.Y461*	1	1											■										Nonsense_Mutation
**ZNF141**	p.*475G	1	0						■															Nonstop_Mutation
**ZNF142**	p.H1000Y	1	0						■															Missense_Mutation
**ZNF142**	p.C980S	1	0			■																		Missense_Mutation
**ZNF148**	p.T782T	1	0			■																		Silent
**ZNF154**	p.K293I	1	4							■														Missense_Mutation
**ZNF154**	p.K411R	1	0			■																		Missense_Mutation
**ZNF154**	p.P244P	2	0							■					■									Silent
**ZNF169**	p.P457P	1	0							■														Silent
**ZNF180**	p.E385D	1	1								■													Missense_Mutation
**ZNF180**	p.A370V	1	1													■								Missense_Mutation
**ZNF189**	p.R546K	1	4					■																Missense_Mutation
**ZNF19**	p.A239A	2	0	■															■					Silent
**ZNF20**	p.R342K	2	0	■															■					Missense_Mutation
**ZNF205**	p.S94S	3	0	■															■	■				Silent
**ZNF208**	p.G1221V	2	5						■		■													Missense_Mutation
**ZNF208**	p.C891Y	1	5						■															Missense_Mutation
**ZNF208**	p.K1111T	3	4	■								■							■					Missense_Mutation
**ZNF208**	p.T744A	3	4	■														■	■					Missense_Mutation
**ZNF208**	p.Y201H	1	3				■																	Missense_Mutation
**ZNF208**	p.L1280P	1	2			■																		Missense_Mutation
**ZNF208**	p.F733V	1	2															■						Missense_Mutation
**ZNF208**	p.Y766*	1	1						■															Nonsense_Mutation
**ZNF208**	p.A1233G	1	0			■																		Missense_Mutation
**ZNF208**	p.P1209S	4	0						■		■		■				■							Missense_Mutation
**ZNF208**	p.M1101I	2	0	■															■					Missense_Mutation
**ZNF208**	p.S1043I	1	0													■								Missense_Mutation
**ZNF208**	p.T1027K	1	0				■																	Missense_Mutation
**ZNF208**	p.P888P	1	0						■															Silent
**ZNF208**	p.A841A	1	0		■																			Silent
**ZNF208**	p.K759N	1	0						■															Missense_Mutation
**ZNF208**	p.Y758F	1	0						■															Missense_Mutation
**ZNF208**	p.A757A	1	0						■															Silent
**ZNF208**	p.K747K	3	0	■														■	■					Silent
**ZNF208**	p.T645A	1	0																■					Missense_Mutation
**ZNF211**	p.R481L	1	5							■														Missense_Mutation
**ZNF219**	p.K514T	1	4				■																	Missense_Mutation
**ZNF225**	p.F484F	3	0			■	■			■														Silent
**ZNF227**	p.E377E	1	0													■								Silent
**ZNF227**	p.K381K	1	0													■								Silent
**ZNF227**	p.G656G	1	0								■													Silent
**ZNF229**	p.N774I	1	0		■																			Missense_Mutation
**ZNF25**	p.M279T	1	0							■														Missense_Mutation
**ZNF25**	p.P229P	1	0								■													Silent

**ZNF250**	p.T403A	1	1									■												Missense_Mutation
**ZNF253**	p.I257T	1	3			■																		Missense_Mutation
**ZNF253**	p.I145V	1	0								■													Missense_Mutation
**ZNF253**	p.S165S	1	0					■																Silent
**ZNF253**	p.H274N	2	0	■															■					Missense_Mutation
**ZNF254**	p.C548W	1	7									■												Missense_Mutation
**ZNF254**	p.K397N	1	6						■															Missense_Mutation
**ZNF254**	p.R458*	1	2													■								Nonsense_Mutation
**ZNF254**	p.Q361R	1	1									■												Missense_Mutation
**ZNF254**	p.K603*	1	1									■												Nonsense_Mutation
**ZNF254**	p.A442A	1	0			■																		Silent
**ZNF256**	p.I344F	1	4											■										Missense_Mutation
**ZNF256**	p.M380K	1	0							■														Missense_Mutation
**ZNF256**	p.H345H	1	0				■																	Silent
**ZNF257**	p.R240L	1	2							■														Missense_Mutation
**ZNF257**	p.R253G	1	0													■								Missense_Mutation
**ZNF257**	p.M336I	1	0										■											Missense_Mutation
**ZNF257**	p.K368E	2	0										■					■						Missense_Mutation
**ZNF26**	p.F281I	1	1			■																		Missense_Mutation
**ZNF267**	p.P687H	2	6	■															■					Missense_Mutation
**ZNF267**	p.E635D	1	2														■							Missense_Mutation
**ZNF267**	p.T649T	4	0	■		■				■									■					Silent
**ZNF267**	p.T705T	1	0												■									Silent
**ZNF268**	p.T587T	1	0							■														Silent
**ZNF273**	p.H368N	1	6			■																		Missense_Mutation
**ZNF273**	p.C516S	1	6							■														Missense_Mutation
**ZNF273**	p.E405K	1	2						■															Missense_Mutation
**ZNF273**	p.N349D	1	1									■												Missense_Mutation
**ZNF273**	p.R391G	1	1								■													Missense_Mutation
**ZNF273**	p.E405D	3	1								■						■		■					Missense_Mutation
**ZNF273**	p.A382A	1	0									■												Silent
**ZNF273**	p.R413W	2	0								■	■												Missense_Mutation
**ZNF28**	p.Q565L	1	2		■																			Missense_Mutation
**ZNF28**	p.E558E	1	0		■																			Silent
**ZNF28**	p.P553P	1	0		■																			Silent
**ZNF280A**	p.N278H	1	1					■																Missense_Mutation
**ZNF280D**	NULLL	1	0											■										Splice_Site
**ZNF283**	p.E395D	1	3								■													Missense_Mutation
**ZNF283**	p.H146H	2	0						■								■							Silent
**ZNF283**	p.T168T	1	0											■										Silent
**ZNF283**	p.K181*	2	0	■															■					Nonsense_Mutation
**ZNF283**	p.Q225Q	1	0							■														Silent
**ZNF283**	p.F301F	2	0									■					■							Silent
**ZNF287**	p.P451P	1	0										■											Silent
**ZNF292**	p.P586L	1	7								■													Missense_Mutation
**ZNF300**	p.C358*	1	1					■																Nonsense_Mutation
**ZNF304**	p.G506C	1	4														■							Missense_Mutation
**ZNF304**	p.S562R	1	3						■															Missense_Mutation
**ZNF304**	p.N366I	1	2										■											Missense_Mutation
**ZNF304**	p.S334P	1	0			■																		Missense_Mutation
**ZNF311**	p.G444V	1	0					■																Missense_Mutation
**ZNF318**	p.L941V	1	6							■														Missense_Mutation
**ZNF318**	p.S1809C	1	2		■																			Missense_Mutation
**ZNF318**	p.E2063K	1	1								■													Missense_Mutation
**ZNF334**	p.H640H	1	0				■																	Silent
**ZNF337**	p.G326G	1	0															■						Silent
**ZNF33A**	p.T567S	1	2							■														Missense_Mutation

**ZNF33A**	p.G381G	1	0					■																Silent
**ZNF33B**	p.F590L	1	5								■													Missense_Mutation
**ZNF33B**	p.I589N	1	3					■																Missense_Mutation
**ZNF33B**	p.T567S	1	2			■																		Missense_Mutation
**ZNF343**	p.Y465Y	1	0														■							Silent
**ZNF343**	p.V410V	1	0																■					Silent
**ZNF345**	p.G264G	1	0					■																Silent
**ZNF345**	p.T350A	2	0								■								■					Missense_Mutation
**ZNF347**	p.Y598*	1	1												■									Nonsense_Mutation
**ZNF347**	p.Q533Q	1	0												■									Silent
**ZNF35**	p.K506Q	1	2		■																			Missense_Mutation
**ZNF350**	p.A27T	1	0				■																	Missense_Mutation
**ZNF382**	p.E460D	1	3								■													Missense_Mutation
**ZNF382**	p.G459G	2	0								■	■												Silent
**ZNF383**	p.C340*	1	1													■								Nonsense_Mutation
**ZNF383**	p.E103Q	3	0	■															■	■				Missense_Mutation
**ZNF396**	p.E31*	1	1						■															Nonsense_Mutation
**ZNF407**	p.R271C	3	4	■															■	■				Missense_Mutation
**ZNF407**	p.K634N	1	0													■								Missense_Mutation
**ZNF408**	p.S574S	1	0									■												Silent
**ZNF415**	p.E493E	1	0															■						Silent
**ZNF416**	p.K315N	1	0							■														Missense_Mutation
**ZNF418**	p.R340I	1	5													■								Missense_Mutation
**ZNF418**	p.E311*	1	2					■																Nonsense_Mutation
**ZNF418**	p.R529L	1	1					■																Missense_Mutation
**ZNF418**	p.S447G	1	1											■										Missense_Mutation
**ZNF418**	p.L385L	1	0											■										Silent
**ZNF418**	p.G376G	1	0									■												Silent
**ZNF418**	p.G317E	1	0												■									Missense_Mutation
**ZNF418**	p.G289G	1	0													■								Silent
**ZNF418**	p.R278R	1	0											■										Silent
**ZNF419**	p.I451T	1	1										■											Missense_Mutation
**ZNF419**	p.K491K	1	0										■											Silent
**ZNF420**	p.H437H	1	0					■																Silent
**ZNF420**	p.I468I	1	0							■														Silent
**ZNF420**	p.T470T	2	0							■	■													Splice_Site
**ZNF420**	p.S488S	1	0								■													Silent
**ZNF429**	p.S325Y	1	3																■					Missense_Mutation
**ZNF429**	p.T274N	1	2			■																		Missense_Mutation
**ZNF429**	p.S280T	1	0								■													Missense_Mutation
**ZNF429**	p.S353S	1	0																■					Silent
**ZNF429**	p.I519I	1	0										■											Silent
**ZNF43**	p.K752R	2	0	■															■					Missense_Mutation
**ZNF43**	p.S740S	1	0							■														Silent
**ZNF43**	p.I664I	1	0									■												Silent
**ZNF43**	p.W655L	1	0									■												Missense_Mutation
**ZNF43**	p.T465K	1	0													■								Missense_Mutation
**ZNF43**	p.V452E	5	0		■	■							■		■	■								Missense_Mutation
**ZNF43**	p.T434N	1	0			■																		Missense_Mutation
**ZNF43**	p.P432S	1	0			■																		Missense_Mutation
**ZNF43**	p.A400A	1	0							■														Silent
**ZNF430**	p.N439K	1	2					■																Missense_Mutation
**ZNF430**	p.V477L	1	1							■														Missense_Mutation
**ZNF430**	p.K271N	1	0						■															Missense_Mutation
**ZNF430**	p.A474E	2	0	■															■					Missense_Mutation
**ZNF431**	p.K314N	1	5							■														Missense_Mutation
**ZNF431**	p.T246P	1	3												■									Missense_Mutation

**ZNF431**	p.W243R	1	1												■									Missense_Mutation
**ZNF431**	p.F244S	1	0												■									Missense_Mutation
**ZNF431**	p.K258K	1	0												■									Silent
**ZNF432**	p.G300C	1	2			■																		Missense_Mutation
**ZNF432**	p.M501I	1	0							■														Missense_Mutation
**ZNF432**	p.I495N	2	0	■															■					Missense_Mutation
**ZNF432**	p.G491G	1	0														■							Silent
**ZNF432**	p.Q392Q	1	0								■													Silent
**ZNF433**	p.K29E	1	5			■																		Missense_Mutation
**ZNF433**	p.G225E	1	4							■														Missense_Mutation
**ZNF433**	p.P595T	1	2												■									Missense_Mutation
**ZNF433**	p.E528K	1	1														■							Missense_Mutation
**ZNF433**	p.Q559Q	1	0								■													Silent
**ZNF433**	p.P526P	1	0														■							Silent
**ZNF436**	p.I57F	1	4				■																	Missense_Mutation
**ZNF436**	p.E56E	1	0				■																	Silent
**ZNF440**	p.I52T	1	2				■																	Missense_Mutation
**ZNF442**	p.D276N	1	2										■											Missense_Mutation
**ZNF442**	p.V281L	1	0										■											Missense_Mutation
**ZNF443**	p.H275Q	1	5											■										Missense_Mutation
**ZNF45**	p.Q455R	2	2	■															■					Missense_Mutation
**ZNF454**	p.G29E	3	0	■															■	■				Missense_Mutation
**ZNF460**	p.P447T	1	6						■															Missense_Mutation
**ZNF468**	p.I484F	1	2										■											Missense_Mutation
**ZNF468**	p.I483L	1	0							■														Missense_Mutation
**ZNF470**	p.K529I	1	3							■														Missense_Mutation
**ZNF470**	p.Y579Y	1	0														■							Silent
**ZNF479**	p.L452L	1	0									■												Silent
**ZNF485**	p.N273H	1	1							■														Missense_Mutation
**ZNF486**	p.I267N	4	1	■					■									■	■					Missense_Mutation
**ZNF486**	p.I267I	1	0						■															Silent
**ZNF491**	p.K190T	1	1													■								Missense_Mutation
**ZNF491**	p.G328V	1	0			■																		Missense_Mutation
**ZNF492**	p.C395Y	1	7							■														Missense_Mutation
**ZNF492**	p.I413M	2	4							■			■											Missense_Mutation
**ZNF492**	p.S405S	1	0							■														Silent
**ZNF492**	p.A485A	1	0			■																		Silent
**ZNF493**	p.T428N	1	6													■								Missense_Mutation
**ZNF493**	p.G513E	1	5																■					Missense_Mutation
**ZNF493**	p.T533S	2	2									■						■						Missense_Mutation
**ZNF493**	p.R528Q	1	1						■															Missense_Mutation
**ZNF493**	p.T456T	1	0									■												Silent
**ZNF493**	p.K576E	1	0						■															Missense_Mutation
**ZNF493**	p.S581A	1	0						■															Missense_Mutation
**ZNF493**	p.S613S	1	0						■															Silent
**ZNF493**	p.I615T	1	0						■															Missense_Mutation
**ZNF493**	p.S617T	1	0						■															Missense_Mutation
**ZNF493**	p.C713Y	1	0						■															Missense_Mutation
**ZNF496**	p.L443P	2	3														■	■						Missense_Mutation
**ZNF496**	p.V559V	1	0													■								Silent
**ZNF532**	p.R569Q	1	3			■																		Missense_Mutation
**ZNF546**	p.R430L	1	1										■											Missense_Mutation
**ZNF546**	p.P328P	2	0					■									■							Silent
**ZNF546**	p.C549G	1	0					■																Missense_Mutation
**ZNF548**	p.R357I	1	6													■								Missense_Mutation
**ZNF548**	p.R291L	2	2							■							■							Missense_Mutation
**ZNF548**	p.K326I	1	2									■												Missense_Mutation

**ZNF548**	p.R312S	1	1	Missense_Mutation
**ZNF548**	p.D320Y	1	1	Missense_Mutation
**ZNF548**	p.P308P	2	0	Silent
**ZNF549**	p.H343H	1	0	Silent
**ZNF549**	p.C363C	1	0	Silent
**ZNF551**	p.S375I	1	2	Missense_Mutation
**ZNF551**	p.G509G	3	1	Silent
**ZNF551**	p.G565G	2	1	Silent
**ZNF551**	p.K566K	1	1	Silent
**ZNF551**	p.S567S	1	1	Silent
**ZNF551**	p.R450S	1	0	Missense_Mutation
**ZNF551**	p.G608V	1	0	Missense_Mutation
**ZNF552**	p.C298*	1	1	Nonsense_Mutation
**ZNF554**	p.Y520C	1	6	Missense_Mutation
**ZNF555**	p.L351H	1	2	Missense_Mutation
**ZNF555**	p.P474T	1	0	Missense_Mutation
**ZNF556**	p.H308D	1	5	Missense_Mutation
**ZNF557**	p.Y208Y	1	0	Silent
**ZNF557**	p.T216S	1	0	Missense_Mutation
**ZNF560**	p.T623S	1	3	Missense_Mutation
**ZNF560**	p.D614Y	1	0	Missense_Mutation
**ZNF560**	p.E611E	1	0	Silent
**ZNF560**	p.T610T	1	0	Silent
**ZNF560**	p.M575K	2	0	Missense_Mutation
**ZNF563**	p.M246K	1	1	Missense_Mutation
**ZNF564**	p.G195*	1	1	Nonsense_Mutation
**ZNF564**	p.G305G	1	0	Silent
**ZNF564**	p.G259G	1	0	Silent
**ZNF566**	p.H173Q	1	7	Missense_Mutation
**ZNF566**	p.G163G	2	0	Silent
**ZNF567**	p.G564E	3	3	Missense_Mutation
**ZNF568**	p.E218*	1	2	Nonsense_Mutation
**ZNF57**	p.Q352R	1	2	Missense_Mutation
**ZNF574**	p.D45H	1	2	Missense_Mutation
**ZNF578**	p.N318Y	1	4	Missense_Mutation
**ZNF578**	p.E312D	1	0	Missense_Mutation
**ZNF578**	p.Y427fs	1	0	Frame_Shift_Ins
**ZNF583**	p.K302I	2	3	Missense_Mutation
**ZNF583**	p.V205A	1	1	Missense_Mutation
**ZNF585A**	p.H596Q	1	6	Missense_Mutation
**ZNF585A**	p.H208Y	1	6	Missense_Mutation
**ZNF585B**	NULLL	1	0	De_novo_Start_InFrame
**ZNF587**	p.H510Y	1	6	Missense_Mutation
**ZNF587B**	p.Y371F	1	1	Missense_Mutation
**ZNF587B**	p.I392S	2	0	Missense_Mutation
**ZNF587B**	p.N595I	1	0	Missense_Mutation
**ZNF596**	p.K249N	1	5	Missense_Mutation
**ZNF596**	p.S263I	1	2	Missense_Mutation
**ZNF596**	p.V395L	1	1	Missense_Mutation
**ZNF596**	p.C424*	1	1	Nonsense_Mutation
**ZNF596**	p.I384T	1	0	Missense_Mutation
**ZNF596**	p.E402Q	1	0	Missense_Mutation
**ZNF596**	p.E402D	1	0	Missense_Mutation
**ZNF596**	p.C424C	2	0	Silent
**ZNF606**	p.K734N	1	7	Missense_Mutation
**ZNF608**	p.S291R	3	0	Missense_Mutation
**ZNF611**	p.K358T	1	5	Missense_Mutation

**ZNF611**	p.Y511S	1	1											■										Missense_Mutation
**ZNF611**	p.H508R	1	1															■						Missense_Mutation
**ZNF614**	p.I443V	1	0											■										Missense_Mutation
**ZNF614**	p.G433G	2	0								■			■										Silent
**ZNF614**	p.N430S	1	0								■													Missense_Mutation
**ZNF614**	p.S426S	1	0				■																	Silent
**ZNF614**	p.S346N	1	0									■												Missense_Mutation
**ZNF616**	p.K441*	1	1							■														Nonsense_Mutation
**ZNF616**	p.C561R	1	0						■															Missense_Mutation
**ZNF616**	p.K552N	1	0						■															Missense_Mutation
**ZNF616**	p.V510A	1	0						■															Missense_Mutation
**ZNF616**	p.H505Y	2	0				■				■													Missense_Mutation
**ZNF616**	p.Q484R	1	0								■													Missense_Mutation
**ZNF616**	p.V473V	1	0						■															Silent
**ZNF618**	p.T567T	1	0	■																				Silent
**ZNF625**	p.A264S	2	1									■		■										Missense_Mutation
**ZNF625**	p.K306K	1	0								■													Silent
**ZNF626**	p.G365E	1	4										■											Missense_Mutation
**ZNF626**	p.K360N	1	4										■											Missense_Mutation
**ZNF626**	p.T357S	1	1							■														Missense_Mutation
**ZNF626**	p.I305R	1	1										■											Missense_Mutation
**ZNF626**	p.A461A	1	0										■											Silent
**ZNF626**	p.Y352*	1	0							■														Nonsense_Mutation
**ZNF626**	p.Y352S	1	0							■														Missense_Mutation
**ZNF626**	p.R296R	1	0										■											Silent
**ZNF626**	p.N295K	2	0	■															■					Missense_Mutation
**ZNF626**	p.S213S	1	0														■							Silent
**ZNF627**	p.T394T	1	0												■									Silent
**ZNF629**	p.K344Q	1	6													■								Missense_Mutation
**ZNF629**	p.K363N	1	4							■														Missense_Mutation
**ZNF629**	p.G356S	1	4							■														Missense_Mutation
**ZNF629**	p.E347K	1	3								■													Missense_Mutation
**ZNF638**	p.H587Y	1	3					■																Missense_Mutation
**ZNF649**	p.C236*	1	1					■																Nonsense_Mutation
**ZNF658**	p.E599G	1	6								■													Missense_Mutation
**ZNF658**	p.E599K	1	4								■													Missense_Mutation
**ZNF658**	p.I987V	1	1												■									Missense_Mutation
**ZNF658**	p.A1002A	1	0										■											Silent
**ZNF658**	p.K1001K	1	0										■											Silent
**ZNF658**	p.T989T	1	0										■											Silent
**ZNF658**	p.H988H	1	0										■											Silent
**ZNF658**	p.C604C	1	0								■													Silent
**ZNF658**	p.E599E	1	0								■													Silent
**ZNF658**	p.D578E	1	0														■							Missense_Mutation
**ZNF66**	p.S270S	1	0									■												Silent
**ZNF66**	p.Y352H	1	0															■						Missense_Mutation
**ZNF664**	p.R200I	1	4						■															Missense_Mutation
**ZNF664**	p.S157I	1	3														■							Missense_Mutation
**ZNF664**	p.C232*	1	2								■													Nonsense_Mutation
**ZNF664**	p.P226P	1	0								■													Silent
**ZNF665**	p.G186S	1	0		■																			Missense_Mutation
**ZNF665**	p.D26D	1	0								■													Silent
**ZNF674**	p.A561A	1	0								■													Silent
**ZNF674**	p.H544Q	1	0								■													Missense_Mutation
**ZNF675**	p.G448E	1	5														■							Missense_Mutation
**ZNF675**	p.R332I	1	3														■							Missense_Mutation
**ZNF675**	p.S268Y	1	3								■													Missense_Mutation

**ZNF675**	p.K444I	2	1	Missense_Mutation
**ZNF675**	p.K406N	1	1	Missense_Mutation
**ZNF675**	p.K399E	2	1	Missense_Mutation
**ZNF675**	p.Q366K	1	1	Missense_Mutation
**ZNF675**	p.T326N	1	1	Missense_Mutation
**ZNF675**	p.K415K	1	0	Silent
**ZNF675**	p.T335T	1	0	Silent
**ZNF676**	p.E522K	1	1	Missense_Mutation
**ZNF676**	p.N235S	2	1	Missense_Mutation
**ZNF676**	p.I525R	1	0	Missense_Mutation
**ZNF676**	p.F464L	1	0	Missense_Mutation
**ZNF676**	p.S431T	2	0	Missense_Mutation
**ZNF676**	p.N235N	1	0	Silent
**ZNF677**	p.G364C	1	2	Missense_Mutation
**ZNF678**	p.R285K	2	2	Missense_Mutation
**ZNF678**	p.T288S	2	2	Missense_Mutation
**ZNF678**	p.E276*	2	1	Nonsense_Mutation
**ZNF678**	p.C277W	2	1	Missense_Mutation
**ZNF678**	p.F473C	1	1	Missense_Mutation
**ZNF678**	p.K142N	1	0	Missense_Mutation
**ZNF678**	p.R145K	1	0	Missense_Mutation
**ZNF678**	p.N170S	1	0	Missense_Mutation
**ZNF678**	p.P180P	1	0	Silent
**ZNF678**	p.C239C	1	0	Silent
**ZNF678**	p.P264P	1	0	Silent
**ZNF678**	p.K266K	2	0	Silent
**ZNF678**	p.T329A	4	0	Missense_Mutation
**ZNF678**	p.R332Q	2	0	Missense_Mutation
**ZNF678**	p.T357A	4	0	Missense_Mutation
**ZNF678**	p.A469A	1	0	Silent
**ZNF678**	p.L476L	1	0	Silent
**ZNF678**	p.R488P	1	0	Missense_Mutation
**ZNF679**	p.E265D	1	6	Missense_Mutation
**ZNF680**	p.S461T	2	2	Missense_Mutation
**ZNF680**	p.R314K	1	1	Missense_Mutation
**ZNF681**	p.C368R	1	7	Missense_Mutation
**ZNF681**	p.H380P	1	4	Missense_Mutation
**ZNF681**	p.G372D	1	4	Missense_Mutation
**ZNF681**	p.I386R	1	1	Missense_Mutation
**ZNF681**	p.G484G	1	0	Silent
**ZNF681**	p.G456G	1	0	Silent
**ZNF681**	p.K385K	1	0	Silent
**ZNF682**	p.A237A	1	0	Silent
**ZNF695**	p.N453H	1	2	Missense_Mutation
**ZNF695**	p.Y422*	1	1	Nonsense_Mutation
**ZNF695**	p.Y422N	1	0	Missense_Mutation
**ZNF695**	p.A416A	2	0	Silent
**ZNF699**	p.Y542C	2	2	Missense_Mutation
**ZNF699**	p.K535E	1	0	Missense_Mutation
**ZNF70**	p.C374S	1	0	Missense_Mutation
**ZNF70**	p.G164G	2	0	Silent
**ZNF700**	p.R448W	1	6	Missense_Mutation
**ZNF700**	p.H560N	2	2	Missense_Mutation
**ZNF700**	p.T440A	1	0	Missense_Mutation
**ZNF701**	p.E171D	1	4	Missense_Mutation
**ZNF704**	p.G326V	1	6	Missense_Mutation
**ZNF705A**	p.E68K	1	1	Missense_Mutation

**ZNF705B**	p.V71E	1	1	Missense_Mutation
**ZNF708**	p.V384E	1	2	Missense_Mutation
**ZNF708**	p.Y409N	2	0	Missense_Mutation
**ZNF708**	p.K383K	1	0	Silent
**ZNF708**	p.N381K	1	0	Missense_Mutation
**ZNF708**	p.N297K	1	0	Missense_Mutation
**ZNF708**	p.N294H	2	0	Missense_Mutation
**ZNF708**	p.Q291R	1	0	Missense_Mutation
**ZNF708**	p.A288A	1	0	Silent
**ZNF708**	p.N266T	1	0	Missense_Mutation
**ZNF708**	p.N266H	1	0	Missense_Mutation
**ZNF708**	p.R241K	1	0	Missense_Mutation
**ZNF708**	p.K206N	1	0	Missense_Mutation
**ZNF709**	p.S458I	3	2	Missense_Mutation
**ZNF709**	p.F519V	2	1	Missense_Mutation
**ZNF709**	p.F491I	1	1	Missense_Mutation
**ZNF709**	p.Q452H	1	1	Missense_Mutation
**ZNF709**	p.E561E	2	0	Silent
**ZNF709**	p.P559P	1	0	Silent
**ZNF709**	p.S518S	1	0	Silent
**ZNF709**	p.E505E	2	0	Silent
**ZNF709**	p.K483K	2	0	Silent
**ZNF71**	p.R318S	1	5	Missense_Mutation
**ZNF71**	p.A243V	1	2	Missense_Mutation
**ZNF71**	p.G238G	1	0	Silent
**ZNF71**	p.G245G	1	0	Silent
**ZNF71**	p.D246E	1	0	Missense_Mutation
**ZNF71**	p.N308S	1	0	Missense_Mutation
**ZNF714**	p.T357I	1	3	Missense_Mutation
**ZNF714**	p.N438K	1	1	Missense_Mutation
**ZNF714**	p.G430G	1	0	Silent
**ZNF714**	p.A432A	1	0	Silent
**ZNF716**	p.Q98P	1	1	Missense_Mutation
**ZNF716**	p.P268P	1	0	Silent
**ZNF716**	p.R274C	2	0	Missense_Mutation
**ZNF721**	p.R584W	3	2	Missense_Mutation
**ZNF721**	p.G583V	1	2	Missense_Mutation
**ZNF721**	p.A558S	1	2	Missense_Mutation
**ZNF721**	p.A306S	1	2	Missense_Mutation
**ZNF721**	p.K873I	2	1	Missense_Mutation
**ZNF721**	p.C351*	2	1	Nonsense_Mutation
**ZNF721**	p.D885E	1	0	Missense_Mutation
**ZNF721**	p.T882T	2	0	Silent
**ZNF721**	p.A870A	1	0	Silent
**ZNF721**	p.A866S	1	0	Missense_Mutation
**ZNF721**	p.S865S	1	0	Silent
**ZNF721**	p.R844K	2	0	Missense_Mutation
**ZNF721**	p.V842E	1	0	Missense_Mutation
**ZNF721**	p.H679H	1	0	Silent
**ZNF721**	p.V562L	1	0	Missense_Mutation
**ZNF721**	p.D353E	1	0	Missense_Mutation
**ZNF724P**	p.E370D	1	3	Missense_Mutation
**ZNF724P**	p.S490F	1	1	Missense_Mutation
**ZNF724P**	p.L489Q	1	0	Missense_Mutation
**ZNF724P**	p.R461Q	1	0	Missense_Mutation
**ZNF724P**	p.Q436H	1	0	Missense_Mutation
**ZNF724P**	p.E342E	1	0	Silent

**ZNF724P**	p.G260G	1	0	Silent
**ZNF727**	p.H290D	1	0	Missense_Mutation
**ZNF727**	p.N315K	1	0	Missense_Mutation
**ZNF727**	p.P437S	1	0	Missense_Mutation
**ZNF728**	p.F436F	2	0	Silent
**ZNF728**	p.T435K	2	0	Missense_Mutation
**ZNF728**	p.P380S	2	0	Missense_Mutation
**ZNF728**	p.A320A	2	0	Silent
**ZNF728**	p.A296V	1	0	Missense_Mutation
**ZNF728**	p.K295N	1	0	Missense_Mutation
**ZNF729**	p.Y656C	1	4	Missense_Mutation
**ZNF729**	p.G400A	1	3	Missense_Mutation
**ZNF729**	p.G512R	1	3	Missense_Mutation
**ZNF729**	p.G662S	3	3	Missense_Mutation
**ZNF729**	p.P683S	1	3	Missense_Mutation
**ZNF729**	p.S414N	1	1	Missense_Mutation
**ZNF729**	p.Q415H	2	1	Missense_Mutation
**ZNF729**	p.A670V	2	1	Missense_Mutation
**ZNF729**	p.K681*	1	1	Nonsense_Mutation
**ZNF729**	p.N278T	2	0	Missense_Mutation
**ZNF729**	p.D281E	3	0	Missense_Mutation
**ZNF729**	p.S358N	1	0	Missense_Mutation
**ZNF729**	p.N498K	1	0	Missense_Mutation
**ZNF729**	p.G512G	1	0	Silent
**ZNF729**	p.P515P	1	0	Silent
**ZNF729**	p.R532S	1	0	Missense_Mutation
**ZNF729**	p.V561I	1	0	Missense_Mutation
**ZNF729**	p.H594H	2	0	Silent
**ZNF729**	p.H667N	2	0	Missense_Mutation
**ZNF729**	p.F668S	2	0	Missense_Mutation
**ZNF729**	p.S875T	1	0	Missense_Mutation
**ZNF729**	p.K933E	1	0	Missense_Mutation
**ZNF729**	p.N946N	1	0	Silent
**ZNF729**	p.T950A	1	0	Missense_Mutation
**ZNF729**	p.M952K	1	0	Missense_Mutation
**ZNF729**	p.M952R	1	0	Missense_Mutation
**ZNF729**	p.P963P	1	0	Silent
**ZNF729**	p.A1056A	1	0	Silent
**ZNF729**	p.W1059R	1	0	Missense_Mutation
**ZNF729**	p.W1059*	2	0	Nonsense_Mutation
**ZNF729**	p.K1062N	2	0	Missense_Mutation
**ZNF729**	p.E1065K	2	0	Missense_Mutation
**ZNF729**	p.I1152V	1	0	Missense_Mutation
**ZNF729**	p.A1168A	1	0	Silent
**ZNF729**	p.H1174N	2	0	Missense_Mutation
**ZNF729**	p.R1177K	3	0	Missense_Mutation
**ZNF729**	p.K1179K	3	0	Silent
**ZNF730**	p.N356T	2	0	Missense_Mutation
**ZNF732**	p.K447E	1	3	Missense_Mutation
**ZNF732**	p.Y474*	2	1	Nonsense_Mutation
**ZNF732**	p.S514S	1	0	Silent
**ZNF732**	p.R487T	1	0	Missense_Mutation
**ZNF732**	p.I438R	1	0	Missense_Mutation
**ZNF732**	p.N325K	1	0	Missense_Mutation
**ZNF732**	p.F278Y	2	0	Missense_Mutation
**ZNF737**	p.F378L	1	5	Missense_Mutation
**ZNF737**	p.F494S	2	0	Missense_Mutation

**ZNF737**	p.F437S	2	0	■															■					Missense_Mutation
**ZNF737**	p.S298S	1	0									■												Silent
**ZNF750**	p.P297P	1	0									■												Silent
**ZNF765**	p.H345N	1	5										■											Missense_Mutation
**ZNF765**	p.F364L	1	4							■														Missense_Mutation
**ZNF765**	p.K409N	1	4										■											Missense_Mutation
**ZNF765**	p.G333S	1	2												■									Missense_Mutation
**ZNF765**	p.F364F	1	0										■											Silent
**ZNF765**	p.F370I	1	0							■														Missense_Mutation
**ZNF765**	p.S397S	1	0										■											Silent
**ZNF765**	p.Y400F	1	0									■												Missense_Mutation
**ZNF766**	p.R319K	1	0													■								Missense_Mutation
**ZNF773**	p.S313R	1	3		■																			Missense_Mutation
**ZNF776**	p.I481F	2	5					■							■									Missense_Mutation
**ZNF776**	p.Q480L	1	2					■																Missense_Mutation
**ZNF776**	p.S474R	1	0			■																		Missense_Mutation
**ZNF780A**	p.E555D	1	0							■														Missense_Mutation
**ZNF780A**	p.H537Q	1	0												■									Missense_Mutation
**ZNF780B**	p.F417Y	1	1							■														Missense_Mutation
**ZNF781**	NULLL	1	0														■							De_novo_Start_OutOfFrame
**ZNF79**	p.K250*	1	1										■											Nonsense_Mutation
**ZNF79**	p.R315S	3	0								■	■					■							Missense_Mutation
**ZNF792**	p.G362G	1	0			■																		Silent
**ZNF799**	p.G590E	1	0									■												Missense_Mutation
**ZNF813**	p.T363S	1	1			■																		Missense_Mutation
**ZNF813**	p.N533I	1	1												■									Missense_Mutation
**ZNF813**	p.F392F	2	0			■							■											Silent
**ZNF813**	p.T486K	1	0														■							Missense_Mutation
**ZNF813**	p.A487A	1	0														■							Silent
**ZNF813**	p.H537N	1	0			■																		Missense_Mutation
**ZNF813**	p.Q562K	1	0															■						Missense_Mutation
**ZNF814**	p.R596C	3	5	■															■	■				Missense_Mutation
**ZNF814**	p.F501L	1	5				■																	Missense_Mutation
**ZNF814**	p.K303N	2	5						■	■														Missense_Mutation
**ZNF814**	p.K276N	1	5							■														Missense_Mutation
**ZNF814**	p.K331N	1	4			■																		Missense_Mutation
**ZNF814**	p.G505R	2	2	■															■					Missense_Mutation
**ZNF814**	p.E521Q	2	1	■															■					Missense_Mutation
**ZNF814**	p.Q503H	1	1										■											Missense_Mutation
**ZNF814**	p.F367I	2	1	■															■					Missense_Mutation
**ZNF814**	p.F339I	1	1								■													Missense_Mutation
**ZNF814**	p.K295N	1	1									■												Missense_Mutation
**ZNF814**	p.G544G	1	0								■													Silent
**ZNF814**	p.L509L	3	0	■											■				■					Silent
**ZNF814**	p.L507L	2	0	■															■					Silent
**ZNF814**	p.F501F	1	0										■											Silent
**ZNF814**	p.S500S	1	0										■											Silent
**ZNF814**	p.V365V	2	0	■															■					Silent
**ZNF814**	p.R41H	1	0			■																		Missense_Mutation
**ZNF816**	p.V573D	1	4		■																			Missense_Mutation
**ZNF816**	p.V601V	2	0			■		■																Silent
**ZNF816**	p.Q582K	1	0													■								Missense_Mutation
**ZNF816**	p.I579S	1	0													■								Missense_Mutation
**ZNF829**	p.R341S	1	5							■														Missense_Mutation
**ZNF83**	p.K175N	1	5							■														Missense_Mutation
**ZNF83**	p.W336L	1	0			■																		Missense_Mutation
**ZNF83**	p.I310I	1	0										■											Silent

**ZNF83**	p.P204P	3	0	Silent
**ZNF836**	p.V786D	2	4	Missense_Mutation
**ZNF836**	p.I362V	1	1	Missense_Mutation
**ZNF836**	p.-914fs	1	0	Frame_Shift_Ins
**ZNF841**	p.E762D	1	4	Missense_Mutation
**ZNF841**	p.S495C	1	4	Missense_Mutation
**ZNF841**	p.V506I	1	1	Missense_Mutation
**ZNF845**	p.K710M	1	2	Missense_Mutation
**ZNF845**	p.A851S	1	2	Missense_Mutation
**ZNF845**	p.E695D	1	0	Missense_Mutation
**ZNF85**	p.C20Y	2	5	Missense_Mutation
**ZNF85**	p.I164S	2	5	Missense_Mutation
**ZNF850**	p.H741R	1	1	Missense_Mutation
**ZNF850**	p.N722I	1	1	Missense_Mutation
**ZNF850**	p.D562N	1	1	Missense_Mutation
**ZNF850**	p.G433R	1	1	Missense_Mutation
**ZNF850**	p.A413G	1	1	Missense_Mutation
**ZNF850**	p.D394G	1	1	Missense_Mutation
**ZNF850**	p.K340N	1	1	Missense_Mutation
**ZNF850**	p.Q329L	1	1	Missense_Mutation
**ZNF850**	p.Q329R	1	1	Missense_Mutation
**ZNF850**	p.Q313E	1	1	Missense_Mutation
**ZNF850**	p.H310D	1	1	Missense_Mutation
**ZNF850**	p.T771S	2	0	Missense_Mutation
**ZNF850**	p.C759C	1	0	Silent
**ZNF850**	p.T743A	1	0	Missense_Mutation
**ZNF850**	p.D725G	2	0	Missense_Mutation
**ZNF850**	p.G399G	1	0	Silent
**ZNF850**	p.H327H	1	0	Silent
**ZNF862**	p.E155G	1	5	Missense_Mutation
**ZNF878**	p.F367Y	1	1	Missense_Mutation
**ZNF878**	p.E396E	1	0	Silent
**ZNF880**	p.A425A	1	0	Silent
**ZNF880**	p.L428V	1	0	Missense_Mutation
**ZNF880**	p.Y436Y	1	0	Silent
**ZNF880**	p.K437E	1	0	Missense_Mutation
**ZNF880**	p.K439N	1	0	Missense_Mutation
**ZNF90**	p.R408L	2	1	Missense_Mutation
**ZNF90**	p.I417R	3	1	Missense_Mutation
**ZNF90**	p.S242S	1	0	Silent
**ZNF90**	p.A246S	1	0	Missense_Mutation
**ZNF90**	p.R256P	1	0	Missense_Mutation
**ZNF90**	p.R256R	2	0	Silent
**ZNF90**	p.S392T	1	0	Missense_Mutation
**ZNF90**	p.K394E	6	0	Missense_Mutation
**ZNF90**	p.S525T	1	0	Missense_Mutation
**ZNF91**	p.S559L	1	6	Missense_Mutation
**ZNF91**	p.Q949K	1	3	Missense_Mutation
**ZNF91**	p.S948R	1	3	Missense_Mutation
**ZNF91**	p.K556R	1	3	Missense_Mutation
**ZNF91**	p.T955A	1	2	Missense_Mutation
**ZNF91**	p.N675K	1	1	Missense_Mutation
**ZNF91**	p.N669H	2	1	Missense_Mutation
**ZNF91**	p.K549E	1	1	Missense_Mutation
**ZNF91**	p.S503*	1	1	Nonsense_Mutation
**ZNF91**	p.T1101T	1	0	Silent
**ZNF91**	p.K815K	1	0	Silent

**ZNF91**	p.K556K	1	0	Silent
**ZNF91**	p.R500R	1	0	Silent
**ZNF92**	p.G177E	1	5	Missense_Mutation
**ZNF92**	p.E318D	1	4	Missense_Mutation
**ZNF92**	p.G317R	1	3	Missense_Mutation
**ZNF92**	p.I173R	2	0	Missense_Mutation
**ZNF92**	p.P264P	1	0	Silent
**ZNF92**	p.S305F	2	0	Missense_Mutation
**ZNF92**	p.K394E	1	0	Missense_Mutation
**ZNF92**	p.K396K	1	0	Silent
**ZNF92**	p.E402E	1	0	Silent
**ZNF93**	p.K258K	2	0	Silent
**ZNF93**	p.S414T	1	0	Missense_Mutation
**ZNF93**	p.S467T	1	0	Missense_Mutation
**ZNF98**	p.H512Y	1	6	Missense_Mutation
**ZNF98**	p.K285N	1	5	Missense_Mutation
**ZNF98**	p.T282S	1	2	Missense_Mutation
**ZNF98**	p.N220K	1	2	Missense_Mutation
**ZNF99**	p.G589E	2	5	Missense_Mutation
**ZNF99**	p.K367Q	1	5	Missense_Mutation
**ZNF99**	p.K535Q	1	4	Missense_Mutation
**ZNF99**	p.V753L	1	1	Missense_Mutation
**ZNF99**	p.A585V	4	1	Missense_Mutation
**ZNF99**	p.G1034G	1	0	Silent
**ZNF99**	p.T874A	1	0	Missense_Mutation
**ZNF99**	p.T760A	1	0	Missense_Mutation
**ZNF99**	p.Y746Y	2	0	Silent
**ZNF99**	p.R777R	1	0	Silent
**ZNF99**	p.K768K	3	0	Silent
**ZNF99**	p.K747N	1	0	Missense_Mutation
**ZNF99**	p.H579H	3	0	Silent
**ZNF99**	p.S577S	3	0	Silent
**ZNF99**	p.Q576Q	2	0	Silent
**ZNF99**	p.Q576R	2	0	Missense_Mutation
**ZNF99**	p.G571G	1	0	Silent
**ZNF99**	p.K562E	2	0	Missense_Mutation
**ZNF99**	p.K556E	3	0	Missense_Mutation
**ZNF99**	p.C458C	1	0	Silent
**ZNF99**	p.T355A	1	0	Missense_Mutation
**ZNF99**	p.C257Y	1	0	Missense_Mutation
**ZNRF3**	p.L40L	1	0	Silent
**ZNRF3**	p.G357G	1	0	Silent
**ZRANB1**	p.R28C	1	5	Missense_Mutation
**ZRANB1**	p.E334K	1	4	Missense_Mutation
**ZSCAN10**	p.K447T	1	4	Missense_Mutation
**ZSCAN2**	p.S168R	1	2	Missense_Mutation
**ZSCAN31**	p.I194S	1	1	Missense_Mutation
**ZXDC**	p.P454L	1	5	Missense_Mutation
**ZZEF1**	p.R1530Q	1	6	Missense_Mutation

Gene	Mutation	Reccurrence	Functional Prediction tools	SNU308	OCUG1	G415	TGBC2TKB	NOZ	Cosmic	dbSNP	TMC-SNPdb	Variant_Classification
MYO1F	p.A559T	1	7				■		COSM1003556			Missense_Mutation
OCM2	p.G57V	2	7	■		■	■	■	COSM1330444	rs201256816		Missense_Mutation
PGAM2	p.G97D	1	7	■				■	COSM150277	rs77938727		Missense_Mutation
ALPL	p.A96V	1	7	■	■	■		■	COSM1687146			Missense_Mutation
SMAD4	p.D441G	1	7					■	COSM168813			Missense_Mutation
TP53	p.R267W	1	7	■			■		COSM179804,CO	rs55832599		Missense_Mutation
PANK3	p.I301F	1	7	■	■	■	■	■	COSM225568	rs77612793		Missense_Mutation
CNNM1	p.V681G	1	7	■	■	■	■	■	COSM227074,COSM4010914			Missense_Mutation
CHRNB1	p.M344T	1	7				■		COSM2979288	rs201776800		Missense_Mutation
TP53	p.R282W	2	7				■		COSM3378339,C	rs28934574		Missense_Mutation
UPB1	p.R326Q	1	7	■	■	■	■	■	COSM3424072	rs118163237		Missense_Mutation
PAX1	p.R171C	1	7		■			■	COSM356942			Missense_Mutation
CASR	p.R638C	1	7	■	■	■	■	■	COSM3586264			Missense_Mutation
KRT6B	p.K189N	3	7	■	■	■	■	■	COSM3998838	rs150221772		p
SLC28A2	p.L163W	4	7		■		■	■	COSM3999546	rs2271437		Missense_Mutation
TUBA8	p.Q235R	1	7		■	■	■		COSM4002088	rs2234333		Missense_Mutation
KRAS	p.G13D	1	7		■				COSM532,COSM1	rs112445441		Missense_Mutation
PMS2	p.Y313C	1	7		■	■	■	■	COSM747347	rs587782513		Missense_Mutation
SLC5A9	p.A473T	1	7			■			COSM910441			Missense_Mutation
ROR1	p.R736T	2	7				■					Missense_Mutation
HSD3B1	p.D242Y	1	7				■					Missense_Mutation
NVL	p.L560V	2	7		■							Missense_Mutation
FGFR2	p.F646V	1	7		■							Missense_Mutation
NELL1	p.C462G	1	7	■								Missense_Mutation
KCNA4	p.F228V	1	7					■				Missense_Mutation
PC	p.S603N	1	7				■					Missense_Mutation
RSF1	p.D182N	1	7		■							Missense_Mutation
GNPTAB	p.F946V	2	7		■							Missense_Mutation
GSC	p.R211C	2	7		■							Missense_Mutation
JAG2	p.C671W	2	7			■						Missense_Mutation
MYO1E	p.I459S	3	7					■				Missense_Mutation
FSD2	p.P424R	2	7			■						Missense_Mutation
STAT3	p.L387R	1	7				■					Missense_Mutation
MED13	p.S1956A	2	7					■				Missense_Mutation
HELZ	p.L463H	1	7				■					Missense_Mutation
TCF4	p.E443D	1	7	■								Missense_Mutation
STK11	p.D237N	2	7				■					Missense_Mutation
GGCX	p.L270P	1	7				■					Missense_Mutation
FAHD2B	p.T279I	1	7	■								Missense_Mutation
ECEL1	p.G666R	1	7		■							Missense_Mutation
CHRNG	p.P110L	1	7	■	■	■						Missense_Mutation
OTOR	p.G98C	1	7			■						Missense_Mutation
MAFB	p.R222S	1	7		■							Missense_Mutation
COL18A1	p.G802R	1	7			■						Missense_Mutation
PDGFB	p.C124Y	2	7		■							Missense_Mutation
ABHD6	p.K334N	2	7	■								Missense_Mutation
CP	p.L969P	1	7				■					Missense_Mutation

CRMP1	p.S255T	2	7									Missense_Mutation
HNRNPDL	p.D214H	2	7									Missense_Mutation
DCLK2	p.Q278R	2	7									Missense_Mutation
HARS2	p.L450F	1	7									Missense_Mutation
PBX2	p.R301H	1	7									Missense_Mutation
BRPF3	p.C232Y	2	7									Missense_Mutation
FIG4	p.T493I	2	7									Missense_Mutation
VNN1	p.F182C	4	7									Missense_Mutation
PHKG1	p.H142R	1	7									Missense_Mutation
SLC12A9	p.L316H	1	7									Missense_Mutation
FLNC	p.D827E	1	7									Missense_Mutation
FLNC	p.T1913N	1	7									Missense_Mutation
FLNC	p.F2114L	2	7									Missense_Mutation
ADCY8	p.W1111C	2	7									Missense_Mutation
SARDH	p.R726P	2	7									Missense_Mutation
ALAS2	p.G364A	1	7									Missense_Mutation
AR	p.K633T	1	7									Missense_Mutation
CLDN2	p.G17E	2	7									Missense_Mutation
PLD1	p.R90H	2	6						COSM1041303	rs149506327		Missense_Mutation
TYK2	p.R701T	1	6						COSM1189868	rs200791116		Missense_Mutation
SLC22A8	p.R75H	2	6						COSM1226068	rs148569801		Missense_Mutation
CLSPN	p.R298Q	1	6						COSM1342210,CO	rs115197921		Missense_Mutation
PTPN23	p.R1237C	1	6						COSM1423597			Missense_Mutation
CHP2	p.D128Y	2	6						COSM148052	rs79455352		Missense_Mutation
FRG1	p.D130V	5	6						COSM1485897			Missense_Mutation
AOX1	p.T755I	2	6						COSM149041	rs35217482		Missense_Mutation
DUPD1	p.P36S	1	6						COSM1580833	rs200149899		Missense_Mutation
MYO5C	p.R1405H	1	6						COSM1581287	rs149974542		Missense_Mutation
CRHR1	p.R225W	1	6						COSM160209	rs575488369		Missense_Mutation
IMP3	p.D44Y	1	6						COSM1608695	rs553010196		Missense_Mutation
CES1	p.G174D	3	6						COSM1609424	rs4784575		Missense_Mutation
FRG1	p.P140Q	2	6						COSM1666170	rs201142987		Missense_Mutation
NTN4	p.E59K	1	6						COSM167127			Missense_Mutation
ATP5C1	p.R277C	1	6						COSM179964	rs200847831		Missense_Mutation
SESN2	p.P87S	1	6						COSM254936	rs74896528		Missense_Mutation
OR51I2	p.R300C	1	6						COSM268431	rs202023944		Missense_Mutation
ST8SIA3	p.R188H	1	6						COSM2880458			Missense_Mutation
MED12	p.L36P	1	6						COSM3095235			Missense_Mutation
UBAC1	p.A321T	1	6						COSM328267			Missense_Mutation
MYO18B	p.S25L	1	6						COSM3405560	rs79294358		Missense_Mutation
DPH6	p.P236R	3	6						COSM3678171	rs10519996		Missense_Mutation
TBXAS1	p.P511L	2	6						COSM3685027,CO	rs13306050		Missense_Mutation
SURF1	p.D202H	1	6						COSM3685528	rs72619327		Missense_Mutation
PPEF2	p.H292R	1	6						COSM371542	rs202142922		Missense_Mutation
VWF	p.S1506L	2	6						COSM3746318	rs61750100		Missense_Mutation
TP53	p.V217G	1	6						COSM3787449,COSM3787448,COSM3787445,CO			Missense_Mutation
SDK2	p.R1172C	1	6						COSM3820452,CO	rs144170771		Missense_Mutation
ANKZF1	p.R297C	3	6						COSM3933539	rs57075420		Missense_Mutation
DDX11	p.P368S	1	6						COSM3954641,CO	rs201612562		Missense_Mutation
OR5W2	p.N286K	2	6						COSM3998470	rs143381179		Missense_Mutation

ANGPTL6	p.G435S	1	6						COSM4000494	rs150235799		Missense_Mutation
RGS12	p.R21Q	1	6						COSM4003064			Missense_Mutation
ASB10	p.R378W	1	6						COSM4004379,CO	rs61628535		Missense_Mutation
TM9SF4	p.G576R	1	6						COSM4097512,COSM2889064			Missense_Mutation
ZNF845	p.C945R	2	6						COSM475213			Missense_Mutation
KRAS	p.G12V	1	6						COSM520,COSM1	rs121913529		Missense_Mutation
PSD4	p.R566C	2	6						COSM72323	rs117870995		Missense_Mutation
GPR161	p.Y3H	1	6						COSM899170			Missense_Mutation
VWA8	p.R204C	2	6						COSM947504			Missense_Mutation
MAP3K3	p.R56Q	2	6						COSM1385172			Splice_Site
CLSTN1	p.R665L	1	6									Missense_Mutation
NIPAL3	p.T252A	2	6									Missense_Mutation
RRAGC	p.D294H	2	6									Missense_Mutation
ABCD3	p.C472F	1	6									Missense_Mutation
CDC14A	p.G121D	1	6									Missense_Mutation
WDR77	p.W17G	1	6									Missense_Mutation
CRB1	p.G573R	1	6									Missense_Mutation
SNAP47	p.P130Q	1	6									Missense_Mutation
LYST	p.L3766F	1	6									Missense_Mutation
AKR1C1	p.D50N	2	6									Missense_Mutation
TUBAL3	p.P182L	2	6									Missense_Mutation
RSU1	p.P217R	1	6									Missense_Mutation
OR13A1	p.L205V	1	6									Missense_Mutation
TLX1	p.S327L	2	6									Missense_Mutation
SH3PXD2A	p.T847P	1	6									Missense_Mutation
DMBT1	p.T2190I	2	6									Missense_Mutation
B4GALNT4	p.D256Y	2	6									Missense_Mutation
LRRC56	p.L143F	1	6									Missense_Mutation
TRPM5	p.L251M	1	6									Missense_Mutation
OR52A5	p.E183Q	1	6									Missense_Mutation
MICALCL	p.R660K	1	6									Missense_Mutation
ANO5	p.V749F	4	6									Missense_Mutation
LRP4	p.D1890E	1	6									Missense_Mutation
HYOU1	p.E312K	2	6									Missense_Mutation
HMBS	p.V202M	2	6									Missense_Mutation
HSPA8	p.I51M	2	6									Missense_Mutation
TEAD4	p.M257L	1	6									Missense_Mutation
KRT4	p.R440H	1	6									Missense_Mutation
SMUG1	p.L38F	1	6									Missense_Mutation
LUM	p.N169H	2	6									Missense_Mutation
FBXO21	p.R313W	2	6									Missense_Mutation
WBP4	p.D311Y	2	6									Missense_Mutation
MYCBP2	p.W4210R	1	6									Missense_Mutation
GAS6	p.C125S	2	6									Missense_Mutation
GAS6	p.D82A	1	6									Missense_Mutation
MYH7	p.D512G	1	6									Missense_Mutation
PLEKHG3	p.R739L	1	6									Missense_Mutation
EML5	p.H736P	1	6									Missense_Mutation
BCL11B	p.A233T	1	6									Missense_Mutation
BRF1	p.K262T	1	6									Missense_Mutation

GABRA5	p.T47M	2	6									Missense_Mutation
TMEM87A	p.G241V	2	6									Missense_Mutation
SECISBP2L	p.D206N	1	6									Missense_Mutation
PTPLAD1	p.F140L	1	6									Missense_Mutation
AP3B2	p.P127R	1	6									Missense_Mutation
MEIOB	p.L117P	2	6									Missense_Mutation
MSRB1	p.H39L	1	6									Missense_Mutation
SYNGR3	p.F120L	2	6									Missense_Mutation
TRAF7	p.T145M	2	6									Missense_Mutation
KCTD5	p.V157M	1	6									Missense_Mutation
CCDC135	p.R112G	1	6									Missense_Mutation
CDH8	p.W63C	2	6									Missense_Mutation
WWP2	p.D320E	2	6									Missense_Mutation
PLCG2	p.H1072R	1	6									Missense_Mutation
ITGAE	p.D658H	1	6									Missense_Mutation
ARHGEF15	p.Y507D	1	6									Missense_Mutation
KCNJ12	p.V13M	1	6									Missense_Mutation
SYNRG	p.H1235Y	1	6									Missense_Mutation
RND2	p.L74V	2	6									Missense_Mutation
NPEPPS	p.E353A	1	6									Missense_Mutation
AATK	p.E529K	1	6									Missense_Mutation
DLGAP1	p.D705Y	1	6									Missense_Mutation
APC2	p.P911L	1	6									Missense_Mutation
APC2	p.D1257N	1	6									Missense_Mutation
MYO9B	p.L1728R	1	6									Missense_Mutation
JAK3	p.I213F	1	6									Missense_Mutation
COMP	p.D469V	2	6									Missense_Mutation
TMEM91	p.A114V	1	6									Missense_Mutation
ZNF575	p.R145P	1	6									Missense_Mutation
FKRP	p.V173I	2	6									Missense_Mutation
KLK15	p.M108L	2	6									Missense_Mutation
NLRP4	p.P341S	1	6									Missense_Mutation
PUM2	p.D713N	1	6									Missense_Mutation
EFR3B	p.I278S	1	6									Missense_Mutation
BCL11A	p.C345R	1	6									Missense_Mutation
PSD4	p.M851I	2	6									Missense_Mutation
RND3	p.C125F	3	6									Missense_Mutation
CDCA7	p.D105N	2	6									Missense_Mutation
GPR155	p.I357S	1	6									Missense_Mutation
OSGEPL1	p.A229S	1	6									Missense_Mutation
SPHKAP	p.N1258K	2	6									Missense_Mutation
NMUR1	p.S360P	1	6									Missense_Mutation
ATRN	p.R1417W	1	6									Missense_Mutation
DEFB123	p.C25S	1	6									Missense_Mutation
TOP1	p.M247T	1	6									Missense_Mutation
PTPRT	p.D1204N	2	6									Missense_Mutation
PTPRT	p.Q479P	1	6									Missense_Mutation
PABPC1L	p.R231Q	1	6									Missense_Mutation
B4GALT5	p.Y246S	1	6									Missense_Mutation
ABHD16B	p.P313L	1	6									Missense_Mutation

SON	p.G440E	1	6									Missense_Mutation
TOP3B	p.M306I	2	6									Missense_Mutation
SGSM1	p.K138E	1	6									Missense_Mutation
MYO18B	p.L2271P	1	6									Missense_Mutation
KDELR3	p.S57C	1	6									Missense_Mutation
CHL1	p.V180L	1	6									Missense_Mutation
CTNNB1	p.R582W	1	6									Missense_Mutation
CXCR6	p.N112H	2	6									Missense_Mutation
KPNA1	p.C424R	1	6									Missense_Mutation
MCM2	p.S644F	1	6									Missense_Mutation
CPNE4	p.G237D	1	6									Missense_Mutation
SIAH2	p.L125Q	1	6									Missense_Mutation
GPR149	p.A316V	1	6									Missense_Mutation
GFM1	p.I645F	1	6									Missense_Mutation
OSTN	p.S95P	2	6									Missense_Mutation
LDB2	p.D47V	1	6									Missense_Mutation
GPR125	p.L1004H	1	6									Missense_Mutation
ARAP2	p.P472S	2	6									Missense_Mutation
NSUN7	p.R193M	1	6									Missense_Mutation
CNGA1	p.E260V	2	6									Missense_Mutation
SHROOM3	p.H1237R	1	6									Missense_Mutation
SFRP2	p.Y55N	2	6									Missense_Mutation
36894	p.I236N	1	6									Missense_Mutation
FRG1	p.A167T	1	6									Missense_Mutation
FBXO4	p.P148A	1	6									Missense_Mutation
OCLN	p.K504N	1	6									Missense_Mutation
CKMT2	p.S319L	2	6									Missense_Mutation
GPR98	p.E2446K	1	6									Missense_Mutation
SHROOM1	p.K763Q	2	6									Missense_Mutation
DDX41	p.F183S	1	6									Missense_Mutation
GNB2L1	p.S35F	2	6									Missense_Mutation
DDX39B	p.R123P	1	6									Missense_Mutation
BRD2	p.S820C	1	6									Missense_Mutation
TBCC	p.T276I	1	6									Missense_Mutation
ME1	p.V55F	2	6									Missense_Mutation
DCBLD1	p.S353Y	2	6									Missense_Mutation
UTRN	p.D2987E	2	6									Missense_Mutation
CNKSR3	p.S306C	1	6									Missense_Mutation
GET4	p.L65R	1	6									Missense_Mutation
INTS1	p.V1094A	1	6									Missense_Mutation
PHF14	p.T427R	1	6									Missense_Mutation
CRHR2	p.L318V	1	6									Missense_Mutation
DBNL	p.V20A	1	6									Missense_Mutation
SLC26A3	p.S211I	1	6									Missense_Mutation
PTPRZ1	p.E1306Q	1	6									Missense_Mutation
AASS	p.R318H	2	6									Missense_Mutation
INSIG1	p.P130R	2	6									Missense_Mutation
NCAPG2	p.W235C	1	6									Missense_Mutation
BIN3	p.L158P	1	6									Missense_Mutation
AGPAT6	p.D358N	1	6									Missense_Mutation

POLB	p.S275G	2	6									Missense_Mutation
SLC26A7	p.L84R	1	6									Missense_Mutation
RAD54B	p.E180Q	1	6									Missense_Mutation
SDC2	p.P16R	2	6									Missense_Mutation
PKHD1L1	p.L3386F	2	6									Missense_Mutation
CSMD3	p.M3681V	1	6									Missense_Mutation
UHRF2	p.T745K	1	6									Missense_Mutation
SH3GL2	p.G337A	2	6									Missense_Mutation
ANKS6	p.D52A	1	6									Missense_Mutation
OR13C8	p.H176D	1	6									Missense_Mutation
SUSD1	p.T389I	1	6									Missense_Mutation
TSC1	p.S278F	1	6									Missense_Mutation
CARD9	p.R305C	2	6									Missense_Mutation
PHF16	p.S246F	1	6									Missense_Mutation
KLF8	p.K171T	1	6									Missense_Mutation
HDAC8	p.S43F	2	6									Missense_Mutation
SLC16A2	p.P338S	2	6									Missense_Mutation
TBX22	p.A7V	2	6									Missense_Mutation
FAM46D	p.E205K	2	6									Missense_Mutation
ARMCX5	p.G444V	2	6									Missense_Mutation
TMEM57	p.E117K	1	6									Splice_Site
RYR1	p.T4882K	2	6									Splice_Site
CHRNA1	p.N115Y	2	6									Splice_Site
AGXT	p.I56S	1	6									Splice_Site
HACL1	p.G141V	2	6									Splice_Site
GABRR1	p.R191T	2	6									Splice_Site
NIPBL	p.M1I	1	6									Start_Codon_SNP
FRG1B	p.P72Q	5	5						COSM1025510,CO	rs145033899		Missense_Mutation
GATA3	p.A396T	1	5						COSM1163766	rs200935603		Missense_Mutation
TRAPPC12	p.M300V	1	5						COSM1185824	rs368230760		Missense_Mutation
NAGS	p.M169L	1	5						COSM1189486	rs569174201		Missense_Mutation
FOLR2	p.R78H	1	5						COSM1207386	rs193165102		Missense_Mutation
MYH14	p.R1920Q	1	5						COSM1216171			Missense_Mutation
TRIM71	p.R496H	2	5						COSM1230411	rs185519071		Missense_Mutation
GPR158	p.R525W	1	5						COSM1236038	rs143713985		Missense_Mutation
COL7A1	p.P2406L	2	5						COSM1238572	rs144788938		Missense_Mutation
CCDC86	p.R314Q	2	5						COSM1247562	rs140538227		Missense_Mutation
KMT2C	p.Q356E	1	5						COSM1312897,COSM1312896			Missense_Mutation
BAI3	p.E581K	2	5						COSM1445638			Missense_Mutation
RLF	p.S260C	1	5						COSM146478	rs61781760		Missense_Mutation
ALDH2	p.E504K	3	5						COSM147611	rs671		Missense_Mutation
ARHGAP5	p.E489K	5	5						COSM147763	rs78337553		Missense_Mutation
FRG1	p.G163E	5	5						COSM1485900			Missense_Mutation
ACAD9	p.K330Q	1	5						COSM149465	rs79530903		Missense_Mutation
TFAP4	p.T230P	2	5						COSM1519193			Missense_Mutation
ZNF638	p.S501L	2	5						COSM1647322,COSM721931			Missense_Mutation
GJB2	p.V27I	4	5						COSM1740036	rs2274084		Missense_Mutation
FNDC5	p.R174H	1	5						COSM2078449	rs140995767		Missense_Mutation
CSMD1	p.R1810Q	1	5						COSM229090,COSM229091,COSM3432341			Missense_Mutation
ADGB	p.A1066V	1	5						COSM3018156,CO	rs558119202		Missense_Mutation

OR10H1	p.A224D	5	5						COSM328549	rs62619246		Missense_Mutation
SLC4A4	p.G424D	1	5						COSM328669,CO	rs148635969		Missense_Mutation
ADPRHL2	p.D134N	2	5						COSM3419170			Missense_Mutation
SUCLA2	p.D367N	2	5						COSM3676601	rs117412559		Missense_Mutation
CHTF18	p.E851Q	2	5						COSM3680039,CO	rs77488103		Missense_Mutation
MYBBP1A	p.E612K	2	5						COSM3680482	rs78265875		Missense_Mutation
RWDD2B	p.R254C	1	5						COSM3681240	rs77085757		Missense_Mutation
THSD7B	p.R723Q	1	5						COSM3682417,CO	rs76693568		Missense_Mutation
THSD7A	p.L1550P	1	5						COSM3684984			Missense_Mutation
TBXAS1	p.P511L	2	5						COSM3685027,CO	rs13306050		Missense_Mutation
LRRC6	p.P288H	3	5						COSM3685319	rs76147813		Missense_Mutation
ZMIZ1	p.R47Q	2	5						COSM3738745	rs199706616		Missense_Mutation
FRG1B	p.A80P	2	5						COSM3748142,COSM477966,COSM477965			Missense_Mutation
EPPK1	p.T2258M	2	5						COSM3774327	rs200167763		Missense_Mutation
CYP2D6	p.Y355C	1	5						COSM393128,CO	rs202102799		Missense_Mutation
PPAN-P2RY11	p.T280A	2	5						COSM3932858,CO	rs143785583		Missense_Mutation
ATP1A4	p.R263Q	1	5						COSM3997013	rs76528638		Missense_Mutation
NLRP10	p.R243W	1	5						COSM3998598	rs59039403		Missense_Mutation
FUT2	p.I140F	3	5						COSM4000813	rs1047781		Missense_Mutation
TTC14	p.S495F	1	5						COSM4002524,CO	rs118079716		Missense_Mutation
RRH	p.T287I	1	5						COSM4002904	rs145995401		Missense_Mutation
ALPK1	p.E910D	1	5						COSM4002908	rs35308602		Missense_Mutation
TBXAS1	p.L70F	1	5						COSM4004324,CO	rs140463378		Missense_Mutation
CES1	p.T291M	1	5						COSM4129124	rs202001817		Missense_Mutation
MFSD12	p.Y182H	1	5						COSM439235,CO	rs2240751		Missense_Mutation
FRG1	p.N153D	4	5						COSM447708	rs200267221		Missense_Mutation
FGF23	p.R140W	2	5						COSM70777			Missense_Mutation
JMJD7-PLA2G4B	p.R203W	2	5						COSM961574,CO	rs146535143		Missense_Mutation
EPHA10	p.V1006M	3	5						COSM98890	rs78757428		Missense_Mutation
SVIL	p.R1173W	1	5						COSM3382832	rs142607117		Splice_Site
SRGAP2	p.R95H	2	5						COSM3773467,CO	rs2987927		Splice_Site
LTA	p.M1I	1	5						COSM124231			Start_Codon_SNP
PEX14	p.R366W	1	5									Missense_Mutation
TNFRSF1B	p.H375P	2	5									Missense_Mutation
UBR4	p.T2861A	1	5									Missense_Mutation
KIF17	p.R1001P	1	5									Missense_Mutation
HSPG2	p.P1129Q	1	5									Missense_Mutation
HSPG2	p.P1052H	2	5									Missense_Mutation
COL9A2	p.E91Q	1	5									Missense_Mutation
KDM4A	p.E991Q	2	5									Missense_Mutation
STIL	p.R724Q	1	5									Missense_Mutation
ZNF644	p.P1209Q	1	5									Missense_Mutation
HIPK1	p.L365F	1	5									Missense_Mutation
FLG2	p.L45M	1	5									Missense_Mutation
KIFAP3	p.L96V	1	5									Missense_Mutation
AXDND1	p.F266L	1	5									Missense_Mutation
IER5	p.R41H	2	5									Missense_Mutation
AHCTF1	p.C348F	1	5									Missense_Mutation
SVIL	p.P659S	1	5									Missense_Mutation
WDFY4	p.L1881P	2	5									Missense_Mutation

JMJD1C	p.Y1093F	2	5				■					Missense_Mutation
AFAP1L2	p.R667Q	2	5				■					Missense_Mutation
EMX2	p.A82P	1	5		■							Missense_Mutation
EBF3	p.A406V	1	5	■								Missense_Mutation
SCUBE2	p.N405S	1	5			■						Missense_Mutation
ANO1	p.A547T	1	5					■				Missense_Mutation
UCP2	p.N182S	1	5				■					Missense_Mutation
DLG2	p.K171N	2	5		■							Missense_Mutation
WNK1	p.P2116A	2	5				■					Missense_Mutation
CHD4	p.R1297Q	2	5	■								Missense_Mutation
C1R	p.R443C	2	5				■					Missense_Mutation
EMP1	p.T15S	2	5	■								Missense_Mutation
PTPRO	p.W283C	2	5	■	■	■		■				Missense_Mutation
BCAT1	p.L98S	1	5		■							Missense_Mutation
DENND5B	p.L793F	2	5			■						Missense_Mutation
HOXC13	p.D71V	1	5				■					Missense_Mutation
CAND1	p.C301S	1	5		■							Missense_Mutation
NAV3	p.K1759Q	4	5	■								Missense_Mutation
ATP2A2	p.D886Y	1	5					■				Missense_Mutation
BRAP	p.I496T	1	5		■							Missense_Mutation
RNFT2	p.L171M	1	5		■							Missense_Mutation
DHX37	p.P320S	1	5			■						Missense_Mutation
SFSWAP	p.F188I	1	5		■							Missense_Mutation
MEDAG	p.V159L	1	5	■	■	■		■				Missense_Mutation
FRY	p.S2432R	1	5		■							Missense_Mutation
CAB39L	p.G233V	1	5			■						Missense_Mutation
NALCN	p.H1189N	1	5		■							Missense_Mutation
ARHGEF40	p.P1395A	1	5				■					Missense_Mutation
HECTD1	p.R2137P	1	5				■					Missense_Mutation
SRP54	p.D199H	1	5					■				Missense_Mutation
HERC1	p.G1684W	2	5		■							Missense_Mutation
EFTUD1	p.N1092H	1	5	■								Missense_Mutation
MAN2A2	p.D160Y	1	5				■					Missense_Mutation
ADCY7	p.Q1032K	1	5			■						Missense_Mutation
FA2H	p.S171C	1	5	■		■	■					Missense_Mutation
WDR59	p.H70L	1	5									Missense_Mutation
TCF25	p.N60T	1	5		■							Missense_Mutation
KSR1	p.T137P	1	5				■					Missense_Mutation
SLC13A2	p.V533M	1	5				■					Missense_Mutation
CRLF3	p.R42W	1	5	■	■	■						Missense_Mutation
GGNBP2	p.E184K	1	5		■							Missense_Mutation
GGNBP2	p.E256A	1	5		■							Missense_Mutation
PLEKHH3	p.Y251C	1	5					■				Missense_Mutation
NME2	p.E267A	3	5			■						Missense_Mutation
CHMP1B	p.K27E	2	5	■								Missense_Mutation
DSG4	p.E381Q	2	5									Missense_Mutation
FSD1	p.R337L	2	5			■						Missense_Mutation
C3	p.I900M	1	5				■					Missense_Mutation
OR7C1	p.A119T	1	5		■							Missense_Mutation
GPATCH1	p.D145H	2	5	■								Missense_Mutation

TTC9B	p.A65S	1	5				■					Missense_Mutation
EHD2	p.E274K	1	5					■				Missense_Mutation
MYH14	p.A615S	2	5				■					Missense_Mutation
ALK	p.F921L	2	5				■					Missense_Mutation
THUMPD2	p.L313R	1	5					■				Missense_Mutation
BMP10	p.D309A	3	5		■							Missense_Mutation
AFF3	p.R1074Q	1	5			■						Missense_Mutation
IL18RAP	p.L389V	2	5		■							Missense_Mutation
MFSD9	p.L74F	2	5				■					Missense_Mutation
GPR39	p.I224M	1	5		■							Missense_Mutation
UBR3	p.K1355T	1	5			■						Missense_Mutation
TTN	p.L8270V	1	5	■								Missense_Mutation
TYW5	p.G54R	2	5			■						Missense_Mutation
ALS2	p.S1554L	1	5					■				Missense_Mutation
METTL21A	p.D36E	1	5		■							Missense_Mutation
ERBB4	p.K881Q	1	5					■				Missense_Mutation
GIGYF2	p.E1112K	1	5	■			■					Missense_Mutation
GBX2	p.S102C	2	5	■		■		■				Missense_Mutation
C2orf54	p.R28W	1	5			■						Missense_Mutation
HDLBP	p.L793F	2	5		■							Missense_Mutation
DTYMK	p.G144E	2	5			■						Missense_Mutation
SLC23A2	p.H413P	1	5				■					Missense_Mutation
FAM65C	p.D404V	2	5			■						Missense_Mutation
AURKA	p.G173V	1	5			■						Missense_Mutation
CABLES2	p.S152P	1	5	■								Missense_Mutation
NRIP1	p.K111N	1	5				■					Missense_Mutation
DOPEY2	p.D1674N	1	5		■							Missense_Mutation
HMGXB4	p.K33R	1	5					■				Missense_Mutation
MAPK11	p.G137R	1	5	■								Missense_Mutation
CNTN4	p.I972F	1	5					■				Missense_Mutation
OSBPL10	p.D133N	1	5		■							Missense_Mutation
CELSR3	p.G1190D	1	5					■				Missense_Mutation
ARHGEF3	p.Q482H	1	5					■				Missense_Mutation
MCM2	p.I771L	1	5			■						Missense_Mutation
EFCC1	p.R175S	1	5				■					Missense_Mutation
EPHB1	p.N739H	1	5		■							Missense_Mutation
PFN2	p.K69N	1	5		■							Missense_Mutation
IGSF10	p.P1773S	1	5					■				Missense_Mutation
PLCH1	p.E1501V	2	5			■						Missense_Mutation
GFM1	p.L646F	2	5				■					Missense_Mutation
KIAA0232	p.L1202V	1	5	■								Missense_Mutation
ZNF518B	p.E214A	1	5					■				Missense_Mutation
NSUN7	p.R193S	1	5					■				Missense_Mutation
SCARB2	p.F310Y	2	5		■							Missense_Mutation
CCNI	p.N342T	1	5	■								Missense_Mutation
LRBA	p.A2535G	1	5		■							Missense_Mutation
FRG1	p.A151T	2	5	■								Missense_Mutation
FRG1	p.K170Q	2	5		■							Missense_Mutation
MRPS30	p.L188P	1	5				■					Missense_Mutation
MIER3	p.E13A	5	5				■					Missense_Mutation

PCDHB2	p.V266D	1	5									Missense_Mutation
PBX2	p.E180K	1	5									Missense_Mutation
COL11A2	p.P1095L	2	5									Missense_Mutation
SLC39A7	p.G242R	2	5									Missense_Mutation
TEAD3	p.E140K	1	5									Missense_Mutation
MRPS10	p.T64R	1	5									Missense_Mutation
IBTK	p.L383F	2	5									Missense_Mutation
SLC35A1	p.V306L	1	5									Missense_Mutation
ORC3	p.I236L	2	5									Missense_Mutation
FABP7	p.K22Q	2	5									Missense_Mutation
TXLNB	p.R243G	1	5									Missense_Mutation
SYNE1	p.P5684L	2	5									Missense_Mutation
SCAF8	p.S22A	2	5									Missense_Mutation
THSD7A	p.P488T	1	5									Missense_Mutation
DNAH11	p.W1733C	1	5									Missense_Mutation
C7orf31	p.K555T	2	5									Missense_Mutation
CHN2	p.I43S	1	5									Missense_Mutation
RAMP3	p.N58K	1	5									Missense_Mutation
POM121C	p.E45G	1	5									Missense_Mutation
SRCRB4D	p.D371Y	2	5									Missense_Mutation
SRCRB4D	p.V61L	1	5									Missense_Mutation
GRM3	p.F652L	1	5									Missense_Mutation
METTL2B	p.L240Q	1	5									Missense_Mutation
FLNC	p.R2173P	2	5									Missense_Mutation
KIAA1549	p.T1002I	2	5									Missense_Mutation
RAB19	p.R57C	1	5									Missense_Mutation
MGAM	p.S456C	2	5									Missense_Mutation
SHH	p.A344T	1	5									Missense_Mutation
MYOM2	p.R470M	1	5									Missense_Mutation
CSMD1	p.P1992S	2	5									Missense_Mutation
SLC7A2	p.E140K	1	5									Missense_Mutation
DOCK5	p.Q1614H	1	5									Missense_Mutation
IDO1	p.R231H	1	5									Missense_Mutation
RB1CC1	p.G466E	2	5									Missense_Mutation
EPPK1	p.D1424A	1	5									Missense_Mutation
PLEC	p.E4314G	1	5									Missense_Mutation
CER1	p.K53T	1	5									Missense_Mutation
VCP	p.E756A	1	5									Missense_Mutation
DAPK1	p.K175I	2	5									Missense_Mutation
SYK	p.D221E	2	5									Missense_Mutation
SVEP1	p.Y1108S	1	5									Missense_Mutation
NTMT1	p.L199F	2	5									Missense_Mutation
C9orf96	p.V291M	2	5									Missense_Mutation
NOTCH1	p.P1227S	1	5									Missense_Mutation
TOR4A	p.H193Y	1	5									Missense_Mutation
CXorf38	p.R69G	1	5									Missense_Mutation
RLIM	p.R515T	1	5									Missense_Mutation
CENPI	p.I134L	1	5									Missense_Mutation
PDZD4	p.R414C	1	5									Missense_Mutation
FTCD	p.L468L	1	5									Silent

TMEM106A	p.T143M	1	5										Splice_Site
DTNA	p.R23S	1	5										Splice_Site
ZNF385D	p.G8C	2	5										Splice_Site
GABRA4	p.G241S	1	5										Splice_Site
PIK3R1	p.W220R	2	5										Splice_Site
GPRASP1	p.R1167W	1	4							COSM1112140	rs181661823		Missense_Mutation
CDC42BPG	p.R1237W	1	4							COSM1162171	rs55975541		Missense_Mutation
NOD2	p.R311W	1	4							COSM1172338	rs104895427		Missense_Mutation
SLC25A38	p.M288V	1	4							COSM1186239			Missense_Mutation
GHDC	p.R408W	1	4							COSM1189477,COSM1189478			Missense_Mutation
PEPD	p.R314H	1	4							COSM1220193	rs201447445		Missense_Mutation
TTN	p.R21639Q	2	4							COSM1288723,CO	rs373282633		Missense_Mutation
SEC14L6	p.R20Q	2	4							COSM1318776,CO	rs118116676		Missense_Mutation
IGSF3	p.R449C	2	4							COSM1333075,COSM1333076			Missense_Mutation
VPS37B	p.P241L	1	4							COSM1359694	rs370211610		Missense_Mutation
MFSD5	p.R25P	1	4							COSM1470573			Missense_Mutation
KDM3A	p.L520H	1	4							COSM148891,COS	rs186664065		Missense_Mutation
GAB2	p.R354P	2	4							COSM1492729			Missense_Mutation
TRMT11	p.T378M	2	4							COSM150179	rs79197242		Missense_Mutation
LRGUK	p.R552H	1	4							COSM150392	rs34890031		Missense_Mutation
WIF1	p.V82L	1	4							COSM1581945,CO	rs144873880		Missense_Mutation
CAND2	p.G752R	1	4							COSM1733089,CO	rs149084778		Missense_Mutation
RP11-156E8.1	p.R252G	1	4							COSM1734030			Missense_Mutation
FBN3	p.G2133S	1	4							COSM180004	rs138232748		Missense_Mutation
CCAR1	p.R327H	1	4							COSM2159552			Missense_Mutation
KMT2C	p.D348N	5	4							COSM228110,COS	rs201834857		Missense_Mutation
DOCK5	p.M987L	2	4							COSM249512	rs74894413		Missense_Mutation
PPP1R3B	p.R18C	2	4							COSM2790143	rs376926182		Missense_Mutation
KALRN	p.T1207M	1	4							COSM3057630,CO	rs139468141		Missense_Mutation
GPR35	p.Y278C	1	4							COSM320668			Missense_Mutation
DBH	p.T467M	1	4							COSM3213813	rs13306303		Missense_Mutation
OR6M1	p.S65Y	1	4							COSM322412			Missense_Mutation
FRG1B	p.A83T	3	4							COSM343576,COSM3748144,COSM343577			Missense_Mutation
LIPK	p.R252C	2	4							COSM3441339,CO	rs376096296		Missense_Mutation
CDH9	p.E375K	1	4							COSM3615286			Missense_Mutation
FBXO34	p.G704V	1	4							COSM3677967	rs10138395		Missense_Mutation
C2orf53	p.R222C	3	4							COSM3682625	rs3739097		Missense_Mutation
HBS1L	p.M174V	1	4							COSM3683966	rs79687747		Missense_Mutation
SLIT3	p.E994G	1	4							COSM371452	rs2305993		Missense_Mutation
PANK2	p.L111Q	1	4							COSM3749703	rs71647828		Missense_Mutation
MANEA	p.F344I	2	4							COSM3774267	rs35772543		Missense_Mutation
RHCG	p.G352S	2	4							COSM3786595	rs139788541		Missense_Mutation
TUBB2B	p.A248V	2	4							COSM3830204			Missense_Mutation
OXCT2	p.D267G	3	4							COSM3930778	rs112946539		Missense_Mutation
HELZ2	p.T2566A	1	4							COSM39333	rs114036943		Missense_Mutation
C8orf74	p.C145F	1	4							COSM3982347	rs77073793		Missense_Mutation
ANKHD1	p.N1760S	1	4							COSM4003302,CO	rs3752704		Missense_Mutation
CCHCR1	p.Q575H	3	4							COSM4003907,CO	rs147733073		Missense_Mutation
RSF1	p.P1133L	1	4							COSM4036875	rs200271333		Missense_Mutation
ADAMTS9	p.R209C	1	4							COSM446978	rs140444427		Missense_Mutation

FRG1	p.C159Y	3	4				■		COSM586784			Missense_Mutation
EPHB3	p.A217T	1	4				■		COSM98894	rs187877110		Missense_Mutation
VIT	p.D291N	2	4	■		■		■	COSM1531993			Splice_Site
CDHR2	p.R1092W	1	4	■	■	■		■	COSM3697151			Splice_Site
PLCH2	p.R1359P	1	4			■						Missense_Mutation
RNF207	p.E462K	2	4			■						Missense_Mutation
EPHA8	p.A866T	1	4				■					Missense_Mutation
ZSCAN20	p.N542S	1	4		■	■						Missense_Mutation
CLDN19	p.S204F	1	4	■								Missense_Mutation
ZSWIM5	p.T851A	1	4					■				Missense_Mutation
DMBX1	p.Q30K	1	4					■				Missense_Mutation
TGFBR3	p.P194S	1	4			■						Missense_Mutation
WNT2B	p.R94C	1	4				■					Missense_Mutation
CSDE1	p.I257M	2	4		■							Missense_Mutation
CSDE1	p.M226I	1	4		■							Missense_Mutation
ANKRD35	p.T908M	1	4				■					Missense_Mutation
C1orf189	p.E99D	2	4			■						Missense_Mutation
TDRD10	p.P308T	2	4					■				Missense_Mutation
FCRLB	p.D229H	1	4		■	■						Missense_Mutation
SCYL3	p.G733W	1	4			■						Missense_Mutation
CNTN2	p.A406T	1	4		■							Missense_Mutation
PGBD5	p.R344C	2	4	■								Missense_Mutation
PLD5	p.S257T	2	4					■				Missense_Mutation
AHCTF1	p.I898S	1	4		■							Missense_Mutation
ZNF438	p.P804S	1	4				■					Missense_Mutation
ZNF485	p.L313F	2	4			■	■	■				Missense_Mutation
FGFR2	p.P180Q	1	4					■				Missense_Mutation
MUC6	p.G948E	2	4			■						Missense_Mutation
METTL15	p.R397L	1	4									Missense_Mutation
OR4A16	p.P127T	1	4	■	■	■	■	■				Missense_Mutation
CYB561A3	p.T64P	2	4					■				Missense_Mutation
SLC25A45	p.T32I	2	4				■					Missense_Mutation
SCYL1	p.E55K	2	4					■				Missense_Mutation
INPPL1	p.Q589P	1	4				■					Missense_Mutation
MYO7A	p.T1992M	1	4				■					Missense_Mutation
DYNC2H1	p.T1857A	2	4					■				Missense_Mutation
PATE2	p.L109H	2	4				■					Missense_Mutation
NCAPD2	p.R1215H	2	4			■						Missense_Mutation
CLEC9A	p.P112R	1	4				■					Missense_Mutation
PTPRO	p.P284S	2	4	■	■	■		■				Missense_Mutation
PPFIBP1	p.D7G	1	4			■						Missense_Mutation
TMTC1	p.L205F	2	4		■							Missense_Mutation
ERBB3	p.D112N	1	4				■					Missense_Mutation
NTN4	p.T572M	1	4				■					Missense_Mutation
ERP29	p.R76H	2	4				■					Missense_Mutation
GCN1L1	p.Y1643N	1	4		■							Missense_Mutation
RBM26	p.Y448D	1	4	■	■	■		■				Missense_Mutation
CLDN10	p.S68T	1	4			■						Missense_Mutation
ERCC5	p.E727K	1	4			■						Missense_Mutation
SLC10A2	p.S35R	1	4	■								Missense_Mutation

NRXN3	p.S684A	4	4					■				Missense_Mutation
RTL1	p.R285P	1	4	■								Missense_Mutation
USP8	p.E46Q	1	4			■						Missense_Mutation
MYO9A	p.D2053Y	1	4			■						Missense_Mutation
ACSBG1	p.I562L	1	4			■						Missense_Mutation
FAM154B	p.D254H	1	4				■					Missense_Mutation
BLM	p.N1112H	1	4				■					Missense_Mutation
KIFC3	p.N140D	1	4			■						Missense_Mutation
ZFHX3	p.D2812G	1	4	■								Missense_Mutation
PLCG2	p.A1070G	1	4	■								Missense_Mutation
PRPF8	p.S2205Y	1	4		■							Missense_Mutation
OR1D2	p.R292S	1	4					■				Missense_Mutation
ANKFY1	p.V592A	1	4			■						Missense_Mutation
UNC119	p.D87N	2	4				■					Missense_Mutation
EFCAB5	p.G1073E	1	4					■				Missense_Mutation
CPD	p.N1142Y	2	4				■					Missense_Mutation
KRT10	p.G507R	3	4	■								Missense_Mutation
KRT40	p.L292P	1	4				■					Missense_Mutation
DGKE	p.H536Q	1	4				■					Missense_Mutation
RALBP1	p.S343A	3	4			■						Missense_Mutation
KIAA1468	p.Q883H	1	4		■							Missense_Mutation
TNFRSF11A	p.M540L	1	4	■	■	■		■				Missense_Mutation
ODF3L2	p.R193P	1	4				■					Missense_Mutation
NCLN	p.T70M	1	4		■							Missense_Mutation
PLIN4	p.T289N	1	4			■						Missense_Mutation
KEAP1	p.M161I	1	4				■					Missense_Mutation
ZNF700	p.F287L	5	4					■				Missense_Mutation
ZNF737	p.S48A	2	4					■				Missense_Mutation
URI1	p.R46T	2	4				■					Missense_Mutation
CYP2B6	p.G440V	2	4				■					Missense_Mutation
STRN4	p.T479M	1	4	■								Missense_Mutation
RPL13A	p.R94Q	2	4					■				Missense_Mutation
KLK10	p.Y184H	1	4	■								Missense_Mutation
KCNF1	p.E431K	1	4				■					Missense_Mutation
PUM2	p.R709K	1	4				■					Missense_Mutation
MFSD2B	p.V380G	1	4			■						Missense_Mutation
BIRC6	p.G2611A	1	4				■					Missense_Mutation
THUMPD2	p.L309P	1	4					■				Missense_Mutation
FSHR	p.V481L	2	4					■				Missense_Mutation
USP34	p.L3069V	1	4	■								Missense_Mutation
CTNNA2	p.N351H	3	4					■				Missense_Mutation
SFT2D3	p.R76L	1	4					■				Missense_Mutation
NEB	p.R8248H	1	4		■							Missense_Mutation
FASTKD1	p.Q481K	1	4					■				Missense_Mutation
UBR3	p.K1355T	1	4				■					Missense_Mutation
NBEAL1	p.C484F	2	4					■				Missense_Mutation
PTPRN	p.E355K	2	4			■						Missense_Mutation
COL4A4	p.P1426A	1	4					■				Missense_Mutation
RIN2	p.M771L	3	4	■								Missense_Mutation
FRG1B	p.A99T	1	4	■								Missense_Mutation

FRG1B	p.G105E	1	4									Missense_Mutation
RALGAPB	p.I352F	1	4									Missense_Mutation
NRIP1	p.D103N	1	4									Missense_Mutation
GART	p.I7T	1	4									Missense_Mutation
RUNX1	p.L472P	1	4									Missense_Mutation
IL17RA	p.M483I	2	4									Missense_Mutation
NEFH	p.E170K	1	4									Missense_Mutation
SLC35E4	p.A174T	1	4									Missense_Mutation
FAM118A	p.G289R	1	4									Missense_Mutation
PKDREJ	p.S1244Y	1	4									Missense_Mutation
TGM4	p.P337L	1	4									Missense_Mutation
SETD2	p.N1535T	1	4									Missense_Mutation
DOCK3	p.N483K	1	4									Missense_Mutation
FRMD4B	p.K562E	1	4									Missense_Mutation
NDUFB4	p.S9L	1	4									Missense_Mutation
TNK2	p.H182R	1	4									Missense_Mutation
PDGFRA	p.S911N	2	4									Missense_Mutation
SRD5A3	p.V281A	1	4									Missense_Mutation
UGT2B11	p.S34N	2	4									Missense_Mutation
DCHS2	p.T2350S	1	4									Missense_Mutation
DCHS2	p.P2345Q	1	4									Missense_Mutation
TENM3	p.K1784M	1	4									Missense_Mutation
NIPBL	p.E2496Q	2	4									Missense_Mutation
MRPS36	p.M2V	1	4									Missense_Mutation
DMGDH	p.S448L	2	4									Missense_Mutation
CMYA5	p.K2590E	1	4									Missense_Mutation
CHSY3	p.R187L	1	4									Missense_Mutation
NME5	p.V42F	1	4									Missense_Mutation
PCDH1	p.T105S	1	4									Missense_Mutation
SFXN1	p.F274S	2	4									Missense_Mutation
HIVEP1	p.S354C	1	4									Missense_Mutation
OR2W1	p.I256L	2	4									Missense_Mutation
DST	p.S862G	2	4									Missense_Mutation
PTPRK	p.Q1406E	2	4									Missense_Mutation
GRM1	p.P1015H	2	4									Missense_Mutation
CHST12	p.V101M	2	4									Missense_Mutation
AP5Z1	p.P79T	2	4									Missense_Mutation
TMEM196	p.E154D	1	4									Missense_Mutation
FKBP9	p.H567Q	3	4									Missense_Mutation
PCLO	p.K556N	2	4									Missense_Mutation
SLC12A9	p.E331G	1	4									Missense_Mutation
CNTNAP2	p.S75A	1	4									Missense_Mutation
GIMAP8	p.R81C	1	4									Missense_Mutation
KMT2C	p.K339N	2	4									Missense_Mutation
DPP6	p.P22L	1	4									Missense_Mutation
SHH	p.A227G	1	4									Missense_Mutation
SNTG1	p.N229S	2	4									Missense_Mutation
RGS20	p.K228Q	3	4									Missense_Mutation
PDE7A	p.R91P	1	4									Missense_Mutation
VPS13B	p.S1393F	2	4									Missense_Mutation

PLEC	p.E4314D	1	4									Missense_Mutation
APBA1	p.E15Q	1	4									Missense_Mutation
RNF183	p.R161C	2	4									Missense_Mutation
COL27A1	p.R986L	1	4									Missense_Mutation
OR1K1	p.V226M	1	4									Missense_Mutation
RABGAP1	p.N1005Y	1	4									Missense_Mutation
GARNL3	p.S847P	2	4									Missense_Mutation
SPTAN1	p.A2135T	1	4									Missense_Mutation
ZDHHC12	p.W22C	1	4									Missense_Mutation
QSOX2	p.V71E	2	4									Missense_Mutation
ABCA2	p.V804M	2	4									Missense_Mutation
ZNF630	p.K277T	1	4									Missense_Mutation
LONRF3	p.E543A	1	4									Missense_Mutation
OR13H1	p.L153R	1	4									Missense_Mutation
CTD-2192J16.22	p.G144G	1	4									Silent
DCDC1	p.Q1249H	1	4									Splice_Site
PZP	p.Y253D	1	4									Splice_Site
LRMP	p.G82C	1	4									Splice_Site
SIGLEC1	p.G1260R	1	4									Splice_Site
LAMB2	p.R469Q	1	4									Splice_Site
PDZD11	p.K76N	1	4									Splice_Site
FRG1	p.L150S	1	3						COSM1131141	rs575384276		Missense_Mutation
TAS1R1	p.T139M	1	3						COSM1158344	rs141817074		Missense_Mutation
SLC9C1	p.G114S	1	3						COSM1226593			Missense_Mutation
CCDC81	p.E443K	1	3						COSM1247556,CO	rs148008941		Missense_Mutation
RPRD2	p.G1359S	2	3						COSM1289977,CO	rs79565099		Missense_Mutation
USP6	p.I67M	2	3						COSM1315964,COSM1315965			Missense_Mutation
PLXDC2	p.H479Y	2	3						COSM1347203			Missense_Mutation
CATSPER1	p.G244R	3	3						COSM1356129	rs79062509		Missense_Mutation
SLC3A1	p.R229W	1	3						COSM1408181			Missense_Mutation
TRIOBP	p.P2103L	3	3						COSM1416184,CO	rs200250028		Missense_Mutation
FLG	p.H519N	2	3						COSM146619	rs12036682		Missense_Mutation
FBRSL1	p.P515L	1	3						COSM147638	rs117416619		Missense_Mutation
STARD9	p.G4420R	1	3						COSM147891	rs143402747		Missense_Mutation
CBLB	p.N466D	1	3						COSM149439	rs61758360		Missense_Mutation
PRSS38	p.R159C	2	3						COSM1501379	rs200455452		Missense_Mutation
C7orf10	p.I167L	2	3						COSM150273	rs138102615		Missense_Mutation
CAPZA3	p.R39H	1	3						COSM159745	rs117330813		Missense_Mutation
MUC5B	p.T4408M	5	3						COSM1599909,CO	rs201822078		Missense_Mutation
ZNF678	p.C333W	1	3						COSM1601770,COSM1601769			Missense_Mutation
INO80E	p.L203P	1	3						COSM1609193			Missense_Mutation
FRG2C	p.G154R	3	3						COSM1661909	rs201191365		Missense_Mutation
MBLAC1	p.S43C	1	3						COSM166642	rs78225095		Missense_Mutation
GNGT2	p.E7K	1	3						COSM1710443			Missense_Mutation
TGM7	p.T577M	1	3						COSM188115	rs369585501		Missense_Mutation
SYT15	p.E400K	1	3						COSM1958027	rs535884152		Missense_Mutation
CELA1	p.V3G	2	3						COSM2009302			Missense_Mutation
SLC1A2	p.R8Q	1	3						COSM2115833			Missense_Mutation
TAS2R30	p.A227V	1	3						COSM223519	rs200661425		Missense_Mutation
KRTAP9-9	p.S161Y	4	3						COSM225052	rs77134625		Missense_Mutation

FRG1	p.S169N	4	3	■					COSM225855			Missense_Mutation
FRG1	p.I132T	5	3			■		■	COSM228976	rs149113074		Missense_Mutation
MARCO	p.V520I	1	3	■	■	■	■	■	COSM271379	rs200590124		Missense_Mutation
LYPD2	p.E47K	1	3	■	■	■	■	■	COSM2867669	rs145933616		Missense_Mutation
MOCOS	p.S310L	2	3	■	■	■	■	■	COSM288341	rs77940522		Missense_Mutation
CAND2	p.L965V	1	3	■		■			COSM3059732,CO	rs191057169		Missense_Mutation
WNK3	p.G515W	2	3		■		■		COSM3092716			Missense_Mutation
ANKH	p.V87I	1	3	■					COSM3211002			Missense_Mutation
CENPT	p.S121A	2	3			■			COSM3679961,CO	rs187620725		Missense_Mutation
PYGB	p.T514S	2	3	■		■		■	COSM3681168	rs79534510		Missense_Mutation
SFI1	p.E695K	2	3				■		COSM3681418	rs188434444		Missense_Mutation
SCARB2	p.G319E	2	3	■					COSM3683314	rs117157204		Missense_Mutation
NOP2	p.D133N	1	3	■	■	■	■	■	COSM3688405	rs75316694		Missense_Mutation
FAM214A	p.V418M	1	3		■	■	■	■	COSM3690439			Missense_Mutation
KRTAP1-1	p.P58R	1	3	■	■	■	■	■	COSM3717343	rs149188249		Missense_Mutation
FRG1B	p.I110M	5	3				■		COSM3740068,COSM3740069,COSM3740067			Missense_Mutation
TMEM247	p.R129Q	2	3	■				■	COSM3771099	rs79819367		Missense_Mutation
FRG1B	p.N85D	5	3			■			COSM3783477,COSM477968,COSM477967			Missense_Mutation
PDPR	p.N611I	1	3		■	■	■		COSM3818399	rs7192962		Missense_Mutation
CDRT1	p.I23N	3	3				■		COSM3818868,CO	rs201445711		Missense_Mutation
SPINK14	p.T53M	1	3		■				COSM3852545	rs189357431		Missense_Mutation
MUC6	p.T1795I	5	3	■			■		COSM3927662,COSM3927661			Missense_Mutation
CNTNAP3B	p.V275I	1	3		■			■	COSM3930083	rs201844561		Missense_Mutation
CCAR2	p.Q603H	2	3		■				COSM3982473	rs59511580		Missense_Mutation
SHC1	p.A205V	2	3	■	■		■		COSM3996961,CO	rs8191981		Missense_Mutation
TPGS2	p.R47C	3	3		■		■		COSM4000400,CO	rs2303507		Missense_Mutation
CYP2A6	p.I471T	5	3					■	COSM4000741	rs5031016		Missense_Mutation
MUC16	p.P4142L	3	3		■			■	COSM4001071,CO	rs17000819		Missense_Mutation
OBSL1	p.R340C	1	3				■	■	COSM4091793,CO	rs199799438		Missense_Mutation
KRT10	p.S546R	2	3					■	COSM4129976			Missense_Mutation
CPNE7	p.P558L	2	3			■	■	■	COSM435940	rs3751682		Missense_Mutation
CENPF	p.S1993C	2	3			■		■	COSM463922			Missense_Mutation
KRTAP4-9	p.S133R	1	3	■	■		■	■	COSM560842,COS	rs370251849		Missense_Mutation
NLRP11	p.R534C	1	3	■	■				COSM98150	rs115243845		Missense_Mutation
OBSCN	p.A5468T	1	3	■					COSM99096	rs139319906		Missense_Mutation
GALNT9	p.R399R	1	3	■	■	■			COSM2001435,CO	rs544376030		Silent
IKBKB	p.R526Q	2	3	■	■	■	■	■	COSM150490	rs2272736		Splice_Site
RABGAP1	p.K598R	1	3	■		■	■	■	COSM150707,COS	rs75992542		Splice_Site
MIB2	p.D835V	1	3				■					Missense_Mutation
COL9A2	p.E91Q	1	3				■					Missense_Mutation
DPH2	p.V149M	1	3			■						Missense_Mutation
NRD1	p.E395Q	1	3			■						Missense_Mutation
ADORA3	p.Q320K	2	3				■					Missense_Mutation
TRIM33	p.R136H	2	3		■							Missense_Mutation
FLG	p.D2896H	1	3					■				Missense_Mutation
LCE4A	p.G84D	2	3		■							Missense_Mutation
COPA	p.Q1199R	1	3				■					Missense_Mutation
FCRLB	p.D229H	1	3		■	■						Missense_Mutation
IER5	p.E74Q	1	3				■					Missense_Mutation
ZNF281	p.P86R	2	3	■								Missense_Mutation

OR2T10	p.F198C	1	3									Missense_Mutation
KIAA1462	p.D68V	1	3									Missense_Mutation
DMBT1	p.V2284A	1	3									Missense_Mutation
ALX4	p.T283N	1	3									Missense_Mutation
OR4C11	p.Q17H	2	3									Missense_Mutation
OR5M10	p.A180V	1	3									Missense_Mutation
OR5AK2	p.S291R	3	3									Missense_Mutation
B3GNT6	p.G331A	1	3									Missense_Mutation
CCDC81	p.R335L	1	3									Missense_Mutation
NPAT	p.E691D	1	3									Missense_Mutation
NCAPD2	p.R182C	1	3									Missense_Mutation
C12orf77	p.A93V	1	3									Missense_Mutation
TMTC1	p.W79L	1	3									Missense_Mutation
PRICKLE1	p.Q283R	1	3									Missense_Mutation
KMT2D	p.P1097R	1	3									Missense_Mutation
HOXC6	p.G210A	1	3									Missense_Mutation
HOXC6	p.G212A	1	3									Missense_Mutation
MLXIP	p.T134P	1	3									Missense_Mutation
ATP8A2	p.L256V	1	3									Missense_Mutation
LRRC63	p.I490S	1	3									Missense_Mutation
PCDH8	p.R302Q	2	3									Missense_Mutation
EDNRB	p.P177S	2	3									Missense_Mutation
ERCC5	p.E887K	1	3									Missense_Mutation
IRS2	p.R1097H	2	3									Missense_Mutation
ATP11A	p.F1056V	1	3									Missense_Mutation
JKAMP	p.M66T	1	3									Missense_Mutation
CATSPERB	p.T323N	1	3									Missense_Mutation
YY1	p.M83T	2	3									Missense_Mutation
AHNAK2	p.S245F	1	3									Missense_Mutation
RYR3	p.A2933T	2	3									Missense_Mutation
SLC24A5	p.E253V	1	3									Missense_Mutation
HERC1	p.R2997I	1	3									Missense_Mutation
ANKDD1A	p.G291S	1	3									Missense_Mutation
HCN4	p.R949W	2	3									Missense_Mutation
MSLNL	p.Y734S	1	3									Missense_Mutation
UBN1	p.R117C	1	3									Missense_Mutation
BCL7C	p.I25T	1	3									Missense_Mutation
HYDIN	p.K1421M	1	3									Missense_Mutation
BCAR1	p.L297M	1	3									Missense_Mutation
ADAD2	p.S148G	1	3									Missense_Mutation
PRDM7	p.G403V	2	3									Missense_Mutation
MYO1C	p.V294A	1	3									Missense_Mutation
KDM6B	p.R340P	1	3									Missense_Mutation
ARHGEF15	p.R250P	1	3									Missense_Mutation
COX10	p.E335K	1	3									Missense_Mutation
NLK	p.A77V	1	3									Missense_Mutation
KRT15	p.G99V	2	3									Missense_Mutation
CTAGE1	p.E342Q	1	3									Missense_Mutation
ASXL3	p.S729T	1	3									Missense_Mutation
CNDP1	p.S135R	1	3									Missense_Mutation

TMPRSS9	p.E1017D	1	3									Missense_Mutation
ZNF627	p.E438A	4	3									Missense_Mutation
ZNF44	p.E348A	4	3									Missense_Mutation
ZNF492	p.Y393F	1	3									Missense_Mutation
RYR1	p.R274H	1	3									Missense_Mutation
NOVA2	p.A323T	1	3									Missense_Mutation
SAE1	p.S95C	1	3									Missense_Mutation
PRR12	p.G673V	1	3									Missense_Mutation
ZNF468	p.H233R	1	3									Missense_Mutation
PRPF31	p.T410I	1	3									Missense_Mutation
KIR2DL3	p.S148F	2	3									Missense_Mutation
GREB1	p.S1226W	1	3									Missense_Mutation
APOB	p.N3051S	1	3									Missense_Mutation
ITSN2	p.L1245V	1	3									Missense_Mutation
EGR4	p.R187Q	1	3									Missense_Mutation
ALMS1	p.V1919F	2	3									Missense_Mutation
PROM2	p.V768L	2	3									Missense_Mutation
NMI	p.D192Y	1	3									Missense_Mutation
TMEM74B	p.G213D	1	3									Missense_Mutation
PLCB1	p.S291T	1	3									Missense_Mutation
XRN2	p.R281I	2	3									Missense_Mutation
FRG1B	p.H173Y	1	3									Missense_Mutation
CCM2L	p.Q88K	2	3									Missense_Mutation
SAMHD1	p.N504S	2	3									Missense_Mutation
LBP	p.Y431S	2	3									Missense_Mutation
SPATA2	p.G206S	2	3									Missense_Mutation
AP000322.53	p.C104Y	2	3									Missense_Mutation
GAB4	p.Q235H	2	3									Missense_Mutation
ZNF280B	p.S225L	1	3									Missense_Mutation
MKL1	p.M429L	2	3									Missense_Mutation
ARFGAP3	p.D386G	1	3									Missense_Mutation
KIF15	p.D748E	1	3									Missense_Mutation
ZNF717	p.T508I	1	3									Missense_Mutation
ROBO1	p.L27R	2	3									Missense_Mutation
SIDT1	p.V197M	1	3									Missense_Mutation
EEFSEC	p.A193T	1	3									Missense_Mutation
PLCH1	p.G424A	1	3									Missense_Mutation
OTOL1	p.K235R	1	3									Missense_Mutation
VPS8	p.S125F	1	3									Missense_Mutation
C4orf21	p.M2023T	1	3									Missense_Mutation
FAT4	p.D3275V	1	3									Missense_Mutation
FAT4	p.G4885V	1	3									Missense_Mutation
FRG1	p.E166D	5	3									Missense_Mutation
FAM173B	p.R132Q	1	3									Missense_Mutation
MROH2B	p.T492I	1	3									Missense_Mutation
ZFYVE16	p.S637F	1	3									Missense_Mutation
DOCK2	p.Q869K	1	3									Missense_Mutation
BTN2A1	p.L189V	2	3									Missense_Mutation
HIVEP2	p.E649V	2	3									Missense_Mutation
PHACTR2	p.S7C	1	3									Missense_Mutation

SCRN1	p.S280A	1	3			■						Missense_Mutation
NPSR1	p.E361Q	1	3		■							Missense_Mutation
HIP1	p.D446A	1	3		■							Missense_Mutation
ABCB4	p.A791S	1	3			■						Missense_Mutation
CNOT4	p.L469S	2	3		■							Missense_Mutation
CSMD1	p.P1992S	2	3			■						Missense_Mutation
ASAH1	p.T273S	1	3	■		■						Missense_Mutation
WHSC1L1	p.R1309T	1	3	■								Missense_Mutation
TRPA1	p.Q1070R	1	3					■				Missense_Mutation
TRAPPC9	p.N645H	2	3			■						Missense_Mutation
DENND3	p.D517E	2	3			■						Missense_Mutation
MAPK15	p.L15F	1	3	■								Missense_Mutation
FOCAD	p.G374R	1	3				■					Missense_Mutation
GNAQ	p.K311N	2	3			■						Missense_Mutation
FAM120AOS	p.V235F	1	3			■						Missense_Mutation
SARDH	p.V470I	1	3	■								Missense_Mutation
WWC3	p.C954R	1	3			■						Missense_Mutation
UBA1	p.K268N	1	3	■								Missense_Mutation
FOXR2	p.L131V	1	3		■							Missense_Mutation
MAGEE1	p.P926S	2	3				■					Missense_Mutation
SYTL4	p.R81Q	1	3					■				Missense_Mutation
TENM1	p.L850H	1	3	■		■	■					Missense_Mutation
SYDE2	p.E639*	2	3				■					Nonsense_Mutation
SATB1	p.E570*	2	3					■				Nonsense_Mutation
UNC5D	p.E198*	2	3					■				Nonsense_Mutation
CACNA1E	p.P89S	2	3				■					Splice_Site
STRC	p.V1103D	1	3					■				Splice_Site
EPOR	p.E84Q	1	3			■						Splice_Site
FRG1	p.N144I	2	3				■					Splice_Site
MAP3K7	p.G431D	1	3				■					Splice_Site
PCDHGB1	p.A555V	1	2	■					COSM1063061			Missense_Mutation
KRBA1	p.L738V	2	2		■				COSM1187462	rs376516900		Missense_Mutation
KLK11	p.G122S	1	2	■					COSM1256010	rs191149606		Missense_Mutation
IGSF9B	p.T178M	2	2	■	■	■	■	■	COSM1352724	rs77432041		Missense_Mutation
PALM3	p.A643V	2	2		■				COSM1390894			Missense_Mutation
CDKN2A	p.H15R	1	2	■					COSM14253			Missense_Mutation
HLA-A	p.A48S	1	2		■				COSM1442949	rs41552219		Missense_Mutation
HLA-A	p.W191G	4	2		■				COSM1442956			Missense_Mutation
MICALL2	p.K623R	1	2		■			■	COSM1448899	rs61287564		Missense_Mutation
PTAFR	p.A224D	1	2	■	■	■	■	■	COSM146455	rs5938		Missense_Mutation
OR5D18	p.V118M	3	2	■	■	■	■	■	COSM147209	rs11231180		Missense_Mutation
ATP8A2	p.A1069T	1	2			■			COSM147653	rs2296242		Missense_Mutation
RNF157	p.A73S	1	2		■		■		COSM148346	rs145150447		Missense_Mutation
FRG1	p.I157V	1	2					■	COSM1485899	rs561930100		Missense_Mutation
APEH	p.Q572H	1	2					■	COSM149392	rs75318799		Missense_Mutation
POLQ	p.V310G	2	2				■		COSM149454	rs55748151		Missense_Mutation
ROPN1B	p.L197V	1	2		■				COSM149461	rs60516581		Missense_Mutation
TMEM168	p.G659S	1	2				■		COSM150374	rs77145942		Missense_Mutation
CYLC2	p.K190E	2	2		■	■		■	COSM150652	rs2298050		Missense_Mutation
RBMXL3	p.D616N	1	2				■		COSM1569753			Missense_Mutation

FAM104B	p.T112K	1	2						COSM1579621			Missense_Mutation
ZNF474	p.S78L	1	2						COSM1581971	rs114823456		Missense_Mutation
PRSS3	p.F150S	1	2						COSM1599960,COSM1599959,COSM1599958			Missense_Mutation
KRT10	p.S547Y	2	2						COSM1610197			Missense_Mutation
ODF3L2	p.A192P	1	2						COSM1612429			Missense_Mutation
KIAA1244	p.R1883Q	2	2						COSM1620970,COSM1620969			Missense_Mutation
MYO10	p.V1451I	2	2						COSM1733548	rs142263783		Missense_Mutation
PDHA1	p.M282L	1	2						COSM1740793,C	rs2229137		Missense_Mutation
TPSAB1	p.T141I	1	2						COSM1749411			Missense_Mutation
EEA1	p.E1010G	1	2						COSM2045342	rs76077763		Missense_Mutation
RNASET2	p.A127V	1	2						COSM207725	rs117003826		Missense_Mutation
DLG2	p.F18L	1	2						COSM2221185			Missense_Mutation
MUC2	p.T1568M	4	2						COSM224974,CO	rs201415503		Missense_Mutation
TPCN1	p.V203I	1	2						COSM242015			Missense_Mutation
PLA2G15	p.A81V	1	2						COSM246617	rs200107219		Missense_Mutation
CUL7	p.I1139T	1	2						COSM249271	rs77965460		Missense_Mutation
CUBN	p.E304K	3	2						COSM254639	rs78201384		Missense_Mutation
PXDNL	p.T598M	1	2						COSM287278	rs200870550		Missense_Mutation
MUC17	p.P2998L	2	2						COSM3082572	rs142796970		Missense_Mutation
MUC17	p.P3695L	1	2						COSM3082651	rs201783350		Missense_Mutation
MED12	p.L36P	1	2						COSM3095235			Missense_Mutation
ALDOA	p.D68N	1	2						COSM3111664	rs201468609		Missense_Mutation
DPF1	p.N86S	1	2						COSM3201912,C	rs200845328		Missense_Mutation
MYO3A	p.K1488E	3	2						COSM3397071	rs34204285		Missense_Mutation
GSPT1	p.G101C	3	2						COSM3420751	rs185937624		Missense_Mutation
OBSCN	p.P5980L	1	2						COSM3484745,C	rs182318410		Missense_Mutation
CATSPER1	p.G316D	2	2						COSM3676274	rs148686517		Missense_Mutation
SPRR3	p.T147R	1	2						COSM3677395	rs139275808		Missense_Mutation
MYH6	p.G378S	1	2						COSM3677933	rs148962966		Missense_Mutation
SLC6A4	p.K605N	1	2						COSM3680126	rs6352		Missense_Mutation
C2orf53	p.R222C	3	2						COSM3682625	rs3739097		Missense_Mutation
C4orf21	p.I1410T	3	2						COSM3683195	rs3828539		Missense_Mutation
GLI3	p.H1200D	2	2						COSM3685169	rs145069572		Missense_Mutation
LIMK1	p.R452Q	1	2						COSM3685215,C	rs55679316		Missense_Mutation
PDLIM2	p.A352V	2	2						COSM3685374	rs145349678		Missense_Mutation
BNC2	p.T782A	3	2						COSM3745936,C	rs3739714		Missense_Mutation
DPP6	p.E79K	2	2						COSM3747708,COSM3747707			Missense_Mutation
NT5C1B-RDH14	p.R187Q	2	2						COSM3757839,C	rs61742608		Missense_Mutation
REST	p.E692D	1	2						COSM3760680	rs2227902		Missense_Mutation
ZNF469	p.D2777V	4	2						COSM3765944	rs3812954		Missense_Mutation
ZDHHC11	p.V228L	1	2						COSM3768884,C	rs148433225		Missense_Mutation
HLA-DRB1	p.A100G	3	2						COSM3774243			Missense_Mutation
HLA-DRB1	p.Y59C	1	2						COSM3774247	rs3175105		Missense_Mutation
FRG1	p.N160S	5	2						COSM3780264			Missense_Mutation
TP53	p.V217G	1	2						COSM3787449,COSM3787448,COSM3787445,CO			Missense_Mutation
FRG1	p.R135K	3	2						COSM3825658	rs199978807		Missense_Mutation
HLA-DRB1	p.A169T	4	2						COSM3830219			Missense_Mutation
SSC5D	p.T1183M	1	2						COSM3835831	rs76172483		Missense_Mutation
MYPOP	p.P399L	2	2						COSM3892899	rs147675790		Missense_Mutation
MLLT3	p.S42G	1	2						COSM3906523			Missense_Mutation

FRG1B	p.M79V	1	2						COSM3911033,COSM3911035,COSM3911034			Missense_Mutation
MUC6	p.T1889I	5	2						COSM3927655,COSM3927656			Missense_Mutation
PPAN-P2RY11	p.T280A	2	2						COSM3932858,C[illegible]	rs143785583		Missense_Mutation
HLA-DRB1	p.K41T	1	2						COSM3949049	rs1136756		Missense_Mutation
HLA-DRB1	p.Q39H	1	2						COSM3949050	rs9269956		Missense_Mutation
DPYSL2	p.A118T	3	2						COSM3982485	rs2228979		Missense_Mutation
PSG4	p.A225V	4	2						COSM4000769,C[illegible]	rs139058318		Missense_Mutation
BPI	p.R90C	2	2						COSM4001899	rs5743500		Missense_Mutation
CCDC158	p.S27N	2	2						COSM4003146	rs146378981		Missense_Mutation
TMEM168	p.V23I	2	2						COSM4004258	rs150584875		Missense_Mutation
FAM104B	p.G61S	3	2						COSM4156937,COSM4156936			Missense_Mutation
HLA-DRB1	p.Q39L	2	2						COSM4160975	rs17882663		Missense_Mutation
ZNF462	p.N307T	2	2						COSM4163190,C[illegible]	rs80154710		Missense_Mutation
SEC16B	p.V643M	1	2						COSM424677	rs139593986		Missense_Mutation
CCDC74B	p.P201S	1	2						COSM441119	rs142452531		Missense_Mutation
TMEM247	p.Q128E	1	2						COSM442956	rs74318890		Missense_Mutation
FRG1B	p.R67K	4	2						COSM477962,CO[illegible]	rs147809085		Missense_Mutation
FRG1B	p.I89V	2	2						COSM477972,COSM477971,COSM3783478			Missense_Mutation
SMG9	p.P126L	1	2						COSM84532	rs199758349		Missense_Mutation
RAB40B	p.Q85P	1	2						COSM88052	rs111549422		Missense_Mutation
TSSK3	p.R63G	2	2						COSM98839	rs184256666		Missense_Mutation
ADCK5	p.N324S	1	2						COSM99007	rs199991427		Missense_Mutation
PRMT3	p.Q191*	1	2						COSM1158367			Nonsense_Mutation
APC	p.E1547*	2	2						COSM13879			Nonsense_Mutation
FRG1B	p.K100*	1	2						COSM1533107,COSM1533106,COSM3972497			Nonsense_Mutation
SOX14	p.E123*	1	2						COSM1536764			Nonsense_Mutation
PHC1	p.Q389*	3	2						COSM1606741	rs201644380		Nonsense_Mutation
PPP3CC	p.R51*	1	2						COSM163693			Nonsense_Mutation
ARHGAP5	p.E481*	3	2						COSM1666408	rs200628183		Nonsense_Mutation
ARHGAP5	p.R480*	2	2						COSM2153200	rs201986816		Nonsense_Mutation
LILRB5	p.R542P	3	2						COSM4000915,C[illegible]	rs76522818		Splice_Site
PLCH2	p.R989P	1	2									Missense_Mutation
PEX14	p.D332N	1	2									Missense_Mutation
NIPAL3	p.T252A	2	2									Missense_Mutation
ZSWIM5	p.S921G	1	2									Missense_Mutation
FOXD2	p.S207A	2	2									Missense_Mutation
C1orf173	p.S729A	1	2									Missense_Mutation
TCHHL1	p.S220R	1	2									Missense_Mutation
HRNR	p.G1940R	2	2									Missense_Mutation
TMEM79	p.L369P	1	2									Missense_Mutation
NVL	p.L560V	2	2									Missense_Mutation
AKR1C1	p.E77K	2	2									Missense_Mutation
CUBN	p.D705G	1	2									Missense_Mutation
TTC18	p.Q393E	2	2									Missense_Mutation
DPCD	p.F140I	2	2									Missense_Mutation
SH3PXD2A	p.T847P	1	2									Missense_Mutation
MUC6	p.A1640T	1	2									Missense_Mutation
MUC2	p.T1683M	2	2									Missense_Mutation
MUC5B	p.R4981H	2	2									Missense_Mutation
TRIM68	p.E111D	1	2									Missense_Mutation

DNHD1	p.E3837Q	1	2		■							Missense_Mutation
SCUBE2	p.N405S	1	2			■						Missense_Mutation
SPTY2D1	p.P458R	2	2		■							Missense_Mutation
DDB1	p.R626H	1	2					■				Missense_Mutation
CYB561A3	p.T64P	2	2				■					Missense_Mutation
C11orf83	p.Q36P	1	2			■						Missense_Mutation
KCNK4	p.H152N	1	2	■								Missense_Mutation
SF1	p.S468P	1	2					■				Missense_Mutation
GAL	p.R107C	1	2				■					Missense_Mutation
ARRB1	p.N416T	1	2		■							Missense_Mutation
B3GNT6	p.G331A	1	2					■				Missense_Mutation
B3GNT6	p.G331A	1	2					■				Missense_Mutation
FAM181B	p.P140S	1	2			■						Missense_Mutation
PCF11	p.Q467L	1	2					■				Missense_Mutation
C11orf57	p.H151Q	1	2			■						Missense_Mutation
C11orf57	p.H151Q	1	2	■								Missense_Mutation
PRMT8	p.Q32P	5	2					■				Missense_Mutation
SLC2A13	p.V557I	2	2					■				Missense_Mutation
WNT1	p.V135I	2	2		■							Missense_Mutation
ESPL1	p.E141A	4	2				■					Missense_Mutation
MAP3K12	p.S415C	2	2					■				Missense_Mutation
OTOGL	p.R1894P	1	2		■							Missense_Mutation
OTOGL	p.R1894P	1	2					■				Missense_Mutation
ATP2A2	p.D886Y	1	2		■							Missense_Mutation
FBXO21	p.R313W	2	2				■					Missense_Mutation
FREM2	p.M1195V	1	2					■				Missense_Mutation
NEK5	p.H278R	1	2			■						Missense_Mutation
DACH1	p.I319M	1	2	■								Missense_Mutation
MYCBP2	p.W4210R	1	2	■								Missense_Mutation
MYO16	p.S527Y	1	2	■								Missense_Mutation
OR11H4	p.N12H	4	2		■							Missense_Mutation
HECTD1	p.R2137P	1	2			■						Missense_Mutation
ARHGAP5	p.Y587S	3	2				■					Missense_Mutation
NPAS3	p.E36Q	2	2				■					Missense_Mutation
LRR1	p.T373P	1	2		■							Missense_Mutation
CYP46A1	p.I33T	1	2				■					Missense_Mutation
BRF1	p.K262T	1	2	■								Missense_Mutation
DIS3L	p.T13A	1	2					■				Missense_Mutation
PARP6	p.V26I	1	2			■						Missense_Mutation
MAN2A2	p.R735H	2	2					■				Missense_Mutation
ITFG1	p.L99F	2	2	■								Missense_Mutation
RBL2	p.P43L	2	2	■								Missense_Mutation
SLC12A4	p.E1005Q	1	2		■							Missense_Mutation
NFATC3	p.S750F	2	2		■							Missense_Mutation
PMFBP1	p.D567H	1	2			■						Missense_Mutation
CNTNAP4	p.N674H	3	2			■						Missense_Mutation
CENPN	p.Y324F	1	2	■								Missense_Mutation
RP11-830F9.6	p.R167W	1	2	■								Missense_Mutation
ANKRD11	p.E2154A	2	2					■				Missense_Mutation
TUBB3	p.D787N	1	2		■							Missense_Mutation

KSR1	p.T137P	1	2				■					Missense_Mutation
ACACA	p.H2285Y	1	2					■				Missense_Mutation
RP11-1407O15.2	p.Y438D	1	2	■								Missense_Mutation
LEPREL4	p.L169F	2	2			■						Missense_Mutation
GPATCH8	p.P741R	1	2			■						Missense_Mutation
C17orf104	p.D293H	2	2			■						Missense_Mutation
NPEPPS	p.I333F	1	2				■					Missense_Mutation
NPEPPS	p.T669A	2	2		■							Missense_Mutation
KCNJ16	p.E228D	1	2	■								Missense_Mutation
PPP4R1	p.S538R	1	2			■						Missense_Mutation
NPC1	p.D170E	1	2					■				Missense_Mutation
AC012123.1	p.S176P	2	2		■							Missense_Mutation
AZU1	p.V170I	1	2			■						Missense_Mutation
ZNF823	p.N114S	1	2					■				Missense_Mutation
PGLYRP2	p.D529N	1	2			■						Missense_Mutation
AP1M1	p.I295L	5	2			■						Missense_Mutation
TMEM91	p.A23S	1	2					■				Missense_Mutation
ERF	p.P4A	1	2	■								Missense_Mutation
CIC	p.S2501P	1	2	■								Missense_Mutation
BCL3	p.P42L	2	2		■							Missense_Mutation
BCL3	p.S382T	1	2					■				Missense_Mutation
BCAM	p.R191C	1	2					■				Missense_Mutation
GLTSCR2	p.Q340R	2	2	■								Missense_Mutation
CARD8	p.L437F	1	2					■				Missense_Mutation
HEATR5B	p.S1601A	1	2	■		■						Missense_Mutation
CYP1B1	p.Y137S	1	2	■								Missense_Mutation
SOS1	p.T1300R	2	2					■				Missense_Mutation
SMEK2	p.I201L	1	2					■				Missense_Mutation
MERTK	p.A885V	1	2			■						Missense_Mutation
TFCP2L1	p.R431Q	1	2					■				Missense_Mutation
RAB3GAP1	p.R336H	1	2			■						Missense_Mutation
MCM6	p.N665S	2	2		■							Missense_Mutation
NEB	p.R8248H	1	2					■				Missense_Mutation
GALNT3	p.T581S	1	2			■						Missense_Mutation
TTC30B	p.R605H	1	2	■								Missense_Mutation
MSTN	p.Y148C	1	2			■						Missense_Mutation
DNAH7	p.D3728G	1	2			■						Missense_Mutation
ABCA12	p.S1522T	1	2					■				Missense_Mutation
COL4A3	p.T477I	1	2					■				Missense_Mutation
HDLBP	p.L793F	2	2		■							Missense_Mutation
HDLBP	p.L793F	2	2		■							Missense_Mutation
DTYMK	p.G144E	2	2		■	■						Missense_Mutation
PET117	p.V20M	2	2				■					Missense_Mutation
AURKA	p.P70L	1	2			■						Missense_Mutation
SOX18	p.G285R	1	2		■							Missense_Mutation
KRTAP13-4	p.S46P	1	2	■								Missense_Mutation
SON	p.K288N	2	2				■					Missense_Mutation
DOPEY2	p.I1668V	1	2			■						Missense_Mutation
SLC25A1	p.V100A	2	2					■				Missense_Mutation
TOP3B	p.M306I	2	2					■				Missense_Mutation

MYO18B	p.L2271P	1	2									Missense_Mutation
MTFP1	p.R76K	1	2									Missense_Mutation
TRANK1	p.E1282G	1	2									Missense_Mutation
SNRK	p.Y664H	1	2									Missense_Mutation
SCAP	p.P861R	1	2									Missense_Mutation
IL17RD	p.R228H	2	2									Missense_Mutation
HHLA2	p.N303H	1	2									Missense_Mutation
SLC9C1	p.G392A	2	2									Missense_Mutation
NAA50	p.R21T	1	2									Missense_Mutation
CASR	p.G363S	2	2									Missense_Mutation
PLXNA1	p.R942Q	1	2									Missense_Mutation
MCM2	p.I771L	1	2									Missense_Mutation
BFSP2	p.S13I	2	2									Missense_Mutation
MED12L	p.R687S	2	2									Missense_Mutation
HES1	p.G141S	1	2									Missense_Mutation
PAK2	p.P151S	1	2									Missense_Mutation
ATP8A1	p.E16K	2	2									Missense_Mutation
CCNI	p.N342T	1	2									Missense_Mutation
NEUROG2	p.G195A	2	2									Missense_Mutation
LARP7	p.Q317H	1	2									Missense_Mutation
FREM3	p.R1527K	1	2									Missense_Mutation
ZNF827	p.A463V	1	2									Missense_Mutation
DCLK2	p.S402P	1	2									Missense_Mutation
LRBA	p.A2535G	1	2									Missense_Mutation
FGA	p.K625I	1	2									Missense_Mutation
FSTL5	p.T606I	1	2									Missense_Mutation
FAT1	p.F360L	1	2									Missense_Mutation
LPCAT1	p.G512S	1	2									Missense_Mutation
ADAMTS12	p.N1012D	2	2									Missense_Mutation
NIPBL	p.D826G	2	2									Missense_Mutation
C9	p.T170S	1	2									Missense_Mutation
PELO	p.E187A	1	2									Missense_Mutation
AP3S1	p.I182V	1	2									Missense_Mutation
HSPA9	p.I589V	1	2									Missense_Mutation
TAF7	p.S264L	1	2									Missense_Mutation
DOCK2	p.Q869R	1	2									Missense_Mutation
CPEB4	p.E538D	2	2									Missense_Mutation
BTN2A2	p.N124K	2	2									Missense_Mutation
BTN2A1	p.E91D	2	2									Missense_Mutation
HIST1H2AI	p.A53T	1	2									Missense_Mutation
BRD2	p.S820C	1	2									Missense_Mutation
ENPP5	p.E119K	2	2									Missense_Mutation
EYS	p.P723A	1	2									Missense_Mutation
TBX18	p.E29K	1	2									Missense_Mutation
FAM184A	p.E229K	1	2									Missense_Mutation
UTRN	p.D2987E	2	2									Missense_Mutation
TMEM196	p.E154D	1	2									Missense_Mutation
AOAH	p.L657F	1	2									Missense_Mutation
UBE2D4	p.C111S	1	2									Missense_Mutation
SEC61G	p.I19V	2	2									Missense_Mutation

GTPBP10	p.E307Q	1	2									Missense_Mutation
SAMD9	p.R106C	2	2									Missense_Mutation
DYNC1I1	p.S137L	2	2									Missense_Mutation
MUC17	p.E26K	2	2									Missense_Mutation
MUC17	p.T3298M	1	2									Missense_Mutation
MUC17	p.T3487I	1	2									Missense_Mutation
NCAPG2	p.W235C	1	2									Missense_Mutation
MSRA	p.S3W	1	2									Missense_Mutation
POLB	p.S275G	2	2									Missense_Mutation
CHMP4C	p.E121A	1	2									Missense_Mutation
KCNS2	p.R52H	1	2									Missense_Mutation
EPPK1	p.R89W	2	2									Missense_Mutation
CPSF1	p.I1060T	1	2									Missense_Mutation
SLC39A4	p.Q145H	2	2									Missense_Mutation
ZNF658	p.H340Q	3	2									Missense_Mutation
FGD3	p.H208Q	2	2									Missense_Mutation
ZNF782	p.E622K	2	2									Missense_Mutation
SVEP1	p.R3545G	1	2									Missense_Mutation
SUSD1	p.T389I	1	2									Missense_Mutation
SARDH	p.V470I	1	2									Missense_Mutation
ZMYND19	p.P82A	1	2									Missense_Mutation
TLR8	p.V931I	1	2									Missense_Mutation
CXorf22	p.V857L	1	2									Missense_Mutation
KIAA2022	p.G1093E	1	2									Missense_Mutation
UPRT	p.S10T	2	2									Missense_Mutation
MAGEE1	p.S732Y	2	2									Missense_Mutation
DIAPH2	p.D882N	1	2									Missense_Mutation
FRMPD3	p.S602C	1	2									Missense_Mutation
MAGEC1	p.Q362H	1	2									Missense_Mutation
ATP2B3	p.S1178P	1	2									Missense_Mutation
KMT2D	p.E5234*	1	2									Nonsense_Mutation
LRP1	p.Q4289*	2	2									Nonsense_Mutation
KATNBL1	p.S138*	2	2									Nonsense_Mutation
AXIN1	p.Q485*	1	2									Nonsense_Mutation
EXOC3L1	p.Q73*	2	2									Nonsense_Mutation
GHDC	p.W413*	1	2									Nonsense_Mutation
TRAPPC12	p.E113*	1	2									Nonsense_Mutation
FRG1B	p.E159*	5	2									Nonsense_Mutation
PLA2G6	p.E786*	1	2									Nonsense_Mutation
PLCH1	p.E1501*	2	2									Nonsense_Mutation
BDH1	p.R97*	2	2									Nonsense_Mutation
GPRIN3	p.S111*	1	2									Nonsense_Mutation
FRG1	p.K168*	1	2									Nonsense_Mutation
MDN1	p.Y1146*	2	2									Nonsense_Mutation
SYNE1	p.C4021*	2	2									Nonsense_Mutation
FAM188B	p.G83*	2	2									Nonsense_Mutation
NPSR1	p.W201*	2	2									Nonsense_Mutation
C7orf65	p.W15*	1	2									Nonsense_Mutation
PCLO	p.W4444*	1	2									Nonsense_Mutation
MTUS1	p.Q75*	1	2									Nonsense_Mutation

FAM189A2	p.K335*	1	2		■							Nonsense_Mutation
SVEP1	p.E738*	2	2			■						Nonsense_Mutation
LAMC3	p.C722*	1	2				■					Nonsense_Mutation
LAMC3	p.C843*	2	2		■							Nonsense_Mutation
PGAP2	p.F93F	1	2					■				Silent
FADS3	p.L273L	1	2	■								Silent
KCTD15	p.R172R	1	2			■						Silent
FAM172A	p.F67F	2	2				■					Silent
FBXL18	p.V622V	2	2		■							Silent
MYO5C	p.E1101G	2	2			■						Splice_Site
SIGLEC1	p.G1260R	1	2									Splice_Site
AAGAB	p.M1I	2	2				■					Start_Codon_SNP
C3orf20	p.A563T	1	1			■	■	■	COSM1039643	rs572485123		Missense_Mutation
DSPP	p.N1023D	1	1			■			COSM1058645			Missense_Mutation
FRG1B	p.S101N	3	1			■			COSM1130665,COSM1130666,COSM3748147			Missense_Mutation
METTL2B	p.C124R	5	1	■	■	■	■	■	COSM1131716	rs2896399		Missense_Mutation
MUC17	p.G2207D	3	1		■	■		■	COSM1131757	rs201412788		Missense_Mutation
TYSND1	p.P203L	1	1	■	■	■	■	■	COSM1162271	rs11549689		Missense_Mutation
WDR96	p.S226L	3	1	■	■	■	■	■	COSM1185155	rs118083279		Missense_Mutation
TBC1D10B	p.R728Q	2	1		■	■		■	COSM1228547,CO	rs138992028		Missense_Mutation
ABCA6	p.E1463Q	1	1	■	■	■	■		COSM1303261	rs143326198		Missense_Mutation
MUC2	p.P1608H	1	1			■		■	COSM1351123			Missense_Mutation
FAF1	p.P89S	2	1		■	■		■	COSM14017			Missense_Mutation
NMNAT1	p.S255N	3	1		■	■	■	■	COSM146387	rs138626416		Missense_Mutation
TRIM22	p.T232A	2	1	■	■	■	■	■	COSM147126	rs2291843		Missense_Mutation
MICAL2	p.D687E	2	1	■	■	■	■		COSM147147	rs3794084		Missense_Mutation
MUC5B	p.G2588R	1	1	■	■		■		COSM1475202,CO	rs200110372		Missense_Mutation
GLDN	p.A521T	1	1		■		■	■	COSM147906	rs75780940		Missense_Mutation
DNMT1	p.H97R	2	1	■			■	■	COSM148505	rs16999593		Missense_Mutation
NUP62	p.G139S	4	1	■	■	■	■	■	COSM148689	rs3745489		Missense_Mutation
POLH	p.T478M	1	1		■	■	■	■	COSM150093	rs9296419		Missense_Mutation
C7orf10	p.I167L	2	1	■	■	■	■	■	COSM150273	rs138102615		Missense_Mutation
DNAH11	p.V848M	2	1	■	■	■	■	■	COSM160473	rs80328282		Missense_Mutation
TTN	p.V28979I	1	1	■		■			COSM1636871,CO	rs201687390		Missense_Mutation
NPIPB15	p.E353K	1	1	■	■	■	■	■	COSM1716109,CO	rs200974911		Missense_Mutation
LYPLAL1	p.A17V	2	1		■		■	■	COSM1738510	rs200373846		Missense_Mutation
NME3	p.K51R	1	1	■					COSM1743923	rs202165010		Missense_Mutation
ATAD2	p.R295Q	1	1					■	COSM176940			Missense_Mutation
PLCE1	p.V2158I	2	1	■	■	■		■	COSM1970621,CO	rs200549010		Missense_Mutation
RNASET2	p.A127V	1	1	■	■	■	■	■	COSM207725	rs117003826		Missense_Mutation
STK11	p.F354L	1	1	■	■	■		■	COSM21360	rs59912467		Missense_Mutation
NECAP1	p.T3I	2	1		■			■	COSM228147			Missense_Mutation
MUC17	p.T1808N	2	1		■	■	■		COSM228468	rs147991653		Missense_Mutation
ANKLE2	p.V202I	1	1	■					COSM236349	rs185739493		Missense_Mutation
DOCK5	p.M987L	2	1		■				COSM249512	rs74894413		Missense_Mutation
GUCY2D	p.E754K	1	1						COSM2804621			Missense_Mutation
AMER1	p.A29T	1	1	■	■	■	■	■	COSM28715	rs138399473		Missense_Mutation
HEPN1	p.R69W	1	1			■			COSM297812	rs183886198		Missense_Mutation
NYAP2	p.P499L	2	1	■	■				COSM3046839			Missense_Mutation
KALRN	p.T1207M	1	1			■		■	COSM3057630,CO	rs139468141		Missense_Mutation

GPR98	p.R4802Q	1	1						COSM3140875	rs534266547		Missense_Mutation
OBSCN	p.V2305M	1	1						COSM33596	rs545316651		Missense_Mutation
MUC19	p.V285I	1	1						COSM3384320	rs28365246		Missense_Mutation
KCNQ3	p.R757Q	1	1						COSM3645169			Missense_Mutation
SH3PXD2A	p.A391V	2	1						COSM3675564	rs117568095		Missense_Mutation
NCAM1	p.A471V	1	1						COSM3675983,CO	rs117485712		Missense_Mutation
OR5T3	p.W84G	2	1						COSM3676189	rs17150243		Missense_Mutation
DUOX1	p.I962T	1	1						COSM3678219,CO	rs16939743		Missense_Mutation
COQ7	p.R23W	1	1						COSM3678654	rs77337400		Missense_Mutation
ZNF808	p.G99E	1	1						COSM3681024	rs117753855		Missense_Mutation
DEFB129	p.A122V	1	1						COSM3681162	rs79468308		Missense_Mutation
MICAL3	p.T1276I	2	1						COSM3681366	rs78616323		Missense_Mutation
MICAL3	p.T1276I	2	1						COSM3681366	rs78616323		Missense_Mutation
KIAA1210	p.R8L	1	1						COSM3681521	rs142034189		Missense_Mutation
GCKR	p.E77G	1	1						COSM3682636	rs8179206		Missense_Mutation
NCAPG2	p.E867D	1	1						COSM3685126,CO	rs3214000		Missense_Mutation
SOX7	p.T169S	3	1						COSM3685286	rs4841433		Missense_Mutation
ZNF292	p.R2080Q	2	1						COSM368943,COS	rs76690660		Missense_Mutation
NFE2L3	p.T355I	1	1						COSM3698348	rs151210268		Missense_Mutation
CYP2F1	p.R489H	2	1						COSM3746496	rs80029441		Missense_Mutation
ZNF181	p.V349G	2	1						COSM3748052,CO	rs143797666		Missense_Mutation
PHLDA1	p.P16S	1	1						COSM3753495	rs3747549		Missense_Mutation
GNAS	p.A436D	1	1						COSM3758660	rs61749698		Missense_Mutation
MUC6	p.P1569L	1	1						COSM3773559,COSM3773560			Missense_Mutation
KRT3	p.A134P	2	1						COSM3773647			Missense_Mutation
DHRS4L2	p.L126I	2	1						COSM3773700,CO	rs61999853		Missense_Mutation
HRNR	p.Q2469L	1	1						COSM3801865			Missense_Mutation
LCE1D	p.R78H	2	1						COSM3801917	rs41268490		Missense_Mutation
CATSPERG	p.E753K	2	1						COSM3822945,CO	rs184689060		Missense_Mutation
HLA-DRB1	p.G154A	3	1						COSM3830220			Missense_Mutation
SSC5D	p.T1182S	1	1						COSM3835830	rs77400028		Missense_Mutation
SSC5D	p.H1300Y	1	1						COSM3835836	rs201038280		Missense_Mutation
ADAMTSL1	p.V1265I	1	1						COSM3906451	rs77886371		Missense_Mutation
MUC2	p.T1560M	4	1						COSM3927681,COSM3927682			Missense_Mutation
MUC5B	p.V4323F	4	1						COSM3931262,CO	rs201326462		Missense_Mutation
AL445989.1	p.G23S	1	1						COSM3955690,COSM3955689			Missense_Mutation
DHRS4	p.I126L	1	1						COSM3955938	rs17422812		Missense_Mutation
CCAR2	p.Q603H	2	1						COSM3982473	rs59511580		Missense_Mutation
ACER2	p.A134V	2	1						COSM3982800,CO	rs10964136		Missense_Mutation
OTOL1	p.M238R	2	1						COSM3992847			Missense_Mutation
RASSF5	p.V189M	1	1						COSM3997183,CO	rs148860285		Missense_Mutation
USH2A	p.Q4203R	3	1						COSM3997234,CO	rs148556640		Missense_Mutation
MSRB2	p.E46G	1	1						COSM3997897	rs2296466		Missense_Mutation
MUC5B	p.M3122T	5	1						COSM3998324,CO	rs58125533		Missense_Mutation
RNASE13	p.R7W	1	1						COSM3999215	rs140441722		Missense_Mutation
KRTAP4-4	p.T139I	1	1						COSM4000156	rs74572864		Missense_Mutation
MUC16	p.P8789R	3	1						COSM4001062,CO	rs78804712		Missense_Mutation
WDSUB1	p.F328S	1	1						COSM4001257	rs78460202		Missense_Mutation
DEFB126	p.K76R	2	1						COSM4001823	rs74717966		Missense_Mutation
ALPK1	p.G870S	1	1						COSM4002907	rs2074380		Missense_Mutation

SLC26A3	p.R554Q	2	1						COSM4004244	rs2301635		Missense_Mutation
MUC2	p.T1756N	1	1						COSM400441,COSM400440			Missense_Mutation
DNAI2	p.A525V	3	1						COSM4069309	rs145602856		Missense_Mutation
SAMHD1	p.T608A	1	1						COSM4098092			Missense_Mutation
CHTF18	p.S63F	1	1						COSM4129422,CO	rs2277902		Missense_Mutation
SSC5D	p.H1294Y	1	1						COSM4132632	rs113619253		Missense_Mutation
FCGR3A	p.D82N	1	1						COSM4142920	rs200727785		Missense_Mutation
CEP170	p.T684K	3	1						COSM4143584,CO	rs2728430		Missense_Mutation
CEP170	p.T684K	3	1						COSM4143584,CO	rs2728430		Missense_Mutation
ROBO3	p.P868L	1	1						COSM4145447,CO	rs55706177		Missense_Mutation
SVIL	p.S228L	2	1						COSM427457			Missense_Mutation
EVPL	p.D444E	1	1						COSM437420	rs7218767		Missense_Mutation
PDE1C	p.G698S	1	1						COSM452978,COS	rs74905175		Missense_Mutation
AEBP1	p.R368Q	2	1						COSM461594	rs75175945		Missense_Mutation
ZIM2	p.S320L	1	1						COSM714368	rs138884282		Missense_Mutation
GPR20	p.A66S	1	1						COSM749866			Missense_Mutation
ZP4	p.A222V	2	1						COSM906291			Missense_Mutation
MUC2	p.T1589N	1	1						COSM922852,COS	rs111219026		Missense_Mutation
MUC2	p.Q1602H	1	1						COSM922861			Missense_Mutation
PZP	p.Q598*	1	1						COSM249340,COS	rs117889746		Nonsense_Mutation
ASB15	p.R282*	1	1						COSM249444	rs116956332		Nonsense_Mutation
TP53	p.Q331*	1	1						COSM3522688,COSM289612,COSM1649348,COS			Nonsense_Mutation
HLA-DRB1	p.Q39*	1	1						COSM3949051	rs9269957		Nonsense_Mutation
IFNA4	p.C20*	2	1						COSM4163699	rs113055208		Nonsense_Mutation
TLE4	p.Q458Q	2	1						COSM1555323,COSM1555324			Silent
IPO5	p.Y902Y	3	1						COSM199020	rs140767528		Silent
CYP4A22	p.D293D	4	1						COSM3360938			Silent
TRPM3	p.A879A	1	1						COSM3433211,CO	rs35390807		Silent
AATK	p.T196T	1	1						COSM357849			Silent
GCA	p.E121E	2	1						COSM3682443	rs147889616		Silent
COBLL1	p.S39S	2	1						COSM3682448	rs3765059		Silent
PLOD3	p.R313R	3	1						COSM3684938	rs1546840		Silent
GSE1	p.R349R	1	1						COSM3691224	rs111733333		Silent
MSI1	p.A276A	2	1						COSM371564	rs150872271		Silent
SLC23A2	p.P408P	1	1						COSM3770842			Silent
KIAA0226	p.A917A	1	1						COSM4157940,CO	rs200116207		Silent
LRRC7	p.D470D	1	1						COSM911939	rs145241026		Silent
ST8SIA6	p.S207S	2	1						COSM916844			Silent
SYT1	p.Y283Y	1	1						COSM943689	rs146053442		Silent
SIK3	p.Q702Q	1	1						COSM99013	rs560511616		Silent
GPA33	p.0	1	1						COSM1499403	rs150875349		Splice_Site
VIT	p.D291N	2	1						COSM1531993			Splice_Site
U2AF2	p.0	3	1						COSM248171			Splice_Site
PCM1	p.A262T	1	1						COSM2873451	rs140481840		Splice_Site
ZNF717	p.A43T	1	1						COSM3727848,CO	rs199946555		Splice_Site
DZANK1	p.K537K	1	1						COSM3840519,CO	rs141536016		Splice_Site
FRG1B	p.0	5	1						COSM3911032,COSM3911031,COSM3911030			Splice_Site
COL7A1	p.0	1	1						COSM730653			Splice_Site
CCL4L2	p.0	1	1						COSM978092	rs184899362		Splice_Site
VWA1	p.D280G	1	1									Missense_Mutation

MIB2	p.D835V	1	1									Missense_Mutation
PLCH2	p.R1359P	1	1									Missense_Mutation
NPHP4	p.P527L	1	1									Missense_Mutation
FBXO42	p.V537A	1	1									Missense_Mutation
HSPG2	p.T2511S	1	1									Missense_Mutation
SNRNP40	p.H22R	1	1									Missense_Mutation
HIVEP3	p.P1884S	2	1									Missense_Mutation
CYP4A11	p.L11R	2	1									Missense_Mutation
KANK4	p.F213L	1	1									Missense_Mutation
MCOLN3	p.H14Y	2	1									Missense_Mutation
LPPR4	p.S30T	1	1									Missense_Mutation
TCHH	p.L413V	1	1									Missense_Mutation
LMX1A	p.F11L	1	1									Missense_Mutation
NPHS2	p.A27G	1	1									Missense_Mutation
IGFN1	p.G2123S	1	1									Missense_Mutation
RYR2	p.D1196G	1	1									Missense_Mutation
WDFY4	p.E2044G	1	1									Missense_Mutation
PLAU	p.P160S	1	1									Missense_Mutation
DLG5	p.A1130S	1	1									Missense_Mutation
ITPRIP	p.V187M	2	1									Missense_Mutation
C10orf137	p.N292S	2	1									Missense_Mutation
C11orf35	p.V514L	1	1									Missense_Mutation
MUC2	p.T1570I	3	1									Missense_Mutation
MUC2	p.P1721T	3	1									Missense_Mutation
OR52W1	p.P51T	1	1									Missense_Mutation
OR52W1	p.P51Q	1	1									Missense_Mutation
NLRP14	p.E471D	1	1									Missense_Mutation
PGA5	p.V374I	1	1									Missense_Mutation
SF1	p.S468P	1	1									Missense_Mutation
FOLR3	p.H40Y	1	1									Missense_Mutation
PDE2A	p.A594V	1	1									Missense_Mutation
CREBZF	p.V75L	1	1									Missense_Mutation
CEP164	p.T1311P	1	1									Missense_Mutation
C2CD2L	p.L297V	1	1									Missense_Mutation
PRMT8	p.Q32H	2	1									Missense_Mutation
PRB2	p.P73R	1	1									Missense_Mutation
SLC38A1	p.I399T	1	1									Missense_Mutation
NAV3	p.K1759Q	4	1									Missense_Mutation
FICD	p.H161Y	1	1									Missense_Mutation
MMAB	p.T168A	1	1									Missense_Mutation
TRAFD1	p.M179I	2	1									Missense_Mutation
MEDAG	p.V159L	1	1									Missense_Mutation
CSNK1A1L	p.N151S	1	1									Missense_Mutation
CCDC168	p.G1124E	2	1									Missense_Mutation
LRR1	p.T373P	1	1									Missense_Mutation
AK7	p.D245N	1	1									Missense_Mutation
TNFAIP2	p.R342H	2	1									Missense_Mutation
DIS3L	p.N251S	1	1									Missense_Mutation
TICRR	p.S1074F	2	1									Missense_Mutation
SV2B	p.D8E	2	1									Missense_Mutation

SRRM2	p.P1278R	1	1									Missense_Mutation
RP11-166B2.1	p.R94C	1	1									Missense_Mutation
ARMC5	p.N36T	2	1									Missense_Mutation
ADCY7	p.R525C	1	1									Missense_Mutation
ADAD2	p.S148G	1	1									Missense_Mutation
ZNF469	p.T3815M	1	1									Missense_Mutation
OR1E1	p.F113L	1	1									Missense_Mutation
PLD2	p.P487T	1	1									Missense_Mutation
C17orf59	p.D136V	1	1									Missense_Mutation
SLFN14	p.L427V	1	1									Missense_Mutation
RP11-1407O15.2	p.Y438D	1	1									Missense_Mutation
STAC2	p.S78P	1	1									Missense_Mutation
KRT10	p.H491N	1	1									Missense_Mutation
EZH1	p.A357S	1	1									Missense_Mutation
TBC1D16	p.D506N	1	1									Missense_Mutation
RNF213	p.G2489D	1	1									Missense_Mutation
LAMA1	p.H2572N	2	1									Missense_Mutation
LOXHD1	p.A489S	1	1									Missense_Mutation
MUC16	p.T4266P	1	1									Missense_Mutation
ZNF441	p.E466A	1	1									Missense_Mutation
ZNF69	p.F285L	1	1									Missense_Mutation
ZNF14	p.A332T	2	1									Missense_Mutation
C19orf69	p.I89T	1	1									Missense_Mutation
ZNF234	p.S156C	2	1									Missense_Mutation
PVR	p.C378G	2	1									Missense_Mutation
GIPR	p.S397F	1	1									Missense_Mutation
CLEC11A	p.V127L	1	1									Missense_Mutation
APOB	p.R2309K	2	1									Missense_Mutation
RHOQ	p.A33S	1	1									Missense_Mutation
FSHR	p.C188G	1	1									Missense_Mutation
KIAA1841	p.I345V	1	1									Missense_Mutation
USP34	p.L3069V	1	1									Missense_Mutation
NEB	p.T492I	1	1									Missense_Mutation
FSIP2	p.T1337I	1	1									Missense_Mutation
FSIP2	p.T2188K	1	1									Missense_Mutation
ORC2	p.N505D	1	1									Missense_Mutation
PECR	p.S271L	1	1									Missense_Mutation
AC064874.1	p.M54K	2	1									Missense_Mutation
GBX2	p.S102C	2	1									Missense_Mutation
SNED1	p.Q1242P	1	1									Missense_Mutation
OXT	p.L101S	1	1									Missense_Mutation
RRBP1	p.A777T	1	1									Missense_Mutation
FRG1B	p.V58L	3	1									Missense_Mutation
RBM12	p.P568Q	1	1									Missense_Mutation
KCNS1	p.Q480R	1	1									Missense_Mutation
ZMYND8	p.E642D	2	1									Missense_Mutation
TSHZ2	p.K774R	2	1									Missense_Mutation
ANKRD60	p.V77F	1	1									Missense_Mutation
KRTAP10-6	p.K162E	1	1									Missense_Mutation
GNAZ	p.I286V	2	1									Missense_Mutation

NPTXR	p.P228R	2	1									Missense_Mutation
CBX6	p.L179P	1	1									Missense_Mutation
POMGNT2	p.A156T	1	1									Missense_Mutation
KIF15	p.T243I	1	1									Missense_Mutation
LZTFL1	p.A178V	2	1									Missense_Mutation
RBM15B	p.S9C	1	1									Missense_Mutation
COL6A6	p.A213T	2	1									Missense_Mutation
ACKR4	p.I185M	1	1									Missense_Mutation
EIF2A	p.A235S	1	1									Missense_Mutation
EIF2A	p.A235S	1	1									Missense_Mutation
MED12L	p.R687S	2	1									Missense_Mutation
OTOL1	p.D246V	2	1									Missense_Mutation
TMEM212	p.T57S	2	1									Missense_Mutation
YEATS2	p.P1118L	1	1									Missense_Mutation
PPP1R2	p.S205A	2	1									Missense_Mutation
TNK2	p.H182R	1	1									Missense_Mutation
NKX1-1	p.P210A	1	1									Missense_Mutation
EVC	p.H607Q	1	1									Missense_Mutation
BEND4	p.Y18S	1	1									Missense_Mutation
YIPF7	p.T127S	1	1									Missense_Mutation
LARP7	p.Q317H	1	1									Missense_Mutation
FGA	p.K625I	1	1									Missense_Mutation
KIAA0947	p.E1674K	1	1									Missense_Mutation
RAI14	p.M579T	1	1									Missense_Mutation
NIPBL	p.E2496Q	2	1									Missense_Mutation
FER	p.T164N	1	1									Missense_Mutation
LYRM7	p.V40L	1	1									Missense_Mutation
PCDHA5	p.S439N	1	1									Missense_Mutation
CTB-78H18.1	p.H134P	2	1									Missense_Mutation
CTB-78H18.1	p.T136P	3	1									Missense_Mutation
MYLIP	p.S354L	1	1									Missense_Mutation
DPCR1	p.S376F	1	1									Missense_Mutation
RNF8	p.M143I	1	1									Missense_Mutation
DNAH8	p.Q3953K	1	1									Missense_Mutation
EYS	p.M1824L	2	1									Missense_Mutation
SYNE1	p.W2639R	2	1									Missense_Mutation
ARID1B	p.T1078S	2	1									Missense_Mutation
TNRC18	p.S2561I	1	1									Missense_Mutation
ABCB5	p.T817I	1	1									Missense_Mutation
AOAH	p.M659I	1	1									Missense_Mutation
AOAH	p.M659I	1	1									Missense_Mutation
AOAH	p.M659K	2	1									Missense_Mutation
AOAH	p.M659K	2	1									Missense_Mutation
AOAH	p.Q638K	1	1									Missense_Mutation
PCLO	p.E2089K	2	1									Missense_Mutation
FZD1	p.E3Q	1	1									Missense_Mutation
MUC17	p.S1410N	1	1									Missense_Mutation
COG5	p.A760G	2	1									Missense_Mutation
AASS	p.T687M	2	1									Missense_Mutation
CADPS2	p.P36R	1	1									Missense_Mutation

ASB15	p.V127I	1	1									Missense_Mutation
RBM28	p.P603T	2	1									Missense_Mutation
CALD1	p.T442N	1	1									Missense_Mutation
TAS2R3	p.I11T	2	1									Missense_Mutation
AC008060.7	p.R63G	1	1									Missense_Mutation
DEFB105A	p.Q33H	2	1									Missense_Mutation
ZNF572	p.R18M	1	1									Missense_Mutation
DENND3	p.D517E	2	1									Missense_Mutation
TONSL	p.E453V	1	1									Missense_Mutation
SHB	p.S18T	1	1									Missense_Mutation
NOL8	p.S508N	2	1									Missense_Mutation
COL27A1	p.M1700I	2	1									Missense_Mutation
USP20	p.R370W	1	1									Missense_Mutation
ADAMTSL2	p.D108G	2	1									Missense_Mutation
MXRA5	p.R1598K	1	1									Missense_Mutation
AR	p.D354E	1	1									Missense_Mutation
STARD8	p.R32K	2	1									Missense_Mutation
TEX11	p.D847E	1	1									Missense_Mutation
ACRC	p.D231G	1	1									Missense_Mutation
ARMCX4	p.L2043P	1	1									Missense_Mutation
ZCCHC12	p.R339K	1	1									Missense_Mutation
LONRF3	p.E543A	1	1									Missense_Mutation
KIAA1210	p.D184G	1	1									Missense_Mutation
TENM1	p.M516V	1	1									Missense_Mutation
MAGEC1	p.M412R	1	1									Missense_Mutation
MAGEA6	p.D156N	2	1									Missense_Mutation
MAGEA6	p.D156G	2	1									Missense_Mutation
IGSF3	p.R381*	1	1									Nonsense_Mutation
RIN1	p.E315*	1	1									Nonsense_Mutation
CCDC67	p.E308*	2	1									Nonsense_Mutation
CCDC64	p.Q427*	1	1									Nonsense_Mutation
NCOR2	p.E881*	2	1									Nonsense_Mutation
CEP152	p.W105*	1	1									Nonsense_Mutation
SPAG5	p.R431*	1	1									Nonsense_Mutation
TIGD1	p.K128*	1	1									Nonsense_Mutation
COL9A3	p.R682*	2	1									Nonsense_Mutation
PRIMPOL	p.G355*	2	1									Nonsense_Mutation
NIPBL	p.S1517*	1	1									Nonsense_Mutation
ANKRD31	p.L276*	2	1									Nonsense_Mutation
DNAH11	p.E794*	2	1									Nonsense_Mutation
PON1	p.Q192*	2	1									Nonsense_Mutation
GSTK1	p.Q193*	1	1									Nonsense_Mutation
ZNF398	p.K248*	2	1									Nonsense_Mutation
PTCH1	p.Q700*	2	1									Nonsense_Mutation
DGAT2L6	p.E238*	2	1									Nonsense_Mutation
THEMIS2	p.C110C	2	1									Silent
FAM19A3	p.P105P	2	1									Silent
TRDMT1	p.V182V	1	1									Silent
PGAP2	p.F93F	1	1									Silent
FADS3	p.L273L	1	1									Silent

B3GNT6	p.P330P	5	1									Silent
B3GNT6	p.P330P	5	1									Silent
GDF11	p.A39A	2	1									Silent
GRIP1	p.S740S	1	1									Silent
GLIPR1L2	p.L198L	1	1									Silent
PPFIA2	p.I226I	2	1									Silent
ATP2A2	p.V885V	1	1									Silent
CIT	p.E490E	1	1									Silent
CHD8	p.F1638F	2	1									Silent
ADCY4	p.S502S	1	1									Silent
HEATR5A	p.A2000A	2	1									Silent
LTBP2	p.H1189H	2	1									Silent
GSE1	p.R351R	1	1									Silent
CPAMD8	p.S1036S	1	1									Silent
ZNF155	p.I292I	2	1									Silent
UBR3	p.F1831F	1	1									Silent
DCAF17	p.I297I	2	1									Silent
NEU4	p.L225L	1	1									Silent
MROH8	p.T290T	1	1									Silent
ARHGAP40	p.F210F	1	1									Silent
ITSN1	p.A1336A	1	1									Silent
FTCD	p.L468L	1	1									Silent
MGLL	p.V165V	1	1									Silent
NELFA	p.T173T	2	1									Silent
TBC1D1	p.V997V	1	1									Silent
KIF13A	p.Y1428Y	2	1									Silent
MDC1	p.R1152R	1	1									Silent
MED20	p.V142V	1	1									Silent
FBXL18	p.V622V	2	1									Silent
DMTF1	p.H553H	2	1									Silent
PUS7	p.I197I	1	1									Silent
DENND3	p.F56F	1	1									Silent
DMD	p.K2580K	1	1									Silent
PHF8	p.K492K	2	1									Silent
PHF8	p.K492K	2	1									Silent
MED12	p.Y1812Y	1	1									Silent
MKNK1	p.0	2	1									Splice_Site
MKNK1	p.0	2	1									Splice_Site
FNDC7	p.0	1	1									Splice_Site
SPAG6	p.0	2	1									Splice_Site
MS4A15	p.0	1	1									Splice_Site
PCBP2	p.0	2	1									Splice_Site
RFC3	p.0	1	1									Splice_Site
DICER1	p.0	2	1									Splice_Site
OCA2	p.0	1	1									Splice_Site
SPTBN5	p.0	1	1									Splice_Site
APH1B	p.0	2	1									Splice_Site
KIAA1199	p.0	2	1									Splice_Site
CIAPIN1	p.M210I	1	1									Splice_Site
ARRB2	p.0	1	1									Splice_Site

TEX14	p.0	2	1									Splice_Site
LRRC37A3	p.0	1	1									Splice_Site
ZNF555	p.0	2	1									Splice_Site
DOPEY2	p.0	1	1									Splice_Site
NMU	p.0	1	1									Splice_Site
DNAH5	p.0	1	1									Splice_Site
BRD2	p.0	2	1									Splice_Site
AVL9	p.0	2	1									Splice_Site
LAMB4	p.0	2	1									Splice_Site
MGAM	p.0	1	1									Splice_Site
LAMP2	p.0	1	1									Splice_Site
CAPZB	p.M1V	2	1									Start_Codon_SNP
CYP27A1	p.V238M	1	0						COSM1016501	rs199691576		Missense_Mutation
FAM194A	p.V56E	1	0						COSM1039882	rs573303855		Missense_Mutation
MUC2	p.G1577S	3	0						COSM1127924,COSM1127923			Missense_Mutation
RFPL1	p.S224G	1	0						COSM1130464			Missense_Mutation
NPIPB15	p.A394T	2	0						COSM1135829,COSM472094			Missense_Mutation
TAS1R1	p.T139M	1	0						COSM1158344	rs141817074		Missense_Mutation
KIAA1257	p.M28V	1	0						COSM1168980	rs199583351		Missense_Mutation
BRD1	p.A689V	2	0						COSM1184880,CO	rs145415691		Missense_Mutation
ZNF678	p.S198N	1	0						COSM1185762,COSM1185761			Missense_Mutation
BIRC7	p.T26R	1	0						COSM1190314			Missense_Mutation
EXO1	p.E657K	1	0						COSM1197123	rs200010267		Missense_Mutation
EXO1	p.E657K	1	0						COSM1197123	rs200010267		Missense_Mutation
CHIT1	p.A170S	1	0						COSM1201054	rs151004649		Missense_Mutation
KRTAP9-1	p.T152A	1	0						COSM1212900,COSM1212899			Missense_Mutation
TNRC18	p.A811T	1	0						COSM1229996,COSM1229997			Missense_Mutation
CHST8	p.R363H	2	0						COSM1237738			Missense_Mutation
PRR23C	p.A176T	1	0						COSM1263178			Missense_Mutation
OBSCN	p.A6021V	1	0						COSM1339788,CO	rs56359770		Missense_Mutation
GPR158	p.N1103S	2	0						COSM1347339			Missense_Mutation
GGT1	p.S428N	1	0						COSM137241	rs201313233		Missense_Mutation
FANCI	p.R1019W	1	0						COSM1375326,CO	rs149167939		Missense_Mutation
OSGIN1	p.R438H	1	0						COSM1380141	rs142452879		Missense_Mutation
UNC93A	p.V445A	2	0						COSM140578			Missense_Mutation
SUSD2	p.A485V	2	0						COSM1415232	rs116820424		Missense_Mutation
MUC17	p.I1695M	4	0						COSM1446753			Missense_Mutation
MUC2	p.T1728S	4	0						COSM145643,COS	rs113138128		Missense_Mutation
CFH	p.Y1058H	2	0						COSM146717	rs55679475		Missense_Mutation
CFH	p.V1060L	2	0						COSM146718	rs55771831		Missense_Mutation
LAX1	p.S303T	2	0						COSM146742,COS	rs75396540		Missense_Mutation
WDR64	p.V1066A	1	0						COSM146841	rs151295225		Missense_Mutation
WDR64	p.V1066A	1	0						COSM146841	rs151295225		Missense_Mutation
JMJD1C	p.T516S	1	0						COSM146944	rs41274074		Missense_Mutation
MUC2	p.G1738S	5	0						COSM1475016,CO	rs199525790		Missense_Mutation
MUC5B	p.G2588R	1	0						COSM1475202,CO	rs200110372		Missense_Mutation
SYT16	p.S291T	1	0						COSM147790	rs146869637		Missense_Mutation
NEDD4	p.G166S	2	0						COSM147923	rs12593255		Missense_Mutation
DNAH17	p.I4426V	2	0						COSM148353	rs78023288		Missense_Mutation
PNMAL1	p.P319S	3	0						COSM148674	rs79729613		Missense_Mutation

SLC25A38	p.M128V	2	0						COSM149363	rs146940902		Missense_Mutation
KLHL40	p.A601T	1	0						COSM149366	rs144659891		Missense_Mutation
ZNF318	p.A1580T	1	0						COSM150091	rs3734684		Missense_Mutation
CENPF	p.E2553K	1	0						COSM1501069	rs117254535		Missense_Mutation
PPP1R3A	p.R883S	1	0						COSM150375	rs1800000		Missense_Mutation
KRTAP9-8	p.I60T	1	0						COSM1521033	rs201454642		Missense_Mutation
KRTAP4-7	p.Q31E	2	0						COSM1521063,C	rs200397258		Missense_Mutation
ESX1	p.P305T	1	0						COSM1555892	rs573223696		Missense_Mutation
ESX1	p.W306G	1	0						COSM1555893	rs201963432		Missense_Mutation
GLYAT	p.R131H	1	0						COSM1604794	rs117149346		Missense_Mutation
ADAMTS7	p.G1288R	1	0						COSM1608730	rs201097994		Missense_Mutation
DMKN	p.G288S	1	0						COSM1612077,COSM1392865			Missense_Mutation
CATSPERG	p.M1137T	1	0						COSM1630902,C	rs2286550		Missense_Mutation
HLA-DRB5	p.F96I	3	0						COSM1672304	rs41562819		Missense_Mutation
PRMT8	p.S31P	4	0						COSM1724237,COSM1724238			Missense_Mutation
ZDHHC11	p.T339K	2	0						COSM1735550,C	rs2335585		Missense_Mutation
AKR1B15	p.A95T	2	0						COSM1739384,C	rs201714104		Missense_Mutation
PAAF1	p.G213A	1	0						COSM1739836			Missense_Mutation
ADAMTS7	p.P1270S	1	0						COSM1749334	rs76784555		Missense_Mutation
ARMC4	p.R639Q	2	0						COSM184587	rs148908705		Missense_Mutation
OAS1	p.K172R	2	0						COSM1948853,COSM1948855,COSM1948854			Missense_Mutation
OAS1	p.K172R	2	0						COSM1948853,COSM1948855,COSM1948854			Missense_Mutation
MUC6	p.V105I	1	0						COSM2108788,C	rs148640037		Missense_Mutation
C8A	p.R552G	2	0						COSM216350	rs56334452		Missense_Mutation
MAP1A	p.T2643A	1	0						COSM2188498	rs192770595		Missense_Mutation
OR2T27	p.G235R	1	0						COSM224829	rs151290740		Missense_Mutation
PLIN4	p.V903M	2	0						COSM226374,COSM1600095			Missense_Mutation
OR2L8	p.L132P	2	0						COSM226497	rs202165563		Missense_Mutation
PRDM9	p.N843D	1	0						COSM226557			Missense_Mutation
MUC6	p.P2011S	1	0						COSM227561			Missense_Mutation
OR6C76	p.H307Q	5	0						COSM227846	rs77587450		Missense_Mutation
FAM186A	p.E1598D	2	0						COSM229228			Missense_Mutation
RSPH10B	p.M765T	1	0						COSM229737	rs182575261		Missense_Mutation
PSKH2	p.K86E	1	0						COSM241280			Missense_Mutation
KRTAP1-1	p.S34C	2	0						COSM245362			Missense_Mutation
KIAA2026	p.T1259I	1	0						COSM2777552,C	rs201838248		Missense_Mutation
MUC2	p.T1705I	4	0						COSM288230,COSM288231			Missense_Mutation
KRTAP10-6	p.P216S	1	0						COSM296234	rs9977124		Missense_Mutation
LILRA1	p.I77L	4	0						COSM299682	rs147848714		Missense_Mutation
PDZD2	p.E2533K	2	0						COSM3014699	rs201398838		Missense_Mutation
CCDC108	p.R922Q	2	0						COSM3044291	rs186211637		Missense_Mutation
CEP68	p.E93K	2	0						COSM3055221	rs528823795		Missense_Mutation
ROBO3	p.R1367Q	1	0						COSM307110	rs200255001		Missense_Mutation
CCDC129	p.T780M	1	0						COSM3227791,C	rs200617855		Missense_Mutation
PMFBP1	p.M582I	1	0						COSM3276593,COSM3512002			Missense_Mutation
SLC35G5	p.W103R	2	0						COSM328226	rs76944947		Missense_Mutation
IGFN1	p.E2114K	1	0						COSM3360571			Missense_Mutation
NACC1	p.A237V	1	0						COSM3378617	rs201985022		Missense_Mutation
ZNF71	p.R198C	1	0						COSM3404685			Missense_Mutation
PCNT	p.A1904T	1	0						COSM356963	rs200426591		Missense_Mutation

KRTAP4-9	p.K136R	1	0						COSM363774,COSM363775			Missense_Mutation
METTL2B	p.C124S	5	0						COSM3662914			Missense_Mutation
ANO9	p.R159H	1	0						COSM3676155	rs78972632		Missense_Mutation
OR51G1	p.C317Y	3	0						COSM3676166	rs139629765		Missense_Mutation
ZNF215	p.S263F	1	0						COSM3676284	rs11041115		Missense_Mutation
CCNL2	p.P480S	2	0						COSM3676725	rs139345631		Missense_Mutation
SLX4	p.H1290N	2	0						COSM3679905	rs112596894		Missense_Mutation
PHLPP2	p.N343S	2	0						COSM3679994,CO	rs139051907		Missense_Mutation
B3GNTL1	p.R286H	1	0						COSM3680625	rs148957583		Missense_Mutation
PTH2R	p.V350A	2	0						COSM3682533	rs143730934		Missense_Mutation
PRMT10	p.V777I	1	0						COSM3683235	rs77293186		Missense_Mutation
EFCAB9	p.Y13C	1	0						COSM3683822	rs17074410		Missense_Mutation
NCAPG2	p.E867D	1	0						COSM3685126,CO	rs3214000		Missense_Mutation
SHARPIN	p.P311R	1	0						COSM3685343	rs35844464		Missense_Mutation
SPTBN5	p.H1912R	1	0						COSM3690396	rs8034735		Missense_Mutation
HES6	p.R218Q	2	0						COSM3709518	rs3739061		Missense_Mutation
C2orf81	p.V243A	3	0						COSM3721068	rs116859876		Missense_Mutation
ZNF285	p.A60G	1	0						COSM3746499	rs117953191		Missense_Mutation
GCFC2	p.V61I	1	0						COSM3749677	rs10177030		Missense_Mutation
CALHM3	p.L270P	1	0						COSM3751509,CO	rs112393722		Missense_Mutation
GNAS	p.A436D	1	0						COSM3758660	rs61749698		Missense_Mutation
AHRR	p.G391V	1	0						COSM3761240	rs2303738		Missense_Mutation
MUC17	p.L1112P	5	0						COSM3762266	rs147173571		Missense_Mutation
RP1L1	p.E1343A	1	0						COSM3762978			Missense_Mutation
RP1L1	p.E1343K	1	0						COSM3762979			Missense_Mutation
PRODH	p.T275N	4	0						COSM3766680	rs5747933		Missense_Mutation
PRODH	p.T275N	4	0						COSM3766680	rs5747933		Missense_Mutation
IL15RA	p.T111M	1	0						COSM3769142,CO	rs41294171		Missense_Mutation
IL15RA	p.T111M	1	0						COSM3769142,CO	rs41294171		Missense_Mutation
IGFN1	p.E1988G	1	0						COSM3773457			Missense_Mutation
SSC5D	p.H1186Q	1	0						COSM3773912	rs74585733		Missense_Mutation
SSC5D	p.P1305A	1	0						COSM3773914	rs200305118		Missense_Mutation
SSC5D	p.Y1312D	1	0						COSM3773915	rs200457993		Missense_Mutation
BIRC6	p.T894M	1	0						COSM3773970,CO	rs34996177		Missense_Mutation
HLA-DRB1	p.F55L	1	0						COSM3774250	rs569286159		Missense_Mutation
FUT3	p.R60P	2	0						COSM3783299			Missense_Mutation
HRNR	p.L1722S	2	0						COSM3801869	rs34655925		Missense_Mutation
FCRL6	p.E409K	2	0						COSM3802431			Missense_Mutation
IER5	p.E156Q	1	0						COSM3803073			Missense_Mutation
MUC12	p.E4106K	3	0						COSM3831415,COSM3831414			Missense_Mutation
NFE2L3	p.T332I	1	0						COSM3832562	rs147199325		Missense_Mutation
ETV5	p.A177P	2	0						COSM3846945,COSM3846947,COSM3846946			Missense_Mutation
MMP9	p.T474P	1	0						COSM3911471			Missense_Mutation
MUC6	p.Y1995H	4	0						COSM3927643,COSM3927644			Missense_Mutation
MUC6	p.F1989S	4	0						COSM3927645,COSM3927646			Missense_Mutation
MUC2	p.T1587S	4	0						COSM3927683,CO	rs111170565		Missense_Mutation
MUC2	p.T1649S	4	0						COSM3927686,COSM3927685			Missense_Mutation
MUC2	p.P1650T	4	0						COSM3927688,COSM3927687			Missense_Mutation
SFTPA2	p.A19V	1	0						COSM3931135	rs201847938		Missense_Mutation
SELPLG	p.T242P	1	0						COSM3931481,COSM3931482			Missense_Mutation

TAS2R31	p.R295W	4	0						COSM3931508	rs199894662		Missense_Mutation
SEZ6L2	p.T352N	3	0						COSM3932316,CO	rs75331747		Missense_Mutation
SIRPA	p.T50S	2	0						COSM3933796,CO	rs17855609		Missense_Mutation
OPN1LW	p.I178V	1	0						COSM3964941,CO	rs145009674		Missense_Mutation
ATHL1	p.R358Q	2	0						COSM3979554,CO	rs114112322		Missense_Mutation
ELN	p.A71V	1	0						COSM3982262,CO	rs41350445		Missense_Mutation
C1orf177	p.C343Y	2	0						COSM3997601,CO	rs75191355		Missense_Mutation
ZNF438	p.D613G	2	0						COSM3997947,CO	rs182928199		Missense_Mutation
RNF26	p.E360D	2	0						COSM3998258	rs78682406		Missense_Mutation
METTL15	p.T67A	1	0						COSM3998383,CO	rs141648004		Missense_Mutation
OR5B3	p.I35F	2	0						COSM3998487	rs17152659		Missense_Mutation
AHNAK	p.N3637S	2	0						COSM3998524	rs117618959		Missense_Mutation
KIAA0586	p.G1088D	1	0						COSM3999299,CO	rs77860620		Missense_Mutation
ALPK3	p.T761M	1	0						COSM3999646,CO	rs16974569		Missense_Mutation
CCP110	p.T560M	2	0						COSM3999755	rs78180055		Missense_Mutation
CHST5	p.R184Q	1	0						COSM3999925	rs201072221		Missense_Mutation
CCDC102B	p.R371T	2	0						COSM4000459,CO	rs117721069		Missense_Mutation
COL6A6	p.A1809T	1	0						COSM4002461,CO	rs143290812		Missense_Mutation
ALPK1	p.N916D	1	0						COSM4002909	rs2074381		Missense_Mutation
LIFR	p.D578N	4	0						COSM4003522,CO	rs3729740		Missense_Mutation
DAB2	p.T505I	1	0						COSM4003535	rs145194026		Missense_Mutation
NHSL1	p.R260H	2	0						COSM4003700,CO	rs76776064		Missense_Mutation
C6orf136	p.T43A	3	0						COSM4003876	rs117013517		Missense_Mutation
PMPCB	p.A375T	1	0						COSM4004227	rs117074402		Missense_Mutation
HLA-DRB1	p.Y89S	2	0						COSM4006429	rs1059586		Missense_Mutation
MUC2	p.P1811T	1	0						COSM4017712,CO	rs202172572		Missense_Mutation
KRTAP9-2	p.P84T	1	0						COSM404504	rs34024914		Missense_Mutation
SCAPER	p.A1140V	1	0						COSM4057051,CO	rs149493455		Missense_Mutation
KRTAP1-1	p.S17G	1	0						COSM4129996	rs201337527		Missense_Mutation
SSC5D	p.M1303T	1	0						COSM4132633	rs202130460		Missense_Mutation
SBK3	p.P35R	1	0						COSM4132635	rs56258384		Missense_Mutation
SIRPB1	p.L95F	1	0						COSM4134247			Missense_Mutation
POTED	p.M43V	4	0						COSM4134833			Missense_Mutation
POTED	p.E87D	4	0						COSM4134835			Missense_Mutation
POTED	p.M93T	3	0						COSM4134836			Missense_Mutation
POTED	p.M93I	2	0						COSM4134837			Missense_Mutation
PDE4DIP	p.K2373Q	3	0						COSM4140793,CO	rs2798855		Missense_Mutation
IGFN1	p.E2155G	1	0						COSM4143193	rs371677542		Missense_Mutation
CITED4	p.R57P	2	0						COSM4143951	rs118006907		Missense_Mutation
MUC2	p.T1744P	3	0						COSM4145282,CO	rs55824838		Missense_Mutation
MUC2	p.S1784G	1	0						COSM4145285,CO	rs56104295		Missense_Mutation
MUC2	p.V1790L	1	0						COSM4145290,CO	rs79238439		Missense_Mutation
TAS2R30	p.T312R	5	0						COSM4146644	rs78329184		Missense_Mutation
GOLGA6L2	p.K457E	1	0						COSM4148540,COSM4148539,COSM4148541			Missense_Mutation
AVPR2	p.G12E	2	0						COSM4156719	rs2071126		Missense_Mutation
ZNF717	p.V132I	1	0						COSM4158584,COSM4158583			Missense_Mutation
MUC12	p.R726S	5	0						COSM4161560,COSM4161559			Missense_Mutation
MUC12	p.P4303T	1	0						COSM4161602,CO	rs200533145		Missense_Mutation
KRTAP4-7	p.S57P	2	0						COSM417544,COS	rs189343211		Missense_Mutation
OR2T34	p.H109Y	1	0						COSM425943	rs139616012		Missense_Mutation

CPNE7	p.P558L	2	0				■	■	COSM435940	rs3751682		Missense_Mutation
TPRX1	p.S216P	1	0			■			COSM439880	rs201007421		Missense_Mutation
MUC2	p.P1675T	1	0					■	COSM466294,CO	rs56299570		Missense_Mutation
ZDHHC11	p.F245L	1	0	■	■		■		COSM87837,COS	rs201008730		Missense_Mutation
RPTN	p.M538I	1	0	■					COSM896016			Missense_Mutation
FAM186A	p.E1598A	1	0					■	COSM940337			Missense_Mutation
FAM186A	p.T1458P	2	0		■		■		COSM940340			Missense_Mutation
FSIP1	p.V437I	1	0	■					COSM961053			Missense_Mutation
STK36	p.R389Q	1	0			■			COSM98825	rs149471679		Missense_Mutation
SLK	p.Q670R	1	0	■	■	■	■	■	COSM98957	rs117017856		Missense_Mutation
PIK3R2	p.P4S	1	0	■	■	■	■	■	COSM99103	rs142933317		Missense_Mutation
ACSM2A	p.R115*	3	0		■				COSM1599948	rs59261767		Nonsense_Mutation
ZNF729	p.R121*	1	0	■					COSM993877			Nonsense_Mutation
RBFOX1	p.T379T	2	0			■			COSM1470923,CC	rs150941982		Silent
PLEKHG2	p.D134D	1	0	■					COSM1744125,COSM1744126,COSM1744124			Silent
KSR1	p.F473F	3	0	■	■	■	■	■	COSM3680116,CC	rs2289568		Silent
SUN1	p.R718R	3	0					■	COSM3982285,CC	rs61744747		Silent
MUC16	p.Y13216Y	1	0	■	■	■	■	■	COSM4132839,CC	rs79341062		Silent
AKR1C1	p.H222H	3	0	■	■		■		COSM4144729	rs377035250		Silent
PRKAR1B	p.A328A	4	0	■	■		■	■	COSM4162325	rs28626752		Silent
FERMT3	p.P483P	3	0		■	■			COSM429520,CO	rs3802932		Silent
SIK3	p.Q702Q	1	0	■	■		■	■	COSM99013	rs560511616		Silent
IFNAR2	p.0	1	0			■			COSM1130550			Splice_Site
ZNF717	p.A43T	1	0	■				■	COSM3727848,CC	rs199946555		Splice_Site
PLCH2	p.R989P	1	0			■						Missense_Mutation
PRAMEF1	p.Y194D	1	0				■					Missense_Mutation
KIF17	p.T992P	1	0		■							Missense_Mutation
PNRC2	p.R82S	2	0			■						Missense_Mutation
AUNIP	p.M115T	1	0			■						Missense_Mutation
PTAFR	p.S313N	2	0	■								Missense_Mutation
CLSPN	p.A126V	1	0	■								Missense_Mutation
PLK3	p.Q363E	2	0	■								Missense_Mutation
CYP4A11	p.D14G	1	0	■								Missense_Mutation
NUDT17	p.V4E	2	0				■					Missense_Mutation
TCHH	p.L413Q	1	0			■						Missense_Mutation
HRNR	p.G2410R	1	0				■					Missense_Mutation
HRNR	p.R1996H	1	0				■					Missense_Mutation
FLG	p.E1757G	1	0			■						Missense_Mutation
LCE6A	p.R22K	2	0			■						Missense_Mutation
SPTA1	p.E851Q	1	0				■					Missense_Mutation
ARHGAP30	p.G381S	1	0	■								Missense_Mutation
FCGR2A	p.L274P	5	0					■				Missense_Mutation
TDRD5	p.G767D	1	0		■							Missense_Mutation
IER5	p.G124A	1	0				■					Missense_Mutation
IER5	p.R153K	1	0				■					Missense_Mutation
LMOD1	p.V214G	1	0		■							Missense_Mutation
C4BPB	p.T46I	1	0			■						Missense_Mutation
ENAH	p.D242E	1	0	■								Missense_Mutation
ZNF678	p.S204T	1	0				■					Missense_Mutation
ZNF678	p.E212D	1	0		■							Missense_Mutation

OBSCN	p.H4090R	2	0									Missense_Mutation
ACTN2	p.A882S	1	0									Missense_Mutation
WDR64	p.S825A	2	0									Missense_Mutation
WDR64	p.S825A	2	0									Missense_Mutation
OR14A2	p.M295I	2	0									Missense_Mutation
OR2L5	p.M312V	1	0									Missense_Mutation
OR2T5	p.H27L	1	0									Missense_Mutation
ADARB2	p.P271T	1	0									Missense_Mutation
TRDMT1	p.N242D	1	0									Missense_Mutation
TRDMT1	p.N242D	1	0									Missense_Mutation
C10orf112	p.I1203L	1	0									Missense_Mutation
C10orf112	p.I1203L	1	0									Missense_Mutation
WDFY4	p.E2044G	1	0									Missense_Mutation
TTC18	p.V281L	1	0									Missense_Mutation
LIPM	p.S270G	1	0									Missense_Mutation
CPN1	p.R24H	1	0									Missense_Mutation
TACC2	p.Q783H	2	0									Missense_Mutation
PDDC1	p.D144V	1	0									Missense_Mutation
MUC2	p.P1723T	1	0									Missense_Mutation
MUC2	p.P1723L	3	0									Missense_Mutation
OR51H1P	p.K225N	1	0									Missense_Mutation
FAM160A2	p.R631G	1	0									Missense_Mutation
DNHD1	p.N4632K	2	0									Missense_Mutation
SLC6A5	p.A93S	2	0									Missense_Mutation
OR4P4	p.A85T	2	0									Missense_Mutation
OR4P4	p.A85E	2	0									Missense_Mutation
OR5L1	p.S68F	1	0									Missense_Mutation
SPDYC	p.S227I	1	0									Missense_Mutation
FAM181B	p.G299V	2	0									Missense_Mutation
AMOTL1	p.S864I	1	0									Missense_Mutation
MMP20	p.H390N	2	0									Missense_Mutation
NPAT	p.S108T	1	0									Missense_Mutation
NXPE4	p.H460R	2	0									Missense_Mutation
FXYD6-FXYD2	p.V6F	1	0									Missense_Mutation
FGF6	p.G71R	1	0									Missense_Mutation
C1S	p.P338A	1	0									Missense_Mutation
MANSC1	p.G144D	1	0									Missense_Mutation
OR6C76	p.Q296K	1	0									Missense_Mutation
OR6C76	p.R303K	1	0									Missense_Mutation
TMPO	p.G80R	1	0									Missense_Mutation
ISCU	p.A13V	1	0									Missense_Mutation
SETD1B	p.A1463P	1	0									Missense_Mutation
AC226150.4	p.E70K	1	0									Missense_Mutation
FLT1	p.A289D	1	0									Missense_Mutation
UTP14C	p.M206V	1	0									Missense_Mutation
PCDH8	p.A628T	1	0									Missense_Mutation
CCDC168	p.T90A	1	0									Missense_Mutation
ERCC5	p.R435K	1	0									Missense_Mutation
ATP11A	p.K500T	1	0									Missense_Mutation
ATP11A	p.K500T	1	0									Missense_Mutation

TRIP11	p.K1354N	1	0				■					Missense_Mutation
TRIP11	p.K1354N	1	0	■	■			■				Missense_Mutation
COX8C	p.R5L	1	0			■						Missense_Mutation
RTL1	p.H1276P	1	0				■					Missense_Mutation
RCOR1	p.M467L	1	0	■								Missense_Mutation
MAGEL2	p.K130T	2	0					■				Missense_Mutation
TJP1	p.T991A	1	0			■						Missense_Mutation
NDUFAF1	p.R95K	1	0					■				Missense_Mutation
MAP1A	p.S2561T	2	0					■				Missense_Mutation
MESDC1	p.R32K	1	0			■						Missense_Mutation
ACAN	p.E785Q	2	0			■						Missense_Mutation
CCP110	p.F519V	1	0									Missense_Mutation
PDILT	p.E519G	1	0	■								Missense_Mutation
IGSF6	p.I9V	1	0			■						Missense_Mutation
HSF4	p.R284K	2	0		■							Missense_Mutation
HSF4	p.R284K	2	0					■				Missense_Mutation
HYDIN	p.I586M	2	0		■							Missense_Mutation
ZFHX3	p.T2791A	2	0			■						Missense_Mutation
ZNF469	p.S656P	1	0			■						Missense_Mutation
ZNF469	p.P3067S	2	0		■							Missense_Mutation
RILP	p.A395S	2	0		■							Missense_Mutation
GGT6	p.T423S	1	0		■							Missense_Mutation
CD68	p.A10T	1	0					■				Missense_Mutation
ARHGEF15	p.H252P	4	0				■					Missense_Mutation
ALDH3A1	p.I75L	1	0			■						Missense_Mutation
C17orf66	p.M403I	1	0	■								Missense_Mutation
C17orf66	p.E396K	1	0			■						Missense_Mutation
C17orf66	p.R376K	2	0	■	■							Missense_Mutation
CCL23	p.Q55K	2	0					■				Missense_Mutation
PCGF2	p.A271S	1	0			■						Missense_Mutation
KRTAP16-1	p.P350L	1	0					■				Missense_Mutation
WNK4	p.P128L	2	0	■								Missense_Mutation
KIF2B	p.H189L	2	0	■								Missense_Mutation
TXNDC2	p.F54L	1	0					■				Missense_Mutation
RBBP8	p.R300K	2	0	■	■	■						Missense_Mutation
CDH19	p.K337E	1	0					■				Missense_Mutation
CD226	p.S129N	1	0		■							Missense_Mutation
RTTN	p.T1626M	1	0		■							Missense_Mutation
KDM4B	p.P492L	2	0					■				Missense_Mutation
PPAN-P2RY11	p.R264Q	2	0				■					Missense_Mutation
PPAN-P2RY11	p.R264Q	2	0					■				Missense_Mutation
S1PR2	p.R307W	1	0	■								Missense_Mutation
ZNF257	p.V47A	2	0					■				Missense_Mutation
ZNF254	p.I286T	1	0					■				Missense_Mutation
WDR87	p.K2270E	1	0					■				Missense_Mutation
CATSPERG	p.M643V	2	0				■					Missense_Mutation
NUMBL	p.M502V	1	0		■							Missense_Mutation
ZNF285	p.R222H	1	0		■							Missense_Mutation
BCL3	p.S382T	1	0					■				Missense_Mutation
IRF2BP1	p.T416S	2	0		■							Missense_Mutation

MYPOP	p.A129T	1	0									Missense_Mutation
IGFL3	p.S51L	2	0									Missense_Mutation
TRPM4	p.P750L	1	0									Missense_Mutation
ALDH16A1	p.E611Q	1	0									Missense_Mutation
ZNF880	p.K537N	1	0									Missense_Mutation
ZNF765	p.S189F	1	0									Missense_Mutation
ZNF331	p.G423R	1	0									Missense_Mutation
SSC5D	p.M1303I	1	0									Missense_Mutation
ZSCAN4	p.V426I	1	0									Missense_Mutation
WDR43	p.H412R	1	0									Missense_Mutation
STARD7	p.N110D	2	0									Missense_Mutation
FSIP2	p.E5751K	2	0									Missense_Mutation
MROH2A	p.W978G	1	0									Missense_Mutation
GPC1	p.D187E	1	0									Missense_Mutation
GPC1	p.D187E	1	0									Missense_Mutation
GPC1	p.D187E	1	0									Missense_Mutation
SIRPG	p.V130M	1	0									Missense_Mutation
ZNF343	p.V578I	1	0									Missense_Mutation
SEC23B	p.S127F	1	0									Missense_Mutation
FRG1B	p.S160I	3	0									Missense_Mutation
MROH8	p.N711H	1	0									Missense_Mutation
MROH8	p.N711H	1	0									Missense_Mutation
MROH8	p.N711H	1	0									Missense_Mutation
ZMYND8	p.E642D	2	0									Missense_Mutation
PREX1	p.R778P	1	0									Missense_Mutation
ZNF217	p.L547F	1	0									Missense_Mutation
ANKRD60	p.V77F	1	0									Missense_Mutation
PHACTR3	p.P142S	1	0									Missense_Mutation
LAMA5	p.E2378K	1	0									Missense_Mutation
RTEL1	p.G66S	1	0									Missense_Mutation
AL158091.1	p.G98R	1	0									Missense_Mutation
TMPRSS15	p.Q239H	1	0									Missense_Mutation
KRTAP10-8	p.R214H	1	0									Missense_Mutation
EFCAB6	p.A1416V	1	0									Missense_Mutation
EFCAB6	p.Q586E	1	0									Missense_Mutation
NUP210	p.H1227Q	1	0									Missense_Mutation
GOLGA4	p.V264A	1	0									Missense_Mutation
LAMB2	p.M1384K	2	0									Missense_Mutation
LAMB2	p.A766T	1	0									Missense_Mutation
ZNF717	p.S102L	2	0									Missense_Mutation
ZNF717	p.S42L	1	0									Missense_Mutation
HHLA2	p.N303H	1	0									Missense_Mutation
CCDC37	p.T411M	1	0									Missense_Mutation
PIK3R4	p.H284P	1	0									Missense_Mutation
FAM194A	p.L60V	1	0									Missense_Mutation
RARRES1	p.E85G	1	0									Missense_Mutation
CRIPAK	p.H326P	1	0									Missense_Mutation
YIPF7	p.T127S	1	0									Missense_Mutation
ALPK1	p.G681V	1	0									Missense_Mutation
MAML3	p.P1109S	1	0									Missense_Mutation

LRAT	p.T42S	1	0					■				Missense_Mutation
C4orf47	p.I226T	1	0					■				Missense_Mutation
SORBS2	p.N338K	1	0				■					Missense_Mutation
SORBS2	p.N338K	1	0									Missense_Mutation
SORBS2	p.N338K	1	0					■				Missense_Mutation
CDH9	p.R137L	2	0					■				Missense_Mutation
RXFP3	p.P313L	1	0			■						Missense_Mutation
TTC23L	p.E157K	1	0		■							Missense_Mutation
MAP3K1	p.T103I	1	0				■					Missense_Mutation
SREK1	p.K457N	1	0	■								Missense_Mutation
ZFYVE16	p.T680I	1	0				■					Missense_Mutation
LYRM7	p.V40L	1	0			■						Missense_Mutation
PCDHA12	p.H392L	1	0			■						Missense_Mutation
PCDHGA3	p.P79R	2	0	■								Missense_Mutation
PCDHGA10	p.S173R	2	0				■					Missense_Mutation
PCDHGB7	p.S385T	1	0			■						Missense_Mutation
C5orf54	p.P70A	2	0			■						Missense_Mutation
GCNT6	p.I17S	5	0				■					Missense_Mutation
HIVEP1	p.L1652V	2	0		■	■						Missense_Mutation
HLA-DRB5	p.R100K	3	0			■						Missense_Mutation
HLA-DRB1	p.Q178H	1	0	■		■						Missense_Mutation
PNPLA1	p.Y402H	1	0				■					Missense_Mutation
TDRD6	p.G761A	2	0			■						Missense_Mutation
DEFB133	p.R35I	2	0				■					Missense_Mutation
LCA5	p.N75D	1	0				■					Missense_Mutation
NCOA7	p.N575H	1	0					■				Missense_Mutation
CHST12	p.Q354E	2	0		■							Missense_Mutation
TNRC18	p.S2561I	1	0									Missense_Mutation
RNF216	p.E707Q	1	0	■								Missense_Mutation
USP42	p.R1011G	2	0		■							Missense_Mutation
COL28A1	p.K848E	2	0				■					Missense_Mutation
CDCA7L	p.S163T	1	0	■								Missense_Mutation
AOAH	p.L657F	1	0	■								Missense_Mutation
ZMIZ2	p.A731S	1	0			■						Missense_Mutation
PKD1L1	p.A2362S	1	0	■								Missense_Mutation
PKD1L1	p.S1651F	1	0		■							Missense_Mutation
TRIM50	p.K297R	2	0					■				Missense_Mutation
ABHD11	p.V34L	2	0			■						Missense_Mutation
SRCRB4D	p.G318R	1	0			■						Missense_Mutation
BAIAP2L1	p.N403D	2	0			■						Missense_Mutation
MUC12	p.T354I	1	0					■				Missense_Mutation
MUC17	p.P753S	1	0				■					Missense_Mutation
MUC17	p.P1033S	1	0	■								Missense_Mutation
MUC17	p.T1069P	3	0			■						Missense_Mutation
MUC17	p.P1343T	4	0			■	■	■				Missense_Mutation
MUC17	p.R2199S	1	0			■						Missense_Mutation
MUC17	p.E2236G	3	0	■								Missense_Mutation
PPP1R3A	p.E392D	2	0					■				Missense_Mutation
AASS	p.R745S	2	0					■				Missense_Mutation
OR2A7	p.Y272S	3	0			■						Missense_Mutation

MCPH1	p.H634R	2	0									Missense_Mutation
HR	p.R154Q	2	0									Missense_Mutation
SPIDR	p.G279D	2	0									Missense_Mutation
SPIDR	p.G279D	2	0									Missense_Mutation
CRH	p.A95T	1	0									Missense_Mutation
CPNE3	p.G471D	1	0									Missense_Mutation
GDF6	p.S29L	2	0									Missense_Mutation
PHF20L1	p.S903T	2	0									Missense_Mutation
TRAPPC9	p.N645H	2	0									Missense_Mutation
CYP11B2	p.A21T	1	0									Missense_Mutation
EPPK1	p.Q1065R	1	0									Missense_Mutation
EPPK1	p.M238V	1	0									Missense_Mutation
ZNF250	p.N170S	1	0									Missense_Mutation
GLIS3	p.E472D	1	0									Missense_Mutation
KDM4C	p.I603F	1	0									Missense_Mutation
DENND4C	p.N1592K	1	0									Missense_Mutation
TJP2	p.E262K	2	0									Missense_Mutation
TMEM2	p.A1030G	1	0									Missense_Mutation
VPS13A	p.S1385A	2	0									Missense_Mutation
SHC3	p.K489N	1	0									Missense_Mutation
NOL8	p.H423Y	1	0									Missense_Mutation
ECM2	p.S304F	1	0									Missense_Mutation
TSTD2	p.H140L	1	0									Missense_Mutation
TBC1D2	p.A348T	1	0									Missense_Mutation
TBC1D2	p.A257S	1	0									Missense_Mutation
OR13C2	p.T91S	1	0									Missense_Mutation
PTGR1	p.T244A	1	0									Missense_Mutation
CDK5RAP2	p.V1183L	1	0									Missense_Mutation
TRUB2	p.S60G	1	0									Missense_Mutation
SARDH	p.N845D	2	0									Missense_Mutation
SNAPC4	p.N1446D	2	0									Missense_Mutation
HDAC6	p.P1033S	1	0									Missense_Mutation
IQSEC2	p.S1474R	1	0									Missense_Mutation
LAS1L	p.P576T	2	0									Missense_Mutation
ZCCHC5	p.A142S	2	0									Missense_Mutation
ARMCX4	p.G1533R	1	0									Missense_Mutation
ARMCX2	p.R246T	1	0									Missense_Mutation
ARMCX2	p.D64N	2	0									Missense_Mutation
MAP7D3	p.E360K	1	0									Missense_Mutation
NBPF14	p.E568*	1	0									Nonsense_Mutation
NBPF14	p.E568*	1	0									Nonsense_Mutation
IGFN1	p.E2099*	1	0									Nonsense_Mutation
MS4A18	p.S142*	1	0									Nonsense_Mutation
WDR90	p.S741*	1	0									Nonsense_Mutation
RDH16	p.*318C	1	0									Nonstop_Mutation
ACOX2	p.*668L	2	0									Nonstop_Mutation
NBPF14	p.C550C	1	0									Silent
RNASEL	p.K533K	2	0									Silent
ADAMTS8	p.G35G	1	0									Silent
DMKN	p.D416D	1	0									Silent

DMKN	p.D416D	1	0									Silent
GPR75-ASB3	p.L116L	1	0									Silent
SUN1	p.L358L	2	0									Silent
ZP3	p.V376V	1	0									Silent
KIAA1210	p.L981L	2	0									Silent
GADL1	p.N113S	1	0									Splice_Site
POT1	p.A532A	1	0									Splice_Site

Table S5: Clinical characteristics of gallbladder primary tumor cohort

NGS	ADLAB ID	PATIENT ID	Age	Sex	T stage	N stage	AJCC Stage	Gallstone	Lymphovascular emboli	Perineural invasion	OS_months	Survival Status	Degree of differentiation
Exome	AD0740	1T	46	Female	pT3	N1	IIIB	No	No	No	40	Alive	Moderate
Exome	AD0761	2T	41	Female	Cannot acces	N0	I	Yes	No	No	30	Alive	Poor
Exome	AD0743	3T	73	Female	pT2	N1	IIIB	Yes	No	No	43	Dead	Moderate
Exome	AD0741	4T	75	Female	pT2	N0	II	No	No	No	24	Alive	Moderate
Exome	AD0745	5T	63	Female	pT2	N0	II	No	No	No	77	Dead	Poor
Exome	AD0750	6T	75	Female	pT2	N0	II	Yes	No	No	32	Dead	Moderate
Exome	AD0738	7T	49	Female	pT2	N1	IIIB	No	No	No	7	Alive	Moderate
Exome	AD0739	8T	64	Male	pT2	N0	II	No	No	No	16	Dead	Moderate
Exome	AD0748	9T	52	Male	pT2	N1	IIIB	Yes	Yes	Yes	79	Alive	Moderate
Exome	AD0744	10T	70	Male	pT1	N0	I	Yes	No	No	20	Dead	Moderate
Exome	AD0438	11T	75	Male	pT1	N1	IIIB	No	No	No	26	Dead	Moderate
Exome	AD0440	12T	60	Male	pT2	N0	II	Yes	No	No	22	Alive	Moderate
Exome	AD0758	13T	51	Male	pT1	N0	I	No	No	No	38	Dead	Moderate
Exome	AD0736	14T	64	Male	pT2	N0	II	Yes	No	No	17	Dead	Moderate
Exome	AD0747	15T	42	Male	pT2	N0	II	Yes	No	No	69	Alive	Moderate
Exome	AD0477	16T	42	Male	pT1	N0	I	Yes	No	No	44	Alive	Moderate
Exome	AD0742	17T	54	Male	pT2	N0	II	No	No	No	28	Alive	Moderate
FFPE	AD1138	18T	58	Female	pT2	N0	II	No	No	No	54	Alive	Moderate
FFPE	AD1139	19T	55	Female	pT2	N2	IVB	No	No	No	13	Dead	Poor
FFPE	AD1140	20T	31	Female	pT2	N0	II	No	No	No	49	Alive	Poor
FFPE	AD1141	21T	65	Female	pT2	N0	II	No	No	No	17	Alive	Poor
FFPE	AD1142	22T	48	Male	pT2	N2	IVB	No	No	No	25	Alive	Poor
FFPE	AD1143	23T	55	Male	pT2	N1	IIIB	No	No	Yes	17	Dead	Moderate
FFPE	AD1144	24T	35	Male	pT2	N1	IIIB	No	No	No	12	Dead	Moderate
FFPE	AD1145	25T	69	Male	pT2	N0	II	No	No	No	31	Dead	Moderate
FFPE	AD1146	26T	50	Female	pT3	N0	IIIA	No	No	No	37	Alive	No info
FFPE	AD1147	27T	44	Female	pT3	N0	IIIA	No	No	No	25	Alive	No info
FFPE	AD1148	28T	74	Male	pT3	N0	IIIA	No	No	No	39	Alive	Moderate
FFPE	AD1149	29T	44	Female	pT3	N0	IIIA	No	Yes	No	40	Alive	Moderate
FFPE	AD1150	30T	39	Female	pT3	N2	IVB	No	Yes	No	40	Alive	Well
FFPE	AD1151	31T	43	Female	pT3	N0	IIIA	No	No info	No info	35	Alive	Well
FFPE	AD1152	32T	41	Female	pT3	N1	IIIB	No	No	Yes	37	Alive	Moderate
FFPE	AD1153	33T	57	Female	pT3	N2	IVB	No	No	Yes	31	Alive	No info
FFPE	AD1154	34T	52	Female	pT3	N0	IIIA	No	No	No	35	Alive	No info
FFPE	AD1155	35T	41	Female	pT3	N0	IIIA	No	No	No	28	Alive	Moderate
FFPE	AD1156	36T	56	Male	pT3	N0	IIIA	Yes	No	No	33	Alive	Moderate
FFPE	AD1157	37T	49	Female	pT4	N0	IVA	Yes	No	No	32	Alive	Moderate
FFPE	AD1158	38T	57	Male	pT2	N1	IIIB	No	No	No	62	Alive	Poor
FFPE	AD1159	39T	58	Female	pT2	N0	II	No	No	No	39	Dead	Poor
FFPE	AD1160	40T	48	Female	pT2	N0	II	No	No	No	61	Alive	Moderate
FFPE	AD1161	41T	41	Female	pT2	N1	IIIB	Yes	No	No	66	Alive	Moderate
FFPE	AD1162	42T	52	Female	pT2	N0	II	No	No	No	15	Dead	Poor
FFPE	AD1163	43T	47	Female	pT2	N1	IIIB	No	No	No	33	Dead	Moderate
FFPE	AD1164	44T	57	Female	pT3	N1	IIIB	No	No	No	41	Alive	Poor

Table S6: IHC scores for ERBB2 amplification in gallbladder samples (n=25)

Sr.No.	Sample ID	IHC Score by Pathologist 1	IHC Score by Pathologist 2	Remarks
1	19T	3	3	95% membranous
2	24T	2	3	95% membranous
3	20T	2	3	95% membranous
4	28T	2	2	60% membranous
5	34T	2	2	95% membranous
6	39T	2	2	
7	22T	1	2	70% membranous
8	27T	1	3	95% membranous
9	33T	1	3	95% membranous
10	38T	1	1	95% membranous (less intensity)
11	25T	1	0	Very weak (<1%)
12	18T	0	1	60% membranous
13	29T	0	1	Very weak (mostly cytoplasmic)
14	31T	0	0	
15	44T	0	3	95% membranous
16	21T	0	2	95% membranous
17	43T	0	2	30% membranous
18	32T	0	2	70% membranous
19	30T	0	2	95% membranous
20	40T	0	1	
21	26T	0	1	80% membranous
22	37T	0	0	
23	41T	0	0	
24	36T	0	0	
25	23T	0	0	

Table S7: Primers for validation of alterations

Primer	(5'-3')
OAD1089 _CTNNb1 F_S37C_Forward	TTTGATGGAGTTGGACATGG
OAD1090 _CTNNb1 R_S37C_Reverse	CCTCAGGATTGCCTTTACCA
OAD1091_CTNNB1F_S33C_Forward	AGCTGATTTGATGGAGTTGGA
OAD1092_CTNNB1R_S33C_Reverse	CACTCAGAGAAGGAGCTGTGG
OAD_1093_MLL3F_C1114R_Forward	TCATAGCCCACAGGGAAGAG
OAD_1094_MLL3R_C1114R_Reverse	GATTGCTAGCATCGTGCAAC
OAD_1095_SMAD4F_R265H_Forward	TGAAATGGATGTTCAGGTAGGA
OAD_1096_SMAD4R_R265H_Reverse	TCAATGGCTTCTGTCCTGTG
OAD1097_ERBB2F_I767M_Forward	ATCCCTGATGGGGAGAATGT
OAD1098_ERBB2R_I767M_Reverse	GGGTCCTTCCTGTCCTCCTA
OAD1098_ERBB2F_V777L_Forward	GAGGCTGTGTGGTGTTTGG
OAD1099_ERBB2R_V777L_Reverse	CGTGGATGTCAGGCAGATG
OAD1401_ERBB2_I655V_Forward	ACCCCAAACTAGCCCTCAAT
OAD1402_ERBB2_I655V_Reverse	AGGGGGTGGTGGGTCAGT
OAD_583_TP53_H193L Forward	AGGCCCTTAGCCTCTGTAAG
OAD_584_TP53_H193L Reverse	TGCAGCTGTGGGTTGATTCC
OAD1105_ERBB3F_R1127H_Forward	GAGTCATCAGAGGGGCATGT
OAD1106_ERBB3R_R1127H_Reverse	GGGAATGGTAGGCGCTATCT
OAD1407_TP53_V217G Forward	CTTAACCCCTCCTCCCAGAG
OAD1408_TP53_V217G Reverse	CATGAGCGCTGCTCAGATAG
OAD1409_KRAS_G12V Forward	TTGGATCATATTCGTCCACAA
OAD1410_KRAS_G12V Reverse	TTTTCATGATTGAATTTTGTAAGG
OAD 1411_CTNNB1_R582W Forward	AGGAGAATGCCCTGTTTGTT
OAD 1412_CTNNB1_R582W Reverse	TTATGGTCCCTAATTTTCTGAAATG
OAD 1413_SMAD4_D441G Forward	CGGATTACCCAAGACAGAGC
OAD 1414_SMAD4_D441G Reverse	CTAGGAGCAAGGCAGCAAAC
OAD1401_ERBB2_I655V_Forward	ACCCCAAACTAGCCCTCAAT
OAD1402_ERBB2_I655V_Reverse	AGGGGGTGGTGGGTCAGT
OAD1545_EGFR _I1050V_Forward	ATACCCTCCATGAGGCACAC
OAD1546_EGFR_ I1050V_Reverse	CCAGGAGTCACGCTTTGAAC

Table S8 : STR Profiling of gallbladder cancer cell lines

	AMEL		D5S818		D13S317		D7S820		D16S539		vWA		TH01		TPOX		CSF1PO		D21S11		Analysis
G415	X	X	12		8	10	11	12	12		16		7		11		12		32.2		Matched -Riken cell bank
NOZ	X	X	11	13	8	12	10	11	9	11	19		7	9	8	11	11	13	30	31	Matched-Japanese Cell bank (JCRB)
OCUG1	X	X	10	12	8	11	10	11	9	13	16		7	9.3	8	11	10	12	28	29	Matched-Japanese Cell bank(JCRB)
SNU308	X	X	9		8		7	12	10	13	16		8		8		12		30	31	Matched Korean Cell bank(KCLB)
TGBCT2	X	X	11		12		10	11	9	10	16		6	6	8		12	13	32.2		Matched- Riken cell bank

Table S9 : Tumor volume of mice during the course of treatment

			Tumor volume					
		Mouse No.	Day 13	Day 16	Day 19	Day 22	Day 25	Day 28
G415	Vehicle	1243	112	146	237	267	222	239
		1597	96	88	143	498	490	478
		1605	183	226	238	285	450	750
		1606	153	223	172	311	427	550
		1599	144	203	179	424	384	533
		1607	112	93	94	126	216	505
	Drug	1245	94.28	150.91	116.45	78.04	122.29	73
		1598	113	102	79	77	75	77
		1600	92	124	86	85	89	70
		1601	142	287	200	251	453	1225
		1602	99	71.3	*			
		1603	87	118	65	47	40	42
		1604	118	65	47	40	41	40

*Mice dead

			Tumor volume					
		Mouse No.	Day 6	Day 9	Day 12	Day 15	Day 18	Day 21
NOZ	Vehicle	1372	166	49	173	250	519	446
		1374	96	135	133	215	572	465
		1609	176	350	740	1324	2113	1933
		1371	137	324	423	673	1769	*
	Drug	1373	133.52	353.25	452.05	768	608	836
		1375	114.74	207.1	330.33	401	280	494
		1376	114.82	224.66	478.22	694	681	1050
		1610	90	137	180	314	563	670
		1611	97	163	280	367	579	729
		1608	95	121	275	651	765	1397

*Mice dead

			Tumor volume																	
	Mouse No.	Day 0	Day 3	Day 6	Day 9	Day 12	Day 15	Day18	Day21	Day 24	Day 28	Day 31	Day 34	Day 37	Day 40	Day 43	Day 46	Day 49	Day 51	Day 54
OCUG1	1383	X	X	X	X	X	X	X	X	X	X	X	X	X	X	X	X	X	X	X
	1384	X	X	X	X	X	X	X	X	X	X	X	X	X	X	X	X	X	X	X
	1385	X	X	X	X	X	X	X	X	X	X	X	X	X	X	X	X	X	X	X
	1537	X	X	X	X	X	X	X	X	X	X	X	X	X	X	X	X	X	X	X
	1538	X	X	X	X	X	X	X	X	X	X	X	X	X	X	X	X	X	X	X
	1539	X	X	X	X	X	X	X	X	X	X	X	X	X	X	X	X	X	X	X
	1825	X	X	X	X	X	X	X	X	X	X	X	X	X	X	X	X	X	X	X
	1826	X	X	X	X	X	X	X	X	X	X	X	X	X	X	X	X	X	X	X
	1827	X	X	X	X	X	X	X	X	X	X	X	X	X	X	X	X	X	X	X
	1830	X	X	X	X	X	X	X	X	X	X	X	X	X	X	X	X	X	X	X

X- No tumor
